# BIM and NOXA are mitochondrial effectors of TAF6δ-driven apoptosis

**DOI:** 10.1038/s41419-017-0115-3

**Published:** 2018-01-22

**Authors:** Aurélie Delannoy, Emmanuelle Wilhelm, Sebastian Eilebrecht, Edith Milena Alvarado-Cuevas, Arndt G Benecke, Brendan Bell

**Affiliations:** 1 RNA Group, Département de microbiologie et d’infectiologie, Faculté de médecine et sciences de la santé, Université de Sherbrooke and Centre de recherche du CHUS, Sherbrooke, QC Canada; 2 CNRS UMR8246, Université Pierre et Marie Curie, 75005 Paris, France; 3 ACSIOMA GmbH, Technologiezentrum Ruhr, 44799 Bochum, Germany; 4 Center for Innate Immunity and Immune Disease, University of Washington School of Medicine, Seattle, WA 98195 USA

## Abstract

TAF6δ is a pro-apoptotic splice variant of the RNA polymerase II general transcription factor, TAF6, that can dictate life vs. death decisions in animal cells. TAF6δ stands out from classical pro-apoptotic proteins because it is encoded by a gene that is essential at the cellular level, and because it functions as a component of the basal transcription machinery. TAF6δ has been shown to modulate the transcriptome landscape, but it is not known if changes in gene expression trigger apoptosis nor which TAF6δ-regulated genes contribute to cell death. Here we used microarrays to interrogate the genome-wide impact of TAF6δ on transcriptome dynamics at temporal resolution. The results revealed changes in pro-apoptotic BH3-only mitochondrial genes that correlate tightly with the onset of cell death. These results prompted us to test and validate a role for the mitochondrial pathway by showing that TAF6δ expression causes cytochrome c release into the cytoplasm. To further dissect the mechanism by which TAF6δ drives apoptosis, we pinpointed BIM and NOXA as candidate effectors. siRNA experiments showed that both BIM and NOXA contribute to TAF6δ-dependent cell death. Our results identify mitochondrial effectors of TAF6δ-driven apoptosis, thereby providing the first of mechanistic framework underlying the atypical TAF6δ apoptotic pathway’s capacity to intersect with the classically defined apoptotic machinery to trigger cell death.

## Introduction

Apoptosis represents a genetically programmed form of cellular suicide that is crucial for normal development and homeostasis in multicellular organisms. The TAF6δ pathway of apoptosis can control cell death decisions^1–4^, but its emerging properties distinguish it from other classical apoptotic pathways such as the Bcl-2 family, the caspase family, the death receptor pathway, or the p53 pathway. Classical pro-apoptotic genes, including tumor suppressors (e.g., p53, RB1, and APC) or members of the core apoptotic machinery (e.g., caspases, Bcl-2 family members, and death receptors) have been shown to be non-essential at the cellular level^5^. In stark contrast to these classical apoptotic pathways, the TAF6δ pathway hinges on the expression of the *TAF6* gene that is essential for cellular viability from yeast to humans^1,5^. We therefore refer to TAF6δ as the prototypical member of type E (essential) pro-apoptotic proteins, to distinguish it from traditional type NE (non-essential) pro-apoptotic proteins that include the caspases, Bcl-2 family members, p53, and the death receptors. Another atypical feature of the TAF6δ pathway is that it involves coupling cell signaling pathways to cell death via subunit changes in the RNA polymerase II (Pol II) general transcription factor (GTF), TFIID^2,6^. In contrast, other pro-apoptotic transcription factors, such as the p53 tumor suppressor, act primarily as gene-specific DNA-binding proteins^7^.

TFIID is a multi-protein complex composed of TATA-binding protein (TBP) and 13 TBP associated factors (TAFs)^8^. TFIID plays a well-established role in the recognition of Pol II core promoter elements, cell cycle control, and the recognition of certain modified histones^9,10^. Once TFIID is assembled upon the core promoter, it forms a scaffold for pre-initiation complex (PIC) assembly that allows transcriptional activation. More recently, the TAFs have been shown to play a role in the establishment and maintenance of pluripotency in stem cells^11^. Recently, mutations in the histone-fold domain of the core TFIID subunit TAF6 were linked to neurogenetic disorders in humans^12,13^. In addition to the canonical form of TFIID, tissue-specific or signal-responsive TFIID subunits can be incorporated into functionally distinct PICs that contribute to the combinatorial control of gene expression^14–16^.

TAF6δ is a minor inducible splice variant of the TFIID subunit TAF6 whose expression drives apoptosis^2–4^. The major isoform of TAF6, TAF6α, is constitutively expressed in all cell types under normal culture conditions. In contrast, TAF6δ is not expressed under normal conditions, but can be induced experimentally using antisense splice-switching oligonucleotides (SSOs)^3^, or under specific pro-apoptotic conditions^2^. TAF6δ is produced via the alternative splicing of *taf6* pre-mRNA that results in the loss of 10 amino acids in the second α-helix of its histone-fold domain^1,3^. TAF6δ therefore cannot interact with the normal dimerization partner of TAF6α, TAF9^2^. Consequently, TAF6δ incorporates into a TFIID complex lacking TAF9 termed TFIIDπ that drives apoptosis. Transcriptome analysis revealed that TAF6δ specifically regulated the expression of ~1000 genes, of which more than 90% were upregulated^4^. Gene ontology analysis of the TAF6δ-induced transcriptome signature showed an over-representation of the Notch, oxidative stress response, integrin, p53, apoptosis, p53 feedback loop, and angiogenesis cellular signaling pathways^4^.

The TAF6δ pathway intersects the p53 signaling hub both directly and indirectly. p53 acts primarily as a transcription factor that regulates gene expression to control cell cycle arrest and induce apoptosis^7^. Both the major TAF6α isoform^17^ and its pro-apoptotic TAF6δ counterpart^4^ make direct physical contacts with the *trans*-activation domain of p53. In vitro the TAF6α-p53 interaction is essential for transcriptional activation by p53^17^. Likewise, in vivo the TAF6α-p53 interaction is important for the apoptotic and tumor suppressive functions of p53^18,19^. The presence of TAF6α vs. TAF6δ can influence the activation of target genes, and the presence or absence of p53 can influence the regulation of certain TAF6δ-dependent genes^4^. Importantly, the pro-apoptotic function of TAF6δ is independent of p53^3^. Since the mechanisms by which TAF6δ triggers apoptosis are currently unknown, we have focused here on defining downstream molecular events that underlie TAF6δ-driven cell death.

The two most extensively understood pathways of apoptosis are the extrinsic and intrinsic pathways. The extrinsic pathway is induced by extracellular cell death ligands, such as TRAIL or FAS, and proceeds via ligand-dependent formation of the death-inducing signaling complex that leads to cleavage and activation of the initiator caspase-8^20^. The intrinsic pathway of apoptosis is initiated by intracellular signals, such as DNA damage, that proceeds by permeabilization of the mitochondrial outer membrane, allowing the key event of release of cytochrome c into the cytoplasm to form the apoptosome^21^. Here we employed temporal analysis of TAF6δ-driven transcriptome dynamics to identify positive effector genes of TAF6δ-dependent cell death. Our data show that TAF6δ triggers cell death primarily via the intrinsic pathway, and that BIM and NOXA contribute functionally to TAF6δ-dependent apoptosis.

## Results

### TAF6δ drives dynamic transcriptome changes

**Fig. 1:**
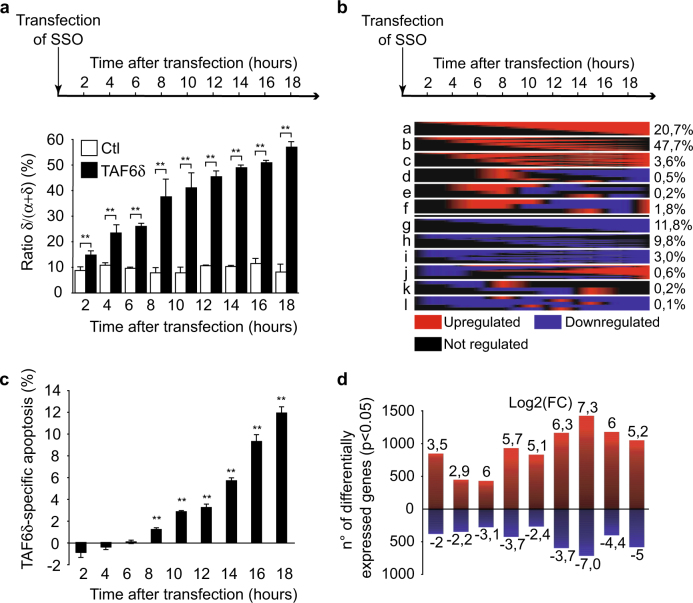
Time-course analysis of TAF6δ-induced cell death HeLa cells were transfected with 100 nM control or TAF6 SSO to induce TAF6δ expression and harvested every 2 h after the transfection. The samples were submitted to apoptosis measurements, RT-PCR, and transcriptome analysis by cDNA microarray. **a** Shift of TAF6 splicing pattern upon SSO transfection. A portion of the endogenous TAF6 mRNA was amplified by RT-PCR and the PCR products were quantified by capillary electrophoresis (*n* = 3). The histogram bars represent the percentage of TAF6δ isoform relative to total TAF6 mRNA and error bars indicate the S.D. of three independent experiments. Statistical significance compared to the control was assessed with Student’s *t*-test: *p < 0.05; **p < 0.01. **b** Classification of TAF6δ-regulated genes depending on their expression profiles. The proportion of each group of genes relative to the total number of genes (5320) is indicated on the right. **c** Quantification of the apoptosis specifically induced by the δ isoform. Apoptosis was measured in scrambled and TAF6 SSO-transfected cells by the detection of cleaved cytokeratin-18 in flow cytometry and the difference (in percentage of cells) between both is represented. The error bars indicate the S.D. for three independent experiments assayed by three technical replicates. Means and S.D.’s for the biological replicates were calculated using the three means of the three technical replicates of each biological replicate. Statistical significance was assessed with the Student’s *t*-test: *p < 0.05; **p < 0.01. **d** Heatmaps representing the probes regulated by TAF6δ in a statistically significant manner (*p* < 0.05) at each point of the time-course. The red color represents a positive regulation and the blue a negative one. The size of each heatmap is proportional to the number of regulated genes (*p* < 0.05) and the maximum and minimum logQ values are indicated

To dissect the molecular mechanisms by which the transcription factor TAF6δ induces apoptosis, we employed a systems biology approach. We reasoned that the expression of functionally relevant pro-apoptotic effectors of TAF6δ would correlate closely in time with the induction of TAF6δ-induced cell death. To identify such genes, we employed genome-wide microarray analysis and induced the expression of TAF6δ in HeLa via transfection of SSOs as we have previously described^3,4,22^. We verified the induction of TAF6δ mRNA levels by reverse transcription-PCR (RT-PCR) (Fig. 1a) and analyzed apoptosis levels by flow cytometry (Fig. 1c). As expected, SSO transfection caused steadily increased levels of TAF6δ mRNA (Fig. 1a). Increased levels of apoptosis achieved statistical significance 8 h after TAF6δ induction and continued to increase until 18 h post induction (Fig. 1c). The transcriptome dynamics were recorded and analyzed using the ace.map algorithm^23^ to quantitate and classify the time-course of gene expression changes. In total, 5320 transcripts displayed statistically significant TAF6δ-dependent regulation (Supplementary Table 1) and the 3 most common classes of changes were transcripts transiently upregulated (Fig. 1b, class b; 47.7% of changes), transcripts whose expression increased constantly throughout the time-course (Fig. 1b, class a; 20.7%), and transcripts whose expression decreased constantly during the time-course (Fig. 1b, class g; 11.8%). More complex expression patterns were detected, albeit with much lower frequencies (Fig. 1b). To further separate transient and evolving expression profiles, we also performed an overlap analysis for all time points compared to the last 18 h time point. The results show increases in the number of genes, and the statistical significance of the overlaps, with the latest time point progressively throughout the time-course (Supplementary Fig. 1). Significant changes were detected at all time points with the largest number of regulated genes detected at 14 h post transfection (Fig. 1d). Throughout the time-course more genes were upregulated than downregulated, reinforcing our previous observations^3,4^ showing that TAF6δ acts primarily to activate gene expression (Fig. 1d).

**Fig. 2:**
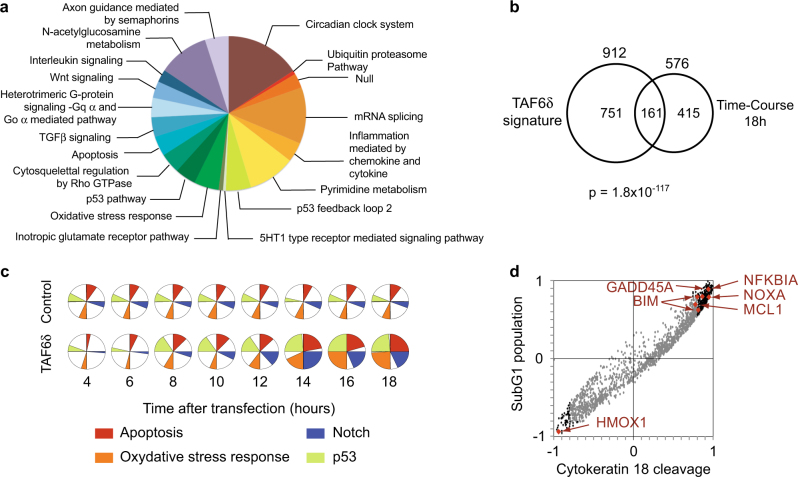
Ontology analysis of microarray data reveals a correlation of apoptotic gene expression and apoptosis-related pathway regulation with TAF6δ induction **a** Representation of the pathways regulated by TAF6δ along the time-course with the highest significance. The size of the slice is proportional to the ratio of observed versus expected numbers of genes with of given ontology. **b** Venn diagram representation of the overlap between TAF6δ-regulated genes at time point 18 h of the time-course and previous independent microarray data^4^. **c** Enrichment of four pro-apoptotic pathways in TAF6δ-regulated genes along the time compared to the control. The dark slices represent the ratio −log10(*p*value)/−log10(*p*value_Max_) that reflects the relative statistical significance of the enrichment at each time point. **d** Correlation between the expression of TAF6δ-regulated genes and apoptosis measurements was determined by Pearson method. Correlation coefficients obtained for the two apoptosis quantification assays are aligned. Gray dots stand for genes with −0.8 < *R*^2^ < 0.8; Black dots for highly correlating dots (l*R*^2^l > 0.8) and red dots for probes of interest

To further dissect the transcriptome impact of TAF6δ over time, we applied gene ontology analysis to the complete set of regulated genes. We found that many of the pathways we previously identified at a single 18 h time point^4^ were over-represented, including oxidative stress, the p53 pathway, and importantly apoptosis (Fig. 2a). In addition, the much more extensive analyses performed here, including multiple early time points, allowed the detection of the enrichment of genes in other new pathways, for example, the transforming growth factor-β and Wnt signaling pathways (Fig. 2a). We further compared the transcriptome changes at the 18 h time point with our previous independent experimental analysis. The data show a statistically significant overlap, confirming the reproducibility of the microarray analysis (Fig. 2b). We next followed four key pathways that we had previously shown to be over-represented upon TAF6δ induction, including the Notch, p53, oxidative stress, and apoptosis pathways^4^. No statistically significant changes were observed for these pathways when cells were treated with negative control SSO, but as expected they were regulated when TAF6δ expression was induced (Fig. 2c). Enrichment of genes within the apoptosis and p53 pathways closely correlated in time with the induction of apoptosis (Figs. 1c and 2c). Genes in the oxidative stress and Notch pathways lagged slightly behind the induction of apoptosis (Fig. 2c). The complete ontology analysis for each time is shown in Supplementary Table 2. We next used a correlation analysis to pinpoint TAF6δ-induced genes whose expression correlated tightly with the levels of apoptosis. Genes that displayed positive correlation with apoptosis levels included *NFKB1A*, *GADD45A*, *BIM*, *MCL1*, and *NOXA* (Fig. 2d). Interestingly, heme oxygenase displayed the strongest anti-correlation with apoptosis levels and has well-documented anti-apoptotic activity^24^. The complete list of genes with a correlation coefficient of 0.8 or higher is shown in Supplementary Table 3. The transcriptomic analysis revealed dynamic changes in gene expression programs upon induction of TAF6δ. The first clue as to the mechanism of action of TAF6δ that emerged from the data was the fact that the induction of genes with established roles in the mitochondrial pathway of apoptosis correlated closely temporally with the levels of apoptotic cell death.

### TAF6δ acts via the mitochondrial pathway of apoptosis

**Fig. 3:**
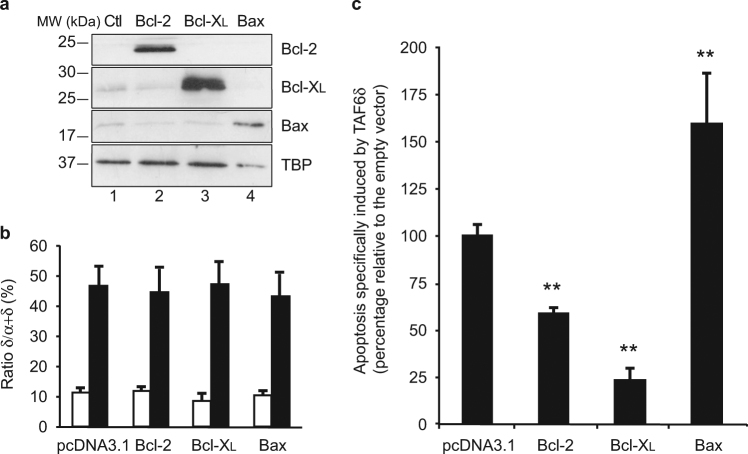
Blockade of the mitochondrial pathway by overexpression of the anti-apoptotic proteins Bcl-2 and Bcl-X_L_ interferes with TAF6δ-induced apoptosis HeLa cells were transfected with either pcDNA3.1-Bcl-2, pcDNA3.1-Bcl-X_L_, pcDNA3.1-Bax, or empty pcDNA3.1 vector, and 16 h later with scrambled or TAF6 SSO. Cells were collected 18 h after the second transfection to carry apoptosis and protein overexpression assessment. **a** Confirmation of Bcl-2, Bcl-X_L_, and BAX overexpression by western blot in HeLa cells. **b** The shift of TAF6 splicing pattern upon control (white bars) or TAF6 (black bars) SSO transfection. Endogenous TAF6 was amplified from cDNA by PCR, and PCR products were quantified by capillary electrophoresis (Agilent). The histogram represents the percentage of TAF6δ isoform relative to total TAF6 mRNA with error bars representing the S.D. for three independent experiments. Statistical significance of the differences of induction of the δ isoform was assessed with Student’s *t*-test by comparing each plasmid transfection to the empty vector control: *p < 0.05; **p < 0.01. **c** Quantification of apoptosis specifically induced by the δ isoform. Apoptosis was measured in scrambled and TAF6 SSO-transfected cells and the difference between TAF6δ-expressing cells and control was normalized to the empty vector (mean ± S.D.) for three independent experiments. Technical replicates were performed for each transfection and the means and S.D.’s for the biological replicates were calculated using the three means of the three technical replicates of each biological replicate. Statistical significance was assessed with Student’s *t*-test: *p < 0.05; **p < 0.01

Based on the dynamic TAF6δ-driven transcriptome landscape recorded above, we hypothesized that TAF6δ could act via the mitochondrial pathway of apoptosis. To determine whether the mitochondrial pathway is important for TAF6δ-dependent cell death, we overexpressed the anti-apoptotic Bcl-2 family members Bcl-2 and Bcl-X_L_ to prevent the release of cytochrome c from the mitochondria^25–27^. Bcl-2, Bcl-X_L_, and Bax were transiently overexpressed in HeLa cells, and their protein levels were verified by western blot analysis (Fig. 3a). TAF6δ levels were induced in the overexpressing cells and confirmed by PCR (Fig. 3b). Apoptosis levels were quantitated by flow cytometry and showed that both Bcl-2 and Bcl-X_L_ expression caused a statistically significant decrease in apoptosis (Fig. 3c). Expression of the pro-apoptotic Bax protein was included as a positive control and showed the opposite effect with increased apoptosis as expected (Fig. 3c). The inhibition of TAF6δ-directed cell death by Bcl-2 and Bcl-X_L_ implicates the mitochondrial pathway as an important contributor to the TAF6δ pathway of cell death.

**Fig. 4:**
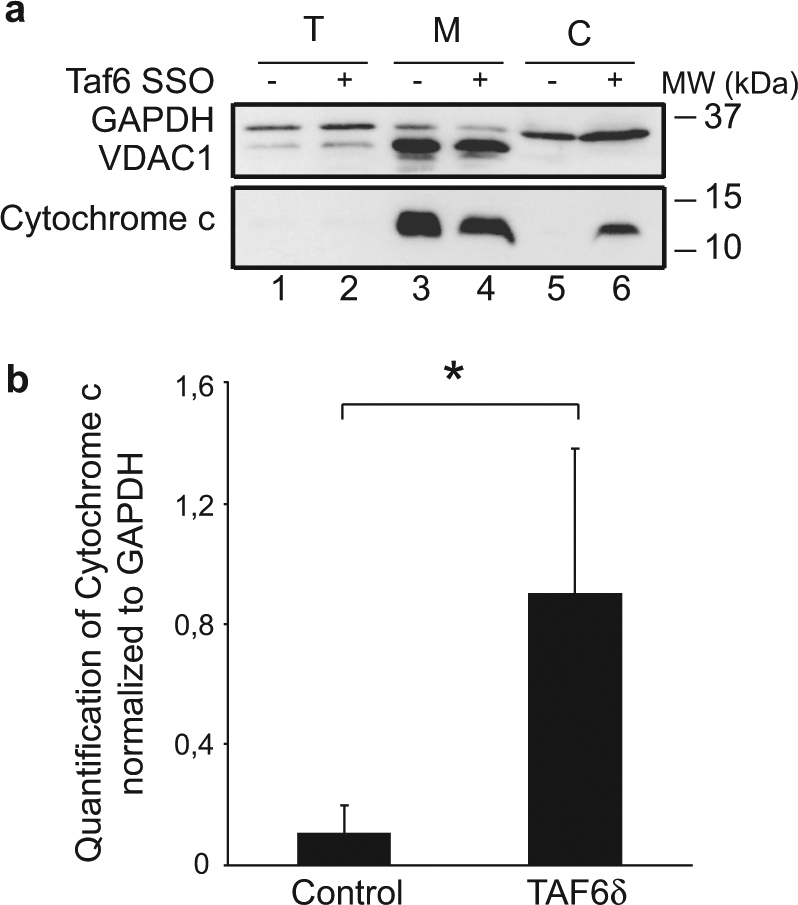
Cytochrome c release observed by cell fractionation in TAF6δ-expressing HeLa cells HeLa cells were transfected with 100 nM scrambled or TAF6 SSO and collected 18 h later. Those samples were subjected to cell fractionation. Then, the total, mitochondrial, and cytoplasmic fractions were used in western blots. **a** Western blot showing the cytochrome c release from the mitochondrial fraction (M) to the cytoplasmic fraction (C) of HeLa cells transfected with TAF6δ (+) or scrambled (−) SSO. The total fraction of those cells (T) was also used as a control. The immunoblot shown here is representative of three experiments. **b** Quantification of the signals (mean ± S.D.) for the three independent experiments performed. Statistical significance was assessed by the Student’s *t*-test: *p < 0.05; **p < 0.01

To obtain direct biochemical evidence that the mitochondrial pathway of cell death is triggered by TAF6δ, we performed cell fractionation experiments to measure the release of cytochrome c from the mitochondrial fraction into the cytoplasmic fraction^28^. TAF6δ-dependent cell death was induced by transfection with SSO, and the presence of cytochrome c and control proteins in cell fractions was monitored by immunoblotting. The efficiency of the fractionation was validated using antibodies against the known mitochondrial protein VDAC-1 that was found in the mitochondrial but not cytoplasmic fraction (Fig. 4a, lanes 3 and 4 vs. 5 and 6). As expected, cytochrome c was found in the mitochondrial fraction (Fig. 4a, lane 3), but not in the cytoplasmic fraction of untreated cells (Fig. 4a, lane 5). Upon induction of endogenous TAF6δ, cytochrome c became readily detectable in the cytoplasmic fraction (Fig. 4a, lane 6). The release of cytochrome c into the cytoplasm during TAF6δ-dependent cell death was reproducible and statistically significant upon quantitation of triplicate experiments (Fig. 4b).

**Fig. 5:**
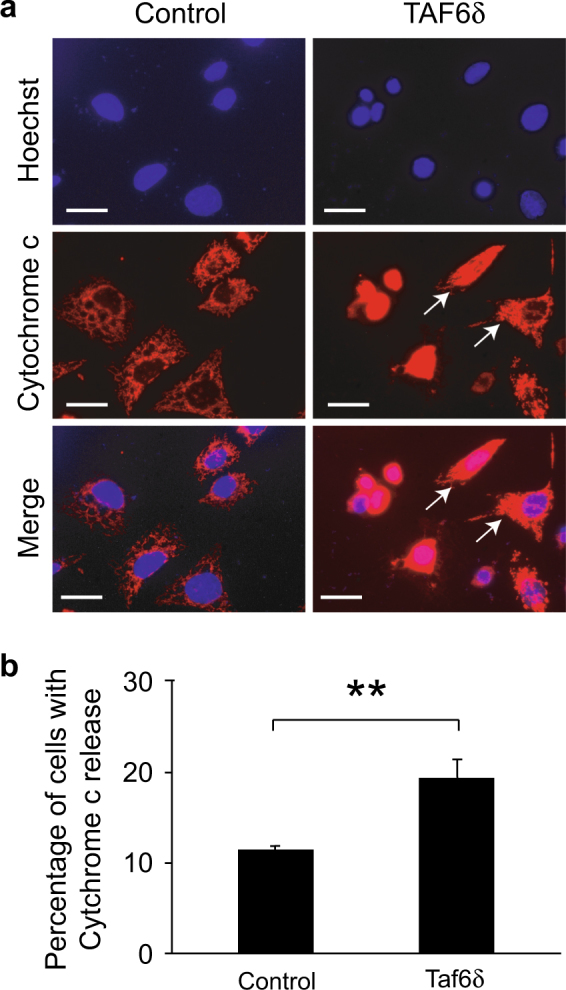
Immunofluorescence visualization of cytochrome c delocalization HeLa cells were transfected with 100 nM scrambled or TAF6 SSO and immunofluorescence detection of the cytochrome c was performed 18 h later. **a** Immunofluorescence detection of cytochrome c in control and TAF6 SSO-transfected cells. White arrows show cells undergoing early mitochondrial structure perturbation. Scale bar represents 25 µm. **b** Quantification of cells presenting a delocalization of cytochrome c in SSO-transfected cells (mean ± S.D.) for three independent experiments. Technical replicates were performed for each transfection and means and S.D.’s for the biological replicates were calculated using the three means of the three technical replicates of each biological replicate. Statistical significance was assessed by the Student’s *t*-test: *p < 0.05; **p < 0.01

We next used immunofluorescence as an independent experimental approach that allows visualization of cytochrome c subcellular localization in individual cells. As expected, control cells displayed cytochrome c organized characteristically within the mitochondria (Fig. 5a). In contrast, cell populations where TAF6δ was induced showed statistically significant (Fig. 5b) increases in the number of cells where cytochrome c localized throughout the cytoplasm (Fig. 5a, b). We note that the release of cytochrome c was observed in cells early in the apoptotic process before the onset of gross morphological changes of apoptosis, such as nuclear fragmentation (Fig. 5a, white arrows). This observation excluded the possibility that the re-localization of cytochrome c could simply be due to late apoptotic morphological changes. These data provide further evidence that cytochrome c release into the cytoplasm, the key event in the mitochondrial pathway of death, is triggered by TAF6δ.

To establish the extent to which the mitochondrial pathway of apoptosis is triggered in other cell types, we established cell lines of different origins overexpressing Bcl-2 and/or Bcl-X_L_ using lentiviral transduction. TAF6δ-dependent apoptosis was induced via SSO transfection and apoptosis was measured by flow cytometry. HeLa cervical carcinoma, MDA-MB-231 breast adenocarcinoma, Saos-2 osteosarcoma, and Panc-1 pancreas ductal carcinoma cells all displayed statistically significant reductions in TAF6δ-dependent apoptosis when the mitochondrial pathway was blocked via overexpression of anti-apoptotic Bcl-2 proteins (Supplementary Fig. 2). These data suggest that the contribution of the mitochondrial pathway to TAF6δ-driven cell death is not limited to HeLa but occurs in a broad range of cell types. Based on the above overexpression, biochemical, and immunofluorescence data, we conclude that TAF6δ triggers the mitochondrial pathway of apoptosis.

### BIM and NOXA are effectors of TAF6δ-driven cell death

Having established that the mitochondria pathway of apoptosis is triggered by TAF6δ, we next parsed the transcriptome data to identify candidate TAF6δ-induced apoptotic effector genes. To this end, we used the Panther database^29^ over-representation test to identify Reactome pathways (http://www.reactome.org) that were over-represented within the subset of 262 mRNAs whose induction most tightly paralleled the temporal onset of apoptosis (Pearson correlation coefficient > 0.8, Fig. 2d; Supplementary Table 3). Within the broad category of *programmed cell death*, four sub-pathways within the *intrinsic pathway for apoptosis* were over-represented (Supplementary Table 4). Two genes were responsible for the over-representation of the four sub-pathways of the intrinsic pathway, namely *BIM* and *NOXA* (Supplementary Table 4). Both BIM and NOXA are pro-apoptotic mitochondrial BH3 domain-only members of the Bcl-2 family that promote the permeabilization of the mitochondrial outer membrane, the known commitment point to cell death via the intrinsic pathway^30^. Based on the facts that TAF6δ causes the release of cytochrome c from the mitochondria (Figs. 4 and 5), that TAF6δ induces BIM and NOXA expression with a timing that closely parallels the levels of apoptosis (Fig. 2d), and that BIM and NOXA have established roles in the permeabilization of the mitochondrial outer membrane^30^, we asked whether these genes could contribute functionally to TAF6δ-driven cell death.

**Fig. 6:**
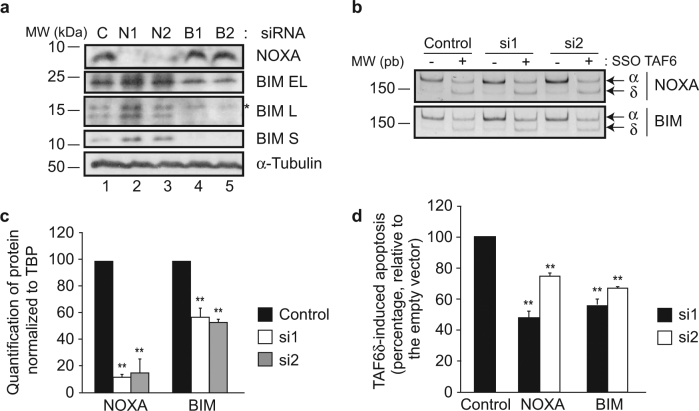
Inhibition of BIM or NOXA expression impairs TAF6δ-induced apoptosis HeLa cells were transfected with 10 nM DsiRNA and 24 h later with 100 nM control or TAF6 SSO. Cells were collected 18 h later for apoptosis measurements. **a** Validation of NOXA and BIM DsiRNA-mediated knockdown. Immunoblot detection of NOXA, BIM, and α-tubulin was performed in HeLa cells transfected with either control (lane 1), NOXA Dsi1 (lane 2), NOXA Dsi2 (lane 3), BIM Dsi1 (lane 4), or BIM Dsi2 (lane 5) after 48 h. **b** Shifting of TAF6 splicing pattern upon SSO transfection. Endogenous TAF6 was amplified from cDNA by PCR, and PCR products were visualized on polyacrylamide gel. The top panel shows samples from HeLa cells transfected with either control, NOXA Dsi1, or Dsi2 RNA. Bottom panel show samples from HeLa cells transfected with either control, BIM Dsi1, or Dsi2 RNA. **c** Quantification of NOXA and BIM protein expression in scrambled (black bars), Dsi1 (white bars), or Dsi2 (gray bars) transfected cells normalized to TBP (mean ± S.D.). This histogram summarizes three independent experiments. Technical replicates were performed for each independent experiment and the means and S.D.’s for the biological replicates were calculated using the three means of the three technical replicates of each biological replicate. Statistical significance was assessed by the Student’s *t*-test: *p < 0.05; **p < 0.01. **d** Quantification of apoptosis specifically induced by the δ isoform. Apoptosis was measured in scrambled and TAF6 SSO-transfected cells, and the difference between TAF6δ-expressing cells and control was normalized to the control siRNA. Black bars represent Dsi1, white bars are for Dsi2. Statistical analysis as in **c**

To test whether the expression of BIM and NOXA impact TAF6δ-dependent apoptosis, we reduced their protein levels using siRNA transfection in HeLa cells. Two independent siRNAs were used to silence BIM and NOXA expression, and the reduction of protein levels was validated by immunoblotting (Fig. 6a, c). TAF6δ-driven apoptosis was then induced by SSO transfection (Fig. 6b) and the levels of apoptosis were then analyzed by flow cytometry. Depletion of both BIM and NOXA resulted in a statistically significant reduction in the levels of TAF6δ-induced apoptosis with both siRNAs (Fig. 6d). Together, the data reveal that BH3-only mitochondrial proteins are effectors of TAF6δ-driven apoptosis.

**Fig. 7:**
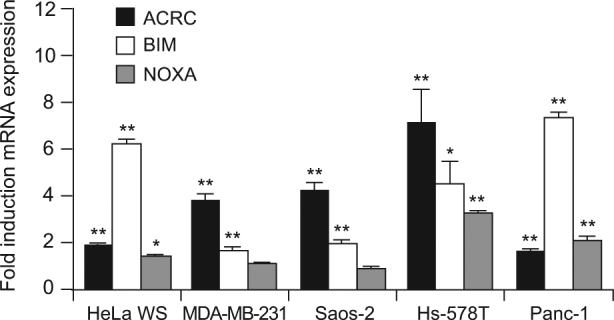
TAF6δ induces transcriptional activation of BIM and NOXA in other cancer cell lines Wild-type cell lines were transfected with 100 nM SSO and samples were collected 18 h later to evaluate BIM (white bars) and NOXA (gray bars) mRNA expression by qPCR. Error bars show the S.D. of three independent experiments. To control TAF6 transactivation, we also assessed ACRC mRNA expression (black bars) (mean ± S.D.) for three independent experiments. Technical replicates were performed for each independent experiment, and the means and S.D.’s for the biological replicates were calculated using the three means of the three technical replicates of each biological replicate. Statistical significance was assessed with the Student’s *t*-test: *p < 0.05; **p < 0.01

Having established that BIM and NOXA are effectors of TAF6δ-driven apoptosis in HeLa cells, we next tested whether the induction of BIM and NOXA occurs in other cell lines in response to TAF6δ. To this end, we induced TAF6δ expression by SSO transfection and measured the levels of BIM and NOXA mRNAs by quantitative RT-PCR. The expression of the *ACRC* gene was included as a positive control since our previous work showed the ACRC mRNA to be highly induced by TAF6δ in different cell lines^3,4^. The analysis showed that BIM was statistically significantly induced in HeLa cervical carcinoma, MDA-MB-231 breast adenocarcinoma, Saos-2 osteosarcoma, Hs-578T breast carcinoma, and Panc-1 cells (Fig. 7, white bars). NOXA mRNA expression was also significantly induced in most cell lines tested, with the exceptions of MDA-MB-231 and Saos-2 where no change was detected (Fig. 7, gray bars). These data suggest that the induction of the identified effector of TAF6δ cell death BIM occurs broadly in distinct cell types. In the case of the TAF6δ effector NOXA, its induction occurs in several types but is not universally induced by TAF6δ in all cell types.

### The extrinsic pathway of apoptosis is dispensable for TAF6δ-mediated cell death

**Fig. 8:**
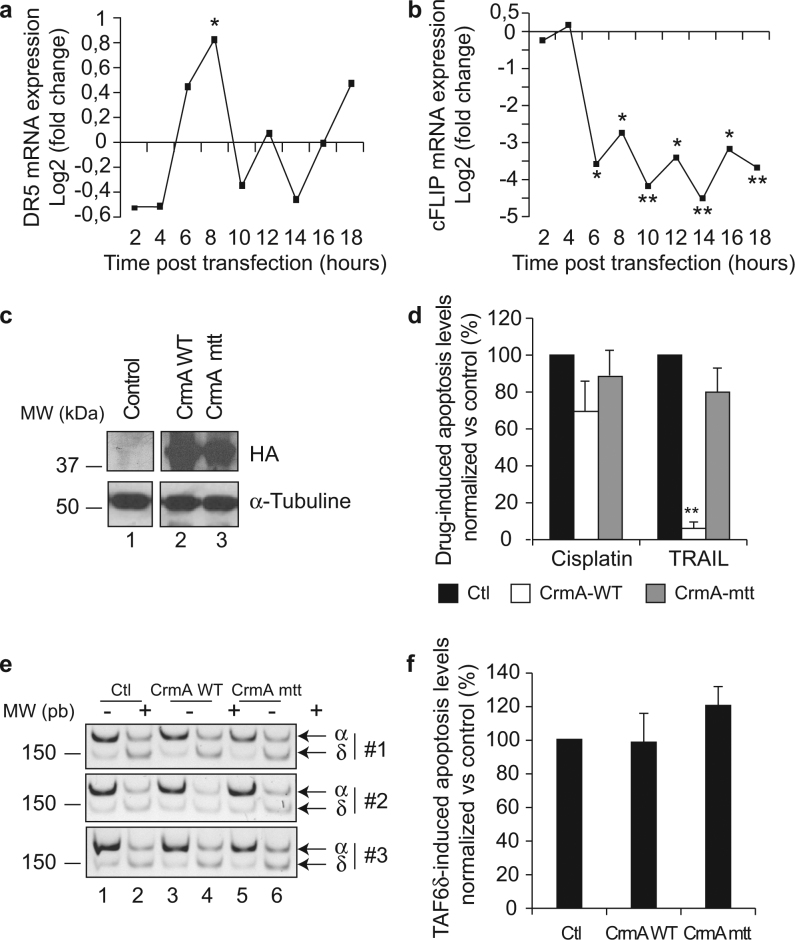
Wild-type CrmA overexpression is sufficient to block TRAIL- but not cisplatin- or TAF6δ-induced cell death Stable cell lines expressing either a wild-type or a mutant version Crm mtt (CrmAT291R) of the viral inhibitor of caspase-8 CrmA were transfected with 100 nM of the scrambled or TAF6 SSO, or treated with TRAIL or cisplatin. **a** The differential expression of DR5/TNFRSF10B between TAF6δ-expressing cells and controls along the time-course was assessed from the microarray data. *p < 0.05; **p < 0.01. **b** The differential expression of c-FLIP/cFLAR between TAF6δ-expressing cells and controls along the time-course was assessed from the microarray data. *p < 0.05; **p < 0.01. **c** The expression of wild-type and mutant CrmA protein was controlled by the detection of their HA-tag by immunoblotting. **d** Quantification of apoptosis induced by cisplatin and TRAIL in control (black bars) and in stable cell lines expressing wild-type (white bars) or mutant Crm mtt (gray bars) versions of CrmA. The bars indicate the average ± S.D. of three independent experiments. Apoptosis levels were determined by technical triplicates for each experiment. The means and S.D.’s for the biological replicates were calculated using the three means of the three technical replicates of each biological replicate, and the statistical significance was determined by the Student’s *t*-test method: *p < 0.05; **p < 0.01. **e** The efficiency of the splicing shift of TAF6 was assessed by RT-PCR. **f** Apoptosis was measured 18 h after TAF6δ induction in control and in HeLa cells expressing wild-type and mutant CrmA. Statistical analysis as in** d**

The above data demonstrate a role for the mitochondrial pathway of apoptosis in TAF6δ-directed cell death, with functional contributions from the TAF6δ-induced genes *BIM* and *NOXA*. Nonetheless, in the time-course transcriptome analysis, certain genes with well-established roles in the extrinsic pathway were regulated by TAF6δ. For example, tumor necrosis factor receptor superfamily member 10B (TNFR10B/DR5/Killer) is a receptor for the death ligand TRAIL^31^ and displayed a wave-like induction in response to TAF6δ (Fig. 8a). Moreover, CFLAR/c-FLIP is a known inhibitor of caspase-8 activation when the extrinsic pathway is triggered, and its expression is strongly downregulated at late time points by TAF6δ (Fig. 8b). We therefore asked whether the extrinsic pathway could play a role in TAF6δ-dependent death. We expressed the cowpox CrmA protein that specifically inhibits caspase-8, thereby blocking the extrinsic pathway^32^. We first verified that CrmA specifically allows discrimination between the extrinsic and intrinsic pathways of apoptosis in our experimental system. HeLa cells were transduced with lentiviral constructs that expressed CrmA or a negative control protein bearing a point mutation T291R that prevents its inhibition of caspase-8^33^. Wild-type and mutated CrmA protein levels were validated by western blotting (Fig. 8c) and specifically and efficiently blocked TRAIL-induced apoptosis, but had no significant effect on the intrinsic pathway of apoptosis induced by cisplatin treatment (Fig. 8d). We next tested the impact of caspase-8 inhibition upon induction of TAF6δ expression (Fig. 8e) and found no detectable inhibition of TAF6δ-induced apoptosis (Fig. 8f). The data show that in this experimental setting the extrinsic pathway of apoptosis is dispensable for TAF6δ-mediated cell death.

## Discussion

TAF6δ is a transcription factor whose expression dictates cell life vs. death decisions, but the underlying mechanisms have remained unknown^3,4^. To define the mechanism by which TAF6δ drives apoptosis, we have performed a transcriptome-wide microarray analysis to temporally dissect gene expression changes during TAF6δ-dependent cell death. The results revealed that genes that correlate temporally with TAF6δ-mediated death include genes with established roles in the permeabilization of the mitochondrial outer membrane (Fig. 2d). Based on this observation, we tested and confirmed a role for the mitochondrial pathway of apoptosis. The overexpression of anti-apoptotic Bcl-2 family proteins blocked TAF6δ-driven apoptosis (Fig. 3), establishing the importance of mitochondrial pathway in TAF6δ-dependent death. Furthermore, TAF6δ-enforced death was accompanied by the release of cytochrome c from the mitochondria as measured by subcellular fractionation (Fig. 4) and immunofluorescent localization of cytochrome c (Fig. 5). These observations establish, for the first time, the importance of the mitochondrial pathway in TAF6δ-induced apoptosis.

Having established the role of the mitochondrial pathway as essential for TAF6δ-dependent cell death, we then identified BIM and NOXA as pro-apoptotic BH3-only genes whose expression correlates temporally with TAF6δ-induced apoptosis. siRNAs were used to demonstrate a functional contribution of BIM and NOXA to TAF6δ-dependent cell death (Fig. 6). BIM and NOXA are induced by TAF6δ and their depletion significantly reduced TAF6δ-driven apoptosis, revealing them as pro-apoptotic effectors of TAF6δ. Although BIM and NOXA are induced by TAF6δ and their expression profiles correlate closely with the induction of apoptosis, it is presently unclear whether BIM or NOXA are direct or indirect targets of TAF6δ. Further work will be required to establish the direct targets of TAF6δ, a challenging task given that TAF6δ is significantly expressed only in cells destined to rapidly undergo apoptosis. It is noteworthy that NOXA expression can be activated by both p53-dependent and -independent apoptotic pathways^34^, a finding compatible with our previous work showing that the pro-apoptotic function of TAF6δ is independent of p53 but shares some overlap on target genes^3^. Our observation that NOXA induction is not a universal feature of TAF6δ-induced apoptosis shows that NOXA induction is not required for death in all cell types. One possible explanation for this finding is simply that the induction of BIM alone can be sufficient for apoptosis. A second possibility is that cell-type-specific TAF6δ-induced genes, potentially other functionally overlapping BH3-only family members^30^, such as PUMA (Supplementary Fig. 3), may contribute to death via the mitochondrial pathway in different cellular contexts. The identification of BIM and NOXA as mitochondrial effectors of TAF6δ represents a fundamental step forward in the understanding of how changes in the subunit composition of the GTF, TFIID, can lead to cell death.

We have focused the current study by applying stringent biological and temporal correlation criteria to identify positive effectors of TAF6δ-dependent apoptosis. Given the complexity of the TAF6δ-directed transcription program, further analysis of our transcriptome data should continue to provide insights into the biological impact of this splice variant. Although we have not pursued them here, reductions in gene expression may also contribute to TAF6δ-driven apoptosis, as exemplified by the drop in mRNA levels of the anti-apoptotic protein heme oxygenase (Fig. 2d). In addition, while we have focused on genes whose expression correlate in a linear manner with apoptosis levels, genes whose mRNA expression is induced in a burst-like manner could also contribute to cell death. For example, the pro-apoptotic BH3 domain-only gene *PUMA*^30^ is induced by TAF6δ transiently only at the 10 h time point and the pro-apoptotic *p53AIP1*^35^ gene is transiently induced at 4 h (Supplementary Fig. 3). Further work will be required to exclude or validate a potential role for transient changes in gene expression as functionally contributing to TAF6δ-induced apoptosis. By more deeply interrogating the transcriptome changes directed by TAF6δ at temporal resolution, the data provided here should also further contribute to our understanding of this pathway beyond the outcome of death that we have explored here. In this vein, we have recently used kinetic ontology enrichment analysis to identify and experimentally validate a functional link between the Notch signaling pathway and the TAF6δ pathway (Alvarado Cuevas et al., unpublished).

The systems biology approach employed here, correlating transcriptome-wide changes in gene expression with a phenotypic outcome, should be applicable to the dissection of other subunit changes in TFIID due to alternative splicing. Such changes in transcription complex composition have biologically important consequences as highlighted by the alternative splicing of the TFIID subunits TAF1^36,37^ and TAF4^38,39^, which have been shown to result in functionally distinct TFIID complexes. More broadly, the strategy we describe herein could contribute to the dissection of any subunit change within transcription factor complexes due to alternative splicing. For example, the functions of as-yet uncharacterized alternative splice variants of subunits of the Mediator complex^40^ could be explored using the methodology we present here.

Drug resistance is a major challenge in the treatment of cancer, and the crippling of apoptotic pathways represents one of the major mechanisms by which tumor cells acquire chemoresistance^41^. One mechanism of chemoresistance is the mutation of the p53 tumor suppressor gene^41^. Elegant genetic studies with transgenic mice have revealed the sobering fact that the restoration of p53 expression in tumors via gene therapy was rapidly overcome by mutation of the non-essential p53 or other genes in the p53 pathway^42,43^. Likewise, mutations within the *BIM* gene can contribute to drug resistance^44^. TAF6 contrasts with previously described pro-apoptotic proteins such as p53, Bcl-2 family members, caspases, and death receptors, which are non-essential (type NE)^5^, as the major isoform of TAF6 protein is essential at the cellular level in human cells^1^. From a therapeutic standpoint, the essential nature of the *TAF6* gene places genetic constraints on the TAF6δ pathway of apoptosis that reduce the possibilities to develop resistance^5^. The essential nature of TAF6 therefore underscores the strategic value in exploring this pathway as a therapeutic target in cancer, since its expression cannot be extinguished to generate resistance.

In conclusion, the data presented here demonstrate a predominant role for the mitochondrial pathway of apoptosis in TAF6δ-mediated cell death. Furthermore, by using temporal resolution transcriptome analysis, our data have identified BIM and NOXA as BH3-only proteins that are effectors of TAF6δ-driven apoptosis.

## Methods

### Cell lines

In this study, HeLa WS (CCL-2, American Type Culture Collection (ATCC), Manassas, VA, USA), Hek293 (CRL-1573, ATCC), MDA-MB-231 (HTB-26, ATCC) and Hs-578T (HTB-126, ATCC), and Panc-1 (CRL-1469, ATCC) cell lines were used.

### Oligos

SSOs were 2′-O-methyl-oligoribonucleoside phosphorothioate antisense 20-mers (Sigma-Aldrich Canada, Oakville, ON, Canada) and were used to induce the splicing of TAF6 pre-messenger RNA towards the δ isoform as previously documented^3,4^. TAF6-specific SSO sequence was 5′-CUGUGCGAUCUCUUUGAUGC-3′ and control SSO 5′-AUGGCCUCGACGUGCGCGCU-3′. DsiRNAs as well as the PCR primers were obtained from Integrated DNA Technologies (Toronto, ON, Canada). As a negative control, we used a duplex without any target in mammalian cells: sense: 5′-CUUCCUCUCUUUCUCUCCCUUGUdGdA-3′; antisense: 5′-UCACAAGGGAGAGAAAGAGAGGAAGGA-3′. The knockdown of BIM (NM_138621) using Bim_Dsi1 sense: 5′-GACCGAGAAGGUAGACAAUUGCAGCCT-3′; antisense: 5′-AGGCUGCAAUUGUCUACCUUCUCGGUCUU-3′; and Bim_Dsi2 sense sequence: 5′-GACCGAGAAGGUAGACAAUUGCAGC-3′ and antisense: 5′-GCUGCAAUUGUCUACCUUCUCGGUCAC-3′. The knockdown of NOXA (NM_021127) was obtained with Noxa Dsi1 sense sequence: 5′-GCAUUGUAAUUGAGAGGAAUGUGAA-3′ and antisense: 5′-UUCACAUUCCUCUCAAUUACAAUGCAG-3′; Noxa Dsi2 sense sequence: 5′-GAGAUGACCUGUGAUUAGACUGGGC-3′ and antisense: 5′-GCCCAGUCUAAUCACAGGUCAUCUCUU-3′. The portion of TAF6 mRNA containing the splicing event involved in the determination between the α and δ isoforms was amplified by RT-PCR with the primers TAF6-1: 5′-AAAAAGGGATCCCATGGGCATCGCCCAGATTCAGG-3′ and TAF6-2: 5′-AAAAAGGAATTCCAAGGCGTAGTCAATGTCACTGG-3′. To amplify CrmAWT and CrmAT291R coding sequences we used the following primers: CrmA-BamHI-L: 5′-GATCCATGGCTTACCCATACGATGTTCC-3′; CrmA-BamHI-c: 5′-CATGGCTTACCCATACGATGTTCC-3′; CrmA-XhoI-L: 5′-TCGAGTTAATTAGTTGTTGGAGAGCAATATCTACC-3′; CrmA-XhoI-c: 5′-GTTAATTAGTTGTTGGAGAGCAATATCTACC-3′. Finally, hRPLPO (as a normalization control) BIM, NOXA, and ACRC were quantified by quantitative PCR with the following primers: hRPLPO-F: 5′-GCAATGTTGCCAGTGTCTG-3′; hRPPO-R: 5′- GCCTTGACCTTTTCAGCAA-3′; TBP-F: 5′-GGGGAGCTGTGATGTGAAGT-3′; TBP-R: 5′-GGAGAACAATTCTGGGTTGA-3′ BIM_F1: 5′-ATGTCTGACTCTGACTCTCG-3′; BIM_R2: 5′-CCTTGTGGCTCTGTCTGTAG-3′; NOXA_R1: 5′-TCCTGAGCAGAAGAGTTTGG-3′; NOXA_F1: 5′-GGAGATGCCTGGGAAGAAGG-3′; ACRC_F2: 5′-CTCATGGTGACGCATGGAAG-3′; and ACRC_R2: 5′-AGCAGCCAATCCTCGTTTTG-3′.

### Plasmids

The pcDNA3.1-Bcl-X_L_ plasmid was kindly provided by Dr. Alain Piché (Université de Sherbrooke, QC, Canada), plenti6V5A, plp1, plp2, and plpVSVg by Pr Nathalie Rivard (Université de Sherbrooke, QC, Canada), and pcDNA3.1-CrmAwt and pcDNA3.1-CrmAT291R by Pr Jean-Bernard Denault (Université de Sherbrooke, QC, Canada).

The plenti6V5A-Bcl-2 and plenti6V5A-Bcl-X_L_ plasmids, which were used to produce the Bcl-2 and Bcl-X_L_ stably overexpressing cell lines, were generated by classic restriction enzyme digestion approach. Bcl-2 and Bcl-X_L_ were excised from pcDNA3.1 backbone with *Eco*RI and *Apa*1, and ligated into plenti6v5A digested with the same enzymes.

To sub-clone the coding sequences of CrmAWT and CrmAT291R into the lentiviral plenti6V5A backbones, we used the PCR-based method previously described^45^ that allowed the addition of *Bam*HI and *Not*I restriction sites, respectively, upstream and downstream of this sequence. Then, the product was ligated into the plenti6V5A backbone digested with the same enzymes.

### Antibodies

Antibodies directed against cytochrome c (#556432 and #556433) were from BD Pharmingen (Mississauga, ON, Canada); BIM (B7929) and α-tubulin (B5-1-2) from Sigma-Aldrich; active caspase 3 (9661 and 9664) and Bcl-X_L_ (2762) from Cell Signaling (Beverly, MA, USA); GAPDH (ab9485) and VDAC-1 (ab15895) from Abcam (Toronto, ON, Canada); Noxa (OP180) from Calbiochem (Etobicoke, ON, Canada); Bax (06-499) from Upstate (Etobicoke, ON, Canada); and Bcl-2 (sc492) and HA (sc7392) from Santa Cruz (Dallas, TX, USA). The TBP antibody was kindly provided by Lazlo Tora from the IGBMC (Strasbourg, France).

### Cell culture

HeLa WS cell line was maintained in Dulbecco’s modified Eagle medium (DMEM; Wisent, St-Bruno, QC, Canada) containing 2.5% fetal calf serum (FCS; Wisent) and 2.5% calf serum CS (Wisent). Hek293, MDA-MB-231, and Hs-578T cells were grown in DMEM containing 10% FCS. Saos-2 cell line was maintained in Mc Coy’s medium (Wisent) enriched with 15% FCS. Panc-1 cells were cultured in DMEM with the addition of 10% FCS, 1% sodium pyruvate (Wisent), 1% Hepes (Wisent), and 1% l-glutamine (Wisent). These cell lines were kept at 37 °C with 5% CO_2_.

### Transfections

When 24-well plates were used, 250 ng plasmid were transfected with the addition of 0.4 µl of DMRIE-C transfecting agent (Invitrogen, Life Technologies, Burlington, ON, Canada) per well. The SSOs were transfected at a final concentration of 100 nM with 0.8 µl/well (HeLa WS, Hs-578T, Saos-2, and Panc-1) or 1 µl/well (MDA-MB-231) Lipofectamine 2000 (Invitrogen, Life Technologies). The DsiRNAs were transfected in HeLa WS cells at a final concentration of 10 nM with 0.8 µl/well Lipofectamine 2000. In 100 cm^2^ Petri dishes, 100 nM SSOs were transfected in HeLa WS cells with 20 µl Lipofectamine 2000. All the transfections were performed in the serum-free Opti-MEM medium (Gibco, Waltham, MA, USA) according to the manufacturer’s instructions. For the Bcl-2 and Bcl-X_L_ overexpression experiments, HeLa WS cells were plated at a concentration of 50 000 cells/well in 24-well plates. The plasmids were transfected 24 h later and the SSOs 16 h after the plasmids. In NOXA and BIM RNA-interference-mediated knockdown experiments, HeLa WS cells were plated in 24-well plates at a concentration of 50 000 cells/well. The DsiRNAs were transfected 24 h later, and the SSOs 24 h after the DsiRNAs. In the time-course experiments and in cell lines overexpressing Bcl-2, Bcl-XL, CrmAWT, or CrmA mtt (CrmAT291R), the cells were plated in 24-well plates at a concentration of 70 000 (HeLa, Hs-578T and Panc-1) or 90 000 (MDA-MB-231 and Saos-2) cells/well and transfected with the SSOs 24 h later. In all these experiments, the cells were harvested 18 h after the transfection with the SSOs.

### Treatment of the cells with drugs

Cells were treated with 100 ng/ml of TRAIL (Peprotech, Embrun, ON, Canada) for 12 h or 5 µM of cisplatin for 18 h (Pharmacy of the Oncology Service, Centre Hospitalier Universitaire de Sherbrooke, QC, Canada).

### RT-PCR

RT-PCR conditions and primers for amplification of both TAF6α and TAF6δ have been described^3^. PCR products were loaded on a 15% polyacrylamide gel to resolve the 150 and 180 bp bands corresponding to the α and δ isoforms, respectively. The products were also quantified by the Agilent capillary electrophoresis system (Agilent, Santa Clara, CA, USA). For this quantification, the samples were prepared with the Agilent DNA 1000 kit, loaded on microfluidic chips and the electrophoresis was performed according to the manufacturer’s instructions (Agilent). The ratio of TAF6δ isoform relative to TAF6 total mRNA (TAF6α + TAF6δ) was calculated for each sample and the histograms represent the mean as well as S.D. of three independent experiments.

### Cytofluorimetric detection of apoptosis

For the time-course measurement of apoptosis, HeLa Ws cells were submitted either to sub-G1 DNA content analysis with propidium iodide as previously described^2^ or detection of caspase-cleaved cytokeratin-18 by flow cytometry using Cytodeath reagent (Roche) according to the manufacturer’s recommendations.

For the other apoptosis quantifications, cells were fixed in 3% formaldehyde, permeabilized with 90% methanol, and incubated for 10 min with blocking buffer (1× phosphate-buffered saline (PBS) containing 0.5% bovine serum albumin (BSA)). Cells where then incubated overnight with anti-cleaved caspase 3 antibody (1/1500 in blocking buffer) at 4 °C, washed with blocking buffer, reincubated 1 h at room temperature with R-phycoerythrin donkey anti-rabbit IgG (1/100), washed again with blocking buffer, and resuspended in PBS 1× before analysis with the Beckton Dickinson FACScan flow cytometer (BD Biosciences, Mississauga, ON, Canada).

For each sample, after the exclusion of aggregates and debris, >10 000 cells were analyzed by flow cytometry. For each figure, three different experiments were performed, each containing three technical replicates. Apoptosis rates were calculated by subtracting the background levels in SSO control transfected cells (Figs. 3, 6, and 8) or untreated cells (Fig. 8) from that of TAF6δ-expressing (Figs. 3, 6, and 8) or TRAIL/cisplatin-treated (Fig. 8) cells. This difference has then been normalized relative to the empty vector (Figs. 3 and 8) or the scrambled siRNA (Fig. 6). The histograms show the mean of three independent experiments, each assayed by three technical replicates. Means and S.D.’s for the biological replicates were calculated using the three means of the three technical replicates of each biological replicate and the *p* values were calculated using the Student’s *t*-test.

### Microarray analysis of gene expression

HeLa WS were split into 24-well plates at a concentration of 70 000 cells/well and transfected 24 h later with 100 nM of scrambled or TAF6 SSO. The samples were collected every 2 h, from 2 to 18 h after the transfection. The samples preparation as well as the data acquisition was performed as described previously^3,4,46,47^. The microarray data are freely available at the database http://mace.ihes.fr under the accession 2853724252. The genes with a statistically significant (*p* < 0.05) difference of expression during the time-course in response TAF6δ expression were selected (Supplementary Table 1) and sorted according to their expression profiles with the kinetics function of the ace.map software^23,46–48^. The ontology analyses were also performed with the ace.map software based on the Panther classification system (http://pantherdb.org/) and previously established methods^49^. For the ontology studies we selected the genes, which were regulated in a statistically significant manner (*p* < 0.05) with a logQ under −1 or above 1 at one or several time points of the time-course. The list of these genes was submitted to the ontology enrichment function of Acemap. Figure 2a shows the 10 pathways over-represented with the best statistical significance.

For Fig. 2c, the gene expression of each time point was normalized to 2 h time point and the genes showing a statistically significant regulation (*p* < 0,05) were submitted to an ontological analysis (leo function of ace.map). Enrichment of four pro-apoptotic pathways in TAF6δ-regulated genes along the time compared to the control is represented. The dark fragments represent the ratio −log10(*p*value)/−log10(*p*value_Max_) at each time point. The correlation analysis was performed with the genes listed in Supplementary Table 1. Linear regressions were calculated based on the gene expression profiles as well as the apoptosis measurements with the Cytodeath and propidium iodide. For each gene, the Pearson method allowed us to determine a correlation coefficient (*R*^2^) with each of the cell death detection method. The genes showing an absolute correlation coefficient above 0.8 (Supplementary Table 3) were selected and submitted to an ontology study.

### Western blots

For the protein lysates preparation, the culture supernatants and the washing buffers were collected, pooled, and centrifuged to pellet the floating apoptotic cells. The cell layers were washed twice with PBS 1×, lysed in Laemmli buffer (100 mM Tris/HCl (pH 6.8), 10% glycerol, and 2.5% SDS) transferred onto the corresponding cell pellets and boiled 5 min at 100 °C. The protein quantifications were performed with the BCA reagent according to the manufacturer’s recommendations (Pierce BCA Protein Assay Kit, Thermo Scientific, Rockford, IL, USA). The proper amounts of protein were loaded and resolved on a polyacrylamide gel before the transfer on a polyvinylidene fluoride (BIM and NOXA) or nitrocellulose (for the other proteins) membrane in a tris/glycine buffer containing 20% ethanol. The membranes were blocked with PBS-T buffer (1× PBS with 0.05% Tween 20) containing 5% milk and incubated overnight with the primary antibodies. The membranes were then washed with PBS-T, incubated with the appropriate secondary antibody, and washed again. The signal was revealed with the ECL Western Lightning Plus reagent (Perkin-Elmer, Waltham, MA, USA) according to the manufacturer’s recommendations and detected with photographic films. Where indicated, the quantitation of western blots was performed by densitometry analysis performed with the ImageJ algorithm (https://imagej.nih.gov/ij/). The signals quantified for the detection of a specific protein (cytochrome *c*, BIM, or NOXA) were normalized to those of the loading control (GAPDH or TBP) and the S.D. of the mean ratios obtained for three independent experiments are shown on Figs. 4b and 6c.

### Cell fractionation

HeLa cells were seeded in 100 cm^2^ Petri dishes at a density of 3.10^6^ cells/dish and transfected with either the control or TAF6 SSO and trypsinized 18 h later. Cells were resuspended in mitochondria buffer (210 mM mannitol, 70 mM sucrose, 1 mM EDTA, 10 mM HEPES (pH 7.5) and protease inhibitors (Roche)), broken with a syringe, and centrifugated at 2000 r.p.m. for 5 min at 4 °C. The supernatant was then submitted to an additionnal centrifugation at 13 000 r.p.m. for 10 min at 4 °C. The pellet was used as the mitochondrial fraction and the supernatant as the cytoplasmic fraction for immunoblotting. This method was adapted from Eskes et al.^50^. To facilitate the loading on the polyacrylamide gels, the cytoplasmic fractions were concentrated with a methanol-chloroforme-based precipitation procedure.

### Immunofluorescence

HeLa WS cells were seeded in 24-well plate on polylysine-coated coverslips at a concentration of 60 000 cells/well. Cells were transfected with SSOs and fixed 14 h later in 3% paraformaldehyde, permeabilized with PBS-0.1% Triton X-100 (PBS-Tx) and incubated for 30 min in blocking buffer (PBS-Tx containing 1% BSA and 0.5% fish gelatine (Sigma-Aldrich)). Cells were then sequentially incubated 1 h at room temperature, followed by washes, with each of the following antibodies diluted in blocking buffer; anti-cytochrome c mAb (1/500) (BD Pharmingen), Alexa Fluor 546 goat anti-mouse IgG1 secondary antibody (1/1250) (Molecular Probes). Cells were then treated with Hoechst 33342 (2 mg/ml) and visualized by fluorescence microscopy. For statistical analysis, three independent experiments were performed. Seven fields were randomly chosen for scoring and a minimum of 400 cells were scored for cytochrome c distribution (mitochondrial vs. cytoplasmic). The number of cells with cytoplasmic cytochrome c was divided by the total number of cells to express the percentage of cells with cytochome c release. The Student’s *t*-test was used to assess the statistical significance through the calculation of *p* values.

### Establishment of stable cell lines overexpressing Bcl-2, Bcl-X_L_, CrmAWT, or CrmAT291R

Hek293 cells were plated in 100 cm^2^ Petri dishes and transfected with 2.5 μg plp1, 2.5 μg plp2, 2 μg plpVSVg, and 7 μg plenti6v5A-Bcl-2, Bcl-X_L,_ CrmAWT, or CrmAT291R with the calcium phosphate method [52]. After 48 h, the supernatant was harvested, filtrated through a 0.45 μm filter and applied on HeLa WS, Saos-2, Hs-578T, MDA-MB-231, and Panc-1 cells pre-treated for 1 h with 8 μg/ml polybrene (1,5-dimethyl-1,5-diazaundecamethylene polymethobromide, hexadimethrine bromide) (Sigma-Aldrich Canada). The infected cells were then selected with the addition of 5 μM Blasticidin S (Gibco) in the culture media.

### Quantitative PCR

RNA purification and reverse transcription into cDNA have been described previously^3^. The quantitative PCR were performed with 10ng of cDNA per reaction with KlenTaq Polymerase in a buffer containing 6 mM Tris-Hcl (pH 8.3), 25 mM KCl, 4 mM MgCl_2_, 75 mM Trealose, 0.1% Tween 20, 0.1 mg/ml non-acetylated BSA, and 0.1× Sybrgreen (Invitrogen, Life Technologies). The amplification of BIM, NOXA, and ACRC was performed with the BIM-F1/BIM-R2, NOXA-F1/NOXA-R1, and ACRC-F1/ACRC-R1 primers. The *hRPLPO* and *TBP* genes were used for the normalization. The raw data were analyzed using the ΔΔCt method and the average of TBP and hRPLPO Ct values for normalization^51^.

## Electronic supplementary material

Supplementary data legends

Supplementary Table 1

Supplementary Figure 1

Supplementary Table 2

Supplementary Table 3

Supplementary Figure 2

Supplementary Table 4

Supplementary Figure 3
