# Supplementary material for: BIM and NOXA are mitochondrial effectors of TAF6δ-driven apoptosis
Source: Cell Death Dis. 2018 Jan 22;9(2):70. doi: 10.1038/s41419-017-0115-3 (PMC5833734; doi:10.1038/s41419-017-0115-3)
Supplement: Supplementary file 1 — Supplementary data legends [file 41419_2017_115_MOESM1_ESM.docx]

**Supplementary data legends**

**Supplementary Table 1.** List of all genes statistically significantly regulated by TAF6δ at one or more time points during the time course. The expression profile of each gene on the microarray was determined with the ace.map algorithm and we selected for the genes which showed a statistically significant regulation (p<0.05) at one time point or more of the time-course.

**Supplementary Figure 1.** Comparison of the different transcription profiles to the latest time point (18H). The transcriptome profiles obtained at 2h (a), 4h (b), 6h (c), 8h (d), 10h (e), 12h (f), 14h (g) and 16h (h) were compared to the latest time point (18h). Venn diagrams were used to represent the number of overlapping genes between time points. The p value was assessed by the hypergeometric distribution method.

**Supplementary Table 2.** Ontology enrichment analysis for each time point during the time course. Genes showing a statistically significant (p<0.05) difference of expression compared to the control, with a LogQ value <-1 or >1 were selected for each time point of the time-course. These lists were then used to perform gene ontology analyses and determine the signaling pathways that were over-represented in a statistically significant manner (p<0.05) in TAF6δ-regulated genes.

**Supplementary Table 3.** List of the genes whose expression profiles correlate the most closely temporally with apoptosis. A correlation coefficient was determined between the expression profiles of each gene statistically significantly regulated by TAF6 and apoptosis measurements via the quantification of cytokeratin 18 cleavage. The genes showing a correlation coefficient higher than 0.8 have been selected to perform ontology studies.

**Supplementary Figure 2.** Blockade of the mitochondrial pathway by overexpression of the anti-apoptotic proteins Bcl-2 and Bcl-X_L_ attenuates TAF6δ-induced cell death in a range of cell lines. Wild-type and stable cell lines overexpressing Bcl-2 or Bcl-X_L_ were transfected with 100nM SSO and samples were collected 18h later to measure cell death. **(a)** Apoptosis quantification in control and Taf6 expressing cells. Black bars stand for scrambled SSO transfected cells and white ones for TAF6δ expressing cells. **(b)** Confirmation of Bcl-2 and Bcl-X_L_ overexpression by Western Blot. EV stands for empty vector. **(c)** Shifting of TAF6 splicing pattern upon SSO transfection. A fragment of the endogenous TAF6 was amplified from cDNA by PCR and PCR products were visualized on polyacrylamide gel. EV stands for empty vector. **(d)** Quantification of apoptosis specifically induced by the δ isoform. Apoptosis was measured in scrambled and TAF6 SSO transfected cells and the difference between TAF6δ expressing cells and control was normalized to the empty vector. Black bars represent empty vector infected cells, grey bars are for Bcl-2 overexpression and white bars for Bcl-X_L_ overexpression. Error bars show the standard deviation of 3 independent experiments. *p<0.05; **p<0.01.

**Supplementary table 4.** Over-representation of four sub-pathways of the intrinsic pathway for apoptosis in the 262 genes that correlate the most closely temporally with cell death measurements. The genes listed in the supplementary table 3 were submitted to an ontology study using Panther’s statistical over-representation test (http://pantherdb.org) based on the Reactome pathways database classification system (http://www.reactome.org). Obs : number of genes in the pathway present in the submitted gene set. Exp : number of genes expected to be observed in a random set of genes of equal number.

**Supplementary Figure 3.** Expression profiles of PUMA and p53AIP1. The differential expression of PUMA **(a)** and p53AIP1 **(b)** between TAF6δ expressing cells and controls along the time course was assessed from the microarray data. *= p<0.05; ** = p<0.01.
