# Supplementary material for: BIM and NOXA are mitochondrial effectors of TAF6δ-driven apoptosis
Source: Cell Death Dis. 2018 Jan 22;9(2):70. doi: 10.1038/s41419-017-0115-3 (PMC5833734; doi:10.1038/s41419-017-0115-3)
Supplement: Supplementary file 2 — Supplementary Table 1 [file 41419_2017_115_MOESM2_ESM.pdf]

Supplementary Table 1.

PROBE	GROUP	CLASS	Gene_Symbol	Gene_Name
100036	b	ooo+ooooo	ZNF532	zinc finger protein 532
100051	b	ooooooo+o	null	null
100057	a	o++++++++	EID3	null
100064	a	ooo++++++	EREG	epiregulin
100089	b	ooo+ooooo	C14orf114	chromosome 14 open reading frame 114
100093	b	ooo+ooooo	GLI3	GLI-Kruppel family member GLI3 (Greig cephalopolysyndactyly syndrome)
100117	a	oooooooo+	null	null
100172	b	ooo++oooo	HD	huntingtin (Huntington disease)
100278	a	ooo++++++	MCART1|MCART2	mitochondrial carrier triple repeat 1|mitochondrial carrier triple repeat 2
100309	b	oo+oooooo	C6orf147	chromosome 6 open reading frame 147
100403	b	ooo+ooooo	null	null
100436	b	oooo+oooo	ITGB6	integrin, beta 6
100485	h	oooooo-oo	ZCCHC6	zinc finger, CCHC domain containing 6
100513	b	ooo++oooo	EDG2	endothelial differentiation, lysophosphatidic acid G-protein-coupled receptor, 2
100596	a	oooooo+++	CEBPG	CCAAT/enhancer binding protein (C/EBP), gamma
100679	b	ooo+ooooo	SBNO1	sno, strawberry notch homolog 1 (Drosophila)
100689	c	ooo++o+++	BRWD1	bromodomain and WD repeat domain containing 1
100719	b	oooooo+oo	C18orf45	chromosome 18 open reading frame 45
100762	h	o-ooooooo	C6orf152	chromosome 6 open reading frame 152
100788	f	oo++----+	null	null
100801	c	oo++oooo+	PAX3	paired box gene 3 (Waardenburg syndrome 1)
100819	b	ooo+ooooo	MTMR9	myotubularin related protein 9
100830	h	o-ooooooo	null	null
100840	g	oooooooo-	BLVRA	biliverdin reductase A
100890	b	ooo+ooooo	OSBPL10	oxysterol binding protein-like 10
100952	b	ooooooo+o	RTBDN	null
101012	b	ooo+ooooo	RNF144	ring finger protein 144
101065	i	o-oo-----	TRA1P2	tumor rejection antigen (gp96) 1 pseudogene 2
101108	b	ooo+ooooo	PRKD3	protein kinase D3
101123	h	oooooo-oo	null	null
101140	a	ooo++++++	ChGn	null
101220	b	oooo+oooo	TRD@|TRA@	T cell receptor delta locus|T cell receptor alpha locus
101300	d	ooo+oooo-	NIN	ninein (GSK3B interacting protein)
101304	a	o++++++++	RAB12	RAB12, member RAS oncogene family
101333	a	ooo++++++	ZNF435	zinc finger protein 435
101347	b	ooo+ooooo	IQCG	IQ motif containing G
101374	b	oooo++++o	null	null
101391	b	ooooooo+o	CBLN1	cerebellin 1 precursor
101444	c	ooo+oo+++	NFKB2	nuclear factor of kappa light polypeptide gene enhancer in B-cells 2 (p49/p100)
101445	h	o-ooooooo	null	null
101505	b	oooooo+oo	null	null
101526	a	o++++++++	MAST2	microtubule associated serine/threonine kinase 2
101527	h	oooo----o	FLJ36492	null
101561	b	oooo+oooo	null	null
101569	b	oo+oooooo	KRTAP1-3	keratin associated protein 1-3
101570	a	oooooooo+	SCAMP1	secretory carrier membrane protein 1
101594	b	oooooo+oo	FLJ90652	null
101603	a	oooooooo+	TMEM57	transmembrane protein 57
101686	a	oooooo+++	C6orf166	chromosome 6 open reading frame 166
101688	b	o+ooooooo	null	null
101725	a	oo+++++++	LOC375449	null
101736	g	o--------	null	null
101742	g	o--------	GRIN2B	glutamate receptor, ionotropic, N-methyl D-aspartate 2B
101777	h	o-ooooooo	null	null
101783	b	ooooooo+o	null	null
101799	b	ooo+ooooo	DYNC2H1	dynein, cytoplasmic 2, heavy chain 1
101800	b	oo+oooooo	BARX2	BarH-like homeobox 2
101821	b	ooooo+ooo	MGC40579	null
101835	g	o--------	CHES1	checkpoint suppressor 1
101843	h	oooo----o	null	null
101845	a	o++++++++	THAP10	THAP domain containing 10
101887	a	o++++++++	N4BP1	null
101949	h	o-ooooooo	null	null
101974	a	ooo++++++	RNPC2	RNA-binding region (RNP1, RRM) containing 2
102001	a	oo+++++++	LOC400986	null
102010	g	o--------	NOL4	nucleolar protein 4
102049	b	ooo+ooooo	TDRD9	tudor domain containing 9
102051	b	oooo+oooo	null	null

102059	b	ooo+ooooo	C14orf102	chromosome 14 open reading frame 102
102063	b	ooooo+++o	C6orf130	chromosome 6 open reading frame 130
102081	a	ooo++++++	NR4A3	nuclear receptor subfamily 4, group A, member 3
102133	b	ooo+ooooo	C3orf9	chromosome 3 open reading frame 9
102148	b	ooo+ooooo	null	null
102161	b	oooooo+oo	RHOF	ras homolog gene family, member F (in filopodia)
102162	b	oooooo+oo	FLJ10661	null
102168	b	oo++ooooo	C10orf41	chromosome 10 open reading frame 41
102211	b	ooo+ooooo	PMS1	PMS1 postmeiotic segregation increased 1 (S. cerevisiae)
102249	h	oooooo-oo	null	null
102263	h	oooo----o	IL17RB	interleukin 17 receptor B
102302	g	oooooooo-	FLJ43855	null
102344	b	ooo+ooooo	PANK4	pantothenate kinase 4
102393	b	ooo+ooooo	null	null
102403	b	ooo+ooooo	PHF16	PHD finger protein 16
102419	b	ooo+ooooo	C19orf7	chromosome 19 open reading frame 7
102488	b	o+ooooooo	null	null
102539	b	ooooooo+o	WTIP	Wilms tumor 1 interacting protein
102558	b	ooooo+ooo	ANPEP	alanyl (membrane) aminopeptidase (aminopeptidase N, aminopeptidase M, microsomal aminopeptidase, CD13, p150)
102581	a	oo+++++++	MTRF1	mitochondrial translational release factor 1
102592	b	ooo+ooooo	SAPS3	SAPS domain family, member 3
102664	b	ooo+ooooo	NOL10	nucleolar protein 10
102688	h	oooo----o	IQSEC1	IQ motif and Sec7 domain 1
102734	a	oooooooo+	SBSN	null
102754	b	ooo+ooooo	null	null
102762	g	o--------	LOC441971	null
102795	h	oooooo-oo	null	null
102826	h	oooooo-oo	null	null
102848	h	ooo-ooooo	MFHAS1	malignant fibrous histiocytoma amplified sequence 1
102896	b	oooooo+oo	NRIP3	nuclear receptor interacting protein 3
102916	b	ooo+ooooo	ZNF662	zinc finger protein 662
102937	a	ooo++++++	PNRC1	proline-rich nuclear receptor coactivator 1
102959	b	ooo+ooooo	KHDRBS3	KH domain containing, RNA binding, signal transduction associated 3
102971	b	ooo+ooooo	SLC29A2	solute carrier family 29 (nucleoside transporters), member 2
103021	b	ooo+ooooo	MYCBP2	MYC binding protein 2
103029	b	ooo+ooooo	null	null
103069	g	oooo-----	CTNND1	catenin (cadherin-associated protein), delta 1
103079	b	ooo++oooo	LOC440677	null
103082	b	ooooooo+o	LOC286080	null
103111	b	oooooo+oo	C22orf23	chromosome 22 open reading frame 23
103264	b	ooooo+ooo	null	null
103288	g	o--------	OR52H1	olfactory receptor, family 52, subfamily H, member 1
103301	b	ooo+ooooo	RHBDF1	rhomboid family 1 (Drosophila)
103310	b	ooo+ooooo	DNER	null
103366	a	oo+++++++	MGC16037	null
103394	a	ooooooo++	LOC440282	null
103395	b	ooo+ooooo	CTAGE1	cutaneous T-cell lymphoma-associated antigen 1
103418	h	o-ooooooo	PMS2|LOC402554	PMS2 postmeiotic segregation increased 2 (S. cerevisiae)
103441	b	ooo+ooooo	C6orf64	chromosome 6 open reading frame 64
103451	a	ooooo++++	CHMP1B	chromatin modifying protein 1B
103461	a	ooo++++++	HIST1H2BG|HIST1H2BF	histone 1, H2bg|histone 1, H2bf
103468	a	oooooo+++	HNRPUL2	heterogeneous nuclear ribonucleoprotein U-like 2
103516	g	ooooo----	FAM65A	family with sequence similarity 65, member A
103522	b	ooo+++ooo	KIAA0040	KIAA0040
103604	b	ooo+ooooo	PRKD2	protein kinase D2
103613	b	oooooo+oo	MUC20	mucin 20
103618	a	oooooo+++	DNAJB4	DnaJ (Hsp40) homolog, subfamily B, member 4
103627	b	oooooo+oo	MAGEB6	melanoma antigen family B, 6
103651	c	ooo+oo+++	GOPC|ROS1	golgi associated PDZ and coiled-coil motif containing|v-ros UR2 sarcoma virus oncogene homolog 1 (avian)
103654	h	oooo----o	FLJ13710	null
103720	b	ooo+ooooo	TRIP12	thyroid hormone receptor interactor 12
103752	c	ooo+oo+++	AMOTL2	angiomotin like 2
103764	b	oooooo+oo	C11orf30	chromosome 11 open reading frame 30
103786	b	ooo+ooooo	ISLR	immunoglobulin superfamily containing leucine-rich repeat
103792	b	oooo+oooo	OR13H1	olfactory receptor, family 13, subfamily H, member 1
103793	b	oooooo++o	FAHD1	fumarylacetoacetate hydrolase domain containing 1
103805	b	ooo+ooooo	null	null
103824	b	ooooooo+o	null	null

103870	b	ooo+ooooo	SLC12A6	solute carrier family 12 (potassium/chloride transporters), member 6
103876	b	ooo+ooooo	null	null
103885	a	oooooooo+	NEK10	NIMA (never in mitosis gene a)- related kinase 10
103888	j	o-oooooo+	CYP3A4	cytochrome P450, family 3, subfamily A, polypeptide 4
103970	d	ooo+oooo-	FAM73B	family with sequence similarity 73, member B
104009	a	ooo++++++	GABPB2	GA binding protein transcription factor, beta subunit 2
104036	b	ooo+ooooo	TMEM16F	transmembrane protein 16F
104081	b	oooooo+oo	IHPK3	inositol hexaphosphate kinase 3
104099	b	ooo+ooooo	FLJ14640	null
104102	a	o++++++++	LOC284865	null
104119	a	oooooo+++	FJX1	four jointed box 1 (Drosophila)
104158	g	oooooo---	DSC3	desmocollin 3
104200	b	ooo++oooo	TRAPPC2	trafficking protein particle complex 2
104213	a	ooo++++++	ZNF274	zinc finger protein 274
104275	b	oooo+oooo	SMCHD1	structural maintenance of chromosomes flexible hinge domain containing 1
104328	b	ooo+ooooo	SMARCA1	SWI/SNF related, matrix associated, actin dependent regulator of chromatin, subfamily a, member 1
104347	b	oooo+oooo	ZCWPW2	zinc finger, CW type with PWWP domain 2
104384	h	oooooo-oo	F13A1	coagulation factor XIII, A1 polypeptide
104387	a	ooo++++++	PAK2	p21 (CDKN1A)-activated kinase 2
104402	a	ooo++++++	KLF10	Kruppel-like factor 10
10441133	b	oooo+oooo	LOC284688	null
104428	a	ooo++++++	ZMYM5	zinc finger, MYM-type 5
104458	f	oo++----+	ELAC1	elaC homolog 1 (E. coli)
104539	a	ooo++++++	CLEC1A	C-type lectin domain family 1, member A
104549	b	oooooo+oo	SYDE1	synapse defective 1, Rho GTPase, homolog 1 (C. elegans)
104572	b	oooo+oooo	KIF27	kinesin family member 27
104610	b	ooo+++++o	PYGM	phosphorylase, glycogen; muscle (McArdle syndrome, glycogen storage disease type V)
104648	b	oooo+oooo	SLC10A1	solute carrier family 10 (sodium/bile acid cotransporter family), member 1
104709	g	o--------	GJB1	gap junction protein, beta 1, 32kDa (connexin 32, Charcot-Marie-Tooth neuropathy, X-linked)
104711	f	oo++----+	CCDC45	coiled-coil domain containing 45
104763	b	oooo+oooo	null	null
104770	b	ooooo+ooo	BEX2	brain expressed X-linked 2
104851	i	o--o-----	TTMA	null
104870	h	o-ooooooo	LOC283970|KIAA0251	null
104873	a	ooooooo++	PNRC2	proline-rich nuclear receptor coactivator 2
104878	b	ooo+ooooo	PGK2	phosphoglycerate kinase 2
104923	a	oooooo+++	IGFBP3	insulin-like growth factor binding protein 3
104955	g	oooooo---	SPAG7	sperm associated antigen 7
105014	b	ooo+++ooo	CASC5	cancer susceptibility candidate 5
105040	b	ooo+ooooo	VPS16	vacuolar protein sorting 16 (yeast)
105061	b	ooo+ooooo	COBLL1	COBL-like 1
105064	b	ooo+ooooo	PEX12	peroxisomal biogenesis factor 12
105075	g	o--------	KIAA1211	null
105105	b	ooo+ooooo	S100PBP	S100P binding protein
105110	b	ooo+ooooo	null	null
105120	a	ooo++++++	CLK3	CDC-like kinase 3
105130	g	ooooooo--	CYP2S1	cytochrome P450, family 2, subfamily S, polypeptide 1
105150	g	o--------	SSX4B|SSX7|SSX4|SSX2	synovial sarcoma, X breakpoint 4B|synovial sarcoma, X breakpoint 7|synovial sarcoma, X breakpoint 4|synovial sarcoma, X breakpoint 2
105271	g	oooooooo-	NME4	non-metastatic cells 4, protein expressed in
105275	h	oooo----o	FLJ44606	null
105321	b	ooo+ooooo	GPR133	G protein-coupled receptor 133
105351	a	o++++++++	EIF2AK3	eukaryotic translation initiation factor 2-alpha kinase 3
105376	b	ooo+ooooo	CARKL	carbohydrate kinase-like
105377	a	oooooo+++	CTH	cystathionase (cystathionine gamma-lyase)
105390	c	ooo++o+++	FOSB	FBJ murine osteosarcoma viral oncogene homolog B
105419	b	ooo+ooooo	ZNF618	zinc finger protein 618
10545131	b	ooooo+ooo	null	null
105457	b	ooo+ooooo	CLPB	ClpB caseinolytic peptidase B homolog (E. coli)
10546171	b	ooooo+ooo	null	null
105475	b	ooooooo+o	null	null
10549129	h	o-ooooooo	null	null
105499	h	oooooo--o	LRRN5	leucine rich repeat neuronal 5
105503	b	oooo+oooo	LOC129607	null
105520	b	ooo+ooooo	SEL1L	sel-1 suppressor of lin-12-like (C. elegans)
105539	g	oooooooo-	KRT8	keratin 8
105544	b	ooo+ooooo	AFF4	AF4/FMR2 family, member 4

105562	b	ooo+ooooo	FLJ20272	null
105575	d	ooo+oooo-	RAI17	retinoic acid induced 17
10558829	a	ooooooo++	null	null
10558922	b	oo+oooooo	LOC157627	null
105603	a	o++++++++	SPOCK2	sparc/osteonectin, cwcv and kazal-like domains proteoglycan (testican) 2
105627	b	ooo+ooooo	ERBB2	v-erb-b2 erythroblastic leukemia viral oncogene homolog 2, neuro/glioblastoma derived oncogene homolog (avian)
105644	b	ooo+ooooo	null	null
105724	h	oooooo-oo	RHCG	Rhesus blood group, C glycoprotein
105744	b	ooo+ooooo	MAGI2	membrane associated guanylate kinase, WW and PDZ domain containing 2
10576549	b	ooo+ooooo	null	null
105802	b	ooo+ooooo	TNPO2	transportin 2 (importin 3, karyopherin beta 2b)
105805	h	oooo----o	RNASE11	ribonuclease, RNase A family, 11 (non-active)
105811	h	oo-oooooo	SMCR7	Smith-Magenis syndrome chromosome region, candidate 7
105813	h	oooo----o	MGC5457	null
105852	b	ooo+ooooo	null	null
105900	a	oo+++++++	LOC51315	null
105914	h	o-ooooooo	ZNF326	zinc finger protein 326
105919	b	ooooooo+o	SYCN	syncollin
105931	a	ooo++++++	YEATS2	YEATS domain containing 2
105957	b	oooooo+oo	null	null
105979	a	ooo++++++	MGC13138	null
105982	a	ooo++++++	ZSWIM3	zinc finger, SWIM-type containing 3
105986	h	oooo----o	LOC388477	null
105989	b	oooo+oooo	ZNF80	zinc finger protein 80 (pT17)
105991	h	o-------o	AJAP1	adherens junction associated protein 1
106015	a	oooooo+++	C17orf59	chromosome 17 open reading frame 59
106026	a	oooooooo+	null	null
106038	b	ooooooo+o	REG4	regenerating islet-derived family, member 4
106094	b	ooo+ooooo	BAZ2B	bromodomain adjacent to zinc finger domain, 2B
106125	b	ooo+ooooo	EXT2	exostoses (multiple) 2
106153	a	oo+++++++	null	null
106172	b	ooo+ooooo	C11orf2	chromosome 11 open reading frame2
106226	a	ooo++++++	CASP8AP2	CASP8 associated protein 2
106261	c	oo++oooo+	SPANXA1|SPANXE|SPANXA2|SPANXC	sperm protein associated with the nucleus, X-linked, family member A1|SPANX family, member E|SPANX family, member A2|SPANX family, member C
106266	a	ooo++++++	RNF182	ring finger protein 182
106314	b	ooo++oooo	WDR52	WD repeat domain 52
10631704	c	ooo+o++++	null	null
106328	a	oooooooo+	OR10T2	olfactory receptor, family 10, subfamily T, member 2
106341	h	oo-oooooo	TXNDC2	thioredoxin domain containing 2 (spermatozoa)
106342	d	ooo+oo---	GPATC2	G patch domain containing 2
106353	b	oooo+oooo	LOC91801	null
106355	b	o+ooooooo	SSFA2	sperm specific antigen 2
106362	b	oooo+oooo	SMAD9	SMAD, mothers against DPP homolog 9 (Drosophila)
106376	a	ooooo++++	NOTCH2NL	Notch homolog 2 (Drosophila) N-terminal like
106382	b	ooo+ooooo	ZFP64	zinc finger protein 64 homolog (mouse)
106407	b	oooo++ooo	ALS2CR19	amyotrophic lateral sclerosis 2 (juvenile) chromosome region, candidate 19
106426	b	ooo+ooooo	ARHGEF3	Rho guanine nucleotide exchange factor (GEF) 3
106526	b	oooooo+oo	F11	coagulation factor XI (plasma thromboplastin antecedent)
106536	h	o-ooooooo	THOC2	THO complex 2
106563	c	ooo+o++++	GRAMD1B	GRAM domain containing 1B
106571	b	ooooo+ooo	LOC388227	null
106573	i	oooo-ooo-	AOX1	aldehyde oxidase 1
106596	a	o++++++++	TACC2	transforming, acidic coiled-coil containing protein 2
106600	a	oooo+++++	GADD45B	growth arrest and DNA-damage-inducible, beta
10662524	b	ooooooo+o	null	null
106626	a	ooooooo++	EPC1	enhancer of polycomb homolog 1 (Drosophila)
106712	a	o++++++++	PREB	prolactin regulatory element binding
106732	h	oooo----o	ABCA1	ATP-binding cassette, sub-family A (ABC1), member 1
106760	b	ooo++oooo	null	null
106788	a	ooo++++++	null	null
106839	a	ooo++++++	null	null
106846	b	ooooooo+o	SHH	sonic hedgehog homolog (Drosophila)
106892	h	o-----ooo	null	null
106893	b	ooo+ooooo	LPL	lipoprotein lipase
10692164	b	ooo+ooooo	LOC440374|RAB43	RAB43, member RAS oncogene family
106932	b	ooooooo+o	PCDHB4	protocadherin beta 4
106960	b	ooo+ooooo	DYX1C1	dyslexia susceptibility 1 candidate 1
10696147	a	oo+++++++	null	null
10696290	b	oo++ooooo	null	null

10697489	b	ooo+ooooo	null	null
10697643	h	o-ooooooo	null	null
106979	a	ooo++++++	MAP1LC3B	microtubule-associated protein 1 light chain 3 beta
10698574	b	ooooo+ooo	null	null
10698651	g	o--------	null	null
106989	b	o+ooooooo	NBEAL1	neurobeachin-like 1
106991	b	ooo+ooooo	KIAA1280	null
10699996	a	o++++++++	null	null
10702467	g	oo-------	null	null
107061	b	ooooo+ooo	PLG	plasminogen
107094	a	ooo++++++	SLC22A4	solute carrier family 22 (organic cation transporter), member 4
107121	b	ooooooo+o	C14orf113	chromosome 14 open reading frame 113
10712347	b	ooo+ooooo	null	null
10713472	f	oo++----+	LOC144817	null
107157	a	oooooo+++	RABGGTB	Rab geranylgeranyltransferase, beta subunit
107191	b	ooo+ooooo	ARHGEF5|LOC441296	Rho guanine nucleotide exchange factor (GEF) 5
107193	a	o++++++++	DDX6	DEAD (Asp-Glu-Ala-Asp) box polypeptide 6
107205	g	o--------	PTRF	polymerase I and transcript release factor
107207	g	o--------	null	null
107212	h	oooooo-oo	SNX9	sorting nexin 9
107243	a	ooo++++++	null	null
107292	a	ooo++++++	SESN2	sestrin 2
107308	b	ooo+ooooo	null	null
107359	h	o-ooooooo	C10orf110	chromosome 10 open reading frame 110
107361	c	oo++oooo+	AMBP	alpha-1-microglobulin/bikunin precursor
107384	b	oooooo+oo	LYG2	null
107428	g	oooooooo-	XPNPEP1	X-prolyl aminopeptidase (aminopeptidase P) 1, soluble
107433	a	ooo++++++	ZNF544	zinc finger protein 544
107436	a	oo+++++++	LOC57149	null
107455	c	oo++oooo+	null	null
107457	a	oooooooo+	LCN2	lipocalin 2 (oncogene 24p3)
107502	h	o--oooooo	LOC390557	null
107531	g	ooooooo--	CELSR2	cadherin, EGF LAG seven-pass G-type receptor 2 (flamingo homolog, Drosophila)
107542	b	ooo+ooooo	ZNF84	zinc finger protein 84 (HPF2)
107593	b	ooo+ooooo	ABCA3	ATP-binding cassette, sub-family A (ABC1), member 3
107614	b	ooo+ooooo	PERLD1	per1-like domain containing 1
107615	b	ooo+ooooo	LOC149086	null
107679	b	ooo+ooooo	C6orf192	chromosome 6 open reading frame 192
107690	c	oooo+++o+	CCNT1	cyclin T1
107713	b	ooo+++ooo	null	null
107720	b	ooo++oooo	LRAT	lecithin retinol acyltransferase (phosphatidylcholine--retinol O-acyltransferase)
107722	b	ooo+ooooo	CUL5	cullin 5
107778	c	oooo+oo++	LOC390908	null
107789	h	o-ooooooo	LOC339970|GRINL1A|Gcom1	glutamate receptor, ionotropic, N-methyl D-aspartate-like 1A
107802	g	oooooo---	C9orf116	chromosome 9 open reading frame 116
107813	b	ooo+ooooo	AASDH	null
107940	b	oooo+oooo	KLHDC6	kelch domain containing 6
107949	g	o--------	STK31	serine/threonine kinase 31
107968	a	oooooooo+	SGCZ	sarcoglycan zeta
108040	b	oooo+oooo	null	null
108041	b	ooo+ooooo	EHBP1	EH domain binding protein 1
108050	a	oooo+++++	LOC158960	null
108052	g	oooo-----	DIAPH3	diaphanous homolog 3 (Drosophila)
108059	i	o-oo-----	SORL1	sortilin-related receptor, L(DLR class) A repeats-containing
108064	b	ooooooo+o	MYCPBP	c-myc promoter binding protein
108133	b	ooo++oooo	C20orf194	chromosome 20 open reading frame 194
108201	c	ooo+oo+++	PHC3	polyhomeotic like 3 (Drosophila)
108203	a	oooo+++++	FOXD1	forkhead box D1
108216	a	ooooo++++	null	null
108218	h	oooo-oooo	AASDH	null
108231	a	oo+++++++	AHSA2	AHA1, activator of heat shock 90kDa protein ATPase homolog 2 (yeast)
108245	b	ooo+ooooo	C9orf102	chromosome 9 open reading frame 102
108246	b	ooo+ooooo	FBXW8	F-box and WD-40 domain protein 8
108275	b	ooooooo+o	NPY6R	neuropeptide Y receptor Y6 (pseudogene)
108278	b	ooo+ooooo	null	null
108301	b	ooo+ooooo	PDGFD	platelet derived growth factor D
108343	b	oooo++ooo	null	null
108363	b	oooo+oooo	null	null
108382	g	o--------	ZNRF3	zinc and ring finger 3

108443	a	oo+++++++	ARMETL1	arginine-rich, mutated in early stage tumors-like 1
108457	a	oo+++++++	TSP-NY	null
108472	b	ooo+ooooo	ZNF291	zinc finger protein 291
108491	b	ooo+ooooo	NID2	nidogen 2 (osteonidogen)
108533	f	oo++----+	FLJ38122	null
108549	b	o+ooooooo	TTC7B	tetratricopeptide repeat domain 7B
108577	b	ooo+ooooo	SRPX	sushi-repeat-containing protein, X-linked
108623	a	oo+++++++	C8orf4	chromosome 8 open reading frame 4
108625	b	oooo++ooo	null	null
108628	g	oooooooo-	MRPL54	mitochondrial ribosomal protein L54
108672	i	o-oo-----	HBD	hemoglobin, delta
108704	b	oo++++++o	RNF166	ring finger protein 166
108706	b	oooo+oooo	EIF1AY	eukaryotic translation initiation factor 1A, Y-linked
108718	c	oo++oo+++	STK38L	serine/threonine kinase 38 like
108742	b	ooo+ooooo	C8orf44|SGK3	chromosome 8 open reading frame 44|serum/glucocorticoid regulated kinase family, member 3
108769	a	ooo++++++	FEM1B	fem-1 homolog b (C. elegans)
108775	b	ooo+ooooo	LHPP	null
108776	b	ooooo+ooo	null	null
108787	b	ooo+ooooo	C1R	complement component 1, r subcomponent
108789	a	oooooooo+	DSG4	desmoglein 4
108808	h	oo-oooooo	KCNV1	potassium channel, subfamily V, member 1
108880	b	ooo+ooooo	GPR124	G protein-coupled receptor 124
108883	a	oo+++++++	SOCS4	suppressor of cytokine signaling 4
108887	h	o-ooooooo	SET8	null
108930	g	o--------	ZNF169	zinc finger protein 169
108946	d	ooo+ooo--	CD99L2	CD99 antigen-like 2
108955	b	oooo+oooo	OCLN	occludin
109097	b	ooo+++ooo	null	null
109100	b	oooo+oooo	null	null
109169	a	ooooo++++	null	null
109316	a	oooooooo+	null	null
109337	h	ooooooo-o	LOC391742	null
109389	b	ooooo+ooo	SYT6	synaptotagmin VI
109390	a	ooo++++++	SETDB1	SET domain, bifurcated 1
109394	g	oo-------	CCDC49	coiled-coil domain containing 49
109399	b	ooo+ooooo	FLJ12334	null
109524	g	ooooooo--	PPP2R5D	protein phosphatase 2, regulatory subunit B (B56), delta isoform
109532	b	ooo+ooooo	HMFN0839	null
109533	g	ooooo----	ALDH1A3	aldehyde dehydrogenase 1 family, member A3
109537	b	oooo++ooo	FLJ13273	null
109540	b	ooo+ooooo	KIAA0701	null
109609	b	oooooo++o	THTPA	thiamine triphosphatase
109628	g	oooooooo-	ATP5D	ATP synthase, H+ transporting, mitochondrial F1 complex, delta subunit
109651	g	o--------	INSM2	insulinoma-associated 2
109656	b	ooooo+ooo	null	null
109684	b	ooo+ooooo	SSX2IP	synovial sarcoma, X breakpoint 2 interacting protein
109708	a	oo+++++++	C1orf79	chromosome 1 open reading frame 79
109729	g	oooo-----	RNF141	ring finger protein 141
109737	b	ooo+ooooo	GNA12	guanine nucleotide binding protein (G protein) alpha 12
109767	a	oo+++++++	ABCC3	ATP-binding cassette, sub-family C (CFTR/MRP), member 3
109780	a	ooo++++++	ECM1	extracellular matrix protein 1
109833	g	o--------	SLBP	stem-loop (histone) binding protein
109839	b	ooo+ooooo	ZCCHC11	zinc finger, CCHC domain containing 11
109870	g	o--------	LOC641518	null
109989	b	ooo+ooooo	MAGI3	membrane associated guanylate kinase, WW and PDZ domain containing 3
110014	b	ooo+ooooo	VLDLR	very low density lipoprotein receptor
110033	b	ooo+ooooo	CES1	carboxylesterase 1 (monocyte/macrophage serine esterase 1)
110046	b	ooo+ooooo	UTP14C	UTP14, U3 small nucleolar ribonucleoprotein, homolog C (yeast)
110071	g	o--------	CCR2	chemokine (C-C motif) receptor 2
110133	b	ooo+ooooo	MOCOS	molybdenum cofactor sulfurase
110143	h	o-ooooooo	MYOZ3	myozenin 3
110172	b	oooo+oooo	SLC6A19	solute carrier family 6 (neutral amino acid transporter), member 19
110188	b	ooo++++oo	C1orf183	chromosome 1 open reading frame 183
110198	g	oo-------	NAT6|HYAL3	N-acetyltransferase 6|hyaluronoglucosaminidase 3
110207	b	o+ooooooo	null	null
110210	a	oo+++++++	SPIB	Spi-B transcription factor (Spi-1/PU.1 related)
110226	c	ooo+o++++	LOC374443	null
110268	b	ooo+ooooo	null	null
110287	b	oooooo+oo	null	null
110310	b	oo++ooooo	TRIM56	tripartite motif-containing 56

110329	g	oooo-----	SYNC1	syncoilin, intermediate filament 1
110362	f	oo++----+	C10orf82	chromosome 10 open reading frame 82
110417	c	oo++oo+++	PDPK1	3-phosphoinositide dependent protein kinase-1
110418	h	o--oooooo	AVIL	advillin
110488	h	o-ooooooo	UBXD8	UBX domain containing 8
110564	a	oo+++++++	null	null
110566	h	ooooo---o	null	null
110569	b	oooo+oooo	DCD	dermcidin
110587	b	ooo+ooooo	EPB41L3	erythrocyte membrane protein band 4.1-like 3
110602	b	ooo+ooooo	GALNAC4S-6ST	null
110609	b	oo++++++o	CYBB	cytochrome b-245, beta polypeptide (chronic granulomatous disease)
110661	g	o--------	PAX8	paired box gene 8
110662	f	oo++----+	LYPD2	LY6/PLAUR domain containing 2
110684	h	o-ooooooo	null	null
110695	b	oo++ooooo	ADAM12	ADAM metallopeptidase domain 12 (meltrin alpha)
110704	b	ooo++oooo	null	null
110721	b	ooo+ooooo	PLXDC1	plexin domain containing 1
110722	b	ooo+++++o	VSNL1	visinin-like 1
110772	b	ooooo+ooo	COL19A1	collagen, type XIX, alpha 1
110790	b	ooo+ooooo	ARHGAP17	Rho GTPase activating protein 17
110801	b	ooo+ooooo	STOX1	storkhead box 1
110915	c	oooooo+o+	IL17C	interleukin 17C
110933	b	ooo++oooo	LOXL2	lysyl oxidase-like 2
110953	b	oooooo++o	LRSAM1	leucine rich repeat and sterile alpha motif containing 1
110971	a	oooooooo+	SLAMF8	SLAM family member 8
110988	b	ooo+ooooo	IFT52	intraflagellar transport 52 homolog (Chlamydomonas)
111003	b	ooo+ooooo	NBLA04196	null
111012	a	ooooo++++	MYT1L	myelin transcription factor 1-like
111032	i	o---o----	P2RY13	purinergic receptor P2Y, G-protein coupled, 13
111111	b	ooo+ooooo	ITPR1	inositol 1,4,5-triphosphate receptor, type 1
111132	g	oooooo---	TXNRD3	thioredoxin reductase 3
111177	h	oooo----o	RORB	RAR-related orphan receptor B
111234	b	ooo++++oo	null	null
111253	h	o-----ooo	MLLT7	myeloid/lymphoid or mixed-lineage leukemia (trithorax homolog, Drosophila); translocated to, 7
111269	b	oooo+oooo	SLC45A3	solute carrier family 45, member 3
111356	a	ooooo++++	NFKBIA	nuclear factor of kappa light polypeptide gene enhancer in B-cells inhibitor, alpha
111362	b	ooooooo+o	MGC10986	null
111384	b	oooo+oooo	OR2A14	olfactory receptor, family 2, subfamily A, member 14
111389	b	ooooooo+o	NOX1	NADPH oxidase 1
111441	b	ooooooo+o	PCDHGA8	protocadherin gamma subfamily A, 8
111499	b	o+++ooooo	EPS8L2	EPS8-like 2
111527	b	ooo+ooooo	KIAA0676	null
111550	a	oo+++++++	HIST1H4K	histone 1, H4k
111742	h	o-ooooooo	LOC132321	null
111796	a	ooo++++++	URB	null
111806	b	ooooooo+o	DKFZp434O0527	null
111812	b	ooo+ooooo	WHSC1L1	Wolf-Hirschhorn syndrome candidate 1-like 1
111840	h	oooo----o	TRBV3-1	T cell receptor beta variable 3-1
111844	a	ooooo++++	HIST1H2AK	histone 1, H2ak
111849	b	ooo+ooooo	FLJ20641	null
111900	b	ooooo++oo	LOC162427	null
111927	b	ooo+ooooo	STS	steroid sulfatase (microsomal), arylsulfatase C, isozyme S
111979	b	ooo+ooooo	KIAA1429	KIAA1429
111987	a	oo+++++++	DNAJB9	DnaJ (Hsp40) homolog, subfamily B, member 9
112052	b	ooo++oooo	AP1M2	adaptor-related protein complex 1, mu 2 subunit
112069	g	o--------	KNG1	kininogen 1
112076	b	ooo+ooooo	LRRIQ2	leucine-rich repeats and IQ motif containing 2
112081	a	o++++++++	POLQ	polymerase (DNA directed), theta
112106	b	oooo+oooo	CCRL2	chemokine (C-C motif) receptor-like 2
112110	b	ooo+ooooo	HYPB	null
112111	g	o--------	MLANA	melan-A
112112	a	o++++++++	NUP43	nucleoporin 43kDa
112159	b	ooooo+ooo	LOC339782	null
112169	b	ooo+ooooo	TMCC1	transmembrane and coiled-coil domain family 1
112188	a	oooooo+++	C18orf19	chromosome 18 open reading frame 19
112248	b	ooooo+ooo	CLEC10A	C-type lectin domain family 10, member A
112249	b	ooo+ooooo	MTR	5-methyltetrahydrofolate-homocysteine methyltransferase
112281	b	ooooo+ooo	C20orf98	chromosome 20 open reading frame 98
112284	b	ooooo+ooo	LRRC25	leucine rich repeat containing 25
112312	b	ooo+ooooo	MYCBP	c-myc binding protein

112323	b	oooo++++o	CNTNAP2	contactin associated protein-like 2
112354	g	o--------	GRM2	glutamate receptor, metabotropic 2
112377	a	oo+++++++	FLJ10287	null
112393	b	ooooooo+o	null	null
112440	b	oooo+oooo	WIPI1	WD repeat domain, phosphoinositide interacting 1
112458	b	ooo+ooooo	JAK2	Janus kinase 2 (a protein tyrosine kinase)
112463	b	oooo+oooo	BRDG1	null
112467	b	ooo+ooooo	SPPL2A	null
112476	b	ooo++++oo	RRAGD	Ras-related GTP binding D
112488	a	ooo++++++	NRIP1	nuclear receptor interacting protein 1
112496	b	oo+oooooo	null	null
112499	f	oo++----+	FLJ13946	null
112528	a	ooo++++++	SLC39A12	solute carrier family 39 (zinc transporter), member 12
112545	c	oo+++o+++	ASH1L	ash1 (absent, small, or homeotic)-like (Drosophila)
112571	b	ooo+ooooo	AMPD2	adenosine monophosphate deaminase 2 (isoform L)
112622	h	o-ooooooo	ODF2L	outer dense fiber of sperm tails 2-like
112626	a	ooo++++++	MAP7	microtubule-associated protein 7
112648	b	ooo+ooooo	THRAP4	thyroid hormone receptor associated protein 4
112661	b	ooo+ooooo	SPTAN1	spectrin, alpha, non-erythrocytic 1 (alpha-fodrin)
112721	b	oooo++ooo	BCMO1	beta-carotene 15,15'-monooxygenase 1
112723	b	ooo+ooooo	MAN2A2	mannosidase, alpha, class 2A, member 2
112804	a	ooo++++++	NET1	neuroepithelial cell transforming gene 1
112888	b	oo+oooooo	IFIT1L	interferon-induced protein with tetratricopeptide repeats 1-like
112906	b	oooo+oooo	null	null
112937	a	ooooo++++	null	null
112994	b	ooo++oooo	OXGR1	oxoglutarate (alpha-ketoglutarate) receptor 1
113036	b	ooo+ooooo	PARD6B	par-6 partitioning defective 6 homolog beta (C. elegans)
113042	b	ooo+ooooo	FGF2	fibroblast growth factor 2 (basic)
113058	a	o++++++++	KIAA1984	KIAA1984
113067	b	oooo+oooo	SAMD10	sterile alpha motif domain containing 10
113083	g	ooooooo--	CMTM4	CKLF-like MARVEL transmembrane domain containing 4
113096	d	ooo+o----	TXNDC13	thioredoxin domain containing 13
113105	g	oo-------	null	null
113118	b	ooo+ooooo	CRNKL1	Crn, crooked neck-like 1 (Drosophila)
113126	c	oooo+o+++	PMP2	peripheral myelin protein 2
113163	g	o--------	null	null
113190	b	ooo+ooooo	LILRB2|LILRB3	leukocyte immunoglobulin-like receptor, subfamily B (with TM and ITIM domains), member 2|leukocyte immunoglobulin-like receptor, subfamily B (with TM and ITIM domains), member 3
113197	a	ooo++++++	PELI1	pellino homolog 1 (Drosophila)
113212	b	ooo+++ooo	C14orf159	chromosome 14 open reading frame 159
113229	b	ooooo+ooo	null	null
113262	b	ooooo+ooo	COL3A1	collagen, type III, alpha 1 (Ehlers-Danlos syndrome type IV, autosomal dominant)
113291	b	ooooooo+o	MAGEB1	melanoma antigen family B, 1
113317	a	oooooo+++	CSPP1	centrosome and spindle pole associated protein 1
113318	h	o-ooooooo	ANKRD18A	ankyrin repeat domain 18A
113324	b	ooo+ooooo	EDD1	E3 ubiquitin protein ligase, HECT domain containing, 1
113330	c	oooo+o+++	null	null
113347	b	ooo+ooooo	DDHD2	DDHD domain containing 2
113356	g	oooooooo-	RPAP1	RNA polymerase II associated protein 1
113367	b	ooooo+ooo	LOC399737	null
113482	b	ooo+++ooo	ABCG4	ATP-binding cassette, sub-family G (WHITE), member 4
113538	b	oooo+oooo	LOC441076|DOCK9	dedicator of cytokinesis 9
113583	b	ooo+ooooo	TUBGCP3	tubulin, gamma complex associated protein 3
113643	b	ooo+ooooo	IQCF2	IQ motif containing F2
113657	b	ooooooo+o	TPK1	thiamin pyrophosphokinase 1
113678	b	ooo+ooooo	CAD	carbamoyl-phosphate synthetase 2, aspartate transcarbamylase, and dihydroorotase
113737	a	ooo++++++	TRIB3	tribbles homolog 3 (Drosophila)
113764	g	oo-------	IFRG15	null
113795	g	oooooooo-	CCL13	chemokine (C-C motif) ligand 13
113797	c	ooo+oo+++	GFPT2	glutamine-fructose-6-phosphate transaminase 2
113803	g	o--------	ZNF177	zinc finger protein 177
113820	a	ooooo++++	SERTAD3	SERTA domain containing 3
113849	a	oooooo+++	null	null
113872	b	ooooo+ooo	null	null
113933	a	oo+++++++	IL23A	interleukin 23, alpha subunit p19
113961	a	ooo++++++	DKFZp547G183	null
113962	b	ooo+ooooo	RPGR	retinitis pigmentosa GTPase regulator
113975	a	oooooo+++	UTP14A	UTP14, U3 small nucleolar ribonucleoprotein, homolog A (yeast)

114017	a	oo+++++++	HNRPH3	heterogeneous nuclear ribonucleoprotein H3 (2H9)
114037	h	o-ooooooo	CDKL3	cyclin-dependent kinase-like 3
114122	h	o-ooooooo	GNG12	guanine nucleotide binding protein (G protein), gamma 12
114141	a	oooooooo+	SCGB3A2	secretoglobin, family 3A, member 2
114188	b	ooooooo+o	S100B	S100 calcium binding protein, beta (neural)
114200	b	ooooo+ooo	DZIP1L	DAZ interacting protein 1-like
114223	b	ooo+ooooo	null	null
114228	b	ooooooo+o	MST1	macrophage stimulating 1 (hepatocyte growth factor-like)
114236	a	oo+++++++	MSH5	mutS homolog 5 (E. coli)
114238	i	o-----o--	KLKB1	kallikrein B, plasma (Fletcher factor) 1
114284	b	oooo++ooo	CORO1A	coronin, actin binding protein, 1A
114301	b	oooooo+oo	OATL1	ornithine aminotransferase-like 1
114304	a	ooo++++++	HOXC13	homeobox C13
114335	g	oooooooo-	EFHD1	EF-hand domain family, member D1
114353	h	o-ooooooo	null	null
114356	a	oo+++++++	ACSL3	acyl-CoA synthetase long-chain family member 3
114396	a	oooooooo+	null	null
114398	b	oooo+oooo	LRRC4C	leucine rich repeat containing 4C
114403	g	oooooooo-	null	null
114516	b	oooo+oooo	null	null
114538	b	ooo+ooooo	POLR1B	polymerase (RNA) I polypeptide B, 128kDa
114539	b	ooo+ooooo	EPB41	erythrocyte membrane protein band 4.1 (elliptocytosis 1, RH-linked)
114650	c	ooo+oo+++	SERPINB5	serpin peptidase inhibitor, clade B (ovalbumin), member 5
114661	a	oooooo+++	HNRPA0	heterogeneous nuclear ribonucleoprotein A0
114680	b	ooooo+ooo	STAC3	SH3 and cysteine rich domain 3
114728	b	oooo+oooo	FUT10	fucosyltransferase 10 (alpha (1,3) fucosyltransferase)
114758	a	oooooo+++	182-FIP	null
114791	h	oooooo--o	ZNF652	zinc finger protein 652
114799	b	ooooo+ooo	EBF2	early B-cell factor 2
114806	b	ooo+ooooo	LRP12	low density lipoprotein-related protein 12
114843	b	ooo+ooooo	ATP6V0A2	ATPase, H+ transporting, lysosomal V0 subunit a isoform 2
114854	g	o--------	null	null
114868	a	ooo++++++	FNBP4	formin binding protein 4
114882	a	oo+++++++	KIAA0907	KIAA0907
114901	b	ooo+ooooo	DMN	desmuslin
114911	b	oooo+oooo	MGC33584	null
114921	b	ooo+ooooo	LOC389992	null
115022	a	ooo++++++	SLC30A1	solute carrier family 30 (zinc transporter), member 1
115034	a	ooo++++++	USP25	ubiquitin specific peptidase 25
115052	b	ooooo+ooo	null	null
115055	c	ooo+oo+++	C2orf26	chromosome 2 open reading frame 26
115065	b	ooo+ooooo	KIAA1632	KIAA1632
115081	b	oo++++++o	null	null
115093	c	ooo+ooo++	ZNF717	zinc finger protein 717
115103	b	ooo+ooooo	PUM2	pumilio homolog 2 (Drosophila)
115141	b	ooo+ooooo	COG7	component of oligomeric golgi complex 7
115178	a	oooooo+++	TRAF4	TNF receptor-associated factor 4
115181	b	oo+oooooo	CES1	carboxylesterase 1 (monocyte/macrophage serine esterase 1)
115186	b	ooo+ooooo	SLC2A13	solute carrier family 2 (facilitated glucose transporter), member 13
115210	b	ooooooo+o	null	null
115220	i	o--o-----	ZDHHC2	zinc finger, DHHC-type containing 2
115249	g	o--------	SFRS5	splicing factor, arginine/serine-rich 5
115298	a	o++++++++	NR2C1	nuclear receptor subfamily 2, group C, member 1
115353	a	oo+++++++	DUSP6	dual specificity phosphatase 6
115354	g	ooooo----	KIF4A	kinesin family member 4A
115368	b	oooooo+oo	AP2A1	adaptor-related protein complex 2, alpha 1 subunit
115387	e	oooooo+-o	COL23A1	collagen, type XXIII, alpha 1
115397	b	o+ooooooo	RTN4R	reticulon 4 receptor
115455	b	oooo+oooo	RAB28	RAB28, member RAS oncogene family
115487	b	ooo+ooooo	RAD54B	RAD54 homolog B (S. cerevisiae)
115495	a	oooooooo+	C9orf50	chromosome 9 open reading frame 50
115500	b	ooo+ooooo	SLITRK5	SLIT and NTRK-like family, member 5
115543	b	o+ooooooo	HELB	helicase (DNA) B
115558	a	ooooo++++	GAS5	growth arrest-specific 5
115568	b	ooo+ooooo	ZBTB16	zinc finger and BTB domain containing 16
115643	b	oooo+oooo	COL6A3	collagen, type VI, alpha 3
115648	b	ooooo+ooo	RBM27	RNA binding motif protein 27
115702	g	o--------	GON4	gon-4 homolog (C.elegans)
115780	h	oooo----o	AMY2B	amylase, alpha 2B; pancreatic
115799	b	ooo+ooooo	RSBN1L	round spermatid basic protein 1-like
115809	b	ooo+ooooo	SCML2	sex comb on midleg-like 2 (Drosophila)

115847	f	oo++----+	MS4A1	membrane-spanning 4-domains, subfamily A, member 1
115882	a	ooo++++++	CHP	null
115883	b	ooo+ooooo	ABCC2	ATP-binding cassette, sub-family C (CFTR/MRP), member 2
115905	a	o++++++++	UGCGL1	UDP-glucose ceramide glucosyltransferase-like 1
115924	b	ooo+ooooo	SESN1	sestrin 1
115927	g	o--------	ARPM1	null
115928	b	ooo+ooooo	NIN	ninein (GSK3B interacting protein)
115963	b	ooo++oooo	null	null
115993	a	ooo++++++	TBX3	T-box 3 (ulnar mammary syndrome)
116004	a	ooo++++++	RAPGEF2	Rap guanine nucleotide exchange factor (GEF) 2
116020	b	oo++++++o	ITGB1BP2	integrin beta 1 binding protein (melusin) 2
116034	b	ooo++oooo	PTPRM	protein tyrosine phosphatase, receptor type, M
116061	b	ooo+ooooo	HSZFP36	null
116074	b	oooo++ooo	OR51I1	olfactory receptor, family 51, subfamily I, member 1
116126	b	ooo+ooooo	TBCD	tubulin-specific chaperone d
116132	a	oooooo+++	PLN	phospholamban
116147	i	o-----o--	CNOT3	CCR4-NOT transcription complex, subunit 3
116177	b	oooo+oooo	null	null
116187	g	o--------	CDC5L	CDC5 cell division cycle 5-like (S. pombe)
116237	b	ooo+ooooo	MKLN1	muskelin 1, intracellular mediator containing kelch motifs
116243	b	ooo+ooooo	LOC352909	null
116331	h	o-ooooooo	EPIM	epimorphin
116372	b	ooo+ooooo	PLEKHA7	pleckstrin homology domain containing, family A member 7
116397	a	o++++++++	FKBP10	FK506 binding protein 10, 65 kDa
116404	b	ooo+ooooo	RASA1	RAS p21 protein activator (GTPase activating protein) 1
116441	b	ooo+ooooo	LTBP2	latent transforming growth factor beta binding protein 2
116509	b	ooooooo+o	IQCF3	IQ motif containing F3
116510	g	o--------	MAGEC3	melanoma antigen family C, 3
116551	a	oooooo+++	null	null
116577	h	o-ooooooo	LHX4	LIM homeobox 4
116611	f	oo++----+	null	null
116629	b	oooooo+oo	CST9L	cystatin 9-like (mouse)
116672	g	oooo-----	CRY2	cryptochrome 2 (photolyase-like)
116772	a	ooooo++++	LOC388796	null
116787	a	oo+++++++	TMED6	transmembrane emp24 protein transport domain containing 6
116793	a	ooooooo++	RGS2	regulator of G-protein signalling 2, 24kDa
116804	b	ooo+ooooo	PTPRA	protein tyrosine phosphatase, receptor type, A
116822	g	o--------	null	null
116825	g	oooooooo-	PKIG	protein kinase (cAMP-dependent, catalytic) inhibitor gamma
116829	g	ooo------	C6orf115	chromosome 6 open reading frame 115
116877	b	ooo+ooooo	KIF17	kinesin family member 17
116879	g	oo-------	null	null
116886	g	oooooooo-	LOC442331	null
116905	b	ooo++oooo	null	null
116907	b	ooo+ooooo	ZNF236	zinc finger protein 236
116915	b	ooo+++++o	LRRN1	leucine rich repeat neuronal 1
116916	b	ooooooo+o	SHB	Src homology 2 domain containing adaptor protein B
116930	b	ooo+ooooo	TOP2B	topoisomerase (DNA) II beta 180kDa
116932	a	o++++++++	FLJ21816	null
116936	c	oo++oooo+	FAM13A1	family with sequence similarity 13, member A1
117001	b	ooooooo+o	null	null
117014	b	o+ooooooo	C10orf79	chromosome 10 open reading frame 79
117055	b	oooo+oooo	C10orf80	chromosome 10 open reading frame 80
117064	b	ooo+ooooo	GNS	glucosamine (N-acetyl)-6-sulfatase (Sanfilippo disease IIID)
117067	b	o+ooooooo	PRRX1	paired related homeobox 1
117076	b	ooo+ooooo	ZNF272	zinc finger protein 272
117132	a	oooooo+++	TSC22D1	TSC22 domain family, member 1
117382	a	ooooooo++	FLJ11021	null
117386	b	ooooo+ooo	DMD	dystrophin (muscular dystrophy, Duchenne and Becker types)
117399	b	ooooooo+o	null	null
117435	b	ooo+ooooo	EP400	E1A binding protein p400
117466	g	oooo-----	COVA1	cytosolic ovarian carcinoma antigen 1
117528	d	ooo+oooo-	ABCC1	ATP-binding cassette, sub-family C (CFTR/MRP), member 1
117570	b	ooo++oooo	PGLYRP2	peptidoglycan recognition protein 2
117630	i	oooo-o---	null	null
117673	b	ooooooo+o	null	null
117683	b	ooooooo+o	TECTB	tectorin beta
117717	a	o++++++++	LPXN	leupaxin
117726	h	o-ooooooo	RBM16	RNA binding motif protein 16
117732	c	oo++oooo+	USP16	ubiquitin specific peptidase 16
117752	a	oooooo+++	CDC42EP2	CDC42 effector protein (Rho GTPase binding) 2

117763	b	ooooo+ooo	ADAM20	ADAM metallopeptidase domain 20
117765	i	o-----o--	PSCA	prostate stem cell antigen
117770	b	ooooo+ooo	null	null
117813	h	o-ooooooo	VCY|VCY1B	variable charge, Y-linked|variable charge, Y-linked 1B
117835	a	oo+++++++	null	null
117844	a	oooooooo+	MCEMP1	null
117851	h	oooooo-oo	GTF2A1	general transcription factor IIA, 1, 19/37kDa
117860	g	o--------	null	null
117864	a	ooooooo++	null	null
117895	b	ooo+ooooo	SCG2	secretogranin II (chromogranin C)
117902	b	ooo+ooooo	TERF2	telomeric repeat binding factor 2
117958	b	ooo+ooooo	ITGAV	integrin, alpha V (vitronectin receptor, alpha polypeptide, antigen CD51)
117960	g	oooo-----	CMAS	cytidine monophosphate N-acetylneuraminic acid synthetase
117987	i	o--oo----	null	null
118004	b	oooooo+oo	LOC124976	null
118205	g	o--------	PADI2	peptidyl arginine deiminase, type II
118274	b	ooo+ooooo	FZD3	frizzled homolog 3 (Drosophila)
118278	b	oooooo+oo	FBXO44	F-box protein 44
118348	b	oooo+oooo	TRIM21	tripartite motif-containing 21
118382	h	o-ooooooo	C6orf113	chromosome 6 open reading frame 113
118384	i	o-----o--	HGS	hepatocyte growth factor-regulated tyrosine kinase substrate
118398	b	ooo+ooooo	WASF1	WAS protein family, member 1
118403	a	oo+++++++	PRPF38B	PRP38 pre-mRNA processing factor 38 (yeast) domain containing B
118428	b	oooo+oooo	null	null
118443	b	ooo+ooooo	ALS2CR11	amyotrophic lateral sclerosis 2 (juvenile) chromosome region, candidate 11
118463	h	ooooooo-o	null	null
118482	b	ooo+ooooo	HRMT1L6	HMT1 hnRNP methyltransferase-like 6 (S. cerevisiae)
118485	a	oo+++++++	KIAA1018	KIAA1018
118492	b	o+ooooooo	STARD13	START domain containing 13
118529	b	ooooo+ooo	HLF	hepatic leukemia factor
118556	i	o--oo----	SORCS2	sortilin-related VPS10 domain containing receptor 2
118564	a	o++++++++	SMAP1	stromal membrane-associated protein 1
118602	g	ooooooo--	FLJ14981	null
118612	b	o+ooooooo	PRRX2	paired related homeobox 2
118620	a	ooo++++++	JMJD1A	jumonji domain containing 1A
118630	g	o--------	MLZE	melanoma-derived leucine zipper, extra-nuclear factor
118653	b	ooo++oooo	LPIN2	lipin 2
118687	i	o-oo-----	FLJ10781	null
118692	b	oooo+oooo	null	null
118811	b	ooooo+ooo	MGC27165	null
118816	b	ooo+++++o	CITED1	Cbp/p300-interacting transactivator, with Glu/Asp-rich carboxy-terminal domain, 1
118830	b	ooo+ooooo	KIF20A	kinesin family member 20A
118877	h	o-ooooooo	BMP1	bone morphogenetic protein 1
118880	b	o+ooooooo	KIAA1276	null
118930	a	ooo++++++	TXNIP	thioredoxin interacting protein
118978	h	oooooo-oo	IL17F	interleukin 17F
118988	a	ooo++++++	null	null
119003	b	ooo+ooooo	C14orf58	chromosome 14 open reading frame 58
119024	b	ooo++oooo	SATB2	SATB family member 2
119103	i	o-oo-----	GOLGA5	golgi autoantigen, golgin subfamily a, 5
119131	a	ooooo++++	CSTF2T	cleavage stimulation factor, 3' pre-RNA, subunit 2, 64kDa, tau variant
119132	b	ooooooo+o	LRRK2	leucine-rich repeat kinase 2
119156	a	oooooo+++	MYLIP	myosin regulatory light chain interacting protein
119157	b	ooo+ooooo	PTPRN	protein tyrosine phosphatase, receptor type, N
119175	b	oooooo+oo	N-PAC	null
119183	b	o+ooooooo	PLCXD1	phosphatidylinositol-specific phospholipase C, X domain containing 1
119184	b	ooo+ooooo	null	null
119187	g	oooooooo-	BET1L	blocked early in transport 1 homolog (S. cerevisiae)-like
119218	b	oooo+oooo	null	null
119299	h	oooooo--o	LOC283219	null
119300	b	ooooo+ooo	FSCN2	fascin homolog 2, actin-bundling protein, retinal (Strongylocentrotus purpuratus)
119323	i	o-----o--	BAT2	HLA-B associated transcript 2
119364	a	oo+++++++	null	null
119391	c	oo++o++++	null	null
119429	a	oooooooo+	ZNF228	zinc finger protein 228
119475	g	o--------	FLJ16331	null
119484	h	o-ooooooo	GNG12	guanine nucleotide binding protein (G protein), gamma 12
119525	g	ooooooo--	FKBP7	FK506 binding protein 7
119539	g	o--------	FLJ20245	null
119663	a	oooooo+++	CRTC3	CREB regulated transcription coactivator 3

119690	b	ooo+ooooo	EML1	echinoderm microtubule associated protein like 1
119693	h	o-ooooooo	DPP3	dipeptidylpeptidase 3
119727	a	ooo++++++	HCP5	HLA complex P5
119729	b	oooo+oooo	AQP1	aquaporin 1 (channel-forming integral protein, 28kDa)
119730	i	o-oo-----	FAM38A	family with sequence similarity 38, member A
119754	j	o-ooooo++	FLJ37953	null
119787	b	ooo++oooo	ACOX1	acyl-Coenzyme A oxidase 1, palmitoyl
119826	b	ooo+ooooo	HACE1	HECT domain and ankyrin repeat containing, E3 ubiquitin protein ligase 1
119847	b	ooo+ooooo	KLHL13	kelch-like 13 (Drosophila)
119872	b	oooo+oooo	NALP6	NACHT, leucine rich repeat and PYD containing 6
119925	i	o-oo-----	MGC42174	null
119955	c	oo++o++++	PRKAR2A	protein kinase, cAMP-dependent, regulatory, type II, alpha
119958	h	o-ooooooo	HPS5	Hermansky-Pudlak syndrome 5
119977	g	ooooooo--	ADA	adenosine deaminase
120001	g	oo-------	NASP	nuclear autoantigenic sperm protein (histone-binding)
120032	a	ooo++++++	EIF2C2	eukaryotic translation initiation factor 2C, 2
120075	b	ooo+ooooo	AP3M2	adaptor-related protein complex 3, mu 2 subunit
120129	a	ooo++++++	ETAA16	null
120135	b	ooooooo+o	PTPRH	protein tyrosine phosphatase, receptor type, H
120144	b	ooo+ooooo	GTF3C1	general transcription factor IIIC, polypeptide 1, alpha 220kDa
120159	g	o--------	KCNJ3	potassium inwardly-rectifying channel, subfamily J, member 3
120176	a	ooo++++++	TOPBP1	topoisomerase (DNA) II binding protein 1
120204	b	oooooo++o	ALS2CR15	amyotrophic lateral sclerosis 2 (juvenile) chromosome region, candidate 15
120256	b	ooooo+ooo	null	null
120260	a	oo+++++++	C1orf54	chromosome 1 open reading frame 54
120459	b	oooo+++oo	null	null
120497	b	ooo+ooooo	WDR21A	WD repeat domain 21A
120502	b	ooo+ooooo	CCHCR1	coiled-coil alpha-helical rod protein 1
120568	a	oo+++++++	DDAH2	dimethylarginine dimethylaminohydrolase 2
120596	g	oooo-----	RAB3A	RAB3A, member RAS oncogene family
120624	b	ooo++oooo	FOXO1A	forkhead box O1A (rhabdomyosarcoma)
120645	g	ooo------	null	null
120648	i	o-oo-----	PDZK6	PDZ domain containing 6
120716	b	ooooooo+o	RAPGEF4	Rap guanine nucleotide exchange factor (GEF) 4
120734	h	oo-oooooo	FADD	Fas (TNFRSF6)-associated via death domain
120791	b	ooo+ooooo	NOSTRIN	nitric oxide synthase trafficker
120823	b	ooo+ooooo	FLJ32926	null
120851	b	ooo+ooooo	TXNRD3	thioredoxin reductase 3
120876	g	oo-------	MYO15A	myosin XVA
120923	b	oooo+oooo	AQP9	aquaporin 9
120930	a	o++++++++	GOLGA8A	golgi autoantigen, golgin subfamily a, 8A
121009	g	oo-------	BRI3	brain protein I3
121011	b	oooooo+oo	ASNA1	arsA arsenite transporter, ATP-binding, homolog 1 (bacterial)
121028	j	o-o++++++	H1F0	H1 histone family, member 0
121040	b	ooo+ooooo	MEIS1	Meis1, myeloid ecotropic viral integration site 1 homolog (mouse)
121081	a	ooo++++++	TNFRSF21	tumor necrosis factor receptor superfamily, member 21
121141	b	ooo+ooooo	ABCB10	ATP-binding cassette, sub-family B (MDR/TAP), member 10
121216	b	ooooo+ooo	ACY3	aspartoacylase (aminocyclase) 3
121217	b	oooooo++o	KRT2A	keratin 2A (epidermal ichthyosis bullosa of Siemens)
121229	a	oo+++++++	RP9	retinitis pigmentosa 9 (autosomal dominant)
121271	b	ooooooo+o	DOK2	docking protein 2, 56kDa
121286	b	ooo+ooooo	CUGBP2	CUG triplet repeat, RNA binding protein 2
121304	h	ooo-ooooo	SDF2	stromal cell-derived factor 2
121365	b	oooo+oooo	C6orf136	chromosome 6 open reading frame 136
121369	j	oooo----+	FBXO41	F-box protein 41
121371	b	ooo+ooooo	MXRA8	matrix-remodelling associated 8
121408	h	oooooo-oo	OAF	OAF homolog (Drosophila)
121548	i	o-----o--	PDLIM7	PDZ and LIM domain 7 (enigma)
121583	a	ooooo++++	FAM100B	family with sequence similarity 100, member B
121600	b	ooooo+ooo	HIST1H1T	histone 1, H1t
121612	a	ooo++++++	DUSP5	dual specificity phosphatase 5
121618	b	oooooo+oo	KCNMB4	potassium large conductance calcium-activated channel, subfamily M, beta member 4
121636	b	oooooo+oo	FAM3B	family with sequence similarity 3, member B
121646	g	o--------	ACIN1	apoptotic chromatin condensation inducer 1
121661	b	ooo+ooooo	USP6NL	USP6 N-terminal like
121681	f	oo++----+	FLJ20345	null
121690	a	oo+++++++	STK19	serine/threonine kinase 19
121695	j	o-ooooo++	CDKN1B	cyclin-dependent kinase inhibitor 1B (p27, Kip1)
121701	b	oooooo+oo	null	null
121716	b	ooo+++++o	null	null

121741	a	ooo++++++	USP6	ubiquitin specific peptidase 6 (Tre-2 oncogene)
121831	b	oooooo+oo	AKT2	v-akt murine thymoma viral oncogene homolog 2
121840	a	oooooo+++	EDN1	endothelin 1
121876	b	oo++++++o	C17orf28	chromosome 17 open reading frame 28
121941	h	o-ooooooo	EML4	echinoderm microtubule associated protein like 4
121944	h	ooooooo-o	FAM26B	family with sequence similarity 26, member B
121972	b	ooo+ooooo	MKKS	McKusick-Kaufman syndrome
121998	a	ooo++++++	HEY1	hairy/enhancer-of-split related with YRPW motif 1
122010	h	oooooo--o	NXPH4	neurexophilin 4
122040	h	o-ooooooo	SLC25A36	solute carrier family 25, member 36
122102	b	oooooo+oo	NUCB1	nucleobindin 1
122171	c	ooo+ooo++	null	null
122205	b	oooo+oooo	LOC338862	null
122246	a	ooo++++++	UBXD7	UBX domain containing 7
122271	g	oooooo---	FAM8A1	family with sequence similarity 8, member A1
122331	b	ooo+ooooo	DIO2	deiodinase, iodothyronine, type II
122333	b	ooo+ooooo	FLJ21901	null
122389	b	ooo+ooooo	MBD5	methyl-CpG binding domain protein 5
122393	b	oooo+oooo	AGER	advanced glycosylation end product-specific receptor
122394	h	o-ooooooo	RAB23	RAB23, member RAS oncogene family
122400	h	o-ooooooo	ACP1	acid phosphatase 1, soluble
122515	g	oooo-----	BDH1	3-hydroxybutyrate dehydrogenase, type 1
122538	b	ooo+ooooo	NSD1	nuclear receptor binding SET domain protein 1
122544	b	ooo+ooooo	TFIP11	tuftelin interacting protein 11
122554	a	oooooo+++	HIST1H2AJ	histone 1, H2aj
122603	a	o++++++++	ADAM8	ADAM metallopeptidase domain 8
122628	a	ooooooo++	HEL308	null
122658	b	ooo+ooooo	null	null
122660	a	oo+++++++	GLCE	null
122698	h	o-ooooooo	PITPNB	phosphatidylinositol transfer protein, beta
122713	g	oooooooo-	FLJ14107	null
122728	i	o-oo-----	DPY19L1	dpy-19-like 1 (C. elegans)
122820	i	o------o-	TM4SF20	transmembrane 4 L six family member 20
122847	b	oooo+oooo	SLC9A7	solute carrier family 9 (sodium/hydrogen exchanger), member 7
122848	b	ooo+ooooo	FAM19A1	family with sequence similarity 19 (chemokine (C-C motif)-like), member A1
122854	b	ooo+ooooo	WDR37	WD repeat domain 37
122912	a	ooo++++++	KLF9	Kruppel-like factor 9
122980	b	oooo+oooo	MGC40178	null
122985	a	o++++++++	INPP5F	inositol polyphosphate-5-phosphatase F
123012	c	ooo+ooo++	JAG1	jagged 1 (Alagille syndrome)
123028	b	oo+++oooo	MGC35366	null
123054	g	ooooo----	PI4K2B	phosphatidylinositol 4-kinase type 2 beta
123056	b	oooo+oooo	null	null
123057	b	oo+++oooo	KIAA1799	null
123066	b	oooo+oooo	null	null
123139	h	o-ooooooo	SYNE1	spectrin repeat containing, nuclear envelope 1
123142	b	ooo+ooooo	null	null
123143	h	ooo-ooooo	AREG	amphiregulin (schwannoma-derived growth factor)
123186	g	o--------	SAMD1	sterile alpha motif domain containing 1
123195	g	o--------	TLCD1	TLC domain containing 1
123217	b	ooo+ooooo	null	null
123229	a	oo+++++++	LOC441395	null
123231	c	oooo+ooo+	SYTL5	synaptotagmin-like 5
123246	b	ooooooo+o	RPS4Y1	ribosomal protein S4, Y-linked 1
123258	b	ooooo++oo	DBH	dopamine beta-hydroxylase (dopamine beta-monooxygenase)
123273	a	ooo++++++	JUN	v-jun sarcoma virus 17 oncogene homolog (avian)
123279	c	oo++oooo+	ULK4	unc-51-like kinase 4 (C. elegans)
123331	a	o++++++++	SSH2	slingshot homolog 2 (Drosophila)
123368	b	ooo+ooooo	DDEFL1	development and differentiation enhancing factor-like 1
123383	b	ooo++oooo	null	null
123395	a	oooooooo+	EPOR	erythropoietin receptor
123427	b	ooo+ooooo	MGC5370	null
123438	a	ooo++++++	FLJ14154	null
123450	a	oo+++++++	NR4A2	nuclear receptor subfamily 4, group A, member 2
123514	b	ooo+ooooo	COX15	COX15 homolog, cytochrome c oxidase assembly protein (yeast)
123518	b	ooo+ooooo	RP11-301I17.1	null
123565	i	o-oo-----	TNRC4	trinucleotide repeat containing 4
123631	b	oooooo+oo	AKT1S1	AKT1 substrate 1 (proline-rich)
123641	g	o--------	IRAK1	interleukin-1 receptor-associated kinase 1
123680	b	ooooooo+o	C20orf70	chromosome 20 open reading frame 70
123699	h	o--oooooo	FZD10	frizzled homolog 10 (Drosophila)

123724	b	oooooo+oo	RAB17	RAB17, member RAS oncogene family
123743	a	oo+++++++	PDE3A	phosphodiesterase 3A, cGMP-inhibited
123781	b	o+ooooooo	GABRA4	gamma-aminobutyric acid (GABA) A receptor, alpha 4
123816	a	oooooooo+	null	null
123821	h	o-ooooooo	LOC153364	null
123933	a	oo+++++++	MLL3	myeloid/lymphoid or mixed-lineage leukemia 3
123950	g	oooooooo-	CPNE2	copine II
123956	c	ooo+oo+++	ZBTB2	zinc finger and BTB domain containing 2
123965	g	o--------	null	null
123978	b	oooo+oooo	FLJ40919	null
124011	i	o---o----	null	null
124025	b	ooo+ooooo	PREP	prolyl endopeptidase
124034	b	oooooo+oo	null	null
124049	g	o--------	KIAA0141	KIAA0141
124073	h	oooo----o	ADARB2	adenosine deaminase, RNA-specific, B2 (RED2 homolog rat)
124110	b	oooooo+oo	DLX1	distal-less homeobox 1
124168	b	oooooo+oo	FLJ13310	null
124258	f	oo++----+	null	null
124270	b	ooo+ooooo	COL12A1	collagen, type XII, alpha 1
124281	b	oooooo+oo	TNRC5	trinucleotide repeat containing 5
124319	h	o-ooooooo	null	null
124368	b	ooo+ooooo	EPB41L2	erythrocyte membrane protein band 4.1-like 2
124416	b	oo+++oooo	FBN2	fibrillin 2 (congenital contractural arachnodactyly)
124424	a	ooooo++++	JMJD3	jumonji domain containing 3
124456	h	o-ooooooo	DKFZP686A01247	null
124473	a	oooooooo+	AQP7	aquaporin 7
124485	b	ooooooo+o	FBXO27	F-box protein 27
124516	a	ooo++++++	C14orf138	chromosome 14 open reading frame 138
124521	b	oooooo+oo	LOC388383	null
124591	a	ooo++++++	C9orf82	chromosome 9 open reading frame 82
124691	h	o-ooooooo	CYB5-M	null
124709	h	o-ooooooo	LOC157381	null
124810	b	o+ooooooo	LOC441137	null
124834	h	oooo---oo	null	null
124848	h	o--oooooo	HAS2	hyaluronan synthase 2
124857	a	o++++++++	UTX	ubiquitously transcribed tetratricopeptide repeat, X chromosome
124878	a	ooo++++++	TMC5	transmembrane channel-like 5
124894	f	oo++----+	null	null
124931	b	ooo+ooooo	LNK	null
124952	g	o--------	null	null
124977	g	o--------	ARGFX	arginine-fifty homeobox
125028	a	oooooo+++	KLF10	Kruppel-like factor 10
125051	b	ooo++oooo	N4BP2	null
125054	b	ooo+ooooo	PITRM1	pitrilysin metallopeptidase 1
125061	b	ooooooo+o	MGC17330	null
125079	g	ooooooo--	INT1	null
125084	a	oo+++++++	FAM80B	family with sequence similarity 80, member B
125108	b	ooo+ooooo	ANKRD11	ankyrin repeat domain 11
125134	b	oooooo++o	DBP	D site of albumin promoter (albumin D-box) binding protein
125148	a	o++++++++	C9orf100	chromosome 9 open reading frame 100
125158	b	oooo+oooo	FBP1	fructose-1,6-bisphosphatase 1
125176	b	oooo+oooo	null	null
125210	g	oooooooo-	HDLBP	high density lipoprotein binding protein (vigilin)
125216	b	ooo++oooo	TMC7	transmembrane channel-like 7
125219	a	oooooo+++	TSPYL1	TSPY-like 1
125286	b	oooo+oooo	ECAT8	null
125299	b	ooo+ooooo	RPS6KB1	ribosomal protein S6 kinase, 70kDa, polypeptide 1
125345	b	ooo+ooooo	RFXDC2	regulatory factor X domain containing 2
125364	b	ooo+ooooo	KIAA1586	KIAA1586
125374	f	oo++----+	MGC72075	null
125410	g	o--------	DKFZp686D0972	null
125454	b	ooo+ooooo	FOXJ2	forkhead box J2
125509	a	ooooo++++	FLJ32363	null
125537	b	oooooo+oo	LOC440066	null
125539	a	oo+++++++	PILRB	paired immunoglobin-like type 2 receptor beta
125547	b	oooo+oooo	null	null
125566	f	oo++----+	LY6G6D	lymphocyte antigen 6 complex, locus G6D
125577	b	ooo+ooooo	MDM1	Mdm4, transformed 3T3 cell double minute 1, p53 binding protein (mouse)
125615	b	ooo+++ooo	GPR162	G protein-coupled receptor 162
125618	b	ooooo+ooo	FMOD	fibromodulin
125674	b	oooooo+oo	PDK2	pyruvate dehydrogenase kinase, isozyme 2

125681	c	ooo+oo+++	OVOS|OVOS2|LOC440080	null
125725	h	o-ooooooo	C5orf15	chromosome 5 open reading frame 15
125726	a	ooooo++++	TRGC2|TRGV2	T cell receptor gamma constant 2|T cell receptor gamma variable 2
125732	h	o-ooooooo	NPEPL1	aminopeptidase-like 1
125818	b	oooo+oooo	ATPBD4	ATP binding domain 4
125845	a	ooo++++++	TGIF2	TGFB-induced factor 2 (TALE family homeobox)
125897	b	oooooo+oo	MGC20983	null
125903	a	ooooo++++	C6orf48	chromosome 6 open reading frame 48
125908	b	ooooo+ooo	TYRP1	tyrosinase-related protein 1
125930	a	oooooo+++	CRI2	CREBBP/EP300 inhibitor 2
125937	a	ooo++++++	DHRS3	dehydrogenase/reductase (SDR family) member 3
125964	e	oo++----o	C6orf201	chromosome 6 open reading frame 201
126039	b	ooo+ooooo	FLJ21415	null
126044	h	o-ooooooo	PNMA2	paraneoplastic antigen MA2
126087	h	ooooo---o	GFRA2	GDNF family receptor alpha 2
126100	g	o--------	TMEM15	transmembrane protein 15
126109	b	ooo++oooo	null	null
126259	h	o-ooooooo	C13orf7	chromosome 13 open reading frame 7
126340	g	o--------	MC2R	melanocortin 2 receptor (adrenocorticotropic hormone)
126351	a	oooo+++++	RND1	Rho family GTPase 1
126367	c	oo++o++++	ORAOV1	oral cancer overexpressed 1
126400	b	oooooo+oo	APBA1	amyloid beta (A4) precursor protein-binding, family A, member 1 (X11)
126414	b	oooo+oooo	DENND2C	DENN/MADD domain containing 2C
126445	b	oooooo+oo	LOC441282	null
126484	b	ooo+ooooo	PCCA	propionyl Coenzyme A carboxylase, alpha polypeptide
126493	a	ooooooo++	SERPINA9	serpin peptidase inhibitor, clade A (alpha-1 antiproteinase, antitrypsin), member 9
126518	b	oooo+oooo	C1orf158	chromosome 1 open reading frame 158
126560	g	o--------	RGS17	regulator of G-protein signalling 17
126602	b	ooo+ooooo	RABGAP1L	RAB GTPase activating protein 1-like
126773	a	ooo++++++	TMEM77	transmembrane protein 77
126775	e	oo++----o	KCNG4	potassium voltage-gated channel, subfamily G, member 4
126816	b	ooooo+ooo	BAZ2B	bromodomain adjacent to zinc finger domain, 2B
126822	b	ooo+ooooo	DKFZP434G1415	null
126848	b	oooo+oooo	FLJ12056	null
126883	b	ooooo+ooo	ACYP2	acylphosphatase 2, muscle type
126953	f	oo++----+	SIGLEC12	sialic acid binding Ig-like lectin 12
126980	b	ooo+ooooo	TXNL2	thioredoxin-like 2
126989	g	oo-------	LOC341371	null
127007	b	oooo+oooo	FCGR2C|FCGR2A	Fc fragment of IgG, low affinity IIa, receptor (CD32)
127029	b	ooooooo+o	GIMAP4	GTPase, IMAP family member 4
127056	h	oooooo-oo	ZBTB17	zinc finger and BTB domain containing 17
127119	g	o--------	LOC613212	null
127161	g	o--------	KIAA0961	null
127162	b	ooo+ooooo	LOC286170	null
127163	b	ooo+ooooo	PSMF1	proteasome (prosome, macropain) inhibitor subunit 1 (PI31)
127226	f	oo++----+	SPRR2C	small proline-rich protein 2C
127242	a	ooo++++++	PMAIP1	phorbol-12-myristate-13-acetate-induced protein 1
127252	g	oooooooo-	BCL7C	B-cell CLL/lymphoma 7C
127276	b	ooo+ooooo	OR4F6	olfactory receptor, family 4, subfamily F, member 6
127331	b	oooooo+oo	C18orf34	chromosome 18 open reading frame 34
127385	b	ooo+ooooo	MLYCD	malonyl-CoA decarboxylase
127423	b	ooo+ooooo	DOCK7	dedicator of cytokinesis 7
127488	e	oo++----o	C10orf81	chromosome 10 open reading frame 81
127569	b	ooo+ooooo	FUBP3	far upstream element (FUSE) binding protein 3
127600	g	oooo-----	FNBP1	formin binding protein 1
127616	a	ooooooo++	G1P3	interferon, alpha-inducible protein (clone IFI-6-16)
127625	a	ooo++++++	CRTC3	CREB regulated transcription coactivator 3
127630	b	oo++ooooo	C13orf21	chromosome 13 open reading frame 21
127640	b	ooo+ooooo	ERO1L	ERO1-like (S. cerevisiae)
127645	a	oooooo+++	RUNX1	runt-related transcription factor 1 (acute myeloid leukemia 1; aml1 oncogene)
127680	a	oooooooo+	null	null
127713	b	ooo+ooooo	06-Sep	septin 6
127726	h	o-ooooooo	null	null
127735	b	ooo+ooooo	C10orf137	chromosome 10 open reading frame 137
127803	b	ooo+ooooo	PLEKHA5	pleckstrin homology domain containing, family A member 5
127828	b	ooo+ooooo	ARMC5	armadillo repeat containing 5
127831	b	oooo++ooo	MGC35030	null
127854	b	ooo+ooooo	C9orf55	chromosome 9 open reading frame 55
127896	a	oooo+++++	ATP10B	ATPase, Class V, type 10B
127904	g	o--------	HDGF2	null

127970	d	oooooo+--	null	null
127989	h	o-ooooooo	RWDD3	RWD domain containing 3
128076	g	ooooo----	TUBB	tubulin, beta
128130	a	ooooo++++	NOTCH2|NOTCH2NL	Notch homolog 2 (Drosophila)|Notch homolog 2 (Drosophila) N-terminal like
128176	a	ooo++++++	WAC	WW domain containing adaptor with coiled-coil
128236	a	oo+++++++	HNRPA3	heterogeneous nuclear ribonucleoprotein A3
128266	b	ooo+ooooo	NUFIP1	nuclear fragile X mental retardation protein interacting protein 1
128268	h	oooo-oooo	C1orf176	chromosome 1 open reading frame 176
128361	g	o--------	GDDR	null
128393	a	ooo++++++	TAS2R14	taste receptor, type 2, member 14
128414	b	ooo+ooooo	C14orf132	chromosome 14 open reading frame 132
128418	g	oooooooo-	PFAS	phosphoribosylformylglycinamidine synthase (FGAR amidotransferase)
128464	g	oooooooo-	DAZL	deleted in azoospermia-like
128572	b	ooooooo+o	null	null
128580	c	ooo+o++++	SETDB2	SET domain, bifurcated 2
128582	a	ooooooo++	null	null
128588	g	ooooooo--	PARP9	poly (ADP-ribose) polymerase family, member 9
128603	b	ooo++oooo	PHF2	PHD finger protein 2
128616	b	oo+++++oo	null	null
128671	g	oooooo---	EPPB9	null
128692	g	oooooooo-	GMPPA	GDP-mannose pyrophosphorylase A
128708	h	oo-oooooo	ZNF124	zinc finger protein 124 (HZF-16)
128722	b	oooo+oooo	ZNF433	zinc finger protein 433
128785	g	o--------	CD1B	CD1b antigen
128786	b	ooooooo+o	LRRC16	leucine rich repeat containing 16
128788	f	oo++----+	null	null
128800	g	oooo-----	RGS10	regulator of G-protein signalling 10
128831	b	oo+oooooo	null	null
128843	h	o-ooooooo	TNFAIP8	tumor necrosis factor, alpha-induced protein 8
128846	h	oooo-oooo	ALKBH	alkB, alkylation repair homolog (E. coli)
128863	a	o++++++++	EIF2B1	eukaryotic translation initiation factor 2B, subunit 1 alpha, 26kDa
128914	b	ooo+ooooo	SPHAR	null
128921	a	oo+++++++	ST7L	suppression of tumorigenicity 7 like
128929	g	o--------	FHAD1	forkhead-associated (FHA) phosphopeptide binding domain 1
128935	b	ooo++oooo	ZNF268	zinc finger protein 268
128961	h	oooo-oooo	TFPT	TCF3 (E2A) fusion partner (in childhood Leukemia)
128999	b	oooooo+oo	C1QTNF6	C1q and tumor necrosis factor related protein 6
129034	i	o-----o--	SPTBN2	spectrin, beta, non-erythrocytic 2
129035	h	o-------o	EVA1	epithelial V-like antigen 1
129046	c	ooo+oo+++	MGC70870	null
129120	g	o--------	CTAGE1	cutaneous T-cell lymphoma-associated antigen 1
129144	b	oo++ooooo	PKP4	plakophilin 4
129180	b	ooo+ooooo	TBL1X	transducin (beta)-like 1X-linked
129186	g	o--------	RAB22A	RAB22A, member RAS oncogene family
129189	a	oooooo+++	SNX13	sorting nexin 13
129200	h	oooo-oooo	GNPNAT1	glucosamine-phosphate N-acetyltransferase 1
129212	b	ooo+ooooo	COG3	component of oligomeric golgi complex 3
129229	b	ooooo++oo	RAB11B	RAB11B, member RAS oncogene family
129234	g	o--------	LOC441790	null
129254	b	oooo+oooo	CATSPER3	cation channel, sperm associated 3
129282	a	oooo+++++	AKR1C3	aldo-keto reductase family 1, member C3 (3-alpha hydroxysteroid dehydrogenase, type II)
129283	b	ooo+ooooo	WDR82	WD repeat domain 82
129290	b	oooooo+oo	SLC2A11	solute carrier family 2 (facilitated glucose transporter), member 11
129294	a	oooooooo+	null	null
129318	h	oooooo-oo	C20orf133	chromosome 20 open reading frame 133
129404	b	o+ooooooo	KIAA0888	null
129465	h	o-ooooooo	FLJ21820	null
129532	a	oooooooo+	null	null
129580	g	o--------	CNTN6	contactin 6
129583	b	ooo+ooooo	ARHGEF4	Rho guanine nucleotide exchange factor (GEF) 4
129603	b	ooo+ooooo	LOC388526	null
129624	b	ooooooo+o	null	null
129629	a	oo+++++++	USP52	ubiquitin specific peptidase 52
129638	b	oo+++oooo	CCDC18	coiled-coil domain containing 18
129653	b	ooo++oooo	FCAR	Fc fragment of IgA, receptor for
129668	a	ooo++++++	PPP4R1	protein phosphatase 4, regulatory subunit 1
129694	d	ooo+-----	KIAA1040	null
129735	b	ooo+ooooo	null	null

129786	b	oooo+oooo	DKFZp547E087|LOC23117|LOC388221|LOC440345	null
129829	j	o-oooo+++	RND3	Rho family GTPase 3
129845	b	oooo+oooo	SLC36A3	solute carrier family 36 (proton/amino acid symporter), member 3
129856	b	ooo+ooooo	NXPH2	neurexophilin 2
129907	b	ooooooo+o	LOC387887	null
129936	a	oooooooo+	RP11-56A21.1	null
130007	g	oooo-----	CNNM2	cyclin M2
130009	b	ooo+ooooo	PKD2	polycystic kidney disease 2 (autosomal dominant)
130015	b	ooooo+ooo	SNRPN	small nuclear ribonucleoprotein polypeptide N
130047	a	oooooo+++	ZNF394	zinc finger protein 394
130052	b	ooo+ooooo	TRPC4AP	transient receptor potential cation channel, subfamily C, member 4 associated protein
130135	j	o-oooo+++	SIAH1	seven in absentia homolog 1 (Drosophila)
130161	b	ooo+ooooo	ATP2C1	ATPase, Ca++ transporting, type 2C, member 1
130196	h	oooo----o	FLJ21986	null
130222	b	ooooo+ooo	null	null
130286	b	ooo+ooooo	FLJ14397	null
130287	a	o++++++++	GABRE	gamma-aminobutyric acid (GABA) A receptor, epsilon
130288	b	ooo+ooooo	WDR7	WD repeat domain 7
130304	d	ooo+oo---	NT5C2L1	5'-nucleotidase, cytosolic II-like 1
130314	a	oooooo+++	ZNF689	zinc finger protein 689
130335	b	ooo+ooooo	KIF13B	kinesin family member 13B
130353	b	oo++ooooo	null	null
130362	b	ooo+ooooo	TDG	thymine-DNA glycosylase
130401	a	o++++++++	FGF18	fibroblast growth factor 18
130492	h	oooo----o	null	null
130497	b	oooo+oooo	OR4A47	olfactory receptor, family 4, subfamily A, member 47
130502	b	ooo+ooooo	MBP	myelin basic protein
130505	b	ooo+ooooo	CDH1	cadherin 1, type 1, E-cadherin (epithelial)
130519	a	oooooo+++	TRAPPC6B	trafficking protein particle complex 6B
130573	h	o-ooooooo	null	null
130575	g	oooooo---	PTTG2	pituitary tumor-transforming 2
130579	g	oooooooo-	C9orf9	chromosome 9 open reading frame 9
130580	b	ooo+ooooo	null	null
130624	g	ooo------	SPBC25	spindle pole body component 25 homolog (S. cerevisiae)
130634	b	o++++oooo	ALG6	asparagine-linked glycosylation 6 homolog (yeast, alpha-1,3-glucosyltransferase)
130638	i	o---o----	null	null
130657	a	oo+++++++	DPPA2	developmental pluripotency associated 2
130675	a	o++++++++	TEX2	testis expressed sequence 2
130677	a	ooo++++++	IFIT2	interferon-induced protein with tetratricopeptide repeats 2
130678	g	o--------	PA2G4	proliferation-associated 2G4, 38kDa
130706	g	oooooooo-	CLPP	ClpP caseinolytic peptidase, ATP-dependent, proteolytic subunit homolog (E. coli)
130722	b	o+ooooooo	NAPB	N-ethylmaleimide-sensitive factor attachment protein, beta
130850	a	oo+++++++	null	null
130854	a	oo+++++++	DCLRE1C	DNA cross-link repair 1C (PSO2 homolog, S. cerevisiae)
130870	b	ooooooo+o	C20orf185	chromosome 20 open reading frame 185
130950	g	ooooooo--	WNT7B	wingless-type MMTV integration site family, member 7B
130951	a	ooo++++++	PTBP2	polypyrimidine tract binding protein 2
130956	b	ooo+ooooo	C1orf164	chromosome 1 open reading frame 164
131002	b	ooo+ooooo	CHD8	chromodomain helicase DNA binding protein 8
131021	b	ooo+ooooo	ZMYM4	zinc finger, MYM-type 4
131033	a	ooo++++++	DDX58	DEAD (Asp-Glu-Ala-Asp) box polypeptide 58
131083	b	oooo+oooo	null	null
131118	a	o++++++++	null	null
131190	a	oo+++++++	LAT2	linker for activation of T cells family, member 2
131232	a	ooo++++++	null	null
131321	a	oo+++++++	TEAD1	TEA domain family member 1 (SV40 transcriptional enhancer factor)
131347	a	oo+++++++	HIST2H2BE	histone 2, H2be
131379	b	ooo+ooooo	null	null
131459	b	oooo+oooo	C9orf18	chromosome 9 open reading frame 18
131493	i	o-----o--	null	null
131500	b	ooo+++ooo	ELMO1	engulfment and cell motility 1 (ced-12 homolog, C. elegans)
131502	b	oooooo+oo	RAB42	RAB42, member RAS homolog family
131512	g	oooooooo-	TMEM107	transmembrane protein 107
131558	b	ooo+ooooo	FAT	FAT tumor suppressor homolog 1 (Drosophila)
131604	b	oo+oooooo	LARP2	La ribonucleoprotein domain family, member 2
131654	g	o--------	TTTY1	testis-specific transcript, Y-linked 1
131673	i	o-----o--	PRAF2	PRA1 domain family, member 2
131680	a	oo+++++++	HOXB5	homeobox B5

131706	a	ooo++++++	CRKL	v-crk sarcoma virus CT10 oncogene homolog (avian)-like
131715	b	ooo+ooooo	RP11-308B5.5	null
131855	g	oooooooo-	GPR75	G protein-coupled receptor 75
131873	b	ooo+ooooo	APBB2	amyloid beta (A4) precursor protein-binding, family B, member 2 (Fe65-like)
131913	b	ooo+ooooo	MXD3	MAX dimerization protein 3
131928	h	o-ooooooo	MLL4	null
131963	b	ooo+ooooo	null	null
131968	a	oo+++++++	LOC285989	null
131975	a	oooooo+++	ING1	inhibitor of growth family, member 1
131980	j	o-oooooo+	OR2M7	olfactory receptor, family 2, subfamily M, member 7
132027	a	oooooo+++	IL18	interleukin 18 (interferon-gamma-inducing factor)
132046	b	ooo+ooooo	CISH	cytokine inducible SH2-containing protein
132057	a	oooooo+++	HIST1H2AC	histone 1, H2ac
132060	b	ooo+ooooo	FLJ20507	null
132093	b	ooo+ooooo	ACAD9	acyl-Coenzyme A dehydrogenase family, member 9
132109	a	oooooo+++	U2AF2	U2 (RNU2) small nuclear RNA auxiliary factor 2
132156	i	o-----o--	LOC90379	null
132159	a	o++++++++	DDHD1	DDHD domain containing 1
132221	h	o--oooooo	YPEL5	yippee-like 5 (Drosophila)
132230	l	o-----+--	MAZ	MYC-associated zinc finger protein (purine-binding transcription factor)
132276	h	oooo----o	APOBEC3G	apolipoprotein B mRNA editing enzyme, catalytic polypeptide-like 3G
132367	b	oooooo+oo	KCNH1	potassium voltage-gated channel, subfamily H (eag-related), member 1
132403	a	oo+++++++	FLJ20010	null
132463	b	o+ooooooo	LOC389323	null
132482	b	ooo+ooooo	BCL11A	B-cell CLL/lymphoma 11A (zinc finger protein)
132576	a	oo+++++++	LOC92270	null
132641	a	ooo++++++	SHOC2	soc-2 suppressor of clear homolog (C. elegans)
132666	a	ooo++++++	MYC	v-myc myelocytomatosis viral oncogene homolog (avian)
132699	b	oooo+oooo	PCNXL3	pecanex-like 3 (Drosophila)
132702	b	oooo+oooo	LOC440726	null
132722	b	oo+oooooo	ECE2	endothelin converting enzyme 2
132738	b	o+ooooooo	null	null
132765	b	ooo+ooooo	LOC441504	null
132797	b	ooo++oooo	C1orf34	chromosome 1 open reading frame 34
132812	b	ooo+ooooo	ZNF165	zinc finger protein 165
132825	b	ooo+ooooo	null	null
132835	h	o-ooooooo	FLJ14126	null
132884	i	o-oo-----	DHDH	dihydrodiol dehydrogenase (dimeric)
132892	a	oo+++++++	HIPK3	homeodomain interacting protein kinase 3
132929	b	oooooo+oo	CAPN1	calpain 1, (mu/I) large subunit
132930	b	ooooooo+o	NME5	non-metastatic cells 5, protein expressed in (nucleoside-diphosphate kinase)
132949	b	oooooo+oo	ZXDB|ZXDA	zinc finger, X-linked, duplicated B|zinc finger, X-linked, duplicated A
132984	a	o++++++++	null	null
132987	b	o+ooooooo	RLN2	relaxin 2
133013	b	ooooo+ooo	CHST4	carbohydrate (N-acetylglucosamine 6-O) sulfotransferase 4
133018	i	o-o------	null	null
133032	a	oo+++++++	FNDC3B	fibronectin type III domain containing 3B
133107	b	oooo++ooo	MCF2L2	MCF.2 cell line derived transforming sequence-like 2
133133	b	ooo+ooooo	SLK	STE20-like kinase (yeast)
133193	b	ooo+ooooo	IFIH1	interferon induced with helicase C domain 1
133236	b	ooo+ooooo	SRISNF2L	null
133244	h	o-ooooooo	ABCF2	ATP-binding cassette, sub-family F (GCN20), member 2
133249	d	ooo+oooo-	MGC15523	null
133302	b	ooooooo+o	CBLB	Cas-Br-M (murine) ecotropic retroviral transforming sequence b
133316	b	oooo+oooo	SLC25A27	solute carrier family 25, member 27
133317	b	oooooo+oo	KLK9	kallikrein 9
133326	a	ooo++++++	USP36	ubiquitin specific peptidase 36
133392	h	o-ooooooo	LOC441909	null
133410	a	oooooooo+	OR7G1	olfactory receptor, family 7, subfamily G, member 1
133470	b	ooooo+ooo	LOC127602	null
133487	h	oooo----o	EMID2	EMI domain containing 2
133510	b	ooo+ooooo	USP54	ubiquitin specific peptidase 54
133525	b	ooo++++oo	null	null
133539	b	ooo+ooooo	PGBD5	piggyBac transposable element derived 5
133574	c	ooo+++o++	FRAT1	frequently rearranged in advanced T-cell lymphomas
133600	j	o--ooo+++	ZNF331	zinc finger protein 331
133640	b	oo+oooooo	FLJ13391	null
133641	b	oo+oooooo	null	null
133654	a	ooooooo++	TP73	tumor protein p73
133712	b	ooooo+ooo	FLJ12355	null
133721	h	oooooo--o	ZNF643	zinc finger protein 643

133818	g	ooooooo--	null	null
133862	b	ooo+ooooo	PRRT1	proline-rich transmembrane protein 1
133923	g	oooooooo-	PBXIP1	pre-B-cell leukemia transcription factor interacting protein 1
133942	g	ooooooo--	C20orf98	chromosome 20 open reading frame 98
133973	b	ooooo+ooo	SULT1C1	sulfotransferase family, cytosolic, 1C, member 1
133979	a	oooooooo+	FLJ43374	null
133990	g	oo-------	LRRC48	leucine rich repeat containing 48
133995	b	ooo+ooooo	FAM91A1	family with sequence similarity 91, member A1
134015	a	ooo++++++	ZNF565	zinc finger protein 565
134034	b	oooo+oooo	MAGEB2	melanoma antigen family B, 2
134083	a	ooo++++++	UGCG	UDP-glucose ceramide glucosyltransferase
134084	g	o--------	BACH2	BTB and CNC homology 1, basic leucine zipper transcription factor 2
134128	b	ooo+ooooo	MYH11	myosin, heavy polypeptide 11, smooth muscle
134135	b	ooooooo+o	FLJ36445	null
134154	h	o-ooooooo	KIAA1604	null
134187	a	oo+++++++	MS4A3	membrane-spanning 4-domains, subfamily A, member 3 (hematopoietic cell-specific)
134215	c	ooo++o+++	YOD1	YOD1 OTU deubiquinating enzyme 1 homolog (yeast)
134236	c	oooooo+o+	GNL1	guanine nucleotide binding protein-like 1
134244	b	ooo+ooooo	C1orf16	chromosome 1 open reading frame 16
134259	b	ooo+ooooo	LRRC50	leucine rich repeat containing 50
134281	g	oo-------	TTC7B	tetratricopeptide repeat domain 7B
134284	b	ooo+ooooo	null	null
134287	a	ooooooo++	TMPRSS5	transmembrane protease, serine 5 (spinesin)
134321	i	o-oo-----	LHFPL2	lipoma HMGIC fusion partner-like 2
134377	b	ooo+ooooo	RCBTB1	regulator of chromosome condensation (RCC1) and BTB (POZ) domain containing protein 1
134437	b	ooo+ooooo	TAF1C	TATA box binding protein (TBP)-associated factor, RNA polymerase I, C, 110kDa
134475	b	ooo+ooooo	USP31	ubiquitin specific peptidase 31
134482	b	oooooo+oo	C8orf30A	chromosome 8 open reading frame 30A
134496	a	ooo++++++	PCNX	pecanex homolog (Drosophila)
134550	b	oooo+oooo	ZNF287	zinc finger protein 287
134551	b	ooooo+ooo	SERPINI1	serpin peptidase inhibitor, clade I (neuroserpin), member 1
134585	b	oooo+oooo	BAGE	B melanoma antigen
134641	b	ooooooo+o	null	null
134716	h	o-ooooooo	RBM23	RNA binding motif protein 23
134722	b	ooooo++oo	LOC441388	null
134760	b	ooo+ooooo	FGFRL1	fibroblast growth factor receptor-like 1
134780	g	oooooooo-	BLOC1S1	biogenesis of lysosome-related organelles complex-1, subunit 1
134901	b	ooo++oooo	SYCP2	synaptonemal complex protein 2
134996	h	oooo----o	null	null
135075	b	o+ooooooo	PADI1	peptidyl arginine deiminase, type I
135086	c	ooo+oo+++	CYP1A1	cytochrome P450, family 1, subfamily A, polypeptide 1
135135	h	o-ooooooo	PIK3C2A	phosphoinositide-3-kinase, class 2, alpha polypeptide
135158	a	oo+++++++	null	null
135242	i	o-oo-----	LOC401305|LOC339879|LOC442505	null
135343	b	ooo+ooooo	BDKRB2	bradykinin receptor B2
135364	g	o--------	CECR4	cat eye syndrome chromosome region, candidate 4
135435	b	ooo+ooooo	DMXL2	Dmx-like 2
135436	b	oooo+oooo	LOC401237	null
135443	a	ooooo++++	TCEAL1	transcription elongation factor A (SII)-like 1
135458	a	oooo+++++	CITED2	Cbp/p300-interacting transactivator, with Glu/Asp-rich carboxy-terminal domain, 2
135465	h	oooo----o	null	null
135473	a	o++++++++	CCDC11	coiled-coil domain containing 11
135517	a	ooo++++++	ADARB1	adenosine deaminase, RNA-specific, B1 (RED1 homolog rat)
135545	h	ooooo-ooo	LOC339351	null
135572	b	ooo+ooooo	AFG3L1	AFG3 ATPase family gene 3-like 1 (yeast)
135597	b	oooooo+oo	KCNK3	potassium channel, subfamily K, member 3
135630	g	oooooo---	MGC4707	null
135647	f	oo++----+	FLJ35808	null
135656	b	oooooo+oo	NPDC1	neural proliferation, differentiation and control, 1
135660	b	ooo+ooooo	SLC12A4	solute carrier family 12 (potassium/chloride transporters), member 4
135746	b	ooo+ooooo	RACGAP1	Rac GTPase activating protein 1
135772	b	oooo+oooo	null	null
135795	b	ooo+ooooo	C1orf170	chromosome 1 open reading frame 170
135806	a	ooooo++++	RNF113A	ring finger protein 113A
135867	a	o++++++++	FBXW10	F-box and WD-40 domain protein 10
135914	h	oooo----o	FOXC2	forkhead box C2 (MFH-1, mesenchyme forkhead 1)
135917	b	oooo+oooo	04-Sep	septin 4

135966	a	o++++++++	C21orf124	chromosome 21 open reading frame 124
135984	a	ooooo++++	C6orf160	chromosome 6 open reading frame 160
136008	a	ooo++++++	ARID4A	AT rich interactive domain 4A (RBP1-like)
136035	b	ooo+ooooo	PHACS	null
136076	g	o--------	CAPN11	calpain 11
136086	b	oooo+oooo	null	null
136096	b	ooo+ooooo	LOC399959	null
136109	g	oooooooo-	C14orf143	chromosome 14 open reading frame 143
136156	a	ooo++++++	SERPINF2	serpin peptidase inhibitor, clade F (alpha-2 antiplasmin, pigment epithelium derived factor), member 2
136170	g	o--------	null	null
136196	b	ooo+ooooo	LARS2	leucyl-tRNA synthetase 2, mitochondrial
136245	a	oo+++++++	NBPF14|NBPF10	neuroblastoma breakpoint family, member 14|neuroblastoma breakpoint family, member 10
136287	b	oooo+oooo	OSCAR	null
136294	g	ooo------	TXNRD2	thioredoxin reductase 2
136315	b	oooo+oooo	BGLAP|PMF1	bone gamma-carboxyglutamate (gla) protein (osteocalcin)|polyamine-modulated factor 1
136424	b	ooo+ooooo	COBL	cordon-bleu homolog (mouse)
136430	g	oooooooo-	SEPW1	selenoprotein W, 1
136439	b	ooo+ooooo	TRIM67	tripartite motif-containing 67
136502	a	o++++++++	NSUN4	NOL1/NOP2/Sun domain family, member 4
136504	a	ooo++++++	C11orf48	chromosome 11 open reading frame 48
136515	b	ooooooo+o	FLJ42486	null
136570	g	o--------	COL4A3	collagen, type IV, alpha 3 (Goodpasture antigen)
136571	a	o++++++++	C1orf60	chromosome 1 open reading frame 60
136576	g	o--------	FSHB	follicle stimulating hormone, beta polypeptide
136580	b	oooooo+oo	LMO6	LIM domain only 6
136598	a	oooooooo+	LOC342918	null
136625	b	ooo+ooooo	TMEM47	transmembrane protein 47
136634	a	o++++++++	LOC389264	null
136661	i	oo-----o-	SLC39A5	solute carrier family 39 (metal ion transporter), member 5
136663	b	ooo+ooooo	ABC1	null
136671	b	ooo+ooooo	FYCO1	FYVE and coiled-coil domain containing 1
136738	a	o++++++++	DKFZp761P1121	null
136743	b	ooo++oooo	NCOA1	nuclear receptor coactivator 1
136761	h	oooo----o	PRTN3	proteinase 3 (serine proteinase, neutrophil, Wegener granulomatosis autoantigen)
136897	h	o--oooooo	SNIP1	Smad nuclear interacting protein 1
136906	a	ooooo++++	CNO	cappuccino homolog (mouse)
136923	g	o--------	RSPO3	R-spondin 3 homolog (Xenopus laevis)
136978	a	ooo++++++	ADAMTS5	ADAM metallopeptidase with thrombospondin type 1 motif, 5 (aggrecanase-2)
137238	h	o-ooooooo	KCNH2	potassium voltage-gated channel, subfamily H (eag-related), member 2
137284	c	oooo+oo++	FLJ40311	null
137286	b	ooo+ooooo	RENT1	regulator of nonsense transcripts 1
137290	a	oooooooo+	FAM11B	family with sequence similarity 11, member B
137292	h	o-----ooo	CDKN1A	cyclin-dependent kinase inhibitor 1A (p21, Cip1)
137294	b	ooo+ooooo	VANGL1	vang-like 1 (van gogh, Drosophila)
137354	b	ooo++oooo	null	null
137355	g	o--------	RPIB9	null
137388	c	ooo+oo+++	PPM1D	protein phosphatase 1D magnesium-dependent, delta isoform
137546	g	ooooooo--	WNT16	wingless-type MMTV integration site family, member 16
137556	b	ooo+ooooo	NARG1L	NMDA receptor regulated 1-like
137559	b	ooo+ooooo	AGL	amylo-1, 6-glucosidase, 4-alpha-glucanotransferase (glycogen debranching enzyme, glycogen storage disease type III)
137567	b	ooo+ooooo	HIT-40|ZNF443	zinc finger protein 443
137592	h	oooooo-oo	null	null
137597	b	ooo+ooooo	null	null
137603	b	ooo+ooooo	MLXIP	MLX interacting protein
137620	b	oooooo+oo	UBTF	upstream binding transcription factor, RNA polymerase I
137648	h	o-ooooooo	PIM1	pim-1 oncogene
137656	g	ooooo----	MKI67	antigen identified by monoclonal antibody Ki-67
137683	b	ooo+ooooo	URG4	null
137695	b	ooo+ooooo	KIAA1598	KIAA1598
137772	a	oo+++++++	ZNF251	zinc finger protein 251
137804	a	oooooooo+	LOC391030	null
137897	h	oooo--ooo	TNFRSF12A	tumor necrosis factor receptor superfamily, member 12A
137911	b	ooo+ooooo	WDFY3	WD repeat and FYVE domain containing 3
137950	b	ooooooo+o	null	null
137970	b	oooooo+oo	XKR7	X Kell blood group precursor-related family, member 7
138012	h	oooo-oooo	C7orf30	chromosome 7 open reading frame 30
138045	b	oooo+oooo	MDFI	MyoD family inhibitor

138125	a	o++++++++	OVOS2	null
138127	a	ooo++++++	SCUBE2	signal peptide, CUB domain, EGF-like 2
138145	g	oooooooo-	LOC139431	null
138283	b	ooo+ooooo	null	null
138294	b	ooo+ooooo	ATP6V1E2	ATPase, H+ transporting, lysosomal 31kDa, V1 subunit E isoform 2
138302	g	oooo-----	LOC401308	null
138389	g	o--------	PIK3R2	phosphoinositide-3-kinase, regulatory subunit 2 (p85 beta)
138399	b	ooo+ooooo	LOC388690|LOC388692	null
138411	g	oooooooo-	MRPL16	mitochondrial ribosomal protein L16
138473	b	ooo+ooooo	FPGT	fucose-1-phosphate guanylyltransferase
138499	a	ooooooo++	CSHL1|CSH1|FCHO2	chorionic somatomammotropin hormone-like 1|chorionic somatomammotropin hormone 1 (placental lactogen)|FCH domain only 2
138515	g	o--------	CD74	CD74 antigen (invariant polypeptide of major histocompatibility complex, class II antigen-associated)
138553	g	ooooo----	ATRX	alpha thalassemia/mental retardation syndrome X-linked (RAD54 homolog, S. cerevisiae)
138592	h	oooo----o	null	null
138633	g	o--------	RHOD	ras homolog gene family, member D
138764	b	ooo+ooooo	PAPLN	papilin, proteoglycan-like sulfated glycoprotein
138768	b	oooo++ooo	STX11	syntaxin 11
138794	a	ooo++++++	SLC38A2	solute carrier family 38, member 2
138808	h	ooooooo-o	C10orf35	chromosome 10 open reading frame 35
138869	a	ooo++++++	FLJ22104	null
138876	b	ooo+ooooo	ZNF185	zinc finger protein 185 (LIM domain)
138955	b	ooo+ooooo	POLQ	polymerase (DNA directed), theta
138956	b	oooo+oooo	LOC131973	null
138968	h	o-ooooooo	null	null
139011	g	o--------	RDH10	retinol dehydrogenase 10 (all-trans)
139025	b	ooo+ooooo	UNQ9356	null
139029	b	ooooooo+o	GPR15	G protein-coupled receptor 15
139039	a	oooooo+++	DUS4L	dihydrouridine synthase 4-like (S. cerevisiae)
139060	b	ooooooo+o	SCGB1D1	secretoglobin, family 1D, member 1
139063	a	ooo++++++	null	null
139068	i	o-oo-----	CENTD1	centaurin, delta 1
139074	i	o-oo-----	PSG7	pregnancy specific beta-1-glycoprotein 7
139075	b	ooo+ooooo	PHLPP	PH domain and leucine rich repeat protein phosphatase
139124	g	o--------	OR5A1	olfactory receptor, family 5, subfamily A, member 1
139142	a	oooooo+++	ZNF232	zinc finger protein 232
139177	a	o++++++++	LOC81691	null
139184	b	ooooooo+o	null	null
139194	a	oooooo+++	TMEM41B	transmembrane protein 41B
139221	b	ooo++oooo	LOC390940	null
139236	h	o-ooooooo	GOSR2	golgi SNAP receptor complex member 2
139247	b	oooo+oooo	null	null
139386	b	ooo++oooo	KIAA0934	KIAA0934
139480	b	ooo++oooo	AGXT2	alanine-glyoxylate aminotransferase 2
139497	a	oooooooo+	LOC284062	null
139521	i	o--oooo--	CHDH	choline dehydrogenase
139550	b	ooooooo+o	DLGAP1	discs, large (Drosophila) homolog-associated protein 1
139601	h	oooooo-oo	psiTPTE22	null
139640	b	ooo+ooooo	FLJ10786	null
139666	b	oooooo++o	MUS81	MUS81 endonuclease homolog (yeast)
139725	b	oooo+oooo	NRXN3	neurexin 3
139736	b	oo++ooooo	RGL2	ral guanine nucleotide dissociation stimulator-like 2
139741	g	ooooooo--	PARVB	parvin, beta
139776	b	oooo+oooo	null	null
139787	b	ooo+ooooo	TFDP1	transcription factor Dp-1
139819	b	oooo+oooo	RAB25	RAB25, member RAS oncogene family
139844	a	oooo+++++	LXN	latexin
139878	b	ooooo+ooo	null	null
139888	i	o-oo-----	RP11-38O23.2	null
139923	a	oooooooo+	SYNE1	spectrin repeat containing, nuclear envelope 1
139930	h	o--oooooo	FLJ20294	null
139956	a	ooo++++++	null	null
139986	b	ooo++oooo	MBNL3	muscleblind-like 3 (Drosophila)
140007	b	ooo+ooooo	HECTD3	HECT domain containing 3
140015	h	oooo----o	FRMD3	FERM domain containing 3
140053	b	oooooo+oo	null	null
140054	b	oo+++++oo	SRPX2	sushi-repeat-containing protein, X-linked 2
140057	b	ooo+ooooo	null	null

140091	b	ooooo+ooo	null	null
140109	h	oooooo--o	null	null
140129	b	ooo+ooooo	RICS	null
140135	a	o++++++++	null	null
140221	b	ooooooo+o	LOC440965	null
140228	b	oooo++++o	C11orf16	chromosome 11 open reading frame 16
140242	b	ooo+ooooo	HKR1	GLI-Kruppel family member HKR1
140257	c	oooooo+o+	NFIC	nuclear factor I/C (CCAAT-binding transcription factor)
140293	b	oooo+++oo	null	null
140314	b	ooo+ooooo	TTC3	tetratricopeptide repeat domain 3
140323	b	ooooooo+o	OR8B12	olfactory receptor, family 8, subfamily B, member 12
140341	b	ooo+ooooo	PANK3	pantothenate kinase 3
140486	h	o-ooooooo	ITLN2	intelectin 2
140531	b	ooo+++ooo	GNAT3	guanine nucleotide binding protein, alpha transducing 3
140632	c	ooo+oo+++	MEF2D	MADS box transcription enhancer factor 2, polypeptide D (myocyte enhancer factor 2D)
140645	i	oo---o---	null	null
140675	c	ooo+o++++	LINS1	lines homolog 1 (Drosophila)
140727	b	ooo+ooooo	USP48	ubiquitin specific peptidase 48
140753	b	oooo++ooo	null	null
140829	a	oo+++++++	null	null
140845	b	ooooooo+o	AER61	null
140995	c	ooo+ooo++	ZNF263	zinc finger protein 263
141012	b	ooo+ooooo	COL18A1	collagen, type XVIII, alpha 1
141059	h	oooo----o	SPSB4	splA/ryanodine receptor domain and SOCS box containing 4
141084	a	oooooooo+	LOC136288	null
141093	g	oooooooo-	DPP7	dipeptidylpeptidase 7
141122	a	oo+++++++	CCBL1	cysteine conjugate-beta lyase; cytoplasmic (glutamine transaminase K, kyneurenine aminotransferase)
141136	a	oooooo+++	ARHGEF2	rho/rac guanine nucleotide exchange factor (GEF) 2
141151	a	oooooooo+	MMP3	matrix metallopeptidase 3 (stromelysin 1, progelatinase)
141190	g	o--------	HOXB13	homeobox B13
141204	a	oooooo+++	SNAI1	snail homolog 1 (Drosophila)
141223	a	oo+++++++	null	null
141227	h	oooo-oooo	CXorf40A|CXorf40B	chromosome X open reading frame 40A|chromosome X open reading frame 40B
141266	b	ooooo+ooo	null	null
141379	a	oooooooo+	LOC392993	null
141392	i	o---oo---	NAPSB	napsin B aspartic peptidase pseudogene
141393	a	oooooo+++	CREM	cAMP responsive element modulator
141460	g	o--------	CAPN3	calpain 3, (p94)
141482	b	ooo+ooooo	ZNF142	zinc finger protein 142 (clone pHZ-49)
141520	b	oooooo+oo	C19orf36	chromosome 19 open reading frame 36
141531	h	o-ooooooo	null	null
141544	a	oo+++++++	MGC2654	null
141605	a	ooooo++++	TIGA1	null
141649	b	ooo+ooooo	ARMC9	armadillo repeat containing 9
141679	a	oo+++++++	TUBG2	tubulin, gamma 2
141723	h	oooo----o	null	null
141785	a	o++++++++	IL11RA	interleukin 11 receptor, alpha
141828	h	o-ooooooo	MYST2	MYST histone acetyltransferase 2
141843	b	ooo+ooooo	PSTPIP2	proline-serine-threonine phosphatase interacting protein 2
141844	c	oo++oooo+	THEG	Theg homolog (mouse)
141854	b	oooooo+oo	CA11	carbonic anhydrase XI
141900	b	ooo+ooooo	FLJ25416	null
141935	b	ooo+ooooo	null	null
141995	b	oooooo+oo	GSDM1	gasdermin 1
142039	a	ooo++++++	PARP6	poly (ADP-ribose) polymerase family, member 6
142051	g	oooooooo-	HSPB1	heat shock 27kDa protein 1
142122	b	ooo+ooooo	PDZK3	PDZ domain containing 3
142146	a	ooo++++++	GAS1	growth arrest-specific 1
142147	b	ooo++oooo	FLJ39501	null
142209	a	oooooo+++	IRF1	interferon regulatory factor 1
142215	b	o+ooooooo	RP3-340N1.3	null
142233	b	ooo+ooooo	SLC6A8	solute carrier family 6 (neurotransmitter transporter, creatine), member 8
142235	b	o+ooooooo	ENO2	enolase 2 (gamma, neuronal)
142237	g	o--------	MMP12	matrix metallopeptidase 12 (macrophage elastase)
142255	g	oooo-----	null	null
142278	b	ooo+ooooo	null	null
142358	i	o-oo-----	KIAA0363|LOC392666	null

142408	h	oooooo-oo	FLJ30934	null
142447	b	ooo+ooooo	MANBA	mannosidase, beta A, lysosomal
142499	j	o-oooo+++	JUB	jub, ajuba homolog (Xenopus laevis)
142534	a	o++++++++	WHSC1	Wolf-Hirschhorn syndrome candidate 1
142563	b	oooooo+oo	MAPK3	mitogen-activated protein kinase 3
142603	a	ooooo++++	SNAI2	snail homolog 2 (Drosophila)
142624	b	oooo++ooo	OR10R3P	olfactory receptor, family 10, subfamily R, member 3 pseudogene
142657	b	oooooo+oo	GDI1	GDP dissociation inhibitor 1
142663	b	oooo+oooo	DLG5	discs, large homolog 5 (Drosophila)
142677	h	o-------o	null	null
142678	a	ooo++++++	RBM4	RNA binding motif protein 4
142719	h	oooo----o	SNCB	synuclein, beta
142752	b	ooooooo+o	SIX3	sine oculis homeobox homolog 3 (Drosophila)
142793	g	ooooooo--	IGLV6-57	immunoglobulin lambda variable 6-57
142827	a	ooo++++++	DUSP8	dual specificity phosphatase 8
142863	b	ooo+ooooo	RCD-8	null
142874	b	ooo++++oo	RHOBTB2	Rho-related BTB domain containing 2
142892	b	ooo+ooooo	KIRREL	kin of IRRE like (Drosophila)
142902	b	ooo+ooooo	BTBD12	BTB (POZ) domain containing 12
142934	b	ooo+ooooo	RAB11FIP2	RAB11 family interacting protein 2 (class I)
142958	a	oooooo+++	MAFG	v-maf musculoaponeurotic fibrosarcoma oncogene homolog G (avian)
142986	i	o-----o--	PKMYT1	protein kinase, membrane associated tyrosine/threonine 1
143028	b	ooooo+ooo	null	null
143036	c	ooo+oo+++	ZNF408	zinc finger protein 408
143047	b	ooo+ooooo	CANT1	calcium activated nucleotidase 1
143083	i	oooo-o---	CARHSP1	calcium regulated heat stable protein 1, 24kDa
143092	a	oo+++++++	LOC389025	null
143102	c	ooo+oo+++	DUSP16	dual specificity phosphatase 16
143103	h	oooooo-oo	RABGGTA	Rab geranylgeranyltransferase, alpha subunit
143111	a	o++++++++	GPT2	glutamic pyruvate transaminase (alanine aminotransferase) 2
143140	a	ooo++++++	PRICKLE1	prickle-like 1 (Drosophila)
143169	b	ooo+ooooo	MAP4K2	mitogen-activated protein kinase kinase kinase kinase 2
143196	a	oooooooo+	null	null
143207	g	ooo------	MRPS6	mitochondrial ribosomal protein S6
143228	b	ooooo+ooo	MTMR6	myotubularin related protein 6
143246	h	o-ooooooo	NVL	nuclear VCP-like
143288	b	oooooo+oo	CSF3	colony stimulating factor 3 (granulocyte)
143297	b	oooo+oooo	KCNE3	potassium voltage-gated channel, Isk-related family, member 3
143310	b	ooo+ooooo	C10orf26	chromosome 10 open reading frame 26
143439	b	o+ooooooo	PUS3	pseudouridylate synthase 3
143456	g	oo-------	FOXP4	forkhead box P4
143495	b	ooo+ooooo	ZNF426	zinc finger protein 426
143539	b	ooo+ooooo	C14orf35	chromosome 14 open reading frame 35
143554	b	ooo+ooooo	TTR	transthyretin (prealbumin, amyloidosis type I)
143560	g	o--------	MAP3K13	mitogen-activated protein kinase kinase kinase 13
143569	i	o-oo-----	ASGR2	asialoglycoprotein receptor 2
143582	b	oooooo+oo	IGHM	immunoglobulin heavy constant mu
143643	b	ooooooo+o	DEFB4	defensin, beta 4
143732	a	oooooooo+	PPP1R1A	protein phosphatase 1, regulatory (inhibitor) subunit 1A
143768	b	ooooooo+o	EFCBP2	EF-hand calcium binding protein 2
143791	h	o-ooooooo	null	null
143828	b	ooo+ooooo	SURF5	surfeit 5
143857	b	o++++oooo	ANKRD47	ankyrin repeat domain 47
143887	i	o--o-----	DAB2IP	DAB2 interacting protein
143892	g	o--------	LOC388938	null
143933	h	o-------o	TMCC3	transmembrane and coiled-coil domain family 3
143980	b	ooo+ooooo	SDC3	syndecan 3 (N-syndecan)
143991	c	ooo+ooo++	ABL2	v-abl Abelson murine leukemia viral oncogene homolog 2 (arg, Abelson-related gene)
143996	b	oooo+oooo	PSCDBP	pleckstrin homology, Sec7 and coiled-coil domains, binding protein
143997	a	oo+++++++	SGNE1	secretory granule, neuroendocrine protein 1 (7B2 protein)
144005	a	oooooo+++	DUSP2	dual specificity phosphatase 2
144020	a	oo+++++++	TUG1	null
144028	a	ooo++++++	CIDEC	cell death-inducing DFFA-like effector c
144041	g	ooooooo--	C14orf173	chromosome 14 open reading frame 173
144049	a	oo+++++++	BTG1	B-cell translocation gene 1, anti-proliferative
144113	a	oooooo+++	FOSB	FBJ murine osteosarcoma viral oncogene homolog B
144134	b	ooo++oooo	FTHL17	ferritin, heavy polypeptide-like 17
144138	b	ooooooo+o	VIP	vasoactive intestinal peptide
144148	g	ooooooo--	PDCD4	programmed cell death 4 (neoplastic transformation inhibitor)
144160	c	oo+ooooo+	PAFAH2	platelet-activating factor acetylhydrolase 2, 40kDa

144171	g	o--------	STXBP3	syntaxin binding protein 3
144172	g	o--------	CXCL9	chemokine (C-X-C motif) ligand 9
144182	g	oooooooo-	CST3	cystatin C (amyloid angiopathy and cerebral hemorrhage)
144226	b	oooo+oooo	FLJ14466	null
144241	h	oooo--ooo	GNPTG	N-acetylglucosamine-1-phosphate transferase, gamma subunit
144248	f	oo++----+	CRTAC1	cartilage acidic protein 1
144258	i	o-oo-----	MGC52057	null
144265	a	oooooooo+	HIST1H2AL	histone 1, H2al
144369	i	o---o----	ITCH	itchy homolog E3 ubiquitin protein ligase (mouse)
144379	b	ooo+ooooo	FBXO18	F-box protein, helicase, 18
144454	h	o-ooooooo	HBZ	hemoglobin, zeta
144477	g	oooo-----	HSA272196	null
144503	b	ooo++oooo	MAPK8IP3	mitogen-activated protein kinase 8 interacting protein 3
144537	a	ooo++++++	INOC1	INO80 complex homolog 1 (S. cerevisiae)
144569	g	oo-------	MT1F	metallothionein 1F (functional)
144600	g	o--------	TFPI2	tissue factor pathway inhibitor 2
144601	b	oooo+oooo	DDAH1	dimethylarginine dimethylaminohydrolase 1
144620	b	ooooooo+o	LOC152195	null
144625	b	oooo+oooo	null	null
144657	a	ooooo++++	null	null
144733	b	ooo+ooooo	LOC374569	null
144755	b	ooo+ooooo	null	null
144788	b	oooo+oooo	LOC401074	null
144826	b	ooo+ooooo	ZNF19|ZNF23	zinc finger protein 19 (KOX 12)|zinc finger protein 23 (KOX 16)
144829	b	oooooo+oo	TF	transferrin
144833	i	o--o-----	CABC1	chaperone, ABC1 activity of bc1 complex like (S. pombe)
144834	a	oooooo+++	HIST2H3C|H3/o	histone 2, H3c
144901	g	o--------	SLITL2	slit-like 2 (Drosophila)
144903	b	ooooo+ooo	SLC16A14	solute carrier family 16 (monocarboxylic acid transporters), member 14
144979	g	oooooooo-	CDCA7L	cell division cycle associated 7-like
144990	b	ooo+ooooo	null	null
145018	b	oooo+oooo	SHE	Src homology 2 domain containing E
145029	g	o--------	DKFZp434J0226	null
145045	d	ooo+oooo-	PREX1	null
145069	a	o++++++++	BIRC2	baculoviral IAP repeat-containing 2
145078	i	o------o-	STARD8	START domain containing 8
145100	b	ooooo+ooo	CACNG2	calcium channel, voltage-dependent, gamma subunit 2
145130	h	ooooo-ooo	LOC442245	null
145150	g	o--------	MESDC1	mesoderm development candidate 1
145154	c	oo+++++o+	ZNF292	zinc finger protein 292
145225	a	ooo++++++	SLC30A7	solute carrier family 30 (zinc transporter), member 7
145241	a	oooooooo+	HTR2A	5-hydroxytryptamine (serotonin) receptor 2A
145259	b	oooo+oooo	null	null
145266	b	ooo+ooooo	IQCC	IQ motif containing C
145287	b	ooo+ooooo	ORC1L	origin recognition complex, subunit 1-like (yeast)
145379	b	ooo+ooooo	TBC1D5	TBC1 domain family, member 5
145456	g	o--------	null	null
145487	b	ooo+ooooo	GPAM	glycerol-3-phosphate acyltransferase, mitochondrial
145498	a	ooo++++++	CXorf45	chromosome X open reading frame 45
145500	i	oo----o--	C10orf79	chromosome 10 open reading frame 79
145563	b	ooo+ooooo	EIF2AK4	eukaryotic translation initiation factor 2 alpha kinase 4
145581	b	oooooo+oo	ALG3	asparagine-linked glycosylation 3 homolog (yeast, alpha-1,3-mannosyltransferase)
145596	b	oooo+oooo	KIAA1205	KIAA1205
145646	a	oooooooo+	DKFZp761H2121	null
145663	h	ooooooo-o	null	null
145693	a	oooooo+++	DIO3	deiodinase, iodothyronine, type III
145726	g	oooooooo-	null	null
145753	h	oooo-oooo	PPAN	peter pan homolog (Drosophila)
145761	a	ooo++++++	FLJ20436	null
145797	g	oooooooo-	C19orf24	chromosome 19 open reading frame 24
145871	g	oooooooo-	CCND3	cyclin D3
145893	b	ooo+ooooo	UBE3B	ubiquitin protein ligase E3B
145914	b	ooo+ooooo	NUDT12	nudix (nucleoside diphosphate linked moiety X)-type motif 12
145937	b	oooooo+oo	LOC400451	null
145947	a	oo+++++++	ADCK2	aarF domain containing kinase 2
145959	i	o-oo-----	ST6GAL1	ST6 beta-galactosamide alpha-2,6-sialyltranferase 1
145983	b	ooo+ooooo	TACC1	transforming, acidic coiled-coil containing protein 1
145991	a	ooo++++++	ZNF3	zinc finger protein 3 (A8-51)
145997	g	ooo------	null	null
146015	b	ooooo++oo	null	null

146058	h	oooooo-oo	MMP2	matrix metallopeptidase 2 (gelatinase A, 72kDa gelatinase, 72kDa type IV collagenase)
146070	b	ooo+ooooo	PRO2964	null
146072	b	oooo+oooo	null	null
146115	b	oo+++oooo	LNPEP	leucyl/cystinyl aminopeptidase
146127	b	oooo+oooo	C1orf68	chromosome 1 open reading frame 68
146130	b	oooooo+oo	TINF2	TERF1 (TRF1)-interacting nuclear factor 2
146136	f	oo++----+	ULK4	unc-51-like kinase 4 (C. elegans)
146228	b	oooo++ooo	null	null
146234	b	oooo+oooo	EHMT2	euchromatic histone-lysine N-methyltransferase 2
146364	c	oo++oooo+	SLC14A2	solute carrier family 14 (urea transporter), member 2
146368	g	ooo------	MT2A	metallothionein 2A
146370	a	ooooooo++	NPPC	natriuretic peptide precursor C
146435	g	oooooooo-	HSPBP1	null
146438	b	ooooooo+o	INADL	InaD-like (Drosophila)
146508	b	ooo+ooooo	SMYD5	SMYD family member 5
146512	g	oooo-----	PARP4	poly (ADP-ribose) polymerase family, member 4
146533	b	o+ooooooo	YPEL2	yippee-like 2 (Drosophila)
146543	b	ooooo+ooo	ROBO4	roundabout homolog 4, magic roundabout (Drosophila)
146548	g	o--------	SMARCB1	SWI/SNF related, matrix associated, actin dependent regulator of chromatin, subfamily b, member 1
146556	b	ooo+ooooo	KIAA1862	null
146563	g	o--------	null	null
146592	g	o--------	null	null
146661	b	ooo+ooooo	ADD1	adducin 1 (alpha)
146663	b	ooo+ooooo	ANKRD15	ankyrin repeat domain 15
146688	b	oooo+oooo	null	null
146709	h	o-ooooooo	FLJ10808	null
146753	a	oooooo+++	C11orf46	chromosome 11 open reading frame 46
146761	a	oo+++++++	MGC14376	null
146786	b	ooo+ooooo	TU12B1-TY	null
146841	b	ooo+ooooo	KBTBD4	kelch repeat and BTB (POZ) domain containing 4
146843	a	oooooo+++	GSTM3	glutathione S-transferase M3 (brain)
146885	b	ooo+ooooo	POMT2	protein-O-mannosyltransferase 2
146893	b	ooo+ooooo	GPD1L	glycerol-3-phosphate dehydrogenase 1-like
146894	a	oooooo+++	RIT1	Ras-like without CAAX 1
147011	h	o-ooooooo	TTC17	tetratricopeptide repeat domain 17
147079	g	oooooooo-	TCIRG1	T-cell, immune regulator 1, ATPase, H+ transporting, lysosomal V0 protein a isoform 3
147106	a	o++++++++	PDRG1	p53 and DNA damage regulated 1
147139	a	ooo++++++	MCL1	myeloid cell leukemia sequence 1 (BCL2-related)
147144	i	oooo--o--	CSGlcA-T	null
147154	b	ooooo+ooo	CCDC19	coiled-coil domain containing 19
147222	h	ooooo-ooo	FLJ40126	null
147300	b	ooo+ooooo	CSNK1G3	casein kinase 1, gamma 3
147307	a	ooooooo++	pp9099	null
147309	b	oooo++ooo	COL22A1	collagen, type XXII, alpha 1
147327	b	oooo+oooo	TNFRSF25	tumor necrosis factor receptor superfamily, member 25
147339	b	ooo+ooooo	SHQ1	SHQ1 homolog (S. cerevisiae)
147353	a	oooooo+++	EGR1	early growth response 1
147379	b	ooo+ooooo	TTF2	transcription termination factor, RNA polymerase II
147385	a	o++++++++	C21orf119	chromosome 21 open reading frame 119
147395	b	oooo+oooo	BZRAP1	benzodiazapine receptor (peripheral) associated protein 1
147403	b	oooooo+oo	ZNF583	zinc finger protein 583
147436	b	oo+oooooo	MAP2	microtubule-associated protein 2
147486	a	ooo++++++	ZNF34	zinc finger protein 34 (KOX 32)
147491	g	oooooooo-	PIGS	phosphatidylinositol glycan, class S
147522	b	oooo+oooo	TBXA2R	thromboxane A2 receptor
147526	c	oo++oooo+	RBM24	RNA binding motif protein 24
147539	e	ooo+-oooo	PPARGC1A	peroxisome proliferative activated receptor, gamma, coactivator 1, alpha
147570	b	ooo+ooooo	null	null
147578	g	ooo------	CLIC2	chloride intracellular channel 2
147603	h	oooo--ooo	null	null
147612	d	ooo+o----	EXOC6	exocyst complex component 6
147628	b	oooooo++o	ESR1	estrogen receptor 1
147646	g	o--------	IMP-2	null
147679	b	ooo+++++o	NPC1L1	NPC1 (Niemann-Pick disease, type C1, gene)-like 1
147730	b	ooo+ooooo	RBM19	RNA binding motif protein 19
147749	a	ooooooo++	CRP	C-reactive protein, pentraxin-related
147764	h	o-ooooooo	OGG1	8-oxoguanine DNA glycosylase
147767	b	ooo+ooooo	VARSL	valyl-tRNA synthetase like

147809	b	ooo+ooooo	ANKRD33	ankyrin repeat domain 33
147822	a	oooooo+++	LATS2	LATS, large tumor suppressor, homolog 2 (Drosophila)
147852	h	o--oooooo	null	null
147920	a	oo+++++++	CPEB1	cytoplasmic polyadenylation element binding protein 1
147930	a	oooooo+++	MKNK2	MAP kinase interacting serine/threonine kinase 2
147942	h	oooo----o	EDG6	endothelial differentiation, G-protein-coupled receptor 6
147962	b	ooooo+ooo	LACE1	lactation elevated 1
147978	a	ooo++++++	FAM84B	family with sequence similarity 84, member B
148004	b	oooo+oooo	null	null
148114	a	o++++++++	TRIT1	tRNA isopentenyltransferase 1
148153	a	ooo++++++	DUSP4	dual specificity phosphatase 4
148166	b	ooo+ooooo	CDCA2	cell division cycle associated 2
148170	g	oooo-----	null	null
148186	b	ooo+ooooo	STMN3	stathmin-like 3
148252	b	oooooo+oo	TFAP2A	transcription factor AP-2 alpha (activating enhancer binding protein 2 alpha)
148303	b	ooo+ooooo	DHX30	DEAH (Asp-Glu-Ala-His) box polypeptide 30
148325	b	ooo+ooooo	SLC7A9	solute carrier family 7 (cationic amino acid transporter, y+ system), member 9
148356	b	ooo+ooooo	SH3MD2	SH3 multiple domains 2
148396	a	ooooo++++	JOSD3	Josephin domain containing 3
148466	h	oooo---oo	FLJ46072	null
148473	i	oo-oo----	PTPN22	protein tyrosine phosphatase, non-receptor type 22 (lymphoid)
148475	c	ooo+ooo++	null	null
148511	b	oooooo+oo	SFN	stratifin
148534	b	ooo+ooooo	KIAA1327	null
148600	b	oooooo+oo	EGLN1	egl nine homolog 1 (C. elegans)
148604	b	ooo+ooooo	TSC2	tuberous sclerosis 2
148619	g	oooo-----	ARHGEF10	Rho guanine nucleotide exchange factor (GEF) 10
148652	a	oooooooo+	LOC342931	null
148656	b	ooo+ooooo	null	null
148661	b	oooo+oooo	DKFZp434O0320	null
148692	i	o-oo-----	KIAA1026	null
148696	a	oooooo+++	JUNB	jun B proto-oncogene
148704	a	ooooooo++	HBA1|HBA2	hemoglobin, alpha 1|hemoglobin, alpha 2
148728	h	oooooo--o	C6orf25	chromosome 6 open reading frame 25
148730	b	oooooo+oo	REM1	RAS (RAD and GEM)-like GTP-binding 1
148731	b	oooooo+oo	KIAA1324	KIAA1324
148741	g	o--------	DNASE1L2	deoxyribonuclease I-like 2
148761	b	ooooooo+o	BAPX1	bagpipe homeobox homolog 1 (Drosophila)
148769	g	ooooooo--	VAT1	vesicle amine transport protein 1 homolog (T californica)
148771	b	ooooooo+o	RENBP	renin binding protein
148806	g	o--------	SRCAP	null
148861	b	oo++ooooo	LOC283901	null
148869	b	oooooo+oo	ATAD3C	ATPase family, AAA domain containing 3C
148882	b	ooo++oooo	TRRAP	transformation/transcription domain-associated protein
148950	a	o++++++++	SCARF2	scavenger receptor class F, member 2
148967	g	o--------	null	null
148987	b	ooooo+++o	GDF5	growth differentiation factor 5 (cartilage-derived morphogenetic protein-1)
149014	i	ooooo-oo-	FLYWCH1	FLYWCH-type zinc finger 1
149065	h	oooo-oooo	FLJ10661	null
149134	g	o--------	LMNB2	lamin B2
149158	g	oooooo---	RRAD	Ras-related associated with diabetes
149212	g	ooo------	LSM6	LSM6 homolog, U6 small nuclear RNA associated (S. cerevisiae)
149236	g	o--------	POLR1C	polymerase (RNA) I polypeptide C, 30kDa
149252	b	oooo+oooo	ZNFN1A3	zinc finger protein, subfamily 1A, 3 (Aiolos)
149253	a	oooooo+++	PAMCI	peptidylglycine alpha-amidating monooxygenase COOH-terminal interactor
149260	i	o-oo-----	PLA2G6	phospholipase A2, group VI (cytosolic, calcium-independent)
149270	c	ooo+o++++	JMJD2D	jumonji domain containing 2D
149292	c	ooo+o++++	C1orf155	chromosome 1 open reading frame 155
149340	b	ooo+ooooo	PKN1	protein kinase N1
149347	c	ooo++oo++	PDIK1L	PDLIM1 interacting kinase 1 like
149353	b	ooo+ooooo	DEPDC5	DEP domain containing 5
149354	a	oooooooo+	SPAG4L	sperm associated antigen 4-like
149363	b	ooooo+ooo	null	null
149434	b	ooo+ooooo	STXBP1	syntaxin binding protein 1
149469	g	o--------	OPN4	opsin 4 (melanopsin)
149553	j	oooo----+	PPP1R9B	protein phosphatase 1, regulatory subunit 9B, spinophilin
149581	a	oo+++++++	SLC22A1	solute carrier family 22 (organic cation transporter), member 1
149591	j	o---oo+++	RHOB	ras homolog gene family, member B
149612	b	ooooo+ooo	HIST1H1E	histone 1, H1e
149647	a	ooooo++++	HIST2H2AA|HIST2H2AC	histone 2, H2aa|histone 2, H2ac

149749	f	oo++----+	null	null
149758	g	oooooooo-	SSBP4	single stranded DNA binding protein 4
149793	b	ooo+ooooo	TPST2	tyrosylprotein sulfotransferase 2
149837	b	ooo+ooooo	MYBBP1A	MYB binding protein (P160) 1a
149868	g	oooooooo-	C6orf148	chromosome 6 open reading frame 148
149874	b	ooo++oooo	ADAMTS6	ADAM metallopeptidase with thrombospondin type 1 motif, 6
149907	b	oooo+oooo	null	null
149984	a	ooo++++++	ZBED1	zinc finger, BED-type containing 1
149997	c	oo++oooo+	NXF3	nuclear RNA export factor 3
150036	g	o--------	GRK5	G protein-coupled receptor kinase 5
150053	a	oooooooo+	KIAA1161	KIAA1161
150074	d	ooo+ooo--	CAMKK1	calcium/calmodulin-dependent protein kinase kinase 1, alpha
150094	c	ooo+oo+++	YTHDF3	YTH domain family, member 3
150159	a	oo+++++++	CDKN2D	cyclin-dependent kinase inhibitor 2D (p19, inhibits CDK4)
150184	i	o--ooooo-	C20orf28	chromosome 20 open reading frame 28
150185	b	ooo+++++o	LOC388022	null
150191	b	ooo+ooooo	PTPN9	protein tyrosine phosphatase, non-receptor type 9
150301	b	ooooo+ooo	SRCRB4D	scavenger receptor cysteine rich domain containing, group B (4 domains)
150432	i	o-----o--	FAM100A	family with sequence similarity 100, member A
150467	b	ooooooo+o	null	null
150534	a	o++++++++	TOMM40L	translocase of outer mitochondrial membrane 40 homolog-like (yeast)
150535	b	ooooo+ooo	C10orf63	chromosome 10 open reading frame 63
150576	d	ooo+ooo--	NSF	N-ethylmaleimide-sensitive factor
150578	h	oooooo--o	PKD2L1	polycystic kidney disease 2-like 1
150591	h	o-ooooooo	OFOXD	oxoglutarate and iron-dependent oxygenase domain containing
150667	b	ooo+ooooo	MAGED1	melanoma antigen family D, 1
150697	b	ooo++++oo	DNMBP	dynamin binding protein
150708	g	oooooooo-	MRPS36	mitochondrial ribosomal protein S36
150739	b	oooo+oooo	null	null
150749	a	ooo++++++	TRIB1	tribbles homolog 1 (Drosophila)
150758	b	ooooooo+o	MGC4093	null
150785	a	ooooooo++	PRKACG	protein kinase, cAMP-dependent, catalytic, gamma
150804	b	oooo+oooo	null	null
150820	b	oo+oooooo	OR7A5	olfactory receptor, family 7, subfamily A, member 5
150849	h	oooo----o	IFNA14	interferon, alpha 14
150864	b	ooooooo+o	null	null
150870	a	oo+++++++	null	null
150889	b	oooooo+oo	PTPRQ	protein tyrosine phosphatase, receptor type, Q
150924	b	ooooo+ooo	LY86	lymphocyte antigen 86
150965	h	o-ooooooo	POMGNT1	protein O-linked mannose beta1,2-N-acetylglucosaminyltransferase
150999	b	ooooooo+o	OR8J1	olfactory receptor, family 8, subfamily J, member 1
151000	a	oooooo+++	CEBPD	CCAAT/enhancer binding protein (C/EBP), delta
151014	a	ooo++++++	NUDT13	nudix (nucleoside diphosphate linked moiety X)-type motif 13
151042	a	o++++++++	CHRNA10	cholinergic receptor, nicotinic, alpha polypeptide 10
151049	a	o++++++++	MLLT11	myeloid/lymphoid or mixed-lineage leukemia (trithorax homolog, Drosophila); translocated to, 11
151050	b	ooooo+ooo	OR5BF1	olfactory receptor, family 5, subfamily BF, member 1
151060	b	ooo++oooo	FAM40A	family with sequence similarity 40, member A
151071	a	o++++++++	ZNF406	zinc finger protein 406
151078	b	ooooooo+o	TNFAIP8L3	tumor necrosis factor, alpha-induced protein 8-like 3
151095	b	oooooo+oo	SLITRK3	SLIT and NTRK-like family, member 3
151107	g	ooooo----	LRBA	LPS-responsive vesicle trafficking, beach and anchor containing
151115	b	ooooo+++o	null	null
151128	b	ooo+ooooo	STAU2	staufen, RNA binding protein, homolog 2 (Drosophila)
151138	h	o-ooooooo	FZD5	frizzled homolog 5 (Drosophila)
151155	a	ooooooo++	PRND	prion protein 2 (dublet)
151175	h	o--oooooo	null	null
151187	h	oooo-oooo	TAS1R1	taste receptor, type 1, member 1
151189	b	oooo+oooo	FEM1A	fem-1 homolog a (C.elegans)
151218	g	o--------	MGC26647	null
151262	b	oooooo+oo	MGC10334	null
151294	b	ooooo+ooo	HHAT	hedgehog acyltransferase
151357	b	ooo+ooooo	LOC200731	null
151377	k	oo-+ooooo	RNF103	ring finger protein 103
151390	b	oooo+oooo	PRKG1	protein kinase, cGMP-dependent, type I
151402	b	oooooo+oo	PPM1M	protein phosphatase 1M (PP2C domain containing)
151439	b	ooo+ooooo	LIMK2	LIM domain kinase 2
151469	b	ooooooo+o	GPR114	G protein-coupled receptor 114
151476	a	oo+++++++	TAZ	tafazzin (cardiomyopathy, dilated 3A (X-linked); endocardial fibroelastosis 2; Barth syndrome)
151529	a	ooo++++++	ZC3H10	zinc finger CCCH-type containing 10

151531	a	o++++++++	DBN1	drebrin 1
151544	b	ooooo+ooo	TMPRSS7	transmembrane protease, serine 7
151559	b	ooooooo+o	null	null
151575	h	oooo----o	FLJ36748	null
151616	b	ooooo+ooo	null	null
151711	h	oooo----o	null	null
151724	c	ooo+o++++	BCL6	B-cell CLL/lymphoma 6 (zinc finger protein 51)
151768	c	oo++oooo+	C18orf45	chromosome 18 open reading frame 45
151818	b	oo+++++oo	null	null
151827	h	o--oooooo	KIAA1754	KIAA1754
151841	g	o--------	PIP5KL1	phosphatidylinositol-4-phosphate 5-kinase-like 1
151858	a	ooo++++++	RBM21	RNA binding motif protein 21
151912	b	oooooo+oo	LMTK2	lemur tyrosine kinase 2
151914	b	ooo+ooooo	RPS6KC1	ribosomal protein S6 kinase, 52kDa, polypeptide 1
151941	b	ooo+ooooo	TRIM68	tripartite motif-containing 68
151956	a	oooooo+++	AXUD1	AXIN1 up-regulated 1
152007	a	ooo++++++	ZNF271	zinc finger protein 271
152058	a	ooooooo++	MKNK2	MAP kinase interacting serine/threonine kinase 2
152070	g	oooo-----	GPR64	G protein-coupled receptor 64
152075	b	oooooo+oo	NAPA	N-ethylmaleimide-sensitive factor attachment protein, alpha
152134	g	oooooo---	ATHL1	ATH1, acid trehalase-like 1 (yeast)
152152	g	ooooo----	PRG4	proteoglycan 4
152158	h	oooo----o	EGFL7	EGF-like-domain, multiple 7
152163	b	oo++ooooo	CASP12P1	caspase 12 pseudogene 1
152202	a	o++++++++	C7orf26	chromosome 7 open reading frame 26
152213	b	oooo+oooo	FLJ10415	null
152231	f	oo++----+	null	null
152243	g	oooooooo-	MGC2747	null
152283	b	ooo+ooooo	MCTP2	multiple C2 domains, transmembrane 2
152363	b	ooo+ooooo	RPS6KA5	ribosomal protein S6 kinase, 90kDa, polypeptide 5
152376	g	ooo------	EMP3	epithelial membrane protein 3
152432	h	oooo----o	LOC343221	null
152463	b	ooo+ooooo	PLCL2	phospholipase C-like 2
152507	b	oooooo+oo	NOTCH4	Notch homolog 4 (Drosophila)
152539	h	o-ooooooo	PSEN1	presenilin 1 (Alzheimer disease 3)
152542	f	oo++----+	null	null
152569	b	ooo+ooooo	RBED1	RNA binding motif and ELMO domain 1
152620	b	ooo+ooooo	C21orf58	chromosome 21 open reading frame 58
152627	b	ooo+ooooo	PYGB	phosphorylase, glycogen; brain
152650	h	ooooooo-o	ANKRD13D	ankyrin repeat domain 13 family, member D
152660	g	o--------	CD6	CD6 antigen
152725	b	ooo+ooooo	PLAT	plasminogen activator, tissue
152726	b	ooo+ooooo	LOC400969	null
152735	b	ooooooo+o	MPP2	membrane protein, palmitoylated 2 (MAGUK p55 subfamily member 2)
152779	b	ooo++oooo	IRS2	insulin receptor substrate 2
152864	b	ooo+ooooo	KIAA0804	KIAA0804
152867	h	o-------o	FLJ14351	null
152911	b	ooo+ooooo	FAM40B	family with sequence similarity 40, member B
152949	j	oooo----+	B3GNT4	UDP-GlcNAc:betaGal beta-1,3-N-acetylglucosaminyltransferase 4
153147	b	ooo+ooooo	KIAA0556	null
153155	b	oooooo+oo	LOC144097	null
153167	i	o-----o--	MID1IP1	MID1 interacting protein 1 (gastrulation specific G12-like (zebrafish))
153177	f	oo++----+	null	null
153182	b	ooo+ooooo	CNTNAP3|CNTNAP3B	contactin associated protein-like 3|contactin associated protein-like 3B
153207	g	o--------	LRFN3	leucine rich repeat and fibronectin type III domain containing 3
153215	h	oooo----o	LRRC29	leucine rich repeat containing 29
153324	c	oooo+oo++	null	null
153383	b	o+ooooooo	CPNE4	copine IV
153388	b	oooo+oooo	TNRC6B	trinucleotide repeat containing 6B
153470	b	ooo+ooooo	TMEM76	transmembrane protein 76
153472	g	o--------	MYBPH	myosin binding protein H
153495	g	oooo-----	null	null
153526	b	oooo+oooo	null	null
153533	h	o-ooooooo	C1orf156	chromosome 1 open reading frame 156
153543	b	ooo++++oo	PLEKHA6	pleckstrin homology domain containing, family A member 6
153544	b	oooooo+oo	NAPA	N-ethylmaleimide-sensitive factor attachment protein, alpha
153619	b	ooooooo+o	SLC5A9	solute carrier family 5 (sodium/glucose cotransporter), member 9
153789	b	o+ooooooo	DNMT3A	DNA (cytosine-5-)-methyltransferase 3 alpha
153845	g	o--------	MTMR10	myotubularin related protein 10
153852	b	ooo+ooooo	C18orf17	chromosome 18 open reading frame 17

153870	g	oooooooo-	RAB38	RAB38, member RAS oncogene family
153898	b	ooo+ooooo	C18orf17	chromosome 18 open reading frame 17
153942	g	oooooooo-	PIGQ	phosphatidylinositol glycan, class Q
153986	a	oooooo+++	TRAJ17	T cell receptor alpha joining 17
154045	b	ooo+ooooo	ARHGAP19	Rho GTPase activating protein 19
154089	b	ooooooo+o	MUC13	mucin 13, epithelial transmembrane
154100	b	ooo+ooooo	SPATA2	spermatogenesis associated 2
154122	b	ooooooo+o	null	null
154143	h	ooooo-ooo	MGC23985	null
154165	b	ooo+ooooo	DNAPTP6	null
154168	h	o-ooooooo	KIAA1245|NBPF11|NBPF10|NBPF15|NBPF8	KIAA1245|neuroblastoma breakpoint family, member 11|neuroblastoma breakpoint family, member 10|neuroblastoma breakpoint family, member 15|neuroblastoma breakpoint family, member 8
154223	b	oo++ooooo	GBE1	glucan (1,4-alpha-), branching enzyme 1 (glycogen branching enzyme, Andersen disease, glycogen storage disease type IV)
154286	b	oooooo+oo	FASN	fatty acid synthase
154357	h	o-ooooooo	ZNF133	zinc finger protein 133 (clone pHZ-13)
154359	b	oo++ooooo	C14orf145	chromosome 14 open reading frame 145
154377	l	o----+---	CST8	cystatin 8 (cystatin-related epididymal specific)
154387	b	oooo+oooo	MLLT6	myeloid/lymphoid or mixed-lineage leukemia (trithorax homolog, Drosophila); translocated to, 6
154392	b	oooo+oooo	null	null
154399	b	ooo+ooooo	ABR	active BCR-related gene
154418	a	oo+++++++	null	null
154434	a	ooo++++++	AMIGO2	adhesion molecule with Ig-like domain 2
154437	a	ooo++++++	LRP6	low density lipoprotein receptor-related protein 6
154443	b	oooo+oooo	GUCA1C	guanylate cyclase activator 1C
154444	i	oo-o-----	JAK3	Janus kinase 3 (a protein tyrosine kinase, leukocyte)
154506	b	oooooo+oo	MYH9	myosin, heavy polypeptide 9, non-muscle
154532	a	ooooooo++	null	null
154663	b	ooo+ooooo	BIRC3	baculoviral IAP repeat-containing 3
154675	b	oooo+++oo	null	null
154689	b	oo+oooooo	OR4E2	olfactory receptor, family 4, subfamily E, member 2
154691	b	ooo++oooo	CLSPN	claspin homolog (Xenopus laevis)
154759	b	ooo+ooooo	LRP5	low density lipoprotein receptor-related protein 5
154827	a	o++++++++	FDFT1	farnesyl-diphosphate farnesyltransferase 1
154858	a	oo+++++++	MLL5	myeloid/lymphoid or mixed-lineage leukemia 5 (trithorax homolog, Drosophila)
154879	b	ooo+ooooo	null	null
154897	g	oo-------	SLC2A4RG	SLC2A4 regulator
154907	a	ooo++++++	CTGF	connective tissue growth factor
154909	b	oooooo+oo	AGPAT7	1-acylglycerol-3-phosphate O-acyltransferase 7 (lysophosphatidic acid acyltransferase, eta)
154915	a	ooo++++++	DCTN5	dynactin 5 (p25)
154981	b	ooooooo+o	KIAA0802	KIAA0802
155094	b	ooooooo+o	QRICH2	glutamine rich 2
155100	g	oooooooo-	MAN2B1	mannosidase, alpha, class 2B, member 1
155110	b	ooo+ooooo	MGC9712	null
155145	b	ooo+ooooo	VPS33B	vacuolar protein sorting 33B (yeast)
155160	a	ooo++++++	CALB1	calbindin 1, 28kDa
155197	g	oo-------	MYRIP	myosin VIIA and Rab interacting protein
155212	b	ooo+ooooo	ICAM2	intercellular adhesion molecule 2
155218	h	o--oooooo	SUV420H1	suppressor of variegation 4-20 homolog 1 (Drosophila)
155235	b	ooo++oooo	RBAF600	null
155313	b	ooo+ooooo	PRSS22	protease, serine, 22
155330	h	o-------o	LOC388882	null
155373	b	ooo+ooooo	AKAP7	A kinase (PRKA) anchor protein 7
155386	b	ooo+ooooo	null	null
155387	g	oo-------	TNFRSF8	tumor necrosis factor receptor superfamily, member 8
155423	h	oooooo-oo	P2RY11	purinergic receptor P2Y, G-protein coupled, 11
155495	a	ooo++++++	ASB7	ankyrin repeat and SOCS box-containing 7
155553	g	ooooo----	PPP1R2	protein phosphatase 1, regulatory (inhibitor) subunit 2
155604	b	ooooo+ooo	ADAMTS18	ADAM metallopeptidase with thrombospondin type 1 motif, 18
155654	g	o--------	EYA3	eyes absent homolog 3 (Drosophila)
155682	f	oo++----+	DENND1C	DENN/MADD domain containing 1C
155703	b	ooo+ooooo	LOC389087	null
155712	b	oooo+oooo	SHC3	SHC (Src homology 2 domain containing) transforming protein 3
155718	a	ooo++++++	SH3MD1	SH3 multiple domains 1
155785	b	ooo+ooooo	SH3BP4	SH3-domain binding protein 4
155797	g	o--------	MGC88387	null
155879	h	oooo-oooo	PXMP4	peroxisomal membrane protein 4, 24kDa
155892	b	ooo+ooooo	DDEF1	development and differentiation enhancing factor 1

155893	b	ooo+ooooo	ABCB9	ATP-binding cassette, sub-family B (MDR/TAP), member 9
155928	f	oo++----+	C20orf95	chromosome 20 open reading frame 95
155932	b	oooo++++o	null	null
155957	a	oo+++++++	NR1H3	nuclear receptor subfamily 1, group H, member 3
155970	g	ooooo----	LOC201895	null
155974	b	ooooooo+o	PPY2	pancreatic polypeptide 2
156011	b	ooo+ooooo	RP11-169K16.3	null
156048	h	ooooooo-o	null	null
156141	a	oooooo+++	LOC148203	null
156149	f	oo++----+	GCK	glucokinase (hexokinase 4, maturity onset diabetes of the young 2)
156161	a	oo+++++++	MUC17	mucin 17
156170	g	o--------	RYR1	ryanodine receptor 1 (skeletal)
156192	b	ooo+ooooo	FARS2	phenylalanine-tRNA synthetase 2 (mitochondrial)
156236	a	ooooooo++	KIAA0409	KIAA0409
156377	g	o--------	TREX1	three prime repair exonuclease 1
156511	a	o++++++++	ME1	malic enzyme 1, NADP(+)-dependent, cytosolic
156527	a	oooooo+++	XBP1	X-box binding protein 1
156539	b	oo++ooooo	OSBPL3	oxysterol binding protein-like 3
156570	b	ooo+ooooo	PPAT	phosphoribosyl pyrophosphate amidotransferase
156579	b	oooo+oooo	LOXL1	lysyl oxidase-like 1
156607	a	ooooooo++	SLC19A3	solute carrier family 19, member 3
156624	b	ooo+ooooo	C2orf23	chromosome 2 open reading frame 23
156653	h	o-----ooo	HNRPL	heterogeneous nuclear ribonucleoprotein L
156730	a	ooo++++++	RHEBL1	Ras homolog enriched in brain like 1
156763	a	oo+++++++	STAG3	stromal antigen 3
156829	g	ooooooo--	ACYP2	acylphosphatase 2, muscle type
156837	a	ooo++++++	MGC4268	null
156864	a	ooo++++++	KLF5	Kruppel-like factor 5 (intestinal)
156869	a	ooo++++++	DYRK1A	dual-specificity tyrosine-(Y)-phosphorylation regulated kinase 1A
156901	b	ooo+ooooo	ALS2	amyotrophic lateral sclerosis 2 (juvenile)
156935	b	ooo++oooo	null	null
156938	b	ooo+ooooo	SETBP1	SET binding protein 1
156986	a	o++++++++	PIGA	phosphatidylinositol glycan, class A (paroxysmal nocturnal hemoglobinuria)
157008	g	o--------	ABHD14B	abhydrolase domain containing 14B
157015	b	ooo+ooooo	LOC391599	null
157016	b	ooo+ooooo	OR52B4	olfactory receptor, family 52, subfamily B, member 4
157027	b	ooooooo+o	PLA1A	phospholipase A1 member A
157060	g	o--------	SERTAD4	SERTA domain containing 4
157132	b	ooo+ooooo	null	null
157174	c	ooo+oo+++	TBC1D3|TBC1D3C|TBC1D3B	TBC1 domain family, member 3|TBC1 domain family, member 3C|TBC1 domain family, member 3B
157195	b	oooooo+oo	KRT1	keratin 1 (epidermolytic hyperkeratosis)
157225	b	ooo+ooooo	PCNT	pericentrin (kendrin)
157242	g	ooo------	UXT	ubiquitously-expressed transcript
157246	a	oooooo+++	NAV1	neuron navigator 1
157279	k	oo-+ooooo	ZNF14	zinc finger protein 14 (KOX 6)
157311	b	ooo+ooooo	KIAA0467	KIAA0467
157335	g	oo-------	null	null
157336	b	ooo+ooooo	NHLRC2	NHL repeat containing 2
157342	j	o----oo++	C1orf63	chromosome 1 open reading frame 63
157389	b	ooo+ooooo	TJP2	tight junction protein 2 (zona occludens 2)
157393	a	oooooooo+	C20orf62	chromosome 20 open reading frame 62
157398	b	oo++++ooo	LOC130951	null
157425	b	oooo+oooo	SNTG2	syntrophin, gamma 2
157528	b	ooooo+ooo	GP5	glycoprotein V (platelet)
157547	c	oo++oooo+	FZD2	frizzled homolog 2 (Drosophila)
157555	b	ooo+ooooo	TRIP	null
157557	b	ooo+ooooo	null	null
157644	f	oo++----+	MMP21	matrix metallopeptidase 21
157659	b	ooo+ooooo	null	null
157665	a	oooooo+++	RNF12	ring finger protein 12
157702	g	oooo-----	NDUFV3	NADH dehydrogenase (ubiquinone) flavoprotein 3, 10kDa
157708	b	oooooo+oo	DKFZp666G057	null
157745	g	o--------	DQX1	DEAQ box polypeptide 1 (RNA-dependent ATPase)
157770	c	ooo++++o+	MAP3K15	mitogen-activated protein kinase kinase kinase 15
157775	b	o+ooooooo	UTS2	urotensin 2
157780	b	ooo+ooooo	SLC26A2	solute carrier family 26 (sulfate transporter), member 2
157795	b	ooo+ooooo	CD109	CD109 antigen (Gov platelet alloantigens)
157802	i	o--ooo---	SEC31L2	SEC31-like 2 (S. cerevisiae)
157891	b	ooo+ooooo	null	null
157943	a	oooooo+++	VEGF	vascular endothelial growth factor

157963	b	ooo+ooooo	CSPG4	chondroitin sulfate proteoglycan 4 (melanoma-associated)
157985	g	ooo------	AICDA	activation-induced cytidine deaminase
158015	b	ooo+ooooo	SLC22A9	solute carrier family 22 (organic anion/cation transporter), member 9
158064	b	ooo++oooo	KLHL25	kelch-like 25 (Drosophila)
158142	b	ooo+ooooo	WFS1	Wolfram syndrome 1 (wolframin)
158223	b	ooo+ooooo	SRR	serine racemase
158227	a	oooooo+++	BRAF	v-raf murine sarcoma viral oncogene homolog B1
158258	b	ooo+ooooo	TLN2	talin 2
158262	b	ooo+ooooo	F2R	coagulation factor II (thrombin) receptor
158358	f	oo++----+	null	null
158366	c	ooo+oo+++	VEZATIN	null
158398	b	oooooo+oo	CRKRS	Cdc2-related kinase, arginine/serine-rich
158400	a	ooo++++++	EPC2	enhancer of polycomb homolog 2 (Drosophila)
158401	f	oo++----+	LOC401072	null
158446	b	ooooo+ooo	BRDG1	null
158495	a	ooo++++++	DDR2	discoidin domain receptor family, member 2
158499	a	oo+++++++	RIBC2	RIB43A domain with coiled-coils 2
158532	g	o--------	WDR42A	WD repeat domain 42A
158554	b	ooo+ooooo	FLJ23342	null
158597	b	oooo+oooo	AMPH	amphiphysin (Stiff-Man syndrome with breast cancer 128kDa autoantigen)
158604	b	ooo+ooooo	SORT1	sortilin 1
158607	a	ooo++++++	GABARAPL1	GABA(A) receptor-associated protein like 1
158610	b	ooo+ooooo	TRIM24	tripartite motif-containing 24
158641	b	oo+oooooo	CMTM7	CKLF-like MARVEL transmembrane domain containing 7
158652	g	oooooooo-	CD81	CD81 antigen (target of antiproliferative antibody 1)
158653	a	ooo++++++	MATR3	matrin 3
158661	c	oo++++o++	JMJD2B	jumonji domain containing 2B
158664	g	oooooooo-	XAB2	XPA binding protein 2
158702	b	ooo+ooooo	CA5B	carbonic anhydrase VB, mitochondrial
158737	b	oo+oooooo	CDC2L5	cell division cycle 2-like 5 (cholinesterase-related cell division controller)
158739	b	ooo+ooooo	GOLGA4	golgi autoantigen, golgin subfamily a, 4
158761	f	oo++----+	ICAM4	intercellular adhesion molecule 4, Landsteiner-Wiener blood group
158810	g	ooooooo--	DGCR2	DiGeorge syndrome critical region gene 2
158840	b	oooo+oooo	null	null
158887	a	o++++++++	SLC27A3	solute carrier family 27 (fatty acid transporter), member 3
158896	b	o+ooooooo	ZNF594	zinc finger protein 594
158926	b	ooo++oooo	null	null
158950	a	oooooo+++	SGK	serum/glucocorticoid regulated kinase
159001	b	ooo+ooooo	null	null
159012	i	o-----o--	LOC92345	null
159025	b	ooo+ooooo	CCL15|CCL14	chemokine (C-C motif) ligand 15|chemokine (C-C motif) ligand 14
159032	b	ooo+ooooo	ATP7A	ATPase, Cu++ transporting, alpha polypeptide (Menkes syndrome)
159086	a	oo+++++++	ZNF212	zinc finger protein 212
159141	b	o+ooooooo	MGC61571	null
159170	a	ooooooo++	null	null
159180	b	ooo+ooooo	FBXL17	F-box and leucine-rich repeat protein 17
159222	h	o-ooooooo	NKIRAS1	NFKB inhibitor interacting Ras-like 1
159269	h	o-ooooooo	NOL9	nucleolar protein 9
159293	b	ooooooo+o	null	null
159300	h	o--oooooo	EBI2	Epstein-Barr virus induced gene 2 (lymphocyte-specific G protein-coupled receptor)
159331	j	o-------+	null	null
159381	g	oooo-----	SLC25A15	solute carrier family 25 (mitochondrial carrier; ornithine transporter) member 15
159394	a	oooo+++++	CX3CL1	chemokine (C-X3-C motif) ligand 1
159434	b	ooo+ooooo	VIPR1	vasoactive intestinal peptide receptor 1
159465	b	ooooooo+o	CCL27	chemokine (C-C motif) ligand 27
159519	g	o--------	null	null
159545	a	ooo++++++	ZNF250	zinc finger protein 250
159555	a	ooooo++++	MIDN	midnolin
159556	b	ooooo+ooo	SPAM1	sperm adhesion molecule 1 (PH-20 hyaluronidase, zona pellucida binding)
159563	b	ooo+ooooo	NPHP3	nephronophthisis 3 (adolescent)
159579	g	o--------	SSX1	synovial sarcoma, X breakpoint 1
159630	b	ooooo++oo	EGFL6	EGF-like-domain, multiple 6
159704	g	o--------	null	null
159713	b	ooo+++ooo	null	null
159770	i	ooooo-o--	DGKA	diacylglycerol kinase, alpha 80kDa
159844	c	oooo+oo++	IL11	interleukin 11
159851	b	oooooo+oo	null	null
159861	b	ooo+ooooo	ST14	suppression of tumorigenicity 14 (colon carcinoma)
159867	b	ooooo+ooo	CX62	null
159875	b	ooo+ooooo	ZFHX1B	zinc finger homeobox 1b

159877	a	ooo++++++	DKFZp667M2411|LRRC37B	leucine rich repeat containing 37B
159897	b	ooo+ooooo	C15orf29	chromosome 15 open reading frame 29
159898	g	oooo-----	null	null
159901	g	ooooo----	CCL2	chemokine (C-C motif) ligand 2
159936	i	o-----o--	NELF	nasal embryonic LHRH factor
159965	i	o---o----	USP26	ubiquitin specific peptidase 26
159966	b	o+ooooooo	HEMGN	hemogen
159984	b	ooo+ooooo	null	null
160011	c	oo++oooo+	FLJ25169	null
160071	a	oooooo+++	CXorf15	chromosome X open reading frame 15
160115	c	oooo+ooo+	SNPH	syntaphilin
160147	i	oo-o-----	LOC441208	null
160158	j	o-ooo++++	CCL5	chemokine (C-C motif) ligand 5
160161	a	o++++++++	C6orf210	chromosome 6 open reading frame 210
160162	b	oooo+oooo	null	null
160257	a	oooooo+++	DDIT4	DNA-damage-inducible transcript 4
160271	h	o-----ooo	CEBPB	CCAAT/enhancer binding protein (C/EBP), beta
160273	b	ooo++oooo	ZADH2	zinc binding alcohol dehydrogenase, domain containing 2
160306	h	oooooo-oo	null	null
160314	g	o--------	DBNL	drebrin-like
160430	h	o-ooooooo	SFXN1	sideroflexin 1
160518	a	oooooo+++	EIF5	eukaryotic translation initiation factor 5
160611	a	oo+++++++	null	null
160628	b	ooooooo+o	MGC12760	null
160701	g	oooo-----	WDR6	WD repeat domain 6
160721	i	o------o-	null	null
160733	b	oooo+oooo	CYP2A6	cytochrome P450, family 2, subfamily A, polypeptide 6
160743	a	oo+++++++	ZFP36L1	zinc finger protein 36, C3H type-like 1
160876	c	oo+++ooo+	ADAMTS9	ADAM metallopeptidase with thrombospondin type 1 motif, 9
160938	b	o+ooooooo	TGM1	transglutaminase 1 (K polypeptide epidermal type I, protein-glutamine-gamma-glutamyltransferase)
161015	b	ooo+ooooo	PDCD1	programmed cell death 1
161035	b	ooo+ooooo	EMR2	egf-like module containing, mucin-like, hormone receptor-like 2
161043	b	o++++oooo	ZNF138	zinc finger protein 138
161049	h	o-ooooooo	XKR5	X Kell blood group precursor-related family, member 5
161050	b	oooooo+oo	null	null
161079	a	ooooo++++	WHDC1L1|WHDC1L2|LOC440259	WAS protein homology region 2 domain containing 1-like 1|WAS protein homology region 2 domain containing 1-like 2
161081	b	oooo+oooo	KIRREL2	kin of IRRE like 2 (Drosophila)
161094	h	oooo----o	LOC127309	null
161179	b	ooo+ooooo	null	null
161183	b	ooo+ooooo	PTGFRN	prostaglandin F2 receptor negative regulator
161192	b	ooo+++++o	TCAM1	testicular cell adhesion molecule 1 homolog (mouse)
161202	b	ooo+ooooo	null	null
161212	b	ooo++++oo	GPNMB	glycoprotein (transmembrane) nmb
161261	h	oo-oooooo	HHEX	homeobox, hematopoietically expressed
161300	b	ooo+ooooo	AACS	acetoacetyl-CoA synthetase
161318	h	oooooo-oo	MPDZ	multiple PDZ domain protein
161333	g	oooo-----	IFI16	interferon, gamma-inducible protein 16
161349	b	oooo+oooo	COL24A1	collagen, type XXIV, alpha 1
161401	b	oo+oooooo	null	null
161404	g	oooooooo-	ASF1B	ASF1 anti-silencing function 1 homolog B (S. cerevisiae)
161432	b	ooo+ooooo	FAM63B	family with sequence similarity 63, member B
161443	b	oo++ooooo	TFCP2	transcription factor CP2
161445	g	ooooo----	GTF2IRD2B|GTF2IRD2|GTF2IRD2P	null
161476	b	oooooo+oo	MC3R	melanocortin 3 receptor
161540	h	oooo--ooo	ZMYND11	zinc finger, MYND domain containing 11
161569	a	oooooo+++	MGC14425	null
161580	b	ooooo+ooo	null	null
161620	b	ooooooo+o	SULT1C3	null
161637	f	oo++----+	C9orf94	chromosome 9 open reading frame 94
161641	k	ooo---+oo	null	null
161660	a	ooooooo++	FAM80B	family with sequence similarity 80, member B
161668	h	o-ooooooo	KHDRBS2	KH domain containing, RNA binding, signal transduction associated 2
161671	a	ooo++++++	PTPN21	protein tyrosine phosphatase, non-receptor type 21
161674	b	ooo+ooooo	UST	uronyl-2-sulfotransferase
161676	h	oooo----o	null	null
161697	a	oo+++++++	MGC11082	null
161729	c	ooo+oo+++	BTN2A1	butyrophilin, subfamily 2, member A1

161757	h	ooooooo-o	ABO	ABO blood group (transferase A, alpha 1-3-N-acetylgalactosaminyltransferase; transferase B, alpha 1-3-galactosyltransferase)
161783	g	ooooooo--	SLC43A2	solute carrier family 43, member 2
161807	g	o--------	DPYSL4	dihydropyrimidinase-like 4
161949	b	ooo+ooooo	FLJ34077	null
162002	b	oooo+oooo	XYLT2	xylosyltransferase II
162024	b	ooo+ooooo	MGC16169	null
162141	b	ooo+ooooo	TFDP2	transcription factor Dp-2 (E2F dimerization partner 2)
162160	g	oooooooo-	NDUFS8	NADH dehydrogenase (ubiquinone) Fe-S protein 8, 23kDa (NADH-coenzyme Q reductase)
162178	g	oooooooo-	BCAT2	branched chain aminotransferase 2, mitochondrial
162197	i	o--ooooo-	C1QB	complement component 1, q subcomponent, beta polypeptide
162222	b	oooo+oooo	PRIC285	null
162223	i	o-----o--	GSK3A	glycogen synthase kinase 3 alpha
162227	b	ooo+ooooo	CYLD	cylindromatosis (turban tumor syndrome)
162261	a	ooo++++++	CTGLF1	centaurin, gamma-like family, member 1
162321	b	ooo+ooooo	SCARB2	scavenger receptor class B, member 2
162336	b	ooooooo+o	PTPRD	protein tyrosine phosphatase, receptor type, D
162364	b	oooooo+oo	null	null
162374	h	oooooo-oo	null	null
162382	a	ooooo++++	HIST1H2AB	histone 1, H2ab
162429	i	o-----o--	SPINK1	serine peptidase inhibitor, Kazal type 1
162483	g	ooo------	BRI3	brain protein I3
162548	b	ooooooo+o	EFEMP2	EGF-containing fibulin-like extracellular matrix protein 2
162561	b	ooo+ooooo	NEDD9	neural precursor cell expressed, developmentally down-regulated 9
162631	h	o-ooooooo	null	null
162644	b	ooo+ooooo	RP11-378J18.4|LOC440751	null
162651	h	o-ooooooo	RP1-112K5.2	null
162661	b	ooo+ooooo	C8orf39	chromosome 8 open reading frame 39
162683	b	oooo+oooo	SLC8A2	solute carrier family 8 (sodium-calcium exchanger), member 2
162748	g	oooooooo-	GPR126	G protein-coupled receptor 126
162764	h	o----oooo	IRX5	iroquois homeobox protein 5
162894	b	ooooooo+o	TMEM108	transmembrane protein 108
162911	a	oooooo+++	THBS1	thrombospondin 1
162987	b	oooooo+oo	LOC402280	null
163001	h	o-ooooooo	SLC25A37	solute carrier family 25, member 37
163015	d	ooo++ooo-	IFITM5	null
163073	b	o+ooooooo	PSG9	pregnancy specific beta-1-glycoprotein 9
163084	b	ooo+ooooo	ECOP	null
163115	b	ooo+ooooo	UNQ9391	null
163152	a	ooo++++++	null	null
163234	b	ooooooo+o	STAP2	null
163241	a	oo+++++++	IL6	interleukin 6 (interferon, beta 2)
163264	b	oooo+oooo	D21S2088E	null
163281	b	ooo+ooooo	SPAST	spastin
163285	a	ooo++++++	C9orf123	chromosome 9 open reading frame 123
163365	b	ooooooo+o	TPO	thyroid peroxidase
163408	h	o-ooooooo	SIAH2	seven in absentia homolog 2 (Drosophila)
163409	g	ooo------	null	null
163480	c	ooo+ooo++	HP	haptoglobin
163509	a	ooo++++++	DDIT3	DNA-damage-inducible transcript 3
163514	g	o--------	FLJ38723	null
163534	b	ooo+ooooo	COMMD3	COMM domain containing 3
163543	i	o-oo-----	PLEKHG3	pleckstrin homology domain containing, family G (with RhoGef domain) member 3
163583	i	o--oooo--	MGC3121	null
163612	a	ooo++++++	IER2	immediate early response 2
163698	h	oooooo-oo	FLJ16636	null
163709	c	ooooo++o+	ZNF193	zinc finger protein 193
163733	b	ooooo+ooo	SLC4A7	solute carrier family 4, sodium bicarbonate cotransporter, member 7
163826	i	oo--o----	LOC387643	null
163861	a	ooo++++++	FGD6	FYVE, RhoGEF and PH domain containing 6
163877	b	ooo+ooooo	SNX25	sorting nexin 25
163892	a	oo+++++++	LOC283130	null
163914	a	ooo++++++	PARP8	poly (ADP-ribose) polymerase family, member 8
163920	a	ooo++++++	UHRF2	ubiquitin-like, containing PHD and RING finger domains, 2
163966	b	ooo++oooo	C6orf105	chromosome 6 open reading frame 105
163968	e	oo++----o	FLJ25818	null
163998	b	ooo++++oo	CSMD2	CUB and Sushi multiple domains 2
164001	g	ooooo----	ZFP106	zinc finger protein 106 homolog (mouse)

164053	a	ooo++++++	PDE4D	phosphodiesterase 4D, cAMP-specific (phosphodiesterase E3 dunce homolog, Drosophila)
164069	b	ooo+ooooo	RNF184	ring finger protein 184
164075	b	ooo++oooo	ZNF42	zinc finger protein 42 (myeloid-specific retinoic acid-responsive)
164081	b	ooo+ooooo	LOC441994	null
164088	b	ooo+ooooo	SMAD3	SMAD, mothers against DPP homolog 3 (Drosophila)
164172	a	oooooo+++	FKBPL	FK506 binding protein like
164174	b	ooo+ooooo	ZNF680	zinc finger protein 680
164291	b	ooooooo+o	CXXC1	CXXC finger 1 (PHD domain)
164337	c	oo++oooo+	null	null
164385	g	oooo-----	CGI-38	null
164416	c	oo++oooo+	PHLDB3	pleckstrin homology-like domain, family B, member 3
164457	a	ooo++++++	NXF1	nuclear RNA export factor 1
164477	b	oooooo+oo	null	null
164511	a	oooooo+++	null	null
164558	g	ooooooo--	ASMTL	acetylserotonin O-methyltransferase-like
164636	h	oooo----o	OR1E2	olfactory receptor, family 1, subfamily E, member 2
164694	h	oooo-oooo	NOLA1	nucleolar protein family A, member 1 (H/ACA small nucleolar RNPs)
164701	b	ooo+ooooo	DLG3	discs, large homolog 3 (neuroendocrine-dlg, Drosophila)
164716	b	oooooo+oo	BCL3	B-cell CLL/lymphoma 3
164721	g	o--------	RPS6KB2	ribosomal protein S6 kinase, 70kDa, polypeptide 2
164834	b	ooooooo+o	null	null
164901	a	ooooo++++	LOC128439	null
164935	g	ooooooo--	FTCD	formiminotransferase cyclodeaminase
165101	h	oooo----o	null	null
165177	b	ooo+ooooo	LYST	lysosomal trafficking regulator
165191	b	ooo+ooooo	null	null
165200	b	oooooo+oo	UCP2	uncoupling protein 2 (mitochondrial, proton carrier)
165230	b	ooo+ooooo	ZBTB4	zinc finger and BTB domain containing 4
165289	b	oooo+oooo	AGTR2	angiotensin II receptor, type 2
165297	h	oooo----o	RGSL2	regulator of G-protein signalling like 2
165327	b	ooo++oooo	null	null
165334	g	o--------	RBAK	null
165355	b	ooooooo+o	null	null
165359	b	ooo++oooo	DYNC1H1	dynein, cytoplasmic 1, heavy chain 1
165396	h	o-ooooooo	DOCK1	dedicator of cytokinesis 1
165400	f	oo++----+	LOC118430	null
165425	g	ooooo----	CENPE	centromere protein E, 312kDa
165436	b	oooo+oooo	FLJ42117	null
165440	b	oooooo++o	MTA2	metastasis associated 1 family, member 2
165472	b	ooo++oooo	TMEM63A	transmembrane protein 63A
165482	h	oooo--ooo	DKK1	dickkopf homolog 1 (Xenopus laevis)
165607	b	o+ooooooo	SLC11A1	solute carrier family 11 (proton-coupled divalent metal ion transporters), member 1
165608	a	ooo++++++	null	null
165636	a	oo+++++++	LIFR	leukemia inhibitory factor receptor
165643	b	ooo+ooooo	FLJ12788	null
165654	b	ooo+ooooo	ZNF611	zinc finger protein 611
165658	a	o++++++++	KIAA1333	KIAA1333
165684	b	oooooo+oo	BTN1A1	butyrophilin, subfamily 1, member A1
165736	b	ooo+ooooo	null	null
165769	b	ooo+ooooo	TCEAL3	transcription elongation factor A (SII)-like 3
165776	b	ooo++oooo	FLJ13614	null
165870	g	o--------	UTS2	urotensin 2
165993	a	ooooo++++	HSPC157	null
165998	i	o-----o--	NCSTN	nicastrin
166020	a	ooo++++++	CYR61	cysteine-rich, angiogenic inducer, 61
166052	b	ooooo+ooo	APLN	apelin, AGTRL1 ligand
166108	h	oo----ooo	SAS10	null
166111	b	ooo++++oo	CD248	CD248 antigen, endosialin
166185	b	ooo+ooooo	KRT10	keratin 10 (epidermolytic hyperkeratosis; keratosis palmaris et plantaris)
166193	b	ooooooo+o	FOXP2	forkhead box P2
166213	a	oooooooo+	2'-PDE	null
166226	a	o++++++++	FBXL5	F-box and leucine-rich repeat protein 5
166285	h	ooooooo-o	C10orf6	chromosome 10 open reading frame 6
166428	a	ooo++++++	RAP80	null
166429	h	ooooo-ooo	C17orf61|PLSCR3	chromosome 17 open reading frame 61|phospholipid scramblase 3
166474	g	oooo-----	SKP2	S-phase kinase-associated protein 2 (p45)
166495	b	ooooo+ooo	LOC388927	null
166505	b	ooo+ooooo	SPAG17	sperm associated antigen 17
166544	a	ooo++++++	CUGBP1	CUG triplet repeat, RNA binding protein 1

166592	c	ooo+++o++	FGD4	FYVE, RhoGEF and PH domain containing 4
166713	b	ooo+++ooo	null	null
166732	b	ooo+ooooo	HERC2	hect domain and RLD 2
166744	b	ooo+ooooo	DHX40	DEAH (Asp-Glu-Ala-His) box polypeptide 40
166747	a	oooooo+++	C20orf111	chromosome 20 open reading frame 111
166860	a	o++++++++	IQCB1	IQ motif containing B1
166864	b	oooooo+oo	null	null
166928	a	o++++++++	NISCH	nischarin
166946	a	oooooo+++	ATF4	activating transcription factor 4 (tax-responsive enhancer element B67)
167052	b	ooo+ooooo	RNGTT	RNA guanylyltransferase and 5'-phosphatase
167055	b	ooo+ooooo	KIAA1468	KIAA1468
167066	a	ooooooo++	null	null
167095	b	ooo+ooooo	NUP210L	nucleoporin 210kDa-like
167121	a	oo+++++++	ASCC3	activating signal cointegrator 1 complex subunit 3
167186	b	oooooo+oo	null	null
167201	a	o++++++++	VPS13A	vacuolar protein sorting 13A (yeast)
167234	b	ooooooo+o	NRK	Nik related kinase
167266	a	ooooo++++	MGC12935	null
167292	b	ooo+ooooo	CHUK	conserved helix-loop-helix ubiquitous kinase
167298	b	ooo+ooooo	F8	coagulation factor VIII, procoagulant component (hemophilia A)
167316	b	ooo+ooooo	MATN2	matrilin 2
167321	b	oooooo+oo	CALM3	calmodulin 3 (phosphorylase kinase, delta)
167324	a	oo+++++++	ACRC	acidic repeat containing
167329	b	ooo+ooooo	MORC3	MORC family CW-type zinc finger 3
167444	b	oooo+oooo	PRR4|FRYL	proline rich 4 (lacrimal)|furry homolog-like (Drosophila)
167471	a	oo+++++++	SLC22A18AS	solute carrier family 22 (organic cation transporter), member 18 antisense
167512	a	oooooooo+	HIST1H3F	histone 1, H3f
167559	b	oooooo+oo	IRF2	interferon regulatory factor 2
167566	b	ooooooo+o	LHFPL5	lipoma HMGIC fusion partner-like 5
167606	b	ooo+ooooo	XPO4	exportin 4
167621	a	ooo++++++	ZNF281	zinc finger protein 281
167664	a	oooooo+++	SNAPC1	small nuclear RNA activating complex, polypeptide 1, 43kDa
167735	c	oooo+oo++	MGC29891	null
167741	a	oooo+++++	FLJ20464	null
167759	a	ooo++++++	ARNTL	aryl hydrocarbon receptor nuclear translocator-like
167773	c	ooo+oo+++	ACVR2A	activin A receptor, type IIA
167799	g	o--------	ANXA8	annexin A8
167868	h	o-----ooo	MAD2L1BP	MAD2L1 binding protein
167891	g	oooooooo-	GALNT2	UDP-N-acetyl-alpha-D-galactosamine:polypeptide N-acetylgalactosaminyltransferase 2 (GalNAc-T2)
167953	b	oooooo+oo	MOSPD3	motile sperm domain containing 3
167987	b	ooo+ooooo	RBM35B	RNA binding motif protein 35B
168032	g	o--------	CHRNA2	cholinergic receptor, nicotinic, alpha polypeptide 2 (neuronal)
168042	b	ooooo+ooo	GRIP1	glutamate receptor interacting protein 1
168085	b	oo+oooooo	null	null
168093	b	ooo+ooooo	KLHL7	kelch-like 7 (Drosophila)
168108	g	o--------	null	null
168127	c	ooo++oo++	PTGS1	prostaglandin-endoperoxide synthase 1 (prostaglandin G/H synthase and cyclooxygenase)
168130	a	oooooooo+	LALBA	lactalbumin, alpha-
168184	h	o-ooooooo	ARL16	ADP-ribosylation factor-like 16
168215	b	oooooo++o	HHLA3	HERV-H LTR-associating 3
168270	h	oooooo-oo	MGC12966	null
168370	h	oooo----o	GOLPH4	golgi phosphoprotein 4
168384	b	oooo+oooo	IL8RA	interleukin 8 receptor, alpha
168414	b	ooo+ooooo	BF	B-factor, properdin
168425	i	o-----o--	TLN1	talin 1
168429	b	oo+++++oo	RIBC1	RIB43A domain with coiled-coils 1
168436	b	ooo++oooo	CD74	CD74 antigen (invariant polypeptide of major histocompatibility complex, class II antigen-associated)
168498	h	o-ooooooo	DAPK3	death-associated protein kinase 3
168565	a	ooo++++++	CARF	null
168584	b	oooooo+oo	RFFL	ring finger and FYVE-like domain containing 1
168609	b	ooo+ooooo	null	null
168621	b	ooooooo+o	null	null
168667	b	ooo+ooooo	PEX6	peroxisomal biogenesis factor 6
168682	b	ooooooo+o	LRAP	null
168722	g	o--------	BCL2L11	BCL2-like 11 (apoptosis facilitator)
168727	a	ooooooo++	ZNF187	zinc finger protein 187
168731	b	ooo+ooooo	ZNF26	zinc finger protein 26 (KOX 20)
168741	a	ooo++++++	TNFAIP3	tumor necrosis factor, alpha-induced protein 3

168744	a	o++++++++	SNX19	sorting nexin 19
168751	b	ooo+ooooo	C13orf7	chromosome 13 open reading frame 7
168773	a	oo+++++++	HOXC9	homeobox C9
168806	a	oooooooo+	null	null
168813	b	ooo+ooooo	OGDHL	oxoglutarate dehydrogenase-like
168890	b	ooo+ooooo	null	null
168914	b	ooo+ooooo	HSMPP8	null
168915	g	oo-------	TUBB2C	tubulin, beta 2C
169001	b	oooo+oooo	FSHR	follicle stimulating hormone receptor
169023	b	ooo+ooooo	null	null
169051	h	o-------o	RKHD3	ring finger and KH domain containing 3
169116	b	oooo+oooo	KLK2	kallikrein 2, prostatic
169119	a	ooo++++++	FZD4	frizzled homolog 4 (Drosophila)
169123	g	o--------	ACTN4	actinin, alpha 4
169183	b	ooo+ooooo	APC	adenomatosis polyposis coli
169298	b	ooo+ooooo	EEA1	early endosome antigen 1, 162kD
169311	c	ooo+++o++	IPMK	inositol polyphosphate multikinase
169331	b	ooo++oooo	ECAT11	null
169345	g	oooooooo-	NUDCD3	NudC domain containing 3
169349	g	o--------	null	null
169378	b	ooooo+ooo	PDZK2	PDZ domain containing 2
169395	h	o-ooooooo	OXR1	oxidation resistance 1
169428	a	oooooooo+	null	null
169440	b	ooo+ooooo	SPEN	spen homolog, transcriptional regulator (Drosophila)
169469	g	oooo-----	SLC1A1	solute carrier family 1 (neuronal/epithelial high affinity glutamate transporter, system Xag), member 1
169477	a	ooo++++++	STATIP1	signal transducer and activator of transcription 3 interacting protein 1
169478	b	ooo+ooooo	EXOC8	exocyst complex component 8
169514	b	o++++oooo	null	null
169515	b	ooo+ooooo	ZNF529	zinc finger protein 529
169601	h	oooooo-oo	MTFR1	mitochondrial fission regulator 1
169608	h	ooooooo-o	KIAA1463	null
169614	b	ooo+ooooo	CPD	carboxypeptidase D
169622	g	o--------	ZZEF1	zinc finger, ZZ-type with EF-hand domain 1
169674	b	ooo+ooooo	PSMD9	proteasome (prosome, macropain) 26S subunit, non-ATPase, 9
169678	g	o--------	null	null
169704	h	oooo----o	TRPM3	transient receptor potential cation channel, subfamily M, member 3
169707	b	ooo+ooooo	MON1B	MON1 homolog B (yeast)
169723	g	ooooooo--	PBK	PDZ binding kinase
169792	b	oooo+oooo	USP54	ubiquitin specific peptidase 54
169848	c	ooo+ooo++	SIPA1L2	signal-induced proliferation-associated 1 like 2
169853	h	o-ooooooo	NKX3-1	NK3 transcription factor related, locus 1 (Drosophila)
169898	b	ooo+ooooo	RNF2	ring finger protein 2
169909	b	oooo+oooo	dJ222E13.2	null
169945	g	o--------	NDRG1	N-myc downstream regulated gene 1
169985	g	ooooooo--	FLJ14800	null
170016	b	ooooo+ooo	C1QA	complement component 1, q subcomponent, alpha polypeptide
170041	a	ooo++++++	NPC1	Niemann-Pick disease, type C1
170083	b	ooo+ooooo	LOC440547|LOC440395|LOC441122|LOC441185|LOC442492|LOC440018	null
170116	b	ooo+ooooo	C9orf121	chromosome 9 open reading frame 121
170168	c	oo++oo+++	null	null
170248	h	oooooo-oo	DLG2	discs, large homolog 2, chapsyn-110 (Drosophila)
170258	b	ooo+ooooo	HLA-DQA1	major histocompatibility complex, class II, DQ alpha 1
170285	g	ooooooo--	SNCA	synuclein, alpha (non A4 component of amyloid precursor)
170317	i	o-oo-----	LOC440334	null
170411	g	oo-------	CFLAR	CASP8 and FADD-like apoptosis regulator
170455	a	ooooo++++	ACVRL1	activin A receptor type II-like 1
170532	b	ooo++++oo	CD226	CD226 antigen
170533	h	o-ooooooo	NBR2	neighbor of BRCA1 gene 2
170538	a	oo+++++++	CRISPLD2	cysteine-rich secretory protein LCCL domain containing 2
170540	b	ooo+ooooo	GCL	null
170545	b	ooo+++++o	OVOS2	null
170572	b	ooo+ooooo	TMEM64	transmembrane protein 64
170585	b	ooooo++oo	DMRTB1	DMRT-like family B with proline-rich C-terminal, 1
170626	a	ooo++++++	LOC284422	null
170644	a	ooo++++++	USP34	ubiquitin specific peptidase 34
170657	b	oooo+oooo	SLC30A8	solute carrier family 30 (zinc transporter), member 8
170698	a	ooooo++++	null	null

170707	b	oooo+oooo	LOC401007	null
170749	b	oooooo+oo	TGFB1	transforming growth factor, beta 1 (Camurati-Engelmann disease)
170767	b	ooo+ooooo	POLD1	polymerase (DNA directed), delta 1, catalytic subunit 125kDa
170769	b	ooo+ooooo	LOC220929	null
170832	b	oooooo+oo	null	null
170863	h	oooooo-oo	MCART1	mitochondrial carrier triple repeat 1
170883	b	ooo+ooooo	BC002942	null
170951	a	ooo++++++	SMOX	spermine oxidase
170963	g	oooooooo-	TBC1D16	TBC1 domain family, member 16
170978	b	ooooooo+o	null	null
170989	a	ooo++++++	ZNF217	zinc finger protein 217
171023	h	oooooo-oo	IFT57	intraflagellar transport 57 homolog (Chlamydomonas)
171047	a	oooooooo+	null	null
171053	a	ooooooo++	C17orf32	chromosome 17 open reading frame 32
171071	b	ooo+ooooo	CCDC41	coiled-coil domain containing 41
171123	b	ooo+ooooo	EXOC2	exocyst complex component 2
171126	h	o-ooooooo	SCAMP4	secretory carrier membrane protein 4
171151	g	ooooooo--	ZNF628	zinc finger protein 628
171175	b	ooo+++ooo	LOC441038	null
171202	g	oooooooo-	SIVA	null
171212	g	oooooooo-	XCR1	chemokine (C motif) receptor 1
171242	a	oooooooo+	LOC387978	null
171247	g	o--------	TSPAN7	tetraspanin 7
171273	c	ooo+oo+++	RP5-1022P6.2	null
171293	g	ooooooo--	CHTF18	CTF18, chromosome transmission fidelity factor 18 homolog (S. cerevisiae)
171374	b	ooooooo+o	null	null
171385	b	ooo+ooooo	ARFGEF2	ADP-ribosylation factor guanine nucleotide-exchange factor 2 (brefeldin A-inhibited)
171420	b	ooo+ooooo	KLHL21	kelch-like 21 (Drosophila)
171433	b	ooooo+++o	SLC5A2	solute carrier family 5 (sodium/glucose cotransporter), member 2
171440	i	o-oo-----	ZW10	ZW10, kinetochore associated, homolog (Drosophila)
171498	a	oooooooo+	null	null
171513	a	oooooo+++	FLJ22833	null
171533	a	oo+++++++	C1orf109	chromosome 1 open reading frame 109
171555	b	ooooooo+o	CALML3	calmodulin-like 3
171576	b	oooo+oooo	EPS15	epidermal growth factor receptor pathway substrate 15
171586	b	ooo+ooooo	PTPRG	protein tyrosine phosphatase, receptor type, G
171589	c	ooo+o++++	ZFX	zinc finger protein, X-linked
171603	h	o-ooooooo	DOK4	docking protein 4
171678	g	o--------	OR8J3	olfactory receptor, family 8, subfamily J, member 3
171686	b	ooo+ooooo	C7orf27	chromosome 7 open reading frame 27
171706	d	ooo+o----	DKFZp434K2435	null
171786	h	o-ooooooo	RIOK1	RIO kinase 1 (yeast)
171828	b	ooo+ooooo	DFFB	DNA fragmentation factor, 40kDa, beta polypeptide (caspase-activated DNase)
171874	b	ooo+ooooo	ZNF651	zinc finger protein 651
171900	h	oooo----o	KAZALD1	Kazal-type serine peptidase inhibitor domain 1
171931	h	oooooo-oo	CHRNB2	cholinergic receptor, nicotinic, beta polypeptide 2 (neuronal)
171939	g	o--------	LOC441462	null
171943	b	oooooo+oo	AK5	adenylate kinase 5
171959	h	o-ooooooo	CYP2D7P1	cytochrome P450, family 2, subfamily D, polypeptide 7 pseudogene 1
172036	b	ooo+ooooo	null	null
172051	i	o-oo-----	MATN4	matrilin 4
172111	h	o-----ooo	CITED4	Cbp/p300-interacting transactivator, with Glu/Asp-rich carboxy-terminal domain, 4
172112	h	oooo----o	FXN	frataxin
172171	h	o-ooooooo	EPPK1	epiplakin 1
172176	h	o----oooo	DUSP4	dual specificity phosphatase 4
172218	b	oooooo+oo	TBX1	T-box 1
172227	b	oooo+oooo	ZDHHC18	zinc finger, DHHC-type containing 18
172240	b	ooo+ooooo	ZCCHC7	zinc finger, CCHC domain containing 7
172274	b	ooo+ooooo	FAM46C	family with sequence similarity 46, member C
172284	b	oooo+oooo	LOC440792	null
172307	h	o-ooooooo	HIST2H2AB	histone 2, H2ab
172316	e	oo++----o	LPO	lactoperoxidase
172362	b	ooooooo+o	null	null
172376	b	ooooo+ooo	RP13-383K5.1	null
172392	i	o-oo-----	C7orf31	chromosome 7 open reading frame 31
172470	b	ooo+ooooo	CNOT1	CCR4-NOT transcription complex, subunit 1
172477	b	ooooo+ooo	KIAA1731	KIAA1731
172507	b	oooo+oooo	null	null
172590	b	oooo++ooo	null	null

172619	h	oooo----o	HLA-DOB	major histocompatibility complex, class II, DO beta
172634	a	ooooooo++	CYP2R1	cytochrome P450, family 2, subfamily R, polypeptide 1
172636	a	oooooooo+	RASSF2	Ras association (RalGDS/AF-6) domain family 2
172651	a	oooooo+++	null	null
172727	g	oooo-----	ANXA3	annexin A3
172733	a	ooo++++++	ENC1	ectodermal-neural cortex (with BTB-like domain)
172736	b	ooooooo+o	LOC205251	null
172752	b	ooo+ooooo	FBXO34	F-box protein 34
172773	a	ooo++++++	ARRDC3	arrestin domain containing 3
172828	i	oooo-o---	BOLA3	bolA-like 3 (E. coli)
172863	h	o-ooooooo	null	null
172882	g	ooooooo--	GALR3	galanin receptor 3
172885	b	ooo+ooooo	NEUROG3	neurogenin 3
172904	c	oooo+oo++	HIST3H2BB	histone 3, H2bb
172931	a	ooo++++++	CIP29	null
172942	b	oooooo+oo	C10orf33	chromosome 10 open reading frame 33
172975	b	o+ooooooo	GRID2	glutamate receptor, ionotropic, delta 2
172984	h	oooo----o	MGC99813	null
173060	a	oooooooo+	ATP2A1	ATPase, Ca++ transporting, cardiac muscle, fast twitch 1
173074	b	ooo+ooooo	BCL9L	B-cell CLL/lymphoma 9-like
173145	a	oooooooo+	null	null
173155	a	oooo+++++	FLJ10038	null
173188	b	ooo+ooooo	COG6	component of oligomeric golgi complex 6
173202	b	oooooo++o	EIF5A|LOC143244	eukaryotic translation initiation factor 5A
173221	b	ooo+ooooo	LAMA1	laminin, alpha 1
173294	a	ooooo++++	ANKRD37	ankyrin repeat domain 37
173320	a	ooo++++++	JMJD1C	jumonji domain containing 1C
173339	b	oooooo+oo	NRBP1	nuclear receptor binding protein 1
173342	b	ooo+ooooo	RASSF5	Ras association (RalGDS/AF-6) domain family 5
173350	g	oooooooo-	FCRLM2	Fc receptor-like and mucin-like 2
173367	h	o-ooooooo	null	null
173378	a	oo+++++++	GNL3L	guanine nucleotide binding protein-like 3 (nucleolar)-like
173408	a	oooooo+++	CXCL2	chemokine (C-X-C motif) ligand 2
173430	a	ooo++++++	C1orf71	chromosome 1 open reading frame 71
173449	b	oooooo+oo	CLTB	clathrin, light polypeptide (Lcb)
173470	a	ooooooo++	THUMPD2	THUMP domain containing 2
173504	h	ooooo---o	RSHL3	radial spokehead-like 3
173518	a	ooo++++++	CTDSP2	CTD (carboxy-terminal domain, RNA polymerase II, polypeptide A) small phosphatase 2
173549	a	ooooo++++	ZNFN1A5	zinc finger protein, subfamily 1A, 5
173582	a	ooo++++++	null	null
173591	i	oo-o-----	TMC8	transmembrane channel-like 8
173650	a	ooo++++++	ASXL1	additional sex combs like 1 (Drosophila)
173692	b	oooooo++o	FLJ20160	null
173703	h	oooooo-oo	ODF2	outer dense fiber of sperm tails 2
173842	b	ooooo+ooo	null	null
173863	b	ooo+ooooo	SLC17A5	solute carrier family 17 (anion/sugar transporter), member 5
173876	h	oooo-oooo	OACT2	O-acyltransferase (membrane bound) domain containing 2
173877	h	o-ooooooo	null	null
173878	b	ooo+ooooo	RAP1GA1	RAP1, GTPase activating protein 1
173910	b	ooo+ooooo	SUHW4	suppressor of hairy wing homolog 4 (Drosophila)
173915	b	ooo+ooooo	DCUN1D2	DCN1, defective in cullin neddylation 1, domain containing 2 (S. cerevisiae)
173923	c	oo+ooooo+	FANK1	fibronectin type III and ankyrin repeat domains 1
173946	b	ooooo+ooo	NKPD1	NTPase, KAP family P-loop domain containing 1
173979	g	o--------	GOLGA6|LOC440289|GOLGA	golgi autoantigen, golgin subfamily a, 6
173982	b	ooo+ooooo	EXOC3	exocyst complex component 3
173987	b	oo+oooooo	P2RY2	purinergic receptor P2Y, G-protein coupled, 2
173989	h	o-ooooooo	DPH5	DPH5 homolog (S. cerevisiae)
174000	b	oooooo+oo	SNX24	sorting nexing 24
174022	b	ooo+++++o	SMP3	null
174130	b	o+ooooooo	CECR6	cat eye syndrome chromosome region, candidate 6
174173	g	o--------	null	null
174178	i	o--oooo--	LSS	lanosterol synthase (2,3-oxidosqualene-lanosterol cyclase)
174192	g	o--------	CRYGC	crystallin, gamma C
174201	b	ooo+ooooo	DOCK9	dedicator of cytokinesis 9
174214	b	ooo+ooooo	HIGD1B	HIG1 domain family, member 1B
174312	g	oooooooo-	OGDH	oxoglutarate (alpha-ketoglutarate) dehydrogenase (lipoamide)
174351	b	ooo+ooooo	CHAF1B	chromatin assembly factor 1, subunit B (p60)
174354	b	ooo+ooooo	HSPC049	null
174393	g	o--------	U2AF1L3	U2(RNU2) small nuclear RNA auxiliary factor 1-like 3

174449	c	oooo+oo++	null	null
174485	c	oo+++o+++	LOC339745	null
174510	b	ooo+ooooo	SIN3B	SIN3 homolog B, transcription regulator (yeast)
174543	a	ooo++++++	SPSB1	splA/ryanodine receptor domain and SOCS box containing 1
174556	a	ooo++++++	KLF2	Kruppel-like factor 2 (lung)
174568	b	ooo+ooooo	OSTalpha	null
174573	b	ooo+ooooo	IGF2R	insulin-like growth factor 2 receptor
174593	a	ooo++++++	YY1AP1	YY1 associated protein 1
174614	b	oooo+oooo	ZNF569	zinc finger protein 569
174627	b	oooo+oooo	null	null
174631	j	oooo----+	RAB41	RAB41, member RAS homolog family
174671	b	ooooooo+o	OR51L1	olfactory receptor, family 51, subfamily L, member 1
174713	b	ooo+ooooo	ITPR2	inositol 1,4,5-triphosphate receptor, type 2
174736	a	oooooo+++	VGF	VGF nerve growth factor inducible
174740	b	ooo+ooooo	TRIM2	tripartite motif-containing 2
174757	g	oooooo---	C10orf79	chromosome 10 open reading frame 79
174775	h	oooooo-oo	null	null
174790	b	ooooooo+o	TMSB4Y	thymosin, beta 4, Y-linked
174799	a	oo+++++++	C17orf27	chromosome 17 open reading frame 27
174818	a	oooooooo+	null	null
174838	b	ooooooo+o	LOC401358|LOC402523	null
174861	h	o-ooooooo	null	null
174874	b	ooo+ooooo	LKAP	null
174895	h	o-ooooooo	RIOK3	RIO kinase 3 (yeast)
174906	g	oooooooo-	IPO13	importin 13
174907	b	ooo+ooooo	CDC27	cell division cycle 27
174993	b	oooo+oooo	SEMA3E	sema domain, immunoglobulin domain (Ig), short basic domain, secreted, (semaphorin) 3E
175009	b	ooo+ooooo	PPFIBP1	PTPRF interacting protein, binding protein 1 (liprin beta 1)
175047	l	o-----+--	FKBP8	FK506 binding protein 8, 38kDa
175076	h	o-ooooooo	LOC441126	null
175105	g	o--------	STOML3	stomatin (EPB72)-like 3
175154	b	oooo++ooo	LOC440131	null
175188	a	ooooo++++	MGC5509	null
175254	b	oooooo+oo	ARF4P3	ADP-ribosylation factor 4 pseudogene 3
175270	b	oooooo+oo	SPATC1	spermatogenesis and centriole associated 1
175296	a	ooo++++++	BCL2L2	BCL2-like 2
175316	h	oo-oooooo	GTPBP1	GTP binding protein 1
175347	a	o++++++++	LOC440450	null
175418	g	oooooo---	RP11-19J3.3	null
175445	g	o--------	null	null
175465	b	ooo+ooooo	FLJ10996	null
175486	g	o--------	ST8SIA3	ST8 alpha-N-acetyl-neuraminide alpha-2,8-sialyltransferase 3
175491	j	o-oooo+++	BRD2	bromodomain containing 2
175513	a	ooo++++++	MED8	mediator of RNA polymerase II transcription, subunit 8 homolog (yeast)
175527	g	o--------	JAK1	Janus kinase 1 (a protein tyrosine kinase)
175565	b	ooo+ooooo	RP11-50D16.3	null
175569	b	ooo+ooooo	TMC6	transmembrane channel-like 6
175573	b	o+ooooooo	APOC1	apolipoprotein C-I
175637	f	oo++----+	FOXP3	forkhead box P3
175647	a	oooooooo+	SLC20A1	solute carrier family 20 (phosphate transporter), member 1
175648	g	o--------	MGC42638	null
175654	f	oo++----+	C20orf12	chromosome 20 open reading frame 12
175669	j	o-oooooo+	TOPORS	topoisomerase I binding, arginine/serine-rich
175672	b	ooooooo+o	DGCR6L	DiGeorge syndrome critical region gene 6-like
175720	b	ooooooo+o	null	null
175766	h	o-ooooooo	RWDD4A	RWD domain containing 4A
175788	b	oooooo+oo	SORBS3	sorbin and SH3 domain containing 3
175789	f	oo++----+	C20orf117	chromosome 20 open reading frame 117
175806	g	oooo-----	ARHGEF16	Rho guanine exchange factor (GEF) 16
175841	b	ooooooo+o	CNKSR1	connector enhancer of kinase suppressor of Ras 1
175863	c	ooo+o++++	ZNF77	zinc finger protein 77 (pT1)
175877	b	ooo+ooooo	EPHA7	EPH receptor A7
175887	b	ooooooo+o	PLTP	phospholipid transfer protein
175957	b	ooo+ooooo	KIAA0922	null
175966	a	ooooooo++	SMOC1	SPARC related modular calcium binding 1
175991	b	ooo+ooooo	null	null
176041	i	o-oo-----	null	null
176143	b	oooo+oooo	RP11-450P7.3	null
176144	b	ooo+ooooo	LOC256021	null

176162	b	oo+oooooo	C20orf117	chromosome 20 open reading frame 117
176228	b	ooooo+ooo	NDRG2	NDRG family member 2
176265	h	ooo-ooooo	S100A5	S100 calcium binding protein A5
176279	i	o-o------	PPP1R14A	protein phosphatase 1, regulatory (inhibitor) subunit 14A
176280	b	ooooo+ooo	LOC92689	null
176287	b	ooo+ooooo	MADD	MAP-kinase activating death domain
176306	h	oooo----o	C20orf166	chromosome 20 open reading frame 166
176322	a	ooooo++++	NOTCH2NL	Notch homolog 2 (Drosophila) N-terminal like
176416	h	o-ooooooo	PPP2R5E	protein phosphatase 2, regulatory subunit B (B56), epsilon isoform
176454	b	ooooo+ooo	TSPY2|TSPY1	testis specific protein, Y-linked 2|testis specific protein, Y-linked 1
176460	a	oooooooo+	DTNB	dystrobrevin, beta
176499	g	o--------	SNPH	syntaphilin
176580	a	oooooo+++	CGGBP1	CGG triplet repeat binding protein 1
176621	b	ooo+ooooo	LOC339077|LOC284701	null
176645	b	ooooo+ooo	CETN1	centrin, EF-hand protein, 1
176646	b	oooo+oooo	LRRC43	leucine rich repeat containing 43
176679	g	oooooooo-	LAMB2	laminin, beta 2 (laminin S)
176743	b	ooo+ooooo	GYG2	glycogenin 2
176749	b	ooo+ooooo	LOC153561	null
176760	h	oooo----o	null	null
176769	b	oooo+oooo	null	null
176788	i	o--o-----	CDADC1	cytidine and dCMP deaminase domain containing 1
176792	b	oooo+oooo	COL9A3	collagen, type IX, alpha 3
176899	j	oooo--oo+	IL8	interleukin 8
176920	b	ooooo+ooo	null	null
176933	b	oooo+oooo	DSCR1L1	Down syndrome critical region gene 1-like 1
176961	g	o--------	null	null
176971	a	oo+++++++	null	null
176980	a	o++++++++	GORASP1	golgi reassembly stacking protein 1, 65kDa
176983	a	oooooo+++	HES1	hairy and enhancer of split 1, (Drosophila)
176992	a	ooo++++++	ZBTB38	zinc finger and BTB domain containing 38
177043	h	ooooooo-o	ALG9	asparagine-linked glycosylation 9 homolog (yeast, alpha 1,2 mannosyltransferase)
177080	a	oo+++++++	ADSSL1	adenylosuccinate synthase like 1
177086	c	ooo++o+++	TSPYL2	TSPY-like 2
177090	a	ooo++++++	RNF111	ring finger protein 111
177124	g	ooooooo--	TNFAIP8L1	tumor necrosis factor, alpha-induced protein 8-like 1
177141	b	ooo+ooooo	KIAA0427	KIAA0427
177147	a	ooo++++++	ZNF184	zinc finger protein 184 (Kruppel-like)
177242	g	o--------	FLJ32356	null
177244	g	o--------	OR1A2	olfactory receptor, family 1, subfamily A, member 2
177302	k	oo-+ooooo	SARS2	seryl-tRNA synthetase 2
177345	b	ooo+ooooo	null	null
177366	g	o--------	null	null
177389	a	oooooo+++	CXCL3	chemokine (C-X-C motif) ligand 3
177411	h	o-ooooooo	null	null
177412	a	ooo++++++	PHLDB2	pleckstrin homology-like domain, family B, member 2
177415	b	o+ooooooo	null	null
177445	a	oooooo+++	IER5	immediate early response 5
177449	a	oo+++++++	FLJ39534	null
177467	b	oooo+oooo	ZBTB26	zinc finger and BTB domain containing 26
177472	g	oooooooo-	KUB3	null
177482	h	o-ooooooo	LOC441056|LOC440014|DUX4C|LOC399839|LOC440015|LOC440016|LOC440017|DUX4|LOC440013|LOC401650	double homeobox, 4
177525	g	ooooooo--	NUMB	numb homolog (Drosophila)
177599	b	ooo+ooooo	HISPPD2A	Histidine acid phosphatase domain containing 2A
177609	b	ooo+++ooo	null	null
177617	a	ooooooo++	null	null
177660	a	o++++++++	null	null
177711	a	ooo++++++	null	null
177717	g	o--------	PPCS	phosphopantothenoylcysteine synthetase
177719	g	o--------	PGS1	phosphatidylglycerophosphate synthase 1
177734	g	ooooooo--	SH3PX3	SH3 and PX domain containing 3
177748	b	oooooo+oo	KIAA1040	null
177749	h	o-ooooooo	TOP1MT	topoisomerase (DNA) I, mitochondrial
177832	b	oooo++ooo	STMN3	stathmin-like 3

177837	h	o-ooooooo	C3orf10	chromosome 3 open reading frame 10
177914	a	o++++++++	HSDL2	hydroxysteroid dehydrogenase like 2
178003	b	oo+oooooo	C11orf39	chromosome 11 open reading frame 39
178009	i	o-oo-----	null	null
178010	b	ooo+ooooo	HK1	hexokinase 1
178011	b	ooo+ooooo	NOD27	null
178029	b	ooo+ooooo	CMA1	chymase 1, mast cell
178043	a	ooo++++++	PSPC1	paraspeckle component 1
178067	a	ooo++++++	FNDC3A	fibronectin type III domain containing 3A
178112	b	oooo+oooo	SCAMP5	secretory carrier membrane protein 5
178117	a	oo+++++++	null	null
178179	b	ooo+ooooo	SEMA4B	sema domain, immunoglobulin domain (Ig), transmembrane domain (TM) and short cytoplasmic domain, (semaphorin) 4B
178187	b	ooo+ooooo	KIAA0090	KIAA0090
178191	g	oooooooo-	NUMA1	nuclear mitotic apparatus protein 1
178198	b	ooo+ooooo	null	null
178221	c	oooo+oo++	KCNS2	potassium voltage-gated channel, delayed-rectifier, subfamily S, member 2
178232	b	ooo+ooooo	RECQL4	RecQ protein-like 4
178313	a	oooooo+++	ETV4	ets variant gene 4 (E1A enhancer binding protein, E1AF)
178330	a	ooo++++++	FOXA1	forkhead box A1
178480	b	oooo+oooo	null	null
178491	b	oooo+oooo	C1orf104	chromosome 1 open reading frame 104
178538	b	ooo+ooooo	HLXB9	homeobox HB9
178581	a	ooooooo++	LOC387763	null
178628	g	ooooooo--	null	null
178658	b	ooo+ooooo	KIAA1787	null
178673	b	ooo+ooooo	LOC92305	null
178692	b	ooo+ooooo	NR2C2	nuclear receptor subfamily 2, group C, member 2
178729	h	o--oooooo	NDEL1	nudE nuclear distribution gene E homolog like 1 (A. nidulans)
178772	a	o++++++++	LHX4	LIM homeobox 4
178796	b	ooooooo+o	CALML5	calmodulin-like 5
178803	b	oooooo+oo	PDLIM7	PDZ and LIM domain 7 (enigma)
178804	b	oooooo+oo	PNPLA2	patatin-like phospholipase domain containing 2
178866	a	ooooooo++	LRRC19	leucine rich repeat containing 19
178907	b	ooo+ooooo	CENTB5	centaurin, beta 5
178913	h	oooo----o	LOC440338	null
178974	g	oooo-----	WDR76	WD repeat domain 76
179018	a	oooooooo+	MYST4	MYST histone acetyltransferase (monocytic leukemia) 4
179041	b	ooo+ooooo	ZNF306	zinc finger protein 306
179099	h	oooooo-oo	CDX4	caudal type homeobox transcription factor 4
179136	f	oo++----+	null	null
179286	b	oooooo+oo	SIX2	sine oculis homeobox homolog 2 (Drosophila)
179303	i	o-ooooo--	POLA	polymerase (DNA directed), alpha
179398	h	oooooo-oo	CD69	CD69 antigen (p60, early T-cell activation antigen)
179404	a	ooo++++++	BCAR3	breast cancer anti-estrogen resistance 3
179413	h	o-ooooooo	null	null
179538	g	o--------	TIMP3	TIMP metallopeptidase inhibitor 3 (Sorsby fundus dystrophy, pseudoinflammatory)
179647	a	ooo++++++	ZNF597	zinc finger protein 597
179656	b	ooo+ooooo	NPNT	nephronectin
179668	a	ooo++++++	C11orf30	chromosome 11 open reading frame 30
179700	b	oooooo+oo	SLC1A4	solute carrier family 1 (glutamate/neutral amino acid transporter), member 4
179739	g	ooooo----	ANKRD5	ankyrin repeat domain 5
179827	a	ooo++++++	ZFP36	zinc finger protein 36, C3H type, homolog (mouse)
179834	c	ooo+oo+++	PVRL3	poliovirus receptor-related 3
179870	g	o--------	SFXN3	sideroflexin 3
179876	b	oooo+oooo	ZSCAN4	zinc finger and SCAN domain containing 4
179892	b	oooo+oooo	ADAMTS17	ADAM metallopeptidase with thrombospondin type 1 motif, 17
179900	b	oooo+oooo	null	null
179906	h	oooo----o	MSR1	macrophage scavenger receptor 1
179965	a	oo+++++++	ACBD4	acyl-Coenzyme A binding domain containing 4
179985	i	o-----o--	RTN2	reticulon 2
180019	b	o+ooooooo	null	null
180037	b	ooooo+ooo	AIF1	allograft inflammatory factor 1
180058	b	ooo+++++o	PDGFRB	platelet-derived growth factor receptor, beta polypeptide
180118	c	oo++oooo+	PRKCBP1	protein kinase C binding protein 1
180184	j	oooo----+	GMFG	glia maturation factor, gamma
180185	h	o-ooooooo	TMEM69	transmembrane protein 69
180186	a	ooo++++++	MIS12	MIS12 homolog (yeast)
180191	c	ooo++o+++	TRIM23	tripartite motif-containing 23
180197	b	ooooooo+o	SUSD3	sushi domain containing 3

180243	b	ooooo+ooo	LCE3E	late cornified envelope 3E
180244	a	oooooooo+	ETV7	ets variant gene 7 (TEL2 oncogene)
180254	h	oo-oooooo	ZNF140	zinc finger protein 140 (clone pHZ-39)
180327	b	ooo+ooooo	LOC285331	null
180347	b	ooo+ooooo	C13orf8	chromosome 13 open reading frame 8
180360	a	oooooooo+	LOC145780	null
180395	c	oo++oooo+	TGFA	transforming growth factor, alpha
180412	b	oooooo+oo	SULT1A1	sulfotransferase family, cytosolic, 1A, phenol-preferring, member 1
180470	b	ooo+ooooo	null	null
180484	b	ooo+ooooo	null	null
180503	a	ooooooo++	CCR5	chemokine (C-C motif) receptor 5
180523	g	ooo------	null	null
180649	g	oooooooo-	C16orf24	chromosome 16 open reading frame 24
180686	g	oooooooo-	HMGCLL1	3-hydroxymethyl-3-methylglutaryl-Coenzyme A lyase-like 1
180764	b	oooo+oooo	H2AFY2	H2A histone family, member Y2
180823	a	oo+++++++	C18orf43	chromosome 18 open reading frame 43
180829	h	o-ooooooo	PMM1	phosphomannomutase 1
180862	h	oooooo-oo	ZIM2|PEG3	zinc finger, imprinted 2|paternally expressed 3
180896	c	ooo+o++++	KIAA0286	null
180949	b	ooo+ooooo	null	null
180957	a	oooooo+++	NEU1	sialidase 1 (lysosomal sialidase)
180992	b	oooooo+oo	SF3A1	splicing factor 3a, subunit 1, 120kDa
180998	a	ooo++++++	SLC2A3	solute carrier family 2 (facilitated glucose transporter), member 3
181039	g	o--------	IGLJ3	immunoglobulin lambda joining 3
181049	a	ooooooo++	null	null
181095	g	o--------	LOC392546	null
181116	h	oooooo-oo	LOC440352	null
181162	b	ooo+ooooo	CES7	carboxylesterase 7
181169	b	oooooo+oo	OR2C1	olfactory receptor, family 2, subfamily C, member 1
181235	g	oooooooo-	null	null
181244	b	ooo++++oo	FLJ13646	null
181281	b	ooo+ooooo	null	null
181287	b	oooo++ooo	OR3A3	olfactory receptor, family 3, subfamily A, member 3
181294	h	o-ooooooo	FLJ10652	null
181304	h	o-ooooooo	H2AFJ	H2A histone family, member J
181312	b	ooo+ooooo	QTRTD1	queuine tRNA-ribosyltransferase domain containing 1
181315	g	o--------	MAGEA10	melanoma antigen family A, 10
181496	b	ooooooo+o	null	null
181500	a	o++++++++	IPO7	importin 7
181510	b	ooo+ooooo	YTHDC2	YTH domain containing 2
181594	b	ooooo+ooo	FLJ14816	null
181608	b	ooo++oooo	CORIN	corin, serine peptidase
181617	b	ooo+ooooo	CREBL2	cAMP responsive element binding protein-like 2
181628	b	ooo+ooooo	DRCTNNB1A	null
181632	a	oooooooo+	C9orf71	chromosome 9 open reading frame 71
181639	b	ooo+++ooo	ASAH3L	N-acylsphingosine amidohydrolase 3-like
181682	h	o-ooooooo	null	null
181683	a	ooo++++++	C14orf162	chromosome 14 open reading frame 162
181685	g	ooooo----	ASPM	asp (abnormal spindle)-like, microcephaly associated (Drosophila)
181713	f	oo++----+	COL13A1	collagen, type XIII, alpha 1
181719	g	o--------	MYL9	myosin, light polypeptide 9, regulatory
181731	h	oooooo-oo	PPY	pancreatic polypeptide
181793	b	oooooo+oo	IMPDH1	IMP (inosine monophosphate) dehydrogenase 1
181804	g	oo-------	LOC342934	null
181815	b	ooo+ooooo	C18orf58	chromosome 18 open reading frame 58
181820	f	oo++----+	DCAMKL3	doublecortin and CaM kinase-like 3
181868	b	ooo+ooooo	RGNEF	null
181889	b	ooooo+ooo	null	null
181922	b	oooooo+oo	SNX15	sorting nexin 15
181923	g	o--------	null	null
181930	b	ooo+ooooo	ZNF18	zinc finger protein 18 (KOX 11)
181935	a	ooooo++++	HPD	4-hydroxyphenylpyruvate dioxygenase
181937	b	ooo+ooooo	TXNDC13	thioredoxin domain containing 13
181957	b	ooo+ooooo	CHCHD7	coiled-coil-helix-coiled-coil-helix domain containing 7
181976	d	ooo+oooo-	COL5A3	collagen, type V, alpha 3
182019	b	ooo+ooooo	FLJ10357	null
182066	a	ooo++++++	LOC344887	null
182080	c	ooo++o+++	ET	null
182081	a	ooo++++++	CYP26B1	cytochrome P450, family 26, subfamily B, polypeptide 1
182096	b	ooo+ooooo	ZNF395	zinc finger protein 395
182113	b	ooo+ooooo	COG1	component of oligomeric golgi complex 1

182118	b	ooo+ooooo	FHOD1	formin homology 2 domain containing 1
182166	a	oo+++++++	TCEA2	transcription elongation factor A (SII), 2
182167	g	oooo-----	null	null
182184	a	ooo++++++	ARIH1	ariadne homolog, ubiquitin-conjugating enzyme E2 binding protein, 1 (Drosophila)
182228	a	ooo++++++	GATA2	GATA binding protein 2
182294	b	oooooo+oo	CACNA1C	calcium channel, voltage-dependent, L type, alpha 1C subunit
182305	i	oooo-o---	MT1A	metallothionein 1A (functional)
182320	b	ooo+ooooo	P2RXL1	purinergic receptor P2X-like 1, orphan receptor
182321	a	oooooo+++	LOC388657	null
182363	g	oooooooo-	COPE	coatomer protein complex, subunit epsilon
182388	b	oo+oooooo	null	null
182404	a	oooo+++++	GDF15	growth differentiation factor 15
182408	h	o-ooooooo	FANCM	Fanconi anemia, complementation group M
182417	a	ooo++++++	DUSP1	dual specificity phosphatase 1
182449	g	ooooooo--	HSPA5BP1	heat shock 70kDa protein 5 (glucose-regulated protein, 78kDa) binding protein 1
182466	b	ooo+ooooo	ADCY9	adenylate cyclase 9
182482	a	oo+++++++	P4HA2	procollagen-proline, 2-oxoglutarate 4-dioxygenase (proline 4-hydroxylase), alpha polypeptide II
182496	h	o---ooooo	LOC441515	null
182504	b	ooo+ooooo	null	null
182561	a	o++++++++	HOM-TES-103	null
182585	b	o++oooooo	C6orf118	chromosome 6 open reading frame 118
182589	a	oo+++++++	ANKRD15	ankyrin repeat domain 15
182618	b	ooo+ooooo	null	null
182619	b	ooooo+ooo	null	null
182648	b	oooooo+oo	BCL2L1	BCL2-like 1
182656	h	oooo----o	null	null
182662	b	ooo+ooooo	RP11-535K18.3	null
182715	g	o--------	KIAA1199	KIAA1199
182763	b	ooo+ooooo	TMEM62	transmembrane protein 62
182799	a	oo+++++++	ACTR8	ARP8 actin-related protein 8 homolog (yeast)
182853	b	ooo+ooooo	ASCIZ	null
182885	b	ooo+ooooo	null	null
182902	b	ooo+ooooo	PIP5K1C	phosphatidylinositol-4-phosphate 5-kinase, type I, gamma
182927	b	ooo+ooooo	null	null
182988	b	ooo+ooooo	RFX2	regulatory factor X, 2 (influences HLA class II expression)
183009	a	oo+++++++	LOC23117|LOC440345|LOC641298	null
183031	b	oooo+oooo	null	null
183052	b	ooo+ooooo	PAQR6	progestin and adipoQ receptor family member VI
183120	c	ooo+++o++	null	null
183122	h	oooo---oo	AFARP1	null
183134	a	ooo++++++	STX16	syntaxin 16
183157	a	o++++++++	EFNA1	ephrin-A1
183158	a	ooo++++++	PHF15	PHD finger protein 15
183176	b	ooo++++oo	SPAG9	sperm associated antigen 9
183181	f	oo++----+	null	null
183231	g	oooo-----	RPL36	ribosomal protein L36
183248	b	oooooo+oo	ZNF238	zinc finger protein 238
183260	b	ooooo++oo	null	null
183303	a	ooo++++++	null	null
183304	b	ooo+ooooo	NHLRC2	NHL repeat containing 2
183313	b	ooo+ooooo	FLJ10379	null
183323	i	o-----o--	CFTR	cystic fibrosis transmembrane conductance regulator, ATP-binding cassette (sub-family C, member 7)
183410	c	oooo+oo++	FLJ35696	null
183413	a	oo+++++++	MGC50559	null
183473	a	oo+++++++	C18orf25	chromosome 18 open reading frame 25
183511	h	o-ooooooo	PTP4A2	protein tyrosine phosphatase type IVA, member 2
183553	a	oo+++++++	null	null
183568	a	ooo++++++	MDC1	mediator of DNA damage checkpoint 1
183571	l	oooo-+---	BAHCC1	BAH domain and coiled-coil containing 1
183590	b	oo+oooooo	RGAG1	retrotransposon gag domain containing 1
183615	a	oooooo+++	SERTAD1	SERTA domain containing 1
183635	a	ooo++++++	RAB6IP1	RAB6 interacting protein 1
183659	i	o-----o--	ZNF703	zinc finger protein 703
183705	g	oooooo---	FCHSD2	FCH and double SH3 domains 2
183726	g	ooooo----	CENPF	centromere protein F, 350/400ka (mitosin)
183727	h	o-ooooooo	FBXL11	F-box and leucine-rich repeat protein 11
183778	a	oooooo+++	SLC25A25	solute carrier family 25 (mitochondrial carrier; phosphate carrier), member 25
183785	b	oooo+oooo	C1QL3	complement component 1, q subcomponent-like 3

183791	b	oooo+oooo	C9orf28	chromosome 9 open reading frame 28
183803	g	o--------	null	null
183828	b	oooo+oooo	LIN7A	lin-7 homolog A (C. elegans)
183856	a	ooo++++++	OASL	2'-5'-oligoadenylate synthetase-like
183866	b	ooo+ooooo	DDB1	damage-specific DNA binding protein 1, 127kDa
183867	a	oo+++++++	ATF4	activating transcription factor 4 (tax-responsive enhancer element B67)
183932	a	oooooooo+	MYLK2	myosin light chain kinase 2, skeletal muscle
183976	b	ooo+++++o	EME1	essential meiotic endonuclease 1 homolog 1 (S. pombe)
183980	f	oo++----+	OR3A1	olfactory receptor, family 3, subfamily A, member 1
184006	b	ooo+++++o	KLHL3	kelch-like 3 (Drosophila)
184034	b	ooo+ooooo	AMOTL1	angiomotin like 1
184048	a	o++++++++	TMEM63A	transmembrane protein 63A
184063	h	o-------o	SDK1	sidekick homolog 1 (chicken)
184102	g	oooooooo-	ARF3	ADP-ribosylation factor 3
184103	b	ooo+ooooo	ADCY7	adenylate cyclase 7
184105	i	o------o-	null	null
184118	b	ooo+ooooo	HYAL1	hyaluronoglucosaminidase 1
184159	h	o-------o	NPPA	natriuretic peptide precursor A
184174	h	o-ooooooo	ALDH6A1	aldehyde dehydrogenase 6 family, member A1
184177	b	oooooo++o	OSBPL6	oxysterol binding protein-like 6
184183	b	oooo+oooo	null	null
184221	c	ooo+oo+++	HRH1	histamine receptor H1
184272	b	ooooo+ooo	CNN1	calponin 1, basic, smooth muscle
184281	c	oooo+oo++	HIST1H3D	histone 1, H3d
184429	b	ooooo+ooo	TLX1	T-cell leukemia homeobox 1
184443	h	oooooo-oo	PRSS36	protease, serine, 36
184453	a	o++++++++	AMH	anti-Mullerian hormone
184497	h	oooo----o	C1orf168	chromosome 1 open reading frame 168
184519	a	ooooooo++	ZNF38	zinc finger protein 38
184567	a	ooooooo++	LRRN3	leucine rich repeat neuronal 3
184582	b	ooooo+ooo	R3HDML	R3H domain containing-like
184605	b	ooo+ooooo	C3orf32	chromosome 3 open reading frame 32
184635	b	ooooooo+o	MGC33367	null
184636	b	ooo+ooooo	IFT88	intraflagellar transport 88 homolog (Chlamydomonas)
184665	h	oooo----o	C6orf141	chromosome 6 open reading frame 141
184754	g	oo-------	PIWIL1	piwi-like 1 (Drosophila)
184769	b	ooo+ooooo	MGC16384	null
184792	a	oo+++++++	null	null
184793	g	oooo-----	CD9	CD9 antigen (p24)
184838	a	ooo++++++	TIPARP	TCDD-inducible poly(ADP-ribose) polymerase
184881	b	ooo+ooooo	GK	glycerol kinase
184936	g	ooooooo--	FADS2	fatty acid desaturase 2
184960	b	ooo+ooooo	null	null
184984	g	oooooooo-	JAG2	jagged 2
185137	h	o-ooooooo	NTN4	netrin 4
185166	c	ooo+o++++	ACSL1	acyl-CoA synthetase long-chain family member 1
185199	b	ooo+ooooo	COG2	component of oligomeric golgi complex 2
185249	b	ooo+ooooo	ERCC5	excision repair cross-complementing rodent repair deficiency, complementation group 5 (xeroderma pigmentosum, complementation group G (Cockayne syndrome))
185294	g	ooooo----	CALML4	calmodulin-like 4
185312	c	ooo+o++++	NUAK1	NUAK family, SNF1-like kinase, 1
185319	g	oooo-----	COPZ2	coatomer protein complex, subunit zeta 2
185340	a	oooooooo+	TNFRSF7	tumor necrosis factor receptor superfamily, member 7
185369	h	oooo----o	COL4A1	collagen, type IV, alpha 1
185460	a	o++++++++	CDC42BPB	CDC42 binding protein kinase beta (DMPK-like)
185493	b	ooooo+ooo	ECGF1	endothelial cell growth factor 1 (platelet-derived)
185563	b	ooooooo+o	null	null
185583	b	oooooo++o	SUSD1	sushi domain containing 1
185597	b	oooooo+oo	null	null
185658	b	ooo+ooooo	KIF3B	kinesin family member 3B
185687	a	ooo++++++	ATF3	activating transcription factor 3
185776	h	o-------o	CACNG4	calcium channel, voltage-dependent, gamma subunit 4
185788	g	oo-------	null	null
185811	b	oooooo+oo	C21orf91	chromosome 21 open reading frame 91
185821	b	oooooo+oo	TRAF1	TNF receptor-associated factor 1
185850	a	oooooooo+	CIB2	calcium and integrin binding family member 2
185855	h	oooo-oooo	PHLDA2	pleckstrin homology-like domain, family A, member 2
185885	a	oooooo+++	PIM2	pim-2 oncogene
185900	f	oo++----+	U2AF1L2	U2(RNU2) small nuclear RNA auxiliary factor 1-like 2
185969	c	ooooo+o++	NEBL	nebulette

186085	b	oooooo+oo	PAFAH1B1|YWHAE	platelet-activating factor acetylhydrolase, isoform Ib, alpha subunit 45kDa|tyrosine 3-monooxygenase/tryptophan 5-monooxygenase activation protein, epsilon polypeptide
186091	b	ooooo+ooo	PLA2G2E	phospholipase A2, group IIE
186092	a	o++++++++	APP	amyloid beta (A4) precursor protein (peptidase nexin-II, Alzheimer disease)
186114	b	ooooo+ooo	SLC38A3	solute carrier family 38, member 3
186160	b	oooooo+oo	null	null
186162	g	o--------	LOC389339	null
186191	f	oo+++---+	null	null
186253	b	oooooo+oo	IER5L	immediate early response 5-like
186259	b	oooo+oooo	null	null
186321	j	o-ooooo++	null	null
186360	a	oooooo+++	KIAA1181	null
186406	b	oooo+oooo	FZR1	fizzy/cell division cycle 20 related 1 (Drosophila)
186415	h	o-ooooooo	FLJ20628	null
186446	h	oooo----o	BM88	null
186504	b	ooo+ooooo	ARL13B	ADP-ribosylation factor-like 13B
186519	a	ooo++++++	PTPN14	protein tyrosine phosphatase, non-receptor type 14
186522	b	ooo++oooo	IQGAP3	IQ motif containing GTPase activating protein 3
186589	g	o--------	MAF	v-maf musculoaponeurotic fibrosarcoma oncogene homolog (avian)
186608	h	ooooo-ooo	MIZF	null
186616	b	oooooo+oo	null	null
186647	a	oooooooo+	GDF3	growth differentiation factor 3
186705	h	o-------o	CD80	CD80 antigen (CD28 antigen ligand 1, B7-1 antigen)
186765	i	o-----o--	C9orf74	chromosome 9 open reading frame 74
186797	b	oooo+oooo	SOX17	SRY (sex determining region Y)-box 17
186800	f	oo++----+	DCST1	DC-STAMP domain containing 1
186835	a	o++++++++	ZZZ3	zinc finger, ZZ-type containing 3
186857	b	oooooo+oo	C19orf32	chromosome 19 open reading frame 32
186894	b	ooo+ooooo	SDCCAG33	serologically defined colon cancer antigen 33
186902	b	ooo+ooooo	null	null
186922	a	ooo++++++	RNF19	ring finger protein 19
186944	b	o+ooooooo	THBS3	thrombospondin 3
186948	b	ooo+ooooo	MUC16	mucin 16
186957	a	ooo++++++	BMPR2	bone morphogenetic protein receptor, type II (serine/threonine kinase)
186960	a	oo+++++++	SLCO1C1	solute carrier organic anion transporter family, member 1C1
186965	a	ooooooo++	ZNF75	zinc finger protein 75 (D8C6)
186987	b	ooooooo+o	null	null
187002	a	oooooo+++	EBAG9	estrogen receptor binding site associated, antigen, 9
187009	a	o++++++++	RASSF1	Ras association (RalGDS/AF-6) domain family 1
187013	a	ooooooo++	null	null
187015	b	ooo+ooooo	MCF2L	MCF.2 cell line derived transforming sequence-like
187019	b	ooo++++oo	AKR1B10	aldo-keto reductase family 1, member B10 (aldose reductase)
187090	a	oo+++++++	USP19	ubiquitin specific peptidase 19
187111	b	o+ooooooo	ZFP62	zinc finger protein 62 homolog (mouse)
187128	a	oo+++++++	PAX6	paired box gene 6 (aniridia, keratitis)
187170	b	ooo+ooooo	FSHPRH1	FSH primary response (LRPR1 homolog, rat) 1
187193	c	ooo+oo+++	SLC24A4	solute carrier family 24 (sodium/potassium/calcium exchanger), member 4
187211	b	oooo+oooo	TRGC2|TRGV5|LOC442535	T cell receptor gamma constant 2|T cell receptor gamma variable 5
187222	b	oo+oooooo	LMOD2	leiomodin 2 (cardiac)
187235	b	ooo+ooooo	GBA2	glucosidase, beta (bile acid) 2
187236	c	ooo+ooo++	PSCD1	pleckstrin homology, Sec7 and coiled-coil domains 1(cytohesin 1)
187238	b	ooo+ooooo	CEACAM3	carcinoembryonic antigen-related cell adhesion molecule 3
187272	b	oooo+oooo	C21orf82	chromosome 21 open reading frame 82
187287	h	o-ooooooo	TP53AP1	TP53 activated protein 1
187321	b	ooo+ooooo	CDH2	cadherin 2, type 1, N-cadherin (neuronal)
187340	c	oo++oooo+	TBC1D12	TBC1 domain family, member 12
187360	a	o++++++++	PIGH	phosphatidylinositol glycan, class H
187415	b	ooooooo+o	null	null
187443	b	ooo+ooooo	GNRHR	gonadotropin-releasing hormone receptor
187446	b	ooo+ooooo	RALBP1	ralA binding protein 1
187470	b	ooo+ooooo	KIAA0247	KIAA0247
187482	a	oooo+++++	null	null
187487	b	ooo+ooooo	null	null
187489	g	ooooooo--	LOC201229	null
187491	b	ooo+ooooo	ZNF181	zinc finger protein 181 (HHZ181)
187521	g	o--------	MYO7B	myosin VIIB
187555	b	oooooo+oo	LOC442461	null
187603	b	ooo+ooooo	PCGF4	polycomb group ring finger 4
187607	b	ooooo++oo	null	null

187636	b	ooooooo+o	TGM4	transglutaminase 4 (prostate)
187637	a	ooooo++++	C14orf128	chromosome 14 open reading frame 128
187667	b	ooo+ooooo	CDRT4	CMT1A duplicated region transcript 4
187682	a	oo+++++++	AAAS	achalasia, adrenocortical insufficiency, alacrimia (Allgrove, triple-A)
187746	b	ooo++oooo	MGC40168	null
187757	b	ooo+ooooo	FLJ20035	null
187766	a	oooooo+++	ABHD5	abhydrolase domain containing 5
187842	b	oooo+oooo	null	null
187860	a	ooooo++++	ZNF664	zinc finger protein 664
187876	c	ooo+oo+++	IRX6	iroquois homeobox protein 6
187953	h	ooooo-ooo	ZNF688	zinc finger protein 688
187979	h	o-ooooooo	PCCB	propionyl Coenzyme A carboxylase, beta polypeptide
187988	g	oooo-----	FUT8	fucosyltransferase 8 (alpha (1,6) fucosyltransferase)
188018	b	ooo+ooooo	TDRD7	tudor domain containing 7
188141	b	ooooo+ooo	LOC440278	null
188184	a	o++++++++	null	null
188189	b	ooo+ooooo	DSG1	desmoglein 1
188204	a	oooooooo+	LOC400573	null
188244	b	ooooooo+o	null	null
188306	b	oo+oooooo	null	null
188311	b	oooo++ooo	null	null
188335	a	ooooooo++	LOC131076	null
188340	b	oo++ooooo	ZBED4	zinc finger, BED-type containing 4
188411	b	ooooo+ooo	ANKRD11	ankyrin repeat domain 11
188434	a	o++++++++	FA2H	fatty acid 2-hydroxylase
188471	b	ooo+ooooo	FGFR1	fibroblast growth factor receptor 1 (fms-related tyrosine kinase 2, Pfeiffer syndrome)
188502	b	ooooooo+o	FLJ23514	null
188504	h	ooooooo-o	ELA2	elastase 2, neutrophil
188512	g	ooooooo--	OBSL1	obscurin-like 1
188526	b	ooo+ooooo	null	null
188528	b	ooooo+ooo	POSTN	periostin, osteoblast specific factor
188590	b	oooo+oooo	ZCCHC12	zinc finger, CCHC domain containing 12
188607	b	ooo+ooooo	PIGN	phosphatidylinositol glycan, class N
188635	h	ooooooo-o	null	null
188654	b	ooo+ooooo	MTSS1	metastasis suppressor 1
188689	b	ooo+ooooo	ARID1A	AT rich interactive domain 1A (SWI- like)
188699	b	ooo+ooooo	NPY2R	neuropeptide Y receptor Y2
188736	b	ooo++oooo	VN1R1	vomeronasal 1 receptor 1
188742	b	oooo+oooo	null	null
188749	a	ooo++++++	ZCCHC8	zinc finger, CCHC domain containing 8
188764	b	ooooo+ooo	null	null
188831	a	o++++++++	CMPK	cytidylate kinase
188838	b	ooo+ooooo	PLD5	phospholipase D family, member 5
188843	h	o-ooooooo	CDK8	cyclin-dependent kinase 8
188867	b	oooo++ooo	ABHD7	abhydrolase domain containing 7
188871	b	ooo+ooooo	PTPN13	protein tyrosine phosphatase, non-receptor type 13 (APO-1/CD95 (Fas)-associated phosphatase)
188926	b	oooo++ooo	null	null
188991	a	o++++++++	C1orf162	chromosome 1 open reading frame 162
189020	b	ooo+ooooo	null	null
189026	a	oooooooo+	LOC200772	null
189066	b	ooo+ooooo	MICAL3	microtubule associated monoxygenase, calponin and LIM domain containing 3
189068	b	oooo++ooo	FLJ39582	null
189090	b	ooooo+ooo	KLF4	Kruppel-like factor 4 (gut)
189125	b	ooo+ooooo	STS-1	null
189133	b	ooo+ooooo	KIAA0146	KIAA0146 protein
189139	a	ooo++++++	FLJ10803	null
189150	a	ooo++++++	DTL	denticleless homolog (Drosophila)
189160	h	oooooo-oo	CPOX	coproporphyrinogen oxidase
189188	g	o--------	null	null
189356	b	ooooo+++o	null	null
189401	a	ooo++++++	C14orf108	chromosome 14 open reading frame 108
189406	i	o-----o--	MGAT1	mannosyl (alpha-1,3-)-glycoprotein beta-1,2-N-acetylglucosaminyltransferase
189502	a	oo+++++++	null	null
189523	b	oo+oooooo	ICK	intestinal cell (MAK-like) kinase
189535	b	ooo+++++o	PSIP1	PC4 and SFRS1 interacting protein 1
189537	b	oooooo+oo	null	null
189588	b	ooo+ooooo	MCCC1	methylcrotonoyl-Coenzyme A carboxylase 1 (alpha)
189610	b	ooo++oooo	null	null
189624	a	ooo++++++	GTF2IRD1	GTF2I repeat domain containing 1

189678	b	ooo+ooooo	FNBP1L	formin binding protein 1-like
189702	b	oooo+oooo	null	null
189708	b	oo+++oooo	SRY	sex determining region Y
189719	b	ooo+ooooo	CEP70	centrosomal protein 70kDa
189732	b	ooo+ooooo	EIF2C3	eukaryotic translation initiation factor 2C, 3
189781	g	o--------	PRCC	papillary renal cell carcinoma (translocation-associated)
189816	b	ooo+ooooo	ABLIM1	actin binding LIM protein 1
189863	b	oooo+oooo	null	null
189905	b	ooo+ooooo	ADAT1	adenosine deaminase, tRNA-specific 1
189906	a	oooooo+++	ZNF7	zinc finger protein 7 (KOX 4, clone HF.16)
189947	b	ooooo+ooo	PMFBP1	polyamine modulated factor 1 binding protein 1
189973	f	oo++----+	OR1F1	olfactory receptor, family 1, subfamily F, member 1
189977	h	o-ooooooo	PBX1	pre-B-cell leukemia transcription factor 1
189980	b	ooooooo+o	OR2T6	olfactory receptor, family 2, subfamily T, member 6
189995	b	ooooooo+o	GPR82	G protein-coupled receptor 82
190013	c	ooo++o+++	ZNF586	zinc finger protein 586
190014	b	ooooo+ooo	SFRP2	secreted frizzled-related protein 2
190129	h	oooo----o	SLC35F4	solute carrier family 35, member F4
190176	b	ooo+ooooo	DKFZP434B0335	null
190256	h	oooo----o	TMPRSS11A	transmembrane protease, serine 11A
190265	b	ooo+ooooo	FLJ16478	null
190319	b	oooo+oooo	null	null
190367	b	oooooo++o	PLXNB3|STK23	plexin B3|serine/threonine kinase 23
190380	a	ooo++++++	SEC61A2	Sec61 alpha 2 subunit (S. cerevisiae)
190390	h	o------oo	HSPG2	heparan sulfate proteoglycan 2 (perlecan)
190444	h	o-ooooooo	RNF126	ring finger protein 126
190448	b	oooo+oooo	SCRT1	scratch homolog 1, zinc finger protein (Drosophila)
190510	b	ooo+++ooo	null	null
190575	a	oooooo+++	LOC126295	null
190586	h	oooo----o	null	null
190637	i	ooooo-o--	RAI1	retinoic acid induced 1
190667	b	oooooo++o	RP3-366L4.2	null
190669	a	oooooo+++	KTI12	KTI12 homolog, chromatin associated (S. cerevisiae)
190731	h	oooooo-oo	LOC158345	null
190749	g	oooooooo-	LOC390488	null
190831	g	o--------	VKORC1L1	vitamin K epoxide reductase complex, subunit 1-like 1
190833	a	ooo++++++	HSPBAP1	HSPB (heat shock 27kDa) associated protein 1
190836	b	ooooooo+o	KRTAP19-3	keratin associated protein 19-3
190902	b	ooo+ooooo	ARFGEF1	ADP-ribosylation factor guanine nucleotide-exchange factor 1(brefeldin A-inhibited)
190918	i	oooo-o---	NIFUN	NifU-like N-terminal domain containing
190921	g	o--------	BCAS2|DENND2C	breast carcinoma amplified sequence 2|DENN/MADD domain containing 2C
190925	b	ooo+ooooo	C17orf41	chromosome 17 open reading frame 41
190932	b	ooooo+ooo	COL21A1	collagen, type XXI, alpha 1
190955	b	ooo+ooooo	null	null
191038	b	ooooooo+o	null	null
191090	a	ooooooo++	C3orf40	chromosome 3 open reading frame 40
191100	g	oo-------	INCENP	inner centromere protein antigens 135/155kDa
191116	a	ooo++++++	EIF4A2	eukaryotic translation initiation factor 4A, isoform 2
191122	b	ooo+ooooo	TAF5	TAF5 RNA polymerase II, TATA box binding protein (TBP)-associated factor, 100kDa
191139	b	oooo+oooo	OR2L2	olfactory receptor, family 2, subfamily L, member 2
191246	b	ooo++oooo	GPR113	G protein-coupled receptor 113
191263	g	o--------	CTCFL	CCCTC-binding factor (zinc finger protein)-like
191272	b	ooooooo+o	ZNF548	zinc finger protein 548
191331	b	oooo+oooo	null	null
191337	a	oooooo+++	CNIH3	cornichon homolog 3 (Drosophila)
191355	h	o-ooooooo	PLEKHB2	pleckstrin homology domain containing, family B (evectins) member 2
191405	b	ooooo+ooo	UPK1B	uroplakin 1B
191410	f	oo++----+	BHMT2	betaine-homocysteine methyltransferase 2
191434	h	o-------o	CLEC7A	C-type lectin domain family 7, member A
191441	b	ooooooo+o	RSAD2	radical S-adenosyl methionine domain containing 2
191463	b	ooo+ooooo	TPCN2	two pore segment channel 2
191495	g	o--------	LOC387770	null
191516	b	ooo+ooooo	IMP-1	null
191526	b	ooo+ooooo	TEP1	telomerase-associated protein 1
191539	a	ooo++++++	RAF1	v-raf-1 murine leukemia viral oncogene homolog 1
191543	b	ooo+ooooo	ANTXR2	anthrax toxin receptor 2
191545	g	o--------	SUPT7L	suppressor of Ty 7 (S. cerevisiae)-like
191606	b	oooo+oooo	LRRC7	leucine rich repeat containing 7
191615	b	ooo+ooooo	ZMYM6	zinc finger, MYM-type 6

191650	b	oooooo+oo	null	null
191693	b	ooo+ooooo	SLC41A2	solute carrier family 41, member 2
191716	h	oooo---oo	null	null
191777	b	oooo+oooo	null	null
191794	a	ooo++++++	IRF2BP2	interferon regulatory factor 2 binding protein 2
191821	a	ooo++++++	CXCR4	chemokine (C-X-C motif) receptor 4
191823	h	oooooo-oo	null	null
191839	a	oo+++++++	LOC375251	null
191842	a	ooooooo++	P8	null
191881	h	o-------o	null	null
191909	b	ooo+ooooo	PPM1K	protein phosphatase 1K (PP2C domain containing)
191921	b	ooooo+ooo	SCD5	stearoyl-CoA desaturase 5
191932	g	o--------	CCDC36	coiled-coil domain containing 36
191935	b	ooo+ooooo	MDFIC	MyoD family inhibitor domain containing
191960	b	ooooo+ooo	CD8A	CD8 antigen, alpha polypeptide (p32)
191962	a	oo+++++++	FKBP11	FK506 binding protein 11, 19 kDa
192079	b	ooo+ooooo	ARHGEF7	Rho guanine nucleotide exchange factor (GEF) 7
192110	b	ooo+ooooo	FAM81A	family with sequence similarity 81, member A
192115	f	oooooo+-+	TNIK	TRAF2 and NCK interacting kinase
192117	b	ooo+ooooo	PIK3R4	phosphoinositide-3-kinase, regulatory subunit 4, p150
192163	a	ooo++++++	null	null
192178	b	ooo+ooooo	GRTP1	growth hormone regulated TBC protein 1
192193	b	ooo+ooooo	APPBP2	amyloid beta precursor protein (cytoplasmic tail) binding protein 2
192239	h	o-ooooooo	PPARG	peroxisome proliferative activated receptor, gamma
192252	b	ooo+ooooo	IFT140	intraflagellar transport 140 homolog (Chlamydomonas)
192258	a	oooooo+++	GADD45A	growth arrest and DNA-damage-inducible, alpha
192318	a	oooooooo+	MAGEB10	melanoma antigen family B, 10
192359	c	ooo+oo+++	RSBN1	round spermatid basic protein 1
192367	a	oooooooo+	STK35	serine/threonine kinase 35
192376	b	oo+oooooo	null	null
192479	l	o--+-----	KIAA0528	null
192507	b	oooo+oooo	GEM	GTP binding protein overexpressed in skeletal muscle
192512	g	o--------	OR4K14	olfactory receptor, family 4, subfamily K, member 14
192539	b	ooo+ooooo	SRGAP1	SLIT-ROBO Rho GTPase activating protein 1
192564	a	oo+++++++	ZNF621	zinc finger protein 621
192614	h	o-ooooooo	RBBP6	retinoblastoma binding protein 6
192654	g	o--------	SSBP3	single stranded DNA binding protein 3
192656	a	oooooooo+	MGAT5	mannosyl (alpha-1,6-)-glycoprotein beta-1,6-N-acetyl-glucosaminyltransferase
192657	b	o++oooooo	null	null
192658	h	o-ooooooo	USF1	upstream transcription factor 1
192662	b	ooo+ooooo	OR52N4	olfactory receptor, family 52, subfamily N, member 4
192711	b	o+ooooooo	null	null
192720	a	ooo++++++	PNRC2	proline-rich nuclear receptor coactivator 2
192742	a	oooooooo+	null	null
192760	a	ooooo++++	null	null
192761	g	o--------	BPY2B|BPY2|BPY2C	basic charge, Y-linked, 2B|basic charge, Y-linked, 2|basic charge, Y-linked, 2C
192795	a	oooooo+++	LOC91614	null
192829	b	ooooo+++o	null	null
192861	b	oo+oooooo	LOC389023	null
192874	a	ooo++++++	KCNK5	potassium channel, subfamily K, member 5
192895	a	oo+++++++	null	null
192905	b	ooo+ooooo	COG5	component of oligomeric golgi complex 5
192916	b	ooo+ooooo	KHSRP	KH-type splicing regulatory protein (FUSE binding protein 2)
192928	b	oooooo++o	AD7C-NTP	null
192950	a	ooo++++++	BRWD1	bromodomain and WD repeat domain containing 1
192962	b	ooo+ooooo	MYCPBP	c-myc promoter binding protein
192975	g	ooo------	GAJ	null
192976	h	oooo----o	PYHIN1	pyrin and HIN domain family, member 1
192990	b	o+ooooooo	LOC147645	null
193009	g	oo-------	null	null
193038	g	oooooooo-	null	null
193055	a	oo+++++++	C9	complement component 9
193067	b	ooo+ooooo	TESK2	testis-specific kinase 2
193083	h	o-ooooooo	ADRA2B	adrenergic, alpha-2B-, receptor
193149	b	ooo+ooooo	KIAA1546	null
193172	a	ooooooo++	HIST1H3G	histone 1, H3g
193196	a	oo+++++++	SIRT7	sirtuin (silent mating type information regulation 2 homolog) 7 (S. cerevisiae)
193261	b	oooooo+oo	FLJ33915	null
193266	b	ooo+ooooo	CXXC4	CXXC finger 4
193293	g	ooooo----	null	null

193415	i	oo----o--	CD1E	CD1E antigen, e polypeptide
193427	b	oooo++++o	null	null
193511	b	oooooo+oo	XDH	xanthine dehydrogenase
193514	b	oooooo+oo	null	null
193576	b	ooo+ooooo	LLGL2	lethal giant larvae homolog 2 (Drosophila)
193585	b	oooo+oooo	ITGB2	integrin, beta 2 (antigen CD18 (p95), lymphocyte function-associated antigen 1; macrophage antigen 1 (mac-1) beta subunit)
193615	a	oooo+++++	FAM46A	family with sequence similarity 46, member A
193629	b	ooo+ooooo	AHI1	Abelson helper integration site 1
193657	g	o--------	FADS3	fatty acid desaturase 3
193665	a	oooooo+++	MED18	mediator of RNA polymerase II transcription, subunit 18 homolog (yeast)
193672	b	oooo+oooo	null	null
193689	j	o-oooo+++	BMP2	bone morphogenetic protein 2
193717	b	ooo+ooooo	ATRN	attractin
193743	c	oo++oooo+	CCRL1	chemokine (C-C motif) receptor-like 1
193759	i	o--o-----	TNKS1BP1	tankyrase 1 binding protein 1, 182kDa
193795	g	o--------	ATP1A2	ATPase, Na+/K+ transporting, alpha 2 (+) polypeptide
193865	g	oo-------	KIR2DS2	killer cell immunoglobulin-like receptor, two domains, short cytoplasmic tail, 2
193959	b	oooo++ooo	ZNF519	zinc finger protein 519
193986	b	ooo+ooooo	STRA6	stimulated by retinoic acid gene 6 homolog (mouse)
194023	c	oo++oooo+	null	null
194024	d	ooo+ooo--	PIAS3	protein inhibitor of activated STAT, 3
194026	a	ooo++++++	C9orf150	chromosome 9 open reading frame 150
194041	a	ooo++++++	TCF12	transcription factor 12 (HTF4, helix-loop-helix transcription factors 4)
194045	g	o--------	B4GALT2	UDP-Gal:betaGlcNAc beta 1,4- galactosyltransferase, polypeptide 2
194114	h	o-ooooooo	FOXO3A	forkhead box O3A
194168	b	ooooooo+o	PRH2	proline-rich protein HaeIII subfamily 2
194196	b	ooo+ooooo	PIP5K2C	phosphatidylinositol-4-phosphate 5-kinase, type II, gamma
194212	a	ooo++++++	ZNF443	zinc finger protein 443
194214	b	ooo+ooooo	MPP7	membrane protein, palmitoylated 7 (MAGUK p55 subfamily member 7)
194223	b	oooo+oooo	null	null
194271	h	ooo-ooooo	TMPRSS2	transmembrane protease, serine 2
194307	b	ooooooo+o	FHAD1	forkhead-associated (FHA) phosphopeptide binding domain 1
194314	b	ooo+ooooo	ABCC4	ATP-binding cassette, sub-family C (CFTR/MRP), member 4
194339	f	oo++----+	FLJ10986	null
194353	b	ooo+++ooo	ZNF75	zinc finger protein 75 (D8C6)
194365	b	ooo+ooooo	PER3	period homolog 3 (Drosophila)
194445	b	ooooooo+o	null	null
194488	a	ooo++++++	VAPB	VAMP (vesicle-associated membrane protein)-associated protein B and C
194493	g	ooo------	FAIM	Fas apoptotic inhibitory molecule
194494	b	ooo+ooooo	LOC441570	null
194498	g	ooooo----	DLG7	discs, large homolog 7 (Drosophila)
194528	a	oooooooo+	TRIM5	tripartite motif-containing 5
194565	b	ooo+ooooo	WDR5B	WD repeat domain 5B
194633	b	ooo+ooooo	ARMC8	armadillo repeat containing 8
194647	f	oo++----+	C19orf9	chromosome 19 open reading frame 9
194654	a	oo+++++++	TRIM23	tripartite motif-containing 23
194691	a	oo+++++++	LOC93622	null
194737	b	ooo+ooooo	KIAA1001	null
194779	b	ooo+ooooo	KIAA0913	KIAA0913
194788	a	oooooo+++	null	null
194821	k	o--+ooooo	APAF1	apoptotic peptidase activating factor
194873	a	oo+++++++	HDC	histidine decarboxylase
194890	b	ooo+ooooo	RIPK5	receptor interacting protein kinase 5
194900	h	ooooo-ooo	KIAA1838	KIAA1838
194945	b	ooo+ooooo	EXTL2	exostoses (multiple)-like 2
194955	b	ooooo++oo	ABCA4	ATP-binding cassette, sub-family A (ABC1), member 4
195002	a	oooo+++++	CA8	carbonic anhydrase VIII
195004	a	o++++++++	DLEU2	deleted in lymphocytic leukemia, 2
195039	c	oo++oooo+	SSX3	synovial sarcoma, X breakpoint 3
195072	g	oo-------	COL25A1	collagen, type XXV, alpha 1
195105	b	ooo+ooooo	OTUD4	OTU domain containing 4
195141	a	ooo++++++	LOC222701	null
195167	h	o-ooooooo	TMEM49	transmembrane protein 49
195304	b	ooo+ooooo	DKFZp762I137	null
195457	b	ooooo+ooo	EMR1	egf-like module containing, mucin-like, hormone receptor-like 1
195497	c	ooo+oo+++	FLJ10707	null
195499	a	oooooo+++	FOXC1	forkhead box C1
195525	b	ooo+ooooo	PRDM4	PR domain containing 4
195535	b	ooo+ooooo	PLXNA2	plexin A2
195553	b	ooo+ooooo	ZNF318	zinc finger protein 318

195748	b	oooooo+oo	LOC147468	null
195751	h	ooooo-ooo	C17orf63	chromosome 17 open reading frame 63
195756	b	ooo++++oo	DKFZp434H2215	null
195806	g	o--------	ZNF483	zinc finger protein 483
195867	g	o--------	SLC17A3	solute carrier family 17 (sodium phosphate), member 3
195887	b	oooo++ooo	ITGA8	integrin, alpha 8
195923	i	o-----o--	LOC440008	null
195953	b	ooo+ooooo	USH1C	Usher syndrome 1C (autosomal recessive, severe)
195957	b	ooo+ooooo	KIAA0922	null
195997	b	ooooooo+o	C3AR1	complement component 3a receptor 1
196037	b	ooooooo+o	ACF	null
196119	a	o++++++++	LOC338758	null
196129	b	oooooo++o	LOC284001	null
196141	a	o++++++++	CD14	CD14 antigen
196146	b	ooo+ooooo	null	null
196168	b	ooo+ooooo	MYO5A	myosin VA (heavy polypeptide 12, myoxin)
196221	b	ooo+ooooo	ARL15	ADP-ribosylation factor-like 15
196248	b	ooooo+ooo	FLJ00060	null
196281	b	o+++ooooo	SNX10	sorting nexin 10
196286	g	oooooooo-	GML	GPI anchored molecule like protein
196289	b	oo+oooooo	null	null
196297	b	ooo+ooooo	PKD1	polycystic kidney disease 1 (autosomal dominant)
196303	b	ooo+ooooo	GOLGA1	golgi autoantigen, golgin subfamily a, 1
196304	b	ooo+ooooo	NFXL1	nuclear transcription factor, X-box binding-like 1
196316	b	oooo+oooo	SULT2A1	sulfotransferase family, cytosolic, 2A, dehydroepiandrosterone (DHEA)-preferring, member 1
196342	c	o+++++o++	LOC56964	null
196385	a	o++++++++	C3orf17	chromosome 3 open reading frame 17
196397	b	oooo+oooo	RNF32	ring finger protein 32
196426	b	ooo+ooooo	REV3L	REV3-like, catalytic subunit of DNA polymerase zeta (yeast)
196524	h	o-ooooooo	WDSOF1	WD repeats and SOF1 domain containing
196532	b	ooo+ooooo	MAGED2	melanoma antigen family D, 2
196533	b	ooo+ooooo	LAMP1	lysosomal-associated membrane protein 1
196546	g	o--------	CNTN3	contactin 3 (plasmacytoma associated)
196556	b	ooooooo+o	FRS3	fibroblast growth factor receptor substrate 3
196578	b	oooooo+oo	PAK7	p21(CDKN1A)-activated kinase 7
196624	b	ooooo+ooo	null	null
196649	i	o---o----	null	null
196657	b	oooo+oooo	TMLHE	trimethyllysine hydroxylase, epsilon
196687	a	ooo++++++	NCOA7	nuclear receptor coactivator 7
196692	b	ooo+ooooo	WDR32	WD repeat domain 32
196695	a	oo+++++++	IER3	immediate early response 3
196735	g	ooooooo--	ProSAPiP1	null
196742	a	ooooooo++	null	null
196744	g	oooooooo-	SCML4	sex comb on midleg-like 4 (Drosophila)
196770	h	o-------o	FOXD2	forkhead box D2
196792	a	oo+++++++	ENTPD5	ectonucleoside triphosphate diphosphohydrolase 5
196795	b	ooooo+ooo	NR5A1	nuclear receptor subfamily 5, group A, member 1
196805	a	ooooooo++	null	null
196875	b	ooo+ooooo	RINT-1	null
196925	b	ooo+ooooo	NCKIPSD	NCK interacting protein with SH3 domain
196957	c	ooo+oo+++	YAP1	Yes-associated protein 1, 65kDa
197053	g	ooo------	null	null
197109	b	ooo+ooooo	TRIM4	tripartite motif-containing 4
197227	a	oooooo+++	KRTHA4	keratin, hair, acidic, 4
197230	b	ooo+ooooo	CD24	CD24 antigen (small cell lung carcinoma cluster 4 antigen)
197251	h	o--oooooo	SLC1A5	solute carrier family 1 (neutral amino acid transporter), member 5
197253	b	ooo+ooooo	LEPRE1	leucine proline-enriched proteoglycan (leprecan) 1
197266	h	ooooooo-o	FZR1	fizzy/cell division cycle 20 related 1 (Drosophila)
197310	a	oo+++++++	IFRD1	interferon-related developmental regulator 1
197326	a	ooooooo++	ARL4D	ADP-ribosylation factor-like 4D
197340	a	oo+++++++	UBC	ubiquitin C
197348	i	o-oo-----	null	null
197353	a	oooo+++++	MME	membrane metallo-endopeptidase (neutral endopeptidase, enkephalinase, CALLA, CD10)
197381	b	ooo+ooooo	C4orf8	chromosome 4 open reading frame 8
197392	b	ooo+ooooo	null	null
197400	f	oo++----+	null	null
197406	b	oooooo+oo	TBC1D19	TBC1 domain family, member 19
197423	b	ooo+ooooo	MUT	methylmalonyl Coenzyme A mutase
197450	a	oooooooo+	LBX2	ladybird homeobox homolog 2 (Drosophila)

197453	b	oooo+oooo	DPY19L2|DPY19L2P1	dpy-19-like 2 (C. elegans)|dpy-19-like 2 pseudogene 1 (C. elegans)
197455	b	oooo+oooo	LOC283514	null
197485	b	ooooooo+o	TRPC5	transient receptor potential cation channel, subfamily C, member 5
197520	h	oooooo-oo	GPR109A	G protein-coupled receptor 109A
197538	h	o-ooooooo	BHLHB2	basic helix-loop-helix domain containing, class B, 2
197592	h	o-ooooooo	null	null
197613	a	ooooo++++	HIST2H4|H4/o	histone 2, H4
197622	b	oooooo+oo	MKRN3	makorin, ring finger protein, 3
197642	g	o--------	null	null
197668	h	o-ooooooo	PDE4A	phosphodiesterase 4A, cAMP-specific (phosphodiesterase E2 dunce homolog, Drosophila)
197684	b	ooo+ooooo	DHX8	DEAH (Asp-Glu-Ala-His) box polypeptide 8
197749	b	oooo+oooo	null	null
197751	b	ooo+ooooo	GRB10	growth factor receptor-bound protein 10
197753	b	ooo+ooooo	ABCC5	ATP-binding cassette, sub-family C (CFTR/MRP), member 5
197765	a	ooo++++++	CDYL2	chromodomain protein, Y-like 2
197797	b	ooooo+ooo	LOC284591	null
197824	a	ooo++++++	TEX14	testis expressed sequence 14
197846	a	oo+++++++	LARP4	La ribonucleoprotein domain family, member 4
197856	h	oooooo-oo	LOC339902	null
197907	b	ooo+ooooo	PLSCR4	phospholipid scramblase 4
197962	b	oooooo+oo	NOC4L	nucleolar complex associated 4 homolog (S. cerevisiae)
197996	b	ooo+ooooo	KIAA0999	null
198050	b	ooooooo+o	null	null
198092	b	oooooo+oo	null	null
198147	g	oooo-----	POLR2I	polymerase (RNA) II (DNA directed) polypeptide I, 14.5kDa
198154	a	oooooo+++	null	null
198237	c	ooo+oooo+	null	null
198298	b	oo++ooooo	NFATC3	nuclear factor of activated T-cells, cytoplasmic, calcineurin-dependent 3
198328	b	ooooo+ooo	PPP1R3A	protein phosphatase 1, regulatory (inhibitor) subunit 3A (glycogen and sarcoplasmic reticulum binding subunit, skeletal muscle)
198391	a	ooo++++++	TGFBR3	transforming growth factor, beta receptor III (betaglycan, 300kDa)
198439	a	ooo++++++	FLJ13195	null
198558	a	o++++++++	05-Mar	membrane-associated ring finger (C3HC4) 5
198574	a	o++++++++	AOC3	amine oxidase, copper containing 3 (vascular adhesion protein 1)
198575	a	o++++++++	NFKBIZ	nuclear factor of kappa light polypeptide gene enhancer in B-cells inhibitor, zeta
198593	b	ooo+ooooo	null	null
198606	c	ooo+oo+++	EMP1	epithelial membrane protein 1
198630	b	oooo+oooo	TGM5	transglutaminase 5
198638	b	oooo+oooo	null	null
198707	b	ooo++++oo	SIGLEC7	sialic acid binding Ig-like lectin 7
198718	b	oooooo+oo	RNF11	ring finger protein 11
198809	b	ooo+ooooo	SLC28A1	solute carrier family 28 (sodium-coupled nucleoside transporter), member 1
198813	b	ooo+ooooo	ACACA	acetyl-Coenzyme A carboxylase alpha
198848	c	ooo+oo+++	KIAA0460	KIAA0460
198878	b	ooo+ooooo	PRKACB	protein kinase, cAMP-dependent, catalytic, beta
198978	b	ooo++oooo	LOC254559	null
199021	g	ooooo----	null	null
199062	b	ooo+ooooo	STOX2	storkhead box 2
199067	g	oo-------	ASB12	ankyrin repeat and SOCS box-containing 12
199106	b	ooo+ooooo	C14orf101	chromosome 14 open reading frame 101
199160	a	oooo+++++	LOC389025	null
199182	a	oooooo+++	PHF23	PHD finger protein 23
199183	h	oooo-oooo	null	null
199291	g	ooooooo--	ADCY6	adenylate cyclase 6
199302	b	ooooooo+o	FAM79B	family with sequence similarity 79, member B
199343	b	ooooooo+o	KCNMA1	potassium large conductance calcium-activated channel, subfamily M, alpha member 1
199358	b	ooo+ooooo	ELOVL4	elongation of very long chain fatty acids (FEN1/Elo2, SUR4/Elo3, yeast)-like 4
199363	b	ooo+ooooo	PCDHB15	protocadherin beta 15
199368	b	ooo+ooooo	PLXNB1	plexin B1
199449	b	ooo+ooooo	GPR31	G protein-coupled receptor 31
199477	a	oooooooo+	PCDHB3	protocadherin beta 3
199492	h	oooooo-oo	FUT4	fucosyltransferase 4 (alpha (1,3) fucosyltransferase, myeloid-specific)
199498	c	ooo+oo+++	ZNF323	zinc finger protein 323
199506	b	ooo+ooooo	ANKRD40	ankyrin repeat domain 40
199511	a	oooooooo+	SOCS7	suppressor of cytokine signaling 7
199514	b	ooo+ooooo	NAG8	null
199535	g	o--------	ELK1	ELK1, member of ETS oncogene family
199589	a	ooo++++++	FER1L4	fer-1-like 4 (C. elegans)

199592	b	ooooooo+o	TGFBRAP1	transforming growth factor, beta receptor associated protein 1
199603	b	ooo+ooooo	CENTD3	centaurin, delta 3
199618	f	oo++----+	SPRR1B	small proline-rich protein 1B (cornifin)
199662	f	oo++----+	KCNJ10	potassium inwardly-rectifying channel, subfamily J, member 10
199757	g	oooo-----	CAV1	caveolin 1, caveolae protein, 22kDa
199772	b	ooo+ooooo	FAM20C	family with sequence similarity 20, member C
199775	a	oo+++++++	ATG4B	ATG4 autophagy related 4 homolog B (S. cerevisiae)
199838	g	o--------	TMEM16D	transmembrane protein 16D
199889	b	oooooo+oo	ODZ4	odz, odd Oz/ten-m homolog 4 (Drosophila)
199900	a	oo+++++++	RBM7	RNA binding motif protein 7
199902	b	oooo+oooo	null	null
199911	a	oo+++++++	TRNT1	tRNA nucleotidyl transferase, CCA-adding, 1
199941	h	oooo-oooo	ARFGAP1	ADP-ribosylation factor GTPase activating protein 1
199946	a	ooooooo++	PFAAP5	null
199956	b	ooo+ooooo	METT5D1|METT5D2	methyltransferase 5 domain containing 1|methyltransferase 5 domain containing 2
199984	f	oo++----+	FLJ30294	null
200021	h	o-ooooooo	LOC90321	null
200033	b	ooo+ooooo	FLJ23518	null
200111	b	ooo+ooooo	KIAA0100	KIAA0100
200133	a	o++++++++	INVS	inversin
200144	a	oo+++++++	SLC7A6OS	solute carrier family 7, member 6 opposite strand
200163	b	ooo+ooooo	CHD4	chromodomain helicase DNA binding protein 4
200242	g	o--------	null	null
200243	h	o--oooooo	THRAP1	thyroid hormone receptor associated protein 1
200280	h	oooooo-oo	DHRS6	dehydrogenase/reductase (SDR family) member 6
200285	b	ooo+ooooo	PCM1	pericentriolar material 1
200335	a	ooo++++++	CASP9	caspase 9, apoptosis-related cysteine peptidase
200352	b	ooo+ooooo	LRRCC1	leucine rich repeat and coiled-coil domain containing 1
200366	b	ooo+ooooo	DKFZp762P2111	null
200377	b	ooo+ooooo	STXBP5	syntaxin binding protein 5 (tomosyn)
200379	b	oooo+oooo	null	null
200388	b	ooo+ooooo	null	null
200440	b	ooo+ooooo	KRT6E	keratin 6E
200469	a	ooo++++++	SLC9A8	solute carrier family 9 (sodium/hydrogen exchanger), member 8
200572	a	ooo++++++	LOC440668	null
200588	b	ooo+ooooo	TYK2	tyrosine kinase 2
200602	b	ooooo+ooo	null	null
200626	h	o--oooooo	SLC5A11	solute carrier family 5 (sodium/glucose cotransporter), member 11
200629	a	oooooo+++	C6orf145	chromosome 6 open reading frame 145
200665	b	oo++ooooo	HMFN0839	null
200678	b	ooo+ooooo	KIAA1815	KIAA1815
200688	a	ooooooo++	null	null
200692	b	ooo++oooo	ATP9B	ATPase, Class II, type 9B
200703	h	oooo-oooo	PYDC1	PYD (pyrin domain) containing 1
200708	i	o-oo-----	IL2RA	interleukin 2 receptor, alpha
200724	g	ooooo----	DAG1	dystroglycan 1 (dystrophin-associated glycoprotein 1)
200759	b	ooo++oooo	ZNF701	zinc finger protein 701
200785	a	oooo+++++	SAMD12	sterile alpha motif domain containing 12
200835	b	ooo+ooooo	ABCA5	ATP-binding cassette, sub-family A (ABC1), member 5
200852	b	ooo+ooooo	NLN	neurolysin (metallopeptidase M3 family)
200856	k	ooooo-+oo	QKI	quaking homolog, KH domain RNA binding (mouse)
200882	h	o-ooooooo	MRRF	mitochondrial ribosome recycling factor
200910	g	o--------	SLC23A3	solute carrier family 23 (nucleobase transporters), member 3
200918	b	ooo+ooooo	PKP2	plakophilin 2
200923	b	ooo+ooooo	FLJ30313	null
200967	a	oooo+++++	null	null
201000	b	ooo+ooooo	USP47	ubiquitin specific peptidase 47
201068	b	ooo+ooooo	TMTC3	transmembrane and tetratricopeptide repeat containing 3
201107	b	ooooo+ooo	COL15A1	collagen, type XV, alpha 1
201130	b	ooo+ooooo	AGPAT6	1-acylglycerol-3-phosphate O-acyltransferase 6 (lysophosphatidic acid acyltransferase, zeta)
201158	g	o--------	FAM64A	family with sequence similarity 64, member A
201279	b	ooo+ooooo	KBTBD6	kelch repeat and BTB (POZ) domain containing 6
201283	b	ooo+ooooo	EPS15L2	epidermal growth factor receptor pathway substrate 15-like 2
201290	b	ooo+ooooo	VPS39	vacuolar protein sorting 39 (yeast)
201397	b	ooooo+ooo	ATP8B3	ATPase, Class I, type 8B, member 3
201429	b	oooooo+oo	EPHX1	epoxide hydrolase 1, microsomal (xenobiotic)
201431	c	ooo++o+++	C8G	complement component 8, gamma polypeptide
201440	g	oooooo---	null	null
201442	b	oooooo+oo	ACD	adrenocortical dysplasia homolog (mouse)

201466	g	oooo-----	HSPA8	heat shock 70kDa protein 8
201484	b	ooo+ooooo	PARP16	poly (ADP-ribose) polymerase family, member 16
201509	b	ooo+ooooo	FLJ12688	null
201581	a	oooo+++++	OAS1	2',5'-oligoadenylate synthetase 1, 40/46kDa
201597	b	ooooooo+o	TTTY12	testis-specific transcript, Y-linked 12
201688	i	o--o-----	null	null
201691	g	oooooooo-	null	null
201715	g	o--------	null	null
201723	b	ooo+ooooo	MOBKL2C	MOB1, Mps One Binder kinase activator-like 2C (yeast)
201735	g	o--------	KIAA1240	null
201788	b	oooooo++o	null	null
201790	b	oooo+oooo	C3orf25	chromosome 3 open reading frame 25
201818	g	o--------	PRSS12	protease, serine, 12 (neurotrypsin, motopsin)
201868	a	oooo+++++	JMJD1C	jumonji domain containing 1C
201876	a	oooooooo+	null	null
201923	b	ooo+ooooo	LOC388174	null
201983	f	oo++----+	TCERG1L	transcription elongation regulator 1-like
202001	a	oo+++++++	CELSR3	cadherin, EGF LAG seven-pass G-type receptor 3 (flamingo homolog, Drosophila)
202003	b	oooooo+oo	VPS37C	vacuolar protein sorting 37C (yeast)
202014	b	ooo+ooooo	FLJ16542	null
202017	b	ooo+ooooo	FLJ14054	null
202037	b	ooo++oooo	GALNT10	UDP-N-acetyl-alpha-D-galactosamine:polypeptide N-acetylgalactosaminyltransferase 10 (GalNAc-T10)
202051	b	ooo+ooooo	CELSR1	cadherin, EGF LAG seven-pass G-type receptor 1 (flamingo homolog, Drosophila)
202061	b	ooo+ooooo	LCHN	null
202160	g	oooo-----	PPP3CB	protein phosphatase 3 (formerly 2B), catalytic subunit, beta isoform (calcineurin A beta)
202169	g	o--------	OR52N1	olfactory receptor, family 52, subfamily N, member 1
202170	g	ooooooo--	null	null
202172	a	ooo++++++	STAM	signal transducing adaptor molecule (SH3 domain and ITAM motif) 1
202286	g	o--------	CRSP9	cofactor required for Sp1 transcriptional activation, subunit 9, 33kDa
202329	b	ooo++++oo	null	null
202353	h	oooo----o	SEC14L3	SEC14-like 3 (S. cerevisiae)
202363	b	ooo+ooooo	NFKB1	nuclear factor of kappa light polypeptide gene enhancer in B-cells 1 (p105)
202407	f	oo++----+	C16orf55	chromosome 16 open reading frame 55
202418	a	o++++++++	ILF3	interleukin enhancer binding factor 3, 90kDa
202427	h	o-ooooooo	MGC33648	null
202435	h	oooo--ooo	CPEB2	cytoplasmic polyadenylation element binding protein 2
202436	a	oooooo+++	INHBE	inhibin, beta E
202457	a	oooooo+++	LY6G5B	lymphocyte antigen 6 complex, locus G5B
202506	b	ooooooo+o	ABCB11	ATP-binding cassette, sub-family B (MDR/TAP), member 11
202522	b	ooo+ooooo	HOOK1	hook homolog 1 (Drosophila)
202523	b	ooo+ooooo	CTAGE5	CTAGE family, member 5
202525	b	ooo+ooooo	null	null
202526	b	ooo+ooooo	null	null
202528	g	o--------	TAS2R14	taste receptor, type 2, member 14
202542	a	oo+++++++	FLJ31438	null
202561	b	ooooooo+o	PRO1580	null
202628	b	o+ooooooo	SEMA6D	sema domain, transmembrane domain (TM), and cytoplasmic domain, (semaphorin) 6D
202631	b	ooo++oooo	GDAP1	ganglioside-induced differentiation-associated protein 1
202634	a	o++++++++	KIAA0423	KIAA0423
202641	a	oo+++++++	LOC400604	null
202723	b	ooo+ooooo	null	null
202743	a	oo+++++++	null	null
202748	a	ooooooo++	TKTL1	transketolase-like 1
202780	a	oooo+++++	ADM	adrenomedullin
202796	g	oo-------	INHBB	inhibin, beta B (activin AB beta polypeptide)
202808	g	o--------	RDH12	retinol dehydrogenase 12 (all-trans and 9-cis)
202870	a	ooo++++++	GDAP2	ganglioside induced differentiation associated protein 2
202880	h	oooo-oooo	HRMT1L1	HMT1 hnRNP methyltransferase-like 1 (S. cerevisiae)
202913	b	ooo+ooooo	ZFYVE16	zinc finger, FYVE domain containing 16
202937	a	ooo++++++	IFIT3	interferon-induced protein with tetratricopeptide repeats 3
202971	a	oo+++++++	CYP21A2	cytochrome P450, family 21, subfamily A, polypeptide 2
203034	b	ooooo+ooo	DCX	doublecortex; lissencephaly, X-linked (doublecortin)
203075	b	ooo+++ooo	C5orf13	chromosome 5 open reading frame 13
203081	a	ooooooo++	null	null
203093	b	ooo+ooooo	TRPV1	transient receptor potential cation channel, subfamily V, member 1
203098	c	ooo+oo+++	BACH1	BTB and CNC homology 1, basic leucine zipper transcription factor 1
203105	a	ooo++++++	MN1	meningioma (disrupted in balanced translocation) 1
203123	g	oo-------	CLEC2B	C-type lectin domain family 2, member B

203137	i	o----o---	BPY2IP1	BPY2 interacting protein 1
203148	b	oo+oooooo	CYP3A4	cytochrome P450, family 3, subfamily A, polypeptide 4
203170	b	oo++++++o	EIF4A1|CD68	eukaryotic translation initiation factor 4A, isoform 1|CD68 antigen
203198	b	ooo+ooooo	ZFP28	zinc finger protein 28 homolog (mouse)
203258	f	oo++----+	SLC6A13	solute carrier family 6 (neurotransmitter transporter, GABA), member 13
203299	a	o++++++++	KIAA1458	null
203432	a	ooooo++++	LOC392529	null
203435	h	oooo----o	FLJ30092	null
203450	b	ooooo+ooo	FLJ35821	null
203455	i	ooooo-oo-	SEC24C	SEC24 related gene family, member C (S. cerevisiae)
203461	b	ooooooo+o	ARHGAP28	Rho GTPase activating protein 28
203481	g	oooo-----	OR8G2	olfactory receptor, family 8, subfamily G, member 2
203507	g	o--------	TTYH3	tweety homolog 3 (Drosophila)
203610	b	ooo+ooooo	ABP1	amiloride binding protein 1 (amine oxidase (copper-containing))
203613	i	o-oo-----	AFMID	arylformamidase
203635	b	ooo+ooooo	C4A|XXbac-BPG116M5.7|C4B	complement component 4A|complement component 4B
203644	c	ooo+oooo+	DKK3	dickkopf homolog 3 (Xenopus laevis)
203726	a	oooooooo+	PABPC5	poly(A) binding protein, cytoplasmic 5
203757	b	ooooooo+o	null	null
203772	h	oooo--ooo	FAM20B	family with sequence similarity 20, member B
203777	a	oooooooo+	LOC283247|NOX4	NADPH oxidase 4
203778	h	o-ooooooo	TAF3	TAF3 RNA polymerase II, TATA box binding protein (TBP)-associated factor, 140kDa
203823	b	ooo+++++o	null	null
203851	b	oo++++++o	MGC16037	null
203860	a	oooooooo+	NR2E1	nuclear receptor subfamily 2, group E, member 1
203863	a	o++++++++	NFIL3	nuclear factor, interleukin 3 regulated
203869	a	ooooooo++	CCNB1IP1	cyclin B1 interacting protein 1
203894	a	oooooooo+	null	null
203920	b	ooo++oooo	PTHB1	null
203927	a	oooooooo+	ATP4B	ATPase, H+/K+ exchanging, beta polypeptide
203941	b	oooooo+oo	KLF13	Kruppel-like factor 13
203961	b	ooooo+ooo	TESSP1	null
204006	a	ooo++++++	CDKN2B	cyclin-dependent kinase inhibitor 2B (p15, inhibits CDK4)
204044	g	oooooo---	PTTG1	pituitary tumor-transforming 1
204067	b	ooo+ooooo	IFT80	intraflagellar transport 80 homolog (Chlamydomonas)
204101	c	ooo+ooo++	USPL1	ubiquitin specific peptidase like 1
204148	b	ooo+ooooo	ZNF482	zinc finger protein 482
204179	a	ooo++++++	SLC35A3	solute carrier family 35 (UDP-N-acetylglucosamine (UDP-GlcNAc) transporter), member A3
204195	b	ooo+ooooo	TTC12	tetratricopeptide repeat domain 12
204262	i	o-----o--	ATF5	activating transcription factor 5
204385	b	ooo+ooooo	TNRC15	trinucleotide repeat containing 15
204401	b	ooo+ooooo	ROD1	ROD1 regulator of differentiation 1 (S. pombe)
204454	b	ooooo+ooo	MNS1	meiosis-specific nuclear structural 1
204458	b	o+ooooooo	MARCO	macrophage receptor with collagenous structure
204492	g	ooooo----	MAP4	microtubule-associated protein 4
204547	h	ooooo-ooo	C22orf8	chromosome 22 open reading frame 8
204591	b	ooo+ooooo	THNSL1	threonine synthase-like 1 (bacterial)
204604	b	ooo+ooooo	ZNF518	zinc finger protein 518
204620	a	ooooooo++	RABGGTB	Rab geranylgeranyltransferase, beta subunit
204643	h	o-ooooooo	BPNT1	3'(2'), 5'-bisphosphate nucleotidase 1
204648	g	o--------	TAAR8	trace amine associated receptor 8
204670	g	ooo------	GRM1	glutamate receptor, metabotropic 1
204717	b	ooo++oooo	C8orf42	chromosome 8 open reading frame 42
204743	a	ooooo++++	ID1	inhibitor of DNA binding 1, dominant negative helix-loop-helix protein
204758	b	ooo++oooo	RXRB	retinoid X receptor, beta
204773	g	oooo-----	MT1A	metallothionein 1A (functional)
204791	a	oo+++++++	null	null
204834	a	ooo++++++	MGC33214	null
204851	a	ooo++++++	HBP1	HMG-box transcription factor 1
204861	b	oo++ooooo	null	null
204892	b	ooo+ooooo	RNF31	ring finger protein 31
204912	b	oooo++ooo	OR1S1	olfactory receptor, family 1, subfamily S, member 1
204939	g	oooo-----	KIAA0195	KIAA0195
205016	a	ooooooo++	PBEF1	pre-B-cell colony enhancing factor 1
205021	b	ooooooo+o	SERPINB12	serpin peptidase inhibitor, clade B (ovalbumin), member 12
205024	b	ooo+ooooo	LOC90826	null
205030	b	oooo+oooo	TSPYL5	TSPY-like 5
205035	b	ooo+ooooo	STIM2	stromal interaction molecule 2

205036	a	oo+++++++	PRDM1	PR domain containing 1, with ZNF domain
205042	c	oo++oooo+	ZNF266	zinc finger protein 266
205100	a	o++++++++	RAB33B	RAB33B, member RAS oncogene family
205128	a	ooo++++++	FOS	v-fos FBJ murine osteosarcoma viral oncogene homolog
205132	b	ooo+ooooo	SLC25A16	solute carrier family 25 (mitochondrial carrier; Graves disease autoantigen), member 16
205146	b	ooo+ooooo	KIAA0971	KIAA0971
205151	h	o-ooooooo	LOC401967	null
205155	b	oooooo+oo	PRDM14	PR domain containing 14
205168	h	o-ooooooo	C17orf41	chromosome 17 open reading frame 41
205184	b	ooo+ooooo	LOC339778	null
205221	i	o-oo-----	ZDHHC5	zinc finger, DHHC-type containing 5
205237	b	oooooo+oo	NR2E3	nuclear receptor subfamily 2, group E, member 3
205247	b	ooo+ooooo	ALOX12B	arachidonate 12-lipoxygenase, 12R type
205292	b	o+ooooooo	GCA	grancalcin, EF-hand calcium binding protein
205301	b	ooo+ooooo	FANCC	Fanconi anemia, complementation group C
205314	b	oo+oooooo	LYNX1	Ly6/neurotoxin 1
205341	b	ooooooo+o	FLJ22349	null
205345	i	o---o----	TRAV20	T cell receptor alpha variable 20
205361	a	oo+++++++	EXOC3	exocyst complex component 3
205367	b	ooo+ooooo	BTBD7	BTB (POZ) domain containing 7
205397	b	ooooooo+o	null	null
205403	g	oooooo---	LOC221143	null
205410	b	o+ooooooo	OR2H1	olfactory receptor, family 2, subfamily H, member 1
205425	i	oooo-o---	SNRPG	small nuclear ribonucleoprotein polypeptide G
205440	b	oo+oooooo	null	null
205467	b	oooo+oooo	null	null
205502	g	oooo-----	LOC441198	null
205519	b	ooooo+ooo	CD2	CD2 antigen (p50), sheep red blood cell receptor
205613	f	oo++----+	HUNK	hormonally upregulated Neu-associated kinase
205623	a	oo+++++++	RPA4	replication protein A4, 34kDa
205633	g	o--------	OR2C3	olfactory receptor, family 2, subfamily C, member 3
205649	b	ooo+++ooo	ASPM	asp (abnormal spindle)-like, microcephaly associated (Drosophila)
205688	b	ooooooo+o	null	null
205706	b	ooo+ooooo	TLE4	transducin-like enhancer of split 4 (E(sp1) homolog, Drosophila)
205794	b	ooo+ooooo	CEP152	centrosomal protein 152kDa
205859	g	o--------	AQP3	aquaporin 3
205920	a	ooo++++++	WDR26	WD repeat domain 26
205933	b	oooooo+oo	PCTK3	PCTAIRE protein kinase 3
205942	a	ooo++++++	GTF2H1	general transcription factor IIH, polypeptide 1, 62kDa
205961	j	o-oooo+++	CLK1	CDC-like kinase 1
205981	b	ooo++oooo	PHKA2	phosphorylase kinase, alpha 2 (liver)
205982	a	ooo++++++	TBC1D2	TBC1 domain family, member 2
206016	g	ooooo----	ATXN10	ataxin 10
206021	f	oo++----+	MGC4172	null
206104	c	oooo+oo++	SLC28A2	solute carrier family 28 (sodium-coupled nucleoside transporter), member 2
206170	a	ooo++++++	KLK12	kallikrein 12
206251	c	ooo+oo+++	ZBTB5	zinc finger and BTB domain containing 5
206283	g	oooo-----	null	null
206301	b	ooo+ooooo	PSD3	pleckstrin and Sec7 domain containing 3
206405	c	oooo+o+++	THEX1	three prime histone mRNA exonuclease 1
206417	b	ooo+ooooo	CHN1	chimerin (chimaerin) 1
206419	b	ooo+++++o	ZBTB1	zinc finger and BTB domain containing 1
206456	g	oooo-----	TMTC1	transmembrane and tetratricopeptide repeat containing 1
206479	b	ooo+ooooo	NEDD4	neural precursor cell expressed, developmentally down-regulated 4
206481	b	ooo+++++o	TAAR3	trace amine associated receptor 3
206502	a	oooooo+++	ZNF669	zinc finger protein 669
206535	g	o--------	CR2	complement component (3d/Epstein Barr virus) receptor 2
206536	g	oooooooo-	null	null
206539	g	o--------	BCL9	B-cell CLL/lymphoma 9
206559	a	oooo+++++	null	null
206565	b	ooooooo+o	TIRAP	toll-interleukin 1 receptor (TIR) domain containing adaptor protein
206576	b	oo++++ooo	SUCLA2	succinate-CoA ligase, ADP-forming, beta subunit
206584	b	ooo+ooooo	KLHL22	kelch-like 22 (Drosophila)
206606	a	oooooo+++	AVPI1	arginine vasopressin-induced 1
206625	b	ooo+ooooo	CLEC1B	C-type lectin domain family 1, member B
206638	h	oooooo-oo	EPB41	erythrocyte membrane protein band 4.1 (elliptocytosis 1, RH-linked)
206647	b	ooo+ooooo	ALG10	asparagine-linked glycosylation 10 homolog (yeast, alpha-1,2-glucosyltransferase)
206668	b	ooooo+ooo	null	null
206703	g	oooooooo-	C9orf16	chromosome 9 open reading frame 16
206714	b	o+ooooooo	AGBL4	ATP/GTP binding protein-like 4

206815	a	ooo++++++	MGC10812	null
206890	b	oooo+oooo	RARB	retinoic acid receptor, beta
206912	a	ooo++++++	ABHD9	abhydrolase domain containing 9
206925	b	ooo+ooooo	WDR59	WD repeat domain 59
206927	f	oo++----+	SLA	Src-like-adaptor
206934	g	o--------	NEU4	sialidase 4
206942	b	ooo+ooooo	MID2	midline 2
206947	c	o+o++++++	SMAD7	SMAD, mothers against DPP homolog 7 (Drosophila)
206958	b	ooo+ooooo	SERPINB10	serpin peptidase inhibitor, clade B (ovalbumin), member 10
206994	a	ooo++++++	ZNF567	zinc finger protein 567
207014	b	ooo+ooooo	CENTD2	centaurin, delta 2
207067	b	oo++++++o	FLJ43663	null
207078	b	oooooo+oo	FLJ90834	null
207106	c	ooo+ooo++	null	null
207111	a	o++++++++	GMFB	glia maturation factor, beta
207124	a	ooo++++++	RBM5	RNA binding motif protein 5
207178	a	ooo++++++	RP13-297E16.1	null
207179	a	ooo++++++	ZBTB25	zinc finger and BTB domain containing 25
207231	h	o-ooooooo	SPRY2	sprouty homolog 2 (Drosophila)
207279	b	oooo+oooo	null	null
207283	c	oo++oo+++	HFE	hemochromatosis
207292	a	ooo++++++	CCNT2	cyclin T2
207317	a	ooo++++++	SHRM	null
207329	b	ooo+ooooo	ZNF526	zinc finger protein 526
207344	b	ooo++oooo	UNQ5810	null
207381	b	ooooooo+o	SLC30A4	solute carrier family 30 (zinc transporter), member 4
207412	b	ooooooo+o	P2RY10	purinergic receptor P2Y, G-protein coupled, 10
207433	b	oooo+oooo	BICD2	bicaudal D homolog 2 (Drosophila)
207463	a	o++++++++	MMAB	methylmalonic aciduria (cobalamin deficiency) cblB type
207510	h	oooo----o	FAM55C	family with sequence similarity 55, member C
207524	b	ooo++oooo	CSPG2	chondroitin sulfate proteoglycan 2 (versican)
207598	g	ooooo----	SLC39A10	solute carrier family 39 (zinc transporter), member 10
207619	b	ooo+ooooo	KIAA1718	null
207660	b	ooo+ooooo	TOP3A	topoisomerase (DNA) III alpha
207681	a	oooooooo+	SLC34A1	solute carrier family 34 (sodium phosphate), member 1
207688	a	ooo++++++	SFRS15	splicing factor, arginine/serine-rich 15
207727	b	oooo+oooo	HYOU1	hypoxia up-regulated 1
207755	b	oooooo+oo	IGFBP4	insulin-like growth factor binding protein 4
207786	g	o--------	null	null
207792	b	ooooooo+o	DLC1	deleted in liver cancer 1
207803	g	oooo-----	MYBL1	v-myb myeloblastosis viral oncogene homolog (avian)-like 1
207869	g	ooooooo--	AURKC	aurora kinase C
207901	h	oooo----o	TAS2R39	taste receptor, type 2, member 39
207919	a	ooo++++++	C10orf88	chromosome 10 open reading frame 88
207932	b	o+ooooooo	null	null
207998	b	oooooo+oo	ERO1LB	ERO1-like beta (S. cerevisiae)
208035	b	ooo+ooooo	GALNT7	UDP-N-acetyl-alpha-D-galactosamine:polypeptide N-acetylgalactosaminyltransferase 7 (GalNAc-T7)
208071	b	o+ooooooo	AFF2	AF4/FMR2 family, member 2
208079	g	ooo------	MED28	mediator of RNA polymerase II transcription, subunit 28 homolog (yeast)
208099	g	o--------	LOC389174	null
208102	b	ooo+ooooo	ARHGAP10	Rho GTPase activating protein 10
208118	a	oooooo+++	RPP38	ribonuclease P/MRP 38kDa subunit
208143	b	oooooo+oo	PEX10	peroxisome biogenesis factor 10
208153	a	oo+++++++	AMY2B	amylase, alpha 2B; pancreatic
208172	h	oooooo--o	null	null
208200	b	ooo++oooo	IFNA21	interferon, alpha 21
208250	a	ooo++++++	null	null
208255	c	oo++oooo+	FAM98C	family with sequence similarity 98, member C
208285	b	ooo++oooo	TXNDC6	thioredoxin domain containing 6
208294	b	ooo+++ooo	null	null
208313	g	oooo-----	MUC20	mucin 20
208387	b	ooo+ooooo	ARHGEF17	Rho guanine nucleotide exchange factor (GEF) 17
208388	a	ooo++++++	PTGS2	prostaglandin-endoperoxide synthase 2 (prostaglandin G/H synthase and cyclooxygenase)
208402	b	ooo+ooooo	MAP3K4	mitogen-activated protein kinase kinase kinase 4
208502	a	oooooooo+	LOC387775	null
208526	b	ooo++oooo	C22orf9	chromosome 22 open reading frame 9
208529	g	o--------	LGR5	leucine-rich repeat-containing G protein-coupled receptor 5
208543	b	ooo+ooooo	RP11-37E23.6	null
208556	b	ooo+ooooo	FLJ13231	null

208583	h	oooooo-oo	null	null
208588	b	ooo+ooooo	C13orf24	chromosome 13 open reading frame 24
208606	a	ooo++++++	BCL2L11	BCL2-like 11 (apoptosis facilitator)
208632	b	ooo+ooooo	IBRDC1	IBR domain containing 1
208663	a	oooooo+++	EIF1	eukaryotic translation initiation factor 1
208695	g	oooooo---	AK3	adenylate kinase 3
208696	b	ooo+ooooo	DZIP3	null
208701	a	o++++++++	FLJ43654	null
208710	b	ooo+ooooo	STAC	SH3 and cysteine rich domain
208725	b	ooo+ooooo	FLJ35779	null
208731	b	oooo+oooo	null	null
208765	b	ooo++oooo	CENTA2	centaurin, alpha 2
208773	a	o++++++++	OTUD3	OTU domain containing 3
208813	i	o---o----	PLA2G5	phospholipase A2, group V
208858	i	o-oo-----	SLC12A3	solute carrier family 12 (sodium/chloride transporters), member 3
208963	b	ooo+ooooo	C14orf45	chromosome 14 open reading frame 45
208980	b	ooo+ooooo	MBD1	methyl-CpG binding domain protein 1
208981	b	oooo+oooo	ADAM21	ADAM metallopeptidase domain 21
209022	b	oooo+oooo	ZNF449	zinc finger protein 449
209051	b	oooooo+oo	SMARCD1	SWI/SNF related, matrix associated, actin dependent regulator of chromatin, subfamily d, member 1
209085	i	o-oo-----	POF1B	premature ovarian failure, 1B
209115	h	oooo--ooo	null	null
209122	b	ooooo+ooo	ADAM18	ADAM metallopeptidase domain 18
209155	b	ooo+ooooo	KIAA1509	KIAA1509
209180	h	oooo----o	MUC7	mucin 7, salivary
209191	a	ooooooo++	MLR1	null
209306	a	o++++++++	UBPH	null
209315	a	ooo++++++	MTO1	mitochondrial translation optimization 1 homolog (S. cerevisiae)
209361	h	o-ooooooo	JARID1A	Jumonji, AT rich interactive domain 1A (RBBP2-like)
209365	b	ooo++oooo	LRRC58	leucine rich repeat containing 58
209385	b	o+ooooooo	SDCBP2	syndecan binding protein (syntenin) 2
209401	a	oo+++++++	UTS2D	urotensin 2 domain containing
209426	a	ooo++++++	CD83	CD83 antigen (activated B lymphocytes, immunoglobulin superfamily)
209427	b	ooo+ooooo	MCM10	MCM10 minichromosome maintenance deficient 10 (S. cerevisiae)
209446	a	o++++++++	SEC24B	SEC24 related gene family, member B (S. cerevisiae)
209513	h	o-ooooooo	ANKRD18A	ankyrin repeat domain 18A
209577	b	ooo++oooo	ATP8B4	ATPase, Class I, type 8B, member 4
209600	a	oooooooo+	RFPL2	ret finger protein-like 2
209663	a	o++++++++	AGBL2	ATP/GTP binding protein-like 2
209668	b	oooo+oooo	null	null
209686	a	ooo++++++	PTP4A1	protein tyrosine phosphatase type IVA, member 1
209723	b	ooo+ooooo	MYO5B	myosin VB
209753	g	oooooooo-	COL16A1	collagen, type XVI, alpha 1
209759	h	o-ooooooo	ZNF297B	zinc finger protein 297B
209773	b	ooo+ooooo	AP3B2	adaptor-related protein complex 3, beta 2 subunit
209780	b	ooo+ooooo	TTC13	tetratricopeptide repeat domain 13
209781	b	ooo+ooooo	TBC1D2B	TBC1 domain family, member 2B
209793	b	ooo+ooooo	DCAMKL2	doublecortin and CaM kinase-like 2
209909	b	ooo+ooooo	GNAO1	guanine nucleotide binding protein (G protein), alpha activating activity polypeptide O
209913	b	ooo+ooooo	FLJ32810	null
209973	g	oooo-----	MCM3	MCM3 minichromosome maintenance deficient 3 (S. cerevisiae)
210109	b	ooo+++++o	null	null
210120	a	ooo++++++	BZW1	basic leucine zipper and W2 domains 1
210149	b	ooo+ooooo	DDEF2	development and differentiation enhancing factor 2
210206	a	oo+++++++	null	null
210229	b	ooo+ooooo	RASAL2	RAS protein activator like 2
210232	h	oooo----o	null	null
210238	b	ooo+ooooo	null	null
210241	b	oooo+oooo	TRERF1	transcriptional regulating factor 1
210290	e	oo++----o	null	null
210350	b	ooo++++oo	DMTF1	cyclin D binding myb-like transcription factor 1
210363	b	oooo+oooo	PPP1R3B	protein phosphatase 1, regulatory (inhibitor) subunit 3B
210377	a	ooooo++++	PFN2	profilin 2
210401	a	ooooo++++	CDK7	cyclin-dependent kinase 7 (MO15 homolog, Xenopus laevis, cdk-activating kinase)
210404	b	oooooo+oo	TNMD	tenomodulin
210420	a	ooo++++++	STX1A	syntaxin 1A (brain)
210449	h	oooo--ooo	LOC442028	null
210455	h	o-ooooooo	MGC35361	null
210503	b	ooo+ooooo	TFDP2	transcription factor Dp-2 (E2F dimerization partner 2)

210521	g	o--------	ASTN	astrotactin
210525	c	oooo+oo++	KCNJ16	potassium inwardly-rectifying channel, subfamily J, member 16
210528	f	oo++----+	null	null
210561	a	oo+++++++	PANK2	pantothenate kinase 2 (Hallervorden-Spatz syndrome)
210579	b	oooo+oooo	null	null
210580	a	ooo++++++	MGC13024	null
210630	b	ooo+ooooo	MGC13005	null
210657	a	oooo+++++	CEL	carboxyl ester lipase (bile salt-stimulated lipase)
210661	b	ooo+ooooo	ZNF607	zinc finger protein 607
210662	b	ooo+ooooo	ABCG1	ATP-binding cassette, sub-family G (WHITE), member 1
210733	b	oooooo+oo	null	null
210751	b	oo+oooooo	OR5U1	olfactory receptor, family 5, subfamily U, member 1
210759	b	oooo+oooo	LOC440025	null
210780	b	ooo++oooo	SLC22A3	solute carrier family 22 (extraneuronal monoamine transporter), member 3
210785	b	oooo+oooo	CLPS	colipase, pancreatic
210786	a	ooo++++++	ZMYM5	zinc finger, MYM-type 5
210798	h	o-ooooooo	CARS	cysteinyl-tRNA synthetase
210813	i	o-oo-----	HECA	headcase homolog (Drosophila)
210834	b	oooo+oooo	FLJ11800|LOC440411	null
211028	b	oooooo+oo	CASP2	caspase 2, apoptosis-related cysteine peptidase (neural precursor cell expressed, developmentally down-regulated 2)
211058	b	ooooo+ooo	ALPL	alkaline phosphatase, liver/bone/kidney
211103	f	oo++----+	C14orf37	chromosome 14 open reading frame 37
211110	g	o--------	OPRD1	opioid receptor, delta 1
211118	a	ooo++++++	GPR37	G protein-coupled receptor 37 (endothelin receptor type B-like)
211134	b	oooooo+oo	ARHGDIA	Rho GDP dissociation inhibitor (GDI) alpha
211137	b	oo+oooooo	LOC440104	null
211174	g	oooo-----	UNC84B	unc-84 homolog B (C. elegans)
211211	b	ooo++oooo	SACS	spastic ataxia of Charlevoix-Saguenay (sacsin)
211223	a	oooooooo+	FLJ35630	null
211237	b	ooo+ooooo	ZNF92	zinc finger protein 92 (HTF12)
211255	b	ooo+ooooo	GFPT1	glutamine-fructose-6-phosphate transaminase 1
211278	b	ooo+ooooo	NT5E	5'-nucleotidase, ecto (CD73)
211294	g	ooooooo--	TRIM6	tripartite motif-containing 6
211296	h	oooo----o	LOC162993	null
211319	h	oooo-oooo	LOC119710	null
211325	b	ooo+ooooo	ZNF650	zinc finger protein 650
211331	c	ooo+ooo++	CD4	CD4 antigen (p55)
211333	g	oooo-----	SLC39A13	solute carrier family 39 (zinc transporter), member 13
211410	b	ooo++oooo	ZNF573	zinc finger protein 573
211412	a	oooooo+++	KLF6	Kruppel-like factor 6
211421	f	oo++----+	RGS9	regulator of G-protein signalling 9
211456	c	oo++oooo+	SFXN5	sideroflexin 5
211473	b	oooooo+oo	KIAA1909	null
211502	b	oooooo+oo	AGPAT1	1-acylglycerol-3-phosphate O-acyltransferase 1 (lysophosphatidic acid acyltransferase, alpha)
211514	a	ooo++++++	KBTBD2	kelch repeat and BTB (POZ) domain containing 2
211519	a	oooooooo+	ZNF24	zinc finger protein 24 (KOX 17)
211534	a	oooooooo+	FABP7	fatty acid binding protein 7, brain
211562	b	ooo+ooooo	FLJ21439	null
211565	b	ooo+ooooo	XK	Kell blood group precursor (McLeod phenotype)
211578	a	ooo++++++	GPBP1	GC-rich promoter binding protein 1
211587	a	ooooooo++	C20orf58	chromosome 20 open reading frame 58
211597	b	oooooo+oo	FBXO31	F-box protein 31
211621	b	ooo++++oo	TIGD7	tigger transposable element derived 7
211675	b	oooooo++o	APITD1|CORT	apoptosis-inducing, TAF9-like domain 1|cortistatin
211683	b	ooo+ooooo	HERC2P2|LOC440248	hect domain and RLD 2 pseudogene 2
211692	b	oooooo+oo	TNFRSF1A	tumor necrosis factor receptor superfamily, member 1A
211694	h	o-ooooooo	SOAT1	sterol O-acyltransferase (acyl-Coenzyme A: cholesterol acyltransferase) 1
211697	b	ooo+ooooo	null	null
211722	b	ooo+ooooo	ZNF676	zinc finger protein 676
211789	c	ooo+o++++	PPP1R15B	protein phosphatase 1, regulatory (inhibitor) subunit 15B
211796	b	ooooooo+o	NAGS	N-acetylglutamate synthase
211806	b	ooooo+ooo	NTN2L	netrin 2-like (chicken)
211834	a	oooooooo+	null	null
211881	b	ooo+ooooo	LOC348840	null
211912	b	ooo+ooooo	FMNL2	formin-like 2
211923	g	oooooooo-	C16orf33	chromosome 16 open reading frame 33
211949	g	o--------	CST7	cystatin F (leukocystatin)

211978	b	ooooo+ooo	MDS028	null
211988	b	ooo+ooooo	ZNF462	zinc finger protein 462
211995	i	o-oo-----	UNKL	unkempt-like (Drosophila)
212022	h	ooooo-ooo	SERINC5	serine incorporator 5
212075	b	ooo+ooooo	CP110	null
212091	b	ooo+ooooo	ZNF227	zinc finger protein 227
212126	b	oo++ooooo	GNGT2	guanine nucleotide binding protein (G protein), gamma transducing activity polypeptide 2
212132	c	oo++oooo+	FBLN5	fibulin 5
212174	a	oo+++++++	POMT1	protein-O-mannosyltransferase 1
212202	b	ooo+ooooo	MAN1B1	mannosidase, alpha, class 1B, member 1
212210	b	oooo+oooo	ZBTB33	zinc finger and BTB domain containing 33
212263	b	ooo+ooooo	null	null
212292	a	oo+++++++	MSI2	musashi homolog 2 (Drosophila)
212324	a	oooooo+++	CAMLG	calcium modulating ligand
212369	g	oo-------	PPAPDC2	phosphatidic acid phosphatase type 2 domain containing 2
212373	i	oooo-o---	MT1L	metallothionein 1L
212378	b	ooo+++++o	PTPN6	protein tyrosine phosphatase, non-receptor type 6
212405	a	oooooooo+	null	null
212421	b	ooo+ooooo	ZNF20	zinc finger protein 20 (KOX 13)
212454	h	oooo----o	CYP17A1	cytochrome P450, family 17, subfamily A, polypeptide 1
212465	g	oo-------	SATB1	special AT-rich sequence binding protein 1 (binds to nuclear matrix/scaffold-associating DNA's)
212478	c	oo++oooo+	C6orf167	chromosome 6 open reading frame 167
212485	b	ooo+ooooo	null	null
212486	b	ooo+ooooo	ZNF343	zinc finger protein 343
212515	a	ooooooo++	null	null
212530	b	ooo++++oo	null	null
212543	b	o+ooooooo	SPTLC2	serine palmitoyltransferase, long chain base subunit 2
212552	b	ooo+++++o	ZNF546	zinc finger protein 546
212615	b	ooo+ooooo	RPL19|LOC390595	ribosomal protein L19
212644	b	ooo++oooo	RP1-199H16.1	null
212677	i	o--o-----	null	null
212759	a	oo+++++++	GPI	glucose phosphate isomerase
212865	b	oooooo+oo	null	null
212871	b	ooooooo+o	FLJ27354	null
212887	g	ooooooo--	SYTL4	synaptotagmin-like 4 (granuphilin-a)
212890	a	oooooo+++	FRAT2	frequently rearranged in advanced T-cell lymphomas 2
212894	h	o--oooooo	ZNF587	zinc finger protein 587
212896	b	ooo+ooooo	FLJ33167	null
212907	b	ooo+ooooo	ZNF198	zinc finger protein 198
212910	a	oooooo+++	C1orf52	chromosome 1 open reading frame 52
212964	a	o++++++++	null	null
212988	b	ooo+ooooo	ADAM10	ADAM metallopeptidase domain 10
212994	g	oo-------	TUBA6	tubulin, alpha 6
213028	h	o-----ooo	BCL7B	B-cell CLL/lymphoma 7B
213143	b	ooo+ooooo	hCAP-D3	null
213145	g	oooooooo-	C21orf45	chromosome 21 open reading frame 45
213152	a	ooo++++++	PRKAA2	protein kinase, AMP-activated, alpha 2 catalytic subunit
213163	b	oooo++ooo	FLJ20309	null
213177	c	oo++oooo+	PCSK1	proprotein convertase subtilisin/kexin type 1
213319	a	oooo+++++	null	null
213366	a	ooo++++++	null	null
213394	a	oooooooo+	null	null
213411	a	o++++++++	null	null
213429	a	oo+++++++	null	null
213482	b	oooooo+oo	PLAC1	placenta-specific 1
213498	a	ooo++++++	null	null
213510	h	oooooo-oo	null	null
213523	a	oooooo+++	ZNF655	zinc finger protein 655
213545	b	ooooo+ooo	null	null
213570	c	oo++oooo+	null	null
213585	b	ooooooo+o	ZNF347	zinc finger protein 347
213598	h	o-ooooooo	MTUS1	mitochondrial tumor suppressor 1
213612	b	oooo+oooo	CD34	CD34 antigen
213617	b	ooo+ooooo	ZNF588	zinc finger protein 588
213647	b	ooooo+ooo	OR4C11	olfactory receptor, family 4, subfamily C, member 11
213652	a	ooo++++++	LETMD1	LETM1 domain containing 1
213724	g	oooooooo-	PHF11	PHD finger protein 11
213739	b	ooo++oooo	RNF150	ring finger protein 150
213740	b	o+++ooooo	CABLES2	Cdk5 and Abl enzyme substrate 2

213820	b	ooo+ooooo	USP40	ubiquitin specific peptidase 40
213826	b	oooooo+oo	RQCD1	RCD1 required for cell differentiation1 homolog (S. pombe)
213842	a	oooooo+++	LOC388503	null
213867	a	ooooooo++	NKX2-6	null
213876	a	ooo++++++	PRR8	proline rich 8
213878	a	ooo++++++	HEL308	null
213922	a	ooo++++++	WTAP	Wilms tumor 1 associated protein
214016	b	oooo+oooo	null	null
214037	c	ooo+oo+++	DKFZp761P0423	null
214083	a	oooooo+++	KIAA1076	null
214121	a	oo+++++++	TATDN2	TatD DNase domain containing 2
214131	b	ooo+ooooo	ATP6V0A1	ATPase, H+ transporting, lysosomal V0 subunit a isoform 1
214151	b	o+ooooooo	TOR1AIP2	torsin A interacting protein 2
214188	b	ooo+ooooo	BRUNOL4	bruno-like 4, RNA binding protein (Drosophila)
214241	h	o-ooooooo	PXK	PX domain containing serine/threonine kinase
214268	a	oo+++++++	HYAL3	hyaluronoglucosaminidase 3
214300	a	oooooooo+	MTX3	metaxin 3
214313	b	oooooo+oo	LOC151534	null
214318	b	oo++ooooo	GALNT11	UDP-N-acetyl-alpha-D-galactosamine:polypeptide N-acetylgalactosaminyltransferase 11 (GalNAc-T11)
214325	a	ooo++++++	TBL2	transducin (beta)-like 2
214412	g	ooooo----	MID1	midline 1 (Opitz/BBB syndrome)
214499	b	ooo+ooooo	CACHD1	cache domain containing 1
214501	h	o-ooooooo	S100Z	S100 calcium binding protein, zeta
214513	i	o----oo--	PB1	null
214524	b	ooo+ooooo	RAPGEF6	Rap guanine nucleotide exchange factor (GEF) 6
214636	a	ooo++++++	TBC1D8	TBC1 domain family, member 8 (with GRAM domain)
214655	b	ooooo+ooo	INHBC	inhibin, beta C
214731	g	o--------	SCNN1G	sodium channel, nonvoltage-gated 1, gamma
214739	g	o--------	CCR1	chemokine (C-C motif) receptor 1
214768	b	o+++++ooo	MEOX1	mesenchyme homeobox 1
214773	a	o++++++++	MAOB	monoamine oxidase B
214792	e	oo++----o	TMEM87B	transmembrane protein 87B
214799	b	ooo+ooooo	OR5B21	olfactory receptor, family 5, subfamily B, member 21
214807	g	oooooooo-	GSTP1	glutathione S-transferase pi
214811	b	ooo+ooooo	null	null
214845	a	oo+++++++	DLEU1	deleted in lymphocytic leukemia, 1
214852	g	oooo-----	GTF2IRD2	null
214856	i	o-oo-----	C4orf13	chromosome 4 open reading frame 13
214913	h	ooooooo-o	TFDP3	transcription factor Dp family, member 3
214959	g	oo-------	TM2D3	TM2 domain containing 3
214997	h	oooo----o	RNF126	ring finger protein 126
215056	b	ooo+ooooo	null	null
215106	b	ooo++oooo	null	null
215130	a	o++++++++	DKFZP586P0123	null
215166	i	o-oo-----	ZNF383	zinc finger protein 383
215199	a	o++++++++	PCGF6	polycomb group ring finger 6
215220	b	oooooo+oo	FMNL1	formin-like 1
215225	b	ooo+ooooo	KIAA0317	KIAA0317
215251	a	ooo++++++	ATF1	activating transcription factor 1
215356	a	oooooo+++	null	null
215435	h	o-ooooooo	PLEKHG1	pleckstrin homology domain containing, family G (with RhoGef domain) member 1
215529	b	ooo++++oo	LOC283278	null
215540	b	ooo+ooooo	FLJ36032	null
215541	a	oo+++++++	ZC3HAV1	zinc finger CCCH-type, antiviral 1
215559	b	oooo+++oo	null	null
215597	b	ooo+ooooo	null	null
215619	b	ooo+ooooo	GTDC1	glycosyltransferase-like domain containing 1
215650	h	o-ooooooo	INSIG1	insulin induced gene 1
215656	b	ooo+ooooo	ITSN2	intersectin 2
215657	b	ooooo+ooo	LOC400097	null
215675	g	oooooooo-	KLHL18	kelch-like 18 (Drosophila)
215694	b	ooooooo+o	null	null
215702	b	oo+++++oo	EML2	echinoderm microtubule associated protein like 2
215824	i	o-----oo-	MRPL2	mitochondrial ribosomal protein L2
215830	b	ooo+ooooo	TMEM30A	transmembrane protein 30A
215854	b	oooo+oooo	LOC390003	null
215855	b	ooo+ooooo	PIP5K1B	phosphatidylinositol-4-phosphate 5-kinase, type I, beta
215909	g	oooooooo-	PGEA1	PKD2 interactor, golgi and endoplasmic reticulum associated 1
215946	b	oooooo+oo	PIM3	pim-3 oncogene
215960	g	o--------	SPAG6	sperm associated antigen 6

215964	b	ooooooo+o	null	null
215985	h	o-ooooooo	SSR3	signal sequence receptor, gamma (translocon-associated protein gamma)
216017	h	o-ooooooo	null	null
216019	b	ooo++oooo	C12orf4	chromosome 12 open reading frame 4
216047	b	ooo++++oo	MRPL55	mitochondrial ribosomal protein L55
216089	h	o-ooooooo	PIK3R3	phosphoinositide-3-kinase, regulatory subunit 3 (p55, gamma)
216108	b	oooooo+oo	C21orf33	chromosome 21 open reading frame 33
216319	a	ooooooo++	CXorf34	chromosome X open reading frame 34
216320	g	oooooo---	MAP4	microtubule-associated protein 4
216334	h	ooooooo-o	C20orf67	chromosome 20 open reading frame 67
216343	h	oooooo-oo	ZNF221	zinc finger protein 221
216349	b	oooooo++o	C1orf173	chromosome 1 open reading frame 173
216353	c	ooo+o++++	ADAMTS1	ADAM metallopeptidase with thrombospondin type 1 motif, 1
216357	g	o--------	LTF	lactotransferrin
216394	a	ooo++++++	HERC1	hect (homologous to the E6-AP (UBE3A) carboxyl terminus) domain and RCC1 (CHC1)-like domain (RLD) 1
216437	a	o++++++++	HORMAD1	HORMA domain containing 1
216440	h	o----oooo	C17orf48	chromosome 17 open reading frame 48
216472	b	ooo+ooooo	PDPR	null
216473	b	ooo+ooooo	DNAH1	dynein, axonemal, heavy polypeptide 1
216481	b	ooo+ooooo	HOOK3	hook homolog 3 (Drosophila)
216519	b	oooooo+oo	TMEM100	transmembrane protein 100
216566	b	ooo+ooooo	ITPR3	inositol 1,4,5-triphosphate receptor, type 3
216584	g	ooooo----	VPS13C	vacuolar protein sorting 13C (yeast)
216597	b	ooooooo+o	P2RX4	purinergic receptor P2X, ligand-gated ion channel, 4
216600	h	o-----ooo	NR4A1	nuclear receptor subfamily 4, group A, member 1
216615	a	oo+++++++	ABCA11	ATP-binding cassette, sub-family A (ABC1), member 11 (pseudogene)
216668	b	ooo+ooooo	COPG2	coatomer protein complex, subunit gamma 2
216696	a	ooo++++++	MYO10	myosin X
216699	b	ooo+ooooo	AGTPBP1	ATP/GTP binding protein 1
216718	b	ooo+ooooo	ITPKA	inositol 1,4,5-trisphosphate 3-kinase A
216721	g	o--------	C20orf74	chromosome 20 open reading frame 74
216775	a	ooo++++++	TNFAIP2	tumor necrosis factor, alpha-induced protein 2
216786	g	oooooooo-	PTOV1	prostate tumor overexpressed gene 1
216796	b	ooo+ooooo	null	null
216857	b	ooooooo+o	C14orf39	chromosome 14 open reading frame 39
216870	c	oo++oooo+	ST6GAL2	ST6 beta-galactosamide alpha-2,6-sialyltranferase 2
216962	b	ooooooo+o	RBBP9	retinoblastoma binding protein 9
216980	b	ooooo+ooo	SRCAP	null
216992	b	ooo+ooooo	CTPS2	CTP synthase II
216999	b	ooo+ooooo	C20orf100	chromosome 20 open reading frame 100
217029	a	ooo++++++	null	null
217138	a	ooo++++++	null	null
217145	a	oooooo+++	ERRFI1	ERBB receptor feedback inhibitor 1
217164	b	ooooooo+o	null	null
217170	g	oooooooo-	POLE2	polymerase (DNA directed), epsilon 2 (p59 subunit)
217233	b	ooo+ooooo	null	null
217237	h	o-ooooooo	GTF2H3	general transcription factor IIH, polypeptide 3, 34kDa
217273	b	ooo+ooooo	ZNF218	zinc finger protein 218
217302	c	ooo+o++++	NUP98	nucleoporin 98kDa
217310	b	oooo++ooo	null	null
217358	i	o--o-----	PRKCBP1	protein kinase C binding protein 1
217426	b	ooooo+ooo	null	null
217443	a	oooooo+++	TUBA3	null
217451	b	oooo+oooo	C21orf86	chromosome 21 open reading frame 86
217468	b	ooo+ooooo	VprBP	null
217488	g	oooooooo-	GADD45GIP1	growth arrest and DNA-damage-inducible, gamma interacting protein 1
217502	b	o+ooooooo	FLJ31166	null
217527	b	oooo+oooo	null	null
217533	h	oooo-oooo	DDX31	DEAD (Asp-Glu-Ala-Asp) box polypeptide 31
217568	b	ooo+ooooo	PHF17	PHD finger protein 17
217629	b	ooooo+ooo	SAMD6	sterile alpha motif domain containing 6
217732	b	ooooooo+o	null	null
217747	b	ooooooo+o	null	null
217832	b	oooooo+oo	MGC45491	null
217865	d	ooo+ooo--	IFT81	intraflagellar transport 81 homolog (Chlamydomonas)
217932	c	ooo+o++++	SON	SON DNA binding protein
217934	b	ooo+++++o	RALGPS1	Ral GEF with PH domain and SH3 binding motif 1
217972	b	ooo++oooo	SP2	Sp2 transcription factor
218013	b	ooo+ooooo	LOC389562	null
218065	b	ooooo++oo	MTMR1	myotubularin related protein 1

218080	b	ooooo+ooo	ZNF233	zinc finger protein 233
218083	c	ooo+ooo++	MAFG	v-maf musculoaponeurotic fibrosarcoma oncogene homolog G (avian)
218109	h	oooo----o	null	null
218140	b	ooo+ooooo	PGBD3	piggyBac transposable element derived 3
218145	a	ooooo++++	BCL2L11	BCL2-like 11 (apoptosis facilitator)
218216	b	ooo+ooooo	null	null
218234	b	ooooooo+o	null	null
218304	b	o+ooooooo	CDC42SE1	CDC42 small effector 1
218348	i	o---o----	PCDHGA2	protocadherin gamma subfamily A, 2
218387	a	oooooooo+	KIAA1618	KIAA1618
218400	a	ooooo++++	null	null
218403	b	oooooo+oo	ADPRHL1	ADP-ribosylhydrolase like 1
218419	a	o++++++++	FBXO38	F-box protein 38
218433	a	ooo++++++	FLJ31715	null
218515	a	ooooooo++	TTC17	tetratricopeptide repeat domain 17
218524	b	oo+oooooo	PCDHGA11	protocadherin gamma subfamily A, 11
218655	h	oooo----o	null	null
218680	b	ooo+ooooo	FBXO32	F-box protein 32
218683	a	ooooooo++	null	null
218703	h	oooo----o	null	null
218747	g	o--------	ASB18	ankyrin repeat and SOCS box-containing 18
218762	g	o--------	null	null
218810	a	o++++++++	IL1RAP	interleukin 1 receptor accessory protein
218814	b	oooo+oooo	ENDOGL1	endonuclease G-like 1
218912	b	o+ooooooo	IQCE	IQ motif containing E
218928	g	o--------	TWIST2	twist homolog 2 (Drosophila)
218945	g	ooooooo--	LOC128977	null
218952	a	ooo++++++	FLJ25006	null
218989	b	oooo+oooo	KIAA1797	KIAA1797
219119	b	oooo+oooo	null	null
219138	a	o++++++++	PDE4D	phosphodiesterase 4D, cAMP-specific (phosphodiesterase E3 dunce homolog, Drosophila)
219201	b	ooo+ooooo	WHSC1	Wolf-Hirschhorn syndrome candidate 1
219235	h	o-------o	null	null
219358	c	oo++oooo+	null	null
219363	b	oooo+oooo	null	null
219378	g	o--------	HIPK1	homeodomain interacting protein kinase 1
219396	g	ooooooo--	null	null
219397	a	oo+++++++	C14orf50	chromosome 14 open reading frame 50
219405	b	ooo+ooooo	RAB6IP2	RAB6 interacting protein 2
219421	b	ooo+ooooo	KLHL18	kelch-like 18 (Drosophila)
219466	b	ooo+ooooo	PBEF1	pre-B-cell colony enhancing factor 1
219548	a	ooo++++++	LOC348801	null
219582	j	o-oooo+++	UPP1	uridine phosphorylase 1
219649	h	o-ooooooo	FLJ31306	null
219663	i	o-oo-----	LUZP2	leucine zipper protein 2
219717	h	o--oooooo	ETV5	ets variant gene 5 (ets-related molecule)
219737	a	oo+++++++	TMEM99	transmembrane protein 99
219817	a	oooooooo+	HOXD4	homeobox D4
219851	h	oooo----o	null	null
219911	b	oooo+oooo	PSMAL	null
219964	a	oo+++++++	DOK3	docking protein 3
219968	b	oooo+oooo	null	null
220345	g	o--------	APOL4	apolipoprotein L, 4
220360	b	ooo+ooooo	MGC24103	null
220364	a	oooo+++++	null	null
220381	h	oooooo-oo	EYA2	eyes absent homolog 2 (Drosophila)
220448	g	o--------	null	null
220463	g	o--------	null	null
220485	c	ooooo+o++	LOC283861	null
220495	b	ooo+ooooo	FAM35A|RP11-38L15.1	family with sequence similarity 35, member A
220507	b	ooooo+ooo	FLJ25770	null
220527	g	o--------	LOC92691	null
220528	h	oooo---oo	HFM1	HFM1, ATP-dependent DNA helicase homolog (S. cerevisiae)
220550	b	ooo++++oo	FLJ12986	null
220556	k	o--+ooooo	SLC11A2	solute carrier family 11 (proton-coupled divalent metal ion transporters), member 2
220574	b	oooooo+oo	RASAL2	RAS protein activator like 2
220575	b	oooo+oooo	ATM	ataxia telangiectasia mutated (includes complementation groups A, C and D)
220588	g	o--------	RP11-393H10.2	null

220620	b	ooooo+ooo	null	null
220630	g	oo-------	null	null
220684	f	oo++----+	C1orf61	chromosome 1 open reading frame 61
220695	b	ooooooo+o	EPHA6	EPH receptor A6
220708	b	ooo+++ooo	C1orf24	chromosome 1 open reading frame 24
220713	b	oooo+oooo	null	null
220741	g	oooooooo-	GPR107	G protein-coupled receptor 107
220888	g	o--------	LOC390003	null
220934	b	ooooo+ooo	DGKB	diacylglycerol kinase, beta 90kDa
220939	h	oooo----o	MGC27165|IGHA1|IGHA2	immunoglobulin heavy constant alpha 1|immunoglobulin heavy constant alpha 2 (A2m marker)
221014	b	ooo+ooooo	null	null
221017	g	oooo-----	LRP8	low density lipoprotein receptor-related protein 8, apolipoprotein e receptor
221030	b	ooooooo+o	KLHDC1	kelch domain containing 1
221051	b	ooo++oooo	SLC29A4	solute carrier family 29 (nucleoside transporters), member 4
221134	c	oo++o++++	C6orf167	chromosome 6 open reading frame 167
221164	a	ooo++++++	GNAS	GNAS complex locus
221231	b	ooooooo+o	null	null
221257	b	ooooo+ooo	C14orf148	chromosome 14 open reading frame 148
221264	h	o-ooooooo	RP11-98F14.6	null
221305	a	ooooo++++	null	null
221503	b	oooooo+oo	GBP3	guanylate binding protein 3
221568	b	ooo+ooooo	PTPRE	protein tyrosine phosphatase, receptor type, E
221608	h	o-ooooooo	NUP107	nucleoporin 107kDa
221617	a	oo+++++++	SFRS3	splicing factor, arginine/serine-rich 3
221618	a	oooooo+++	FAM71A	family with sequence similarity 71, member A
221631	b	ooooo+ooo	SLC1A7	solute carrier family 1 (glutamate transporter), member 7
221772	a	ooo++++++	MGC16385	null
221867	h	oooooo-oo	MBTD1	mbt domain containing 1
221921	b	ooo+ooooo	PLXNA2	plexin A2
222107	h	oooooo--o	null	null
222162	a	ooo++++++	SLC45A4	solute carrier family 45, member 4
222165	h	o-ooooooo	NHN1	null
222219	b	ooo+ooooo	N4BP1	null
222222	b	ooooo+ooo	null	null
222226	b	ooo+ooooo	FLJ46156	null
222234	b	ooo+ooooo	PAPPA	pregnancy-associated plasma protein A, pappalysin 1
222249	h	oooo-oooo	C1orf65	chromosome 1 open reading frame 65
222315	b	oo+oooooo	NOX1	NADPH oxidase 1
222342	b	ooo+ooooo	MGC18216	null
222377	i	ooooo-o--	RP4-694E4.2	null
222408	b	oooo+oooo	SLC35D3	solute carrier family 35, member D3
222445	a	oooooo+++	HIST1H4H	histone 1, H4h
222503	g	ooooooo--	SBF2	SET binding factor 2
222506	f	oo++----+	FLJ46247	null
222540	b	o+ooooooo	null	null
222543	b	ooo+ooooo	PCDHA13	protocadherin alpha 13
222558	b	ooo+ooooo	LIN10	lin-10 homolog (C. elegans)
222569	b	oooooo++o	MCM3APAS	MCM3 minichromosome maintenance deficient 3 (S. cerevisiae) associated protein antisense
222604	h	oooo----o	ZNF570	zinc finger protein 570
222650	b	ooo+ooooo	LOC346702	null
222664	h	o-ooooooo	USP48	ubiquitin specific peptidase 48
222675	f	oo++----+	KIAA1843	null
222678	c	oo++oooo+	null	null
222729	a	oooo+++++	TRIM4	tripartite motif-containing 4
222733	a	ooooo++++	null	null
222747	b	ooo+ooooo	null	null
222773	g	o--------	RNF165|C18orf23	ring finger protein 165|chromosome 18 open reading frame 23
222840	a	ooo++++++	C10orf47	chromosome 10 open reading frame 47
222870	g	oooooooo-	MGC43122	null
222887	b	oooooo+oo	null	null
222896	b	ooooo+ooo	LOC399978	null
222925	h	o--oooooo	LOC552889	null
222926	i	o-oo-----	PELO	pelota homolog (Drosophila)
222936	b	o+ooooooo	RAI1	retinoic acid induced 1
222939	b	ooo+ooooo	null	null
222973	c	oo++oooo+	null	null
223006	b	ooo+ooooo	CHM	choroideremia (Rab escort protein 1)
223019	b	ooooooo+o	SCD5	stearoyl-CoA desaturase 5
223112	b	ooooooo+o	null	null

223116	a	oooo+++++	FAM24B	family with sequence similarity 24, member B
223180	a	oo+++++++	DKFZp586I1420	null
223183	g	ooooo----	KLHL5	kelch-like 5 (Drosophila)
223210	b	ooooooo+o	null	null
223217	h	o-ooooooo	TBPL1	TBP-like 1
223234	b	oo+oooooo	FLJ32894	null
223324	a	oooo+++++	LOC541472	null
223349	b	oooo+oooo	null	null
223425	b	ooooooo+o	null	null
223442	b	oooo+oooo	ITGA2	integrin, alpha 2 (CD49B, alpha 2 subunit of VLA-2 receptor)
223459	a	ooooooo++	HIST1H2BG	histone 1, H2bg
223669	f	oo++----+	null	null
223696	a	oooo+++++	null	null
223705	b	ooo+ooooo	UBE2D4	ubiquitin-conjugating enzyme E2D 4 (putative)
223728	b	oooo+oooo	SNX24	sorting nexing 24
223759	g	o--------	SYNGR1	synaptogyrin 1
223856	g	ooo------	MT1E	metallothionein 1E (functional)
223897	b	ooooooo+o	OLFM1	olfactomedin 1
223944	a	ooo++++++	ZAK	null
223950	g	o--------	PRO1483	null
223958	g	o--------	POFUT1	protein O-fucosyltransferase 1
223990	b	ooo+ooooo	NEUROG2	neurogenin 2
224007	b	oooooo++o	MGC24125	null
224016	b	ooo+ooooo	null	null
224105	b	ooo+ooooo	NOL6	nucleolar protein family 6 (RNA-associated)
224176	b	oooooo+oo	KCNQ2	potassium voltage-gated channel, KQT-like subfamily, member 2
224195	b	ooooo+ooo	MGC35154	null
224218	b	ooo+ooooo	USH2A	Usher syndrome 2A (autosomal recessive, mild)
224221	b	ooo+ooooo	LYSMD4	LysM, putative peptidoglycan-binding, domain containing 4
224250	b	oooo+oooo	LOC123855	null
224256	b	ooo++oooo	LOC493860	null
224260	a	oooooooo+	LOC387715	null
224282	b	ooo+ooooo	NFRKB	nuclear factor related to kappaB binding protein
224297	b	oooo+oooo	LOC126248	null
224305	b	oo++ooooo	PAF1	Paf1, RNA polymerase II associated factor, homolog (S. cerevisiae)
224332	g	o--------	PHLDB1	pleckstrin homology-like domain, family B, member 1
224373	b	oooooo+oo	ZNF278	zinc finger protein 278
224379	a	ooo++++++	WDR55	WD repeat domain 55
224381	a	ooooooo++	HIST1H2AG	histone 1, H2ag
224460	b	oooo+oooo	C21orf117	chromosome 21 open reading frame 117
224495	b	o+ooooooo	null	null
224496	a	oo+++++++	COL27A1	collagen, type XXVII, alpha 1
224505	h	o-ooooooo	null	null
224549	f	oo++----+	null	null
224628	a	ooo++++++	ANKHD1	ankyrin repeat and KH domain containing 1
224634	b	oooooo++o	MGC3196	null
224642	a	ooo++++++	CSNK1A1	casein kinase 1, alpha 1
224645	b	ooo+ooooo	MDM1	Mdm4, transformed 3T3 cell double minute 1, p53 binding protein (mouse)
224660	b	ooo+ooooo	AGGF1	angiogenic factor with G patch and FHA domains 1
224677	b	ooo+ooooo	null	null
224728	b	ooo+ooooo	MESDC2	mesoderm development candidate 2
224737	b	oooo+oooo	IQCE	IQ motif containing E
224771	b	ooooooo+o	LLGL1	lethal giant larvae homolog 1 (Drosophila)
224775	h	o-ooooooo	null	null
224805	g	o--------	C8orf56	chromosome 8 open reading frame 56
224817	h	o-ooooooo	null	null
224837	a	ooo++++++	C10orf9	chromosome 10 open reading frame 9
224851	b	oo++ooooo	SVEP1	sushi, von Willebrand factor type A, EGF and pentraxin domain containing 1
224882	a	o++++++++	ALDH4A1	aldehyde dehydrogenase 4 family, member A1
224883	b	ooo+ooooo	AP1GBP1	AP1 gamma subunit binding protein 1
224908	h	oooooo-oo	RICS	null
224909	b	ooo++oooo	LOC132241	null
224935	b	ooo+ooooo	C21orf89	chromosome 21 open reading frame 89
224983	a	oooooooo+	11-Sep	septin 11
224991	b	oooooo+oo	null	null
225045	a	ooooo++++	TGIF	TGFB-induced factor (TALE family homeobox)
225051	f	oo++----+	null	null
225077	c	ooo+oo+++	C8orf58	chromosome 8 open reading frame 58
225079	b	oooo+oooo	null	null
225107	b	ooooooo+o	null	null
225120	h	oooo-oooo	PRDM2	PR domain containing 2, with ZNF domain

225155	g	ooooooo--	null	null
225165	g	oo-------	C11orf45	chromosome 11 open reading frame 45
225175	a	o++++++++	CXorf52	chromosome X open reading frame 52
225293	b	ooo+ooooo	IFNAR1	interferon (alpha, beta and omega) receptor 1
225333	a	oooooo+++	FLJ25801	null
225336	a	ooooooo++	TLOC1	translocation protein 1
225350	h	oooo----o	SRP9	signal recognition particle 9kDa
225354	b	ooooooo+o	MGC15885	null
225559	h	o-ooooooo	null	null
225588	b	oooooo+oo	RHOBTB1	Rho-related BTB domain containing 1
225603	b	ooo+ooooo	HPS4	Hermansky-Pudlak syndrome 4
225617	a	oo+++++++	HIST1H3H	histone 1, H3h
225648	f	oo++----+	USP24	ubiquitin specific peptidase 24
225677	b	oooo+oooo	FCN2	ficolin (collagen/fibrinogen domain containing lectin) 2 (hucolin)
225706	g	ooo------	null	null
225713	b	oooo+oooo	CD302	CD302 antigen
225764	b	oooo++ooo	TXNDC6	thioredoxin domain containing 6
225816	a	oo+++++++	C11orf47	chromosome 11 open reading frame 47
225854	b	o+ooooooo	TSPAN32	tetraspanin 32
225859	a	ooooooo++	GPR154	G protein-coupled receptor 154
225929	j	oooooo--+	C2orf15	chromosome 2 open reading frame 15
225953	b	oooooo++o	C14orf49	chromosome 14 open reading frame 49
225955	b	ooooo+ooo	OR2L13	olfactory receptor, family 2, subfamily L, member 13
225967	b	ooo+ooooo	null	null
226057	g	oo-------	null	null
226088	l	o--+-----	PCDHGA12	protocadherin gamma subfamily A, 12
226327	b	ooo+ooooo	CABP3|CABP5	calcium binding protein 3|calcium binding protein 5
226336	a	ooo++++++	MAFB	v-maf musculoaponeurotic fibrosarcoma oncogene homolog B (avian)
226343	g	oooo-----	null	null
226399	b	oooo+oooo	null	null
226439	b	ooo+ooooo	PPARA	peroxisome proliferative activated receptor, alpha
226514	b	ooo+ooooo	null	null
226554	b	ooooooo+o	null	null
226580	g	ooooo----	null	null
226588	b	o+ooooooo	C1orf65	chromosome 1 open reading frame 65
226658	h	oooo----o	VAMP4	vesicle-associated membrane protein 4
226686	g	oo-------	LRRC55	leucine rich repeat containing 55
226708	b	ooo+ooooo	null	null
226719	h	oooo----o	EDNRB	endothelin receptor type B
226725	g	o--------	null	null
226740	a	oo+++++++	RBM12	RNA binding motif protein 12
226744	b	ooo++oooo	P2RX2	purinergic receptor P2X, ligand-gated ion channel, 2
226834	f	oo++----+	null	null
226855	g	o--------	null	null
226866	c	oo++oooo+	null	null
226876	b	ooooo+ooo	HFE	hemochromatosis
226908	b	ooo+ooooo	PDPK1	3-phosphoinositide dependent protein kinase-1
226948	h	o-ooooooo	HIST1H4I	histone 1, H4i
227067	b	ooooooo+o	LOC400571	null
227204	a	o++++++++	MGC45800	null
227240	a	oo+++++++	LOC401431	null
227249	a	oooooooo+	ACSS1	acyl-CoA synthetase short-chain family member 1
227268	g	oooo-----	FLJ30901	null
227322	a	ooo++++++	WDR35	WD repeat domain 35
227323	i	o-----o--	VEGFB	vascular endothelial growth factor B
227341	a	ooooo++++	ERN1	endoplasmic reticulum to nucleus signalling 1
227404	b	ooo+ooooo	NRG1	neuregulin 1
227422	b	ooo+ooooo	null	null
227494	b	oooo+oooo	TCF21	transcription factor 21
227501	g	oooooooo-	PMF1	polyamine-modulated factor 1
227524	b	oooo+oooo	FLJ12610	null
227528	b	ooo+ooooo	null	null
227598	g	o--------	C18orf15	chromosome 18 open reading frame 15
227647	b	ooo+ooooo	ARPP-21	null
227694	a	ooo++++++	LCMT2	leucine carboxyl methyltransferase 2
227696	b	ooo+ooooo	GIT2	G protein-coupled receptor kinase interactor 2
227710	b	ooooooo+o	ZNF273	zinc finger protein 273
227734	h	oooooo-oo	null	null
227740	a	o++++++++	CLTCL1	clathrin, heavy polypeptide-like 1
227752	b	oooooo+oo	NBEAL1	neurobeachin-like 1
227814	a	o++++++++	MKRN1	makorin, ring finger protein, 1

227837	b	oooooo+oo	null	null
227856	h	o-ooooooo	NR1D2	nuclear receptor subfamily 1, group D, member 2
227876	b	oooo+oooo	LOC441736	null
227899	b	ooooo+ooo	ZNF626	zinc finger protein 626
227969	b	oooo+oooo	null	null
228018	a	o++++++++	CHST11	carbohydrate (chondroitin 4) sulfotransferase 11
228023	c	ooooo+oo+	null	null
228040	g	o--------	null	null
228051	f	oo++----+	LOC139193	null
228076	a	oooooooo+	null	null
228086	b	oooooo+oo	LOC285628	null
228105	b	ooo+ooooo	SFTPG	surfactant associated protein G
228107	a	ooo++++++	LOC257358	null
228126	b	oooo+oooo	null	null
228171	b	ooooooo+o	null	null
228174	g	oo-------	LOC152225	null
228181	c	oo++oooo+	null	null
228222	j	oooo----+	null	null
228223	g	o--------	null	null
228234	b	ooo+ooooo	ATP6V0A2	ATPase, H+ transporting, lysosomal V0 subunit a isoform 2
228255	a	ooo++++++	null	null
228267	h	oooo----o	null	null
228273	i	o---o----	null	null
228328	a	oo+++++++	LOC153684	null
228337	b	oooo+oooo	LOC554174	null
228344	h	oooo----o	LOC439967|ANTXRL	anthrax toxin receptor-like
228351	b	ooooooo+o	DHRS10	dehydrogenase/reductase (SDR family) member 10
228354	h	ooooooo-o	LOC389385	null
228361	b	ooooo+ooo	null	null
228373	b	ooo+ooooo	BCAN	brevican
228385	a	oooooooo+	LOC392554	null
228400	b	ooo+ooooo	null	null
228420	b	oooo+oooo	null	null
228477	b	o+ooooooo	ADCY1	adenylate cyclase 1 (brain)
228494	b	ooooo+ooo	null	null
228502	f	oo++----+	null	null
228513	b	ooo+ooooo	null	null
228528	c	ooo+o++++	null	null
228532	g	oooooooo-	LOC441113	null
228536	a	oo+++++++	null	null
228609	b	ooo+ooooo	null	null
228627	b	ooooo+ooo	null	null
228632	i	oo-oo----	null	null
228636	a	oooooooo+	null	null
228665	g	ooooooo--	null	null
228686	i	oo---o---	LOC284998	null
228692	a	o++++++++	null	null
228721	h	oooo-oooo	ZNF616	zinc finger protein 616
228722	h	oooo----o	CXorf36	chromosome X open reading frame 36
228727	h	o-ooooooo	null	null
228734	b	oooooo+oo	FLJ33297	null
228749	f	oo++----+	null	null
228763	b	oooo+oooo	ARVCF	armadillo repeat gene deletes in velocardiofacial syndrome
228780	i	o-oo-----	null	null
228823	b	ooo+ooooo	null	null
228835	b	oooo+oooo	null	null
228856	h	oooooo-oo	null	null
228862	b	oooo++ooo	null	null
228872	f	oo++----+	null	null
228879	b	ooo+ooooo	null	null
228921	h	oooo----o	LOC400764	null
228934	b	ooooooo+o	LOC400986	null
228943	b	ooo+ooooo	null	null
228950	b	ooo+ooooo	null	null
228955	b	ooooooo+o	null	null
228967	a	o++++++++	LOC149134	null
229010	a	ooooo++++	TALDO1|HSUP1	transaldolase 1
229057	b	ooooo+ooo	null	null
229058	g	oooooooo-	FLJ46111	null
229068	b	ooo+ooooo	LOC256273	null

229072	g	o--------	DKFZp686L13185	null
229106	b	ooo+ooooo	LOC255177	null
229116	b	ooooo+ooo	LOC388763	null
229124	b	ooo+ooooo	null	null
229131	a	ooo++++++	LOC285193	null
229144	a	ooo++++++	null	null
229172	b	ooo+ooooo	null	null
229173	a	ooooooo++	null	null
229178	g	oooo-----	null	null
229185	b	ooo+ooooo	null	null
229198	i	o-oo-----	MGC51025|FLJ40244	null
229212	b	oo+oooooo	null	null
229219	g	o--------	null	null
229261	b	ooooooo+o	FLJ39005	null
229290	g	o--------	null	null
229297	b	ooo++oooo	FLJ90086	null
229338	a	oo+++++++	null	null
229342	a	oooooo+++	LOC389910|LOC441528|LOC389906	null
229345	b	ooo+ooooo	null	null
229347	b	oooo+oooo	null	null
229354	b	ooo+ooooo	GRAMD2	GRAM domain containing 2
229425	c	ooo+o++++	null	null
229472	f	oo++----+	null	null
229552	h	o-ooooooo	null	null
229581	i	o-oo-----	null	null
229582	b	ooo++++oo	KRTAP10-11	keratin associated protein 10-11
229584	b	oooooo+oo	null	null
229634	g	ooooooo--	null	null
229637	b	oooooo+oo	ZNF471	zinc finger protein 471
229651	g	o--------	null	null
229653	h	oooo----o	null	null
229668	a	oo+++++++	null	null
229673	b	oooo+oooo	CD33L3	CD33 antigen-like 3
229674	b	oooooo+oo	null	null
229726	b	ooo++++oo	null	null
229771	b	oooo++ooo	null	null
229786	c	ooo+oo+++	LOC401463	null
229808	g	oooooooo-	LOC400730	null
229838	g	ooooooo--	null	null
229863	g	ooooo----	LOC284749	null
229864	b	oooo+oooo	null	null
229884	g	o--------	null	null
229942	b	ooo+ooooo	null	null
229969	i	o-oo-----	LOC401097	null
229979	b	ooooooo+o	null	null
229985	a	oo+++++++	null	null
229995	b	o+ooooooo	FLJ20433	null
230003	i	o-oo-----	null	null
230034	b	ooo+ooooo	null	null
230054	g	oooooooo-	LOC344382	null
230057	b	ooooooo+o	null	null
230119	b	ooo+ooooo	SYPL2	synaptophysin-like 2
230144	h	ooooooo-o	null	null
230167	g	o--------	null	null
230171	b	ooooooo+o	null	null
230173	b	ooo+ooooo	null	null
230176	h	oooo----o	LOC440418	null
230181	b	ooooo+ooo	LOC401234	null
230212	b	ooo++oooo	null	null
230227	a	o++++++++	null	null
230238	b	ooo+ooooo	WBSCR16	Williams-Beuren syndrome chromosome region 16
230239	b	ooo+ooooo	null	null
230257	h	o-ooooooo	DKFZp434B1231	null
230275	b	oooo+oooo	null	null
230281	g	oo-------	null	null
230284	b	ooo+ooooo	null	null
230288	b	ooo+ooooo	null	null
230292	b	ooo++oooo	C9orf102	chromosome 9 open reading frame 102
230326	g	o--------	null	null

230336	d	ooo+ooo--	C16orf44	chromosome 16 open reading frame 44
230343	a	ooooooo++	RRN3	RRN3 RNA polymerase I transcription factor homolog (yeast)
230344	g	o--------	null	null
230382	h	ooooooo-o	null	null
230411	a	ooo++++++	null	null
230414	b	ooo+ooooo	LOC149448	null
230419	f	oo++----+	null	null
230432	a	o++++++++	LOC222699	null
230448	g	o--------	LUZP5	leucine zipper protein 5
230474	b	ooooo+ooo	null	null
230484	h	oooooo-oo	null	null
230490	b	ooo+ooooo	null	null
230543	b	ooooo+ooo	LOC283585	null
230545	b	ooo++oooo	null	null
230552	b	ooooooo+o	ITGAX	integrin, alpha X (antigen CD11C (p150), alpha polypeptide)
230569	g	oooo-----	null	null
230584	b	ooooooo+o	FLJ12448	null
230585	e	oo+++-ooo	null	null
230587	a	oooooooo+	null	null
230604	c	ooo+oooo+	PHF21A	PHD finger protein 21A
230610	i	oooo--o--	null	null
230663	b	oooooo+oo	null	null
230676	g	ooooo----	null	null
230688	b	ooooooo+o	null	null
230720	b	ooooo+ooo	LOC285043	null
230763	a	oo+++++++	LOC116349	null
230770	g	o--------	null	null
230772	b	ooooooo+o	null	null
230775	b	o+ooooooo	SYT9	synaptotagmin IX
230791	a	oo+++++++	LOC389289	null
230810	b	oo+oooooo	null	null
230823	c	ooo++o+++	null	null
230825	b	oooooo+oo	null	null
230831	b	oooo++ooo	null	null
230842	b	oooo+oooo	RBM33	RNA binding motif protein 33
230913	b	oooooo+oo	null	null
230919	b	ooooooo+o	null	null
230924	b	oo++ooooo	null	null
230940	h	ooooo--oo	TPM1	tropomyosin 1 (alpha)
230968	i	o------o-	null	null
230984	g	o--------	null	null
230995	b	ooooooo+o	null	null
231083	a	oo+++++++	C10orf75	chromosome 10 open reading frame 75
231085	i	o-oo-----	LOC341356	null
231094	b	ooo+ooooo	LOC402573	null
231095	b	oooooo+oo	BPIL2	bactericidal/permeability-increasing protein-like 2
231117	a	ooo++++++	null	null
231119	b	oooooo+oo	null	null
231155	b	ooooooo+o	null	null
231177	b	ooooo+ooo	null	null
231197	b	oooo+oooo	null	null
231223	i	o------o-	null	null
231267	a	ooooo++++	null	null
231268	c	oo++oooo+	EME2	essential meiotic endonuclease 1 homolog 2 (S. pombe)
231277	a	ooooo++++	null	null
231286	g	oo-------	null	null
231337	b	oooooo+oo	null	null
231372	h	o----oooo	LOC388545|ZA20D2	zinc finger, A20 domain containing 2
231382	i	o------o-	null	null
231388	b	ooo+ooooo	null	null
231444	g	oooooooo-	FLJ42291	null
231452	b	ooooo+ooo	null	null
231458	g	o--------	null	null
231461	b	ooo+ooooo	C6orf120	chromosome 6 open reading frame 120
231479	b	oooo+oooo	FLJ25415	null
231497	c	ooo+oo+++	null	null
231565	b	ooo++++oo	null	null
231615	h	ooooo-ooo	null	null
231631	b	ooo++++oo	FLJ32867	null
231656	b	ooo+ooooo	null	null

231679	h	oooo----o	KIAA0738	null
231681	a	o++++++++	null	null
231699	b	ooo+ooooo	MAN1A2	mannosidase, alpha, class 1A, member 2
231700	b	ooooooo+o	LOC93463	null
231708	i	o--o-----	null	null
231713	g	o--------	PRDM10	PR domain containing 10
231740	f	oo++----+	LOC441993	null
231799	b	ooooo+ooo	null	null
231829	b	oooooo+oo	null	null
231838	b	o+ooooooo	FLJ39609	null
231856	a	ooooooo++	TUSC1	tumor suppressor candidate 1
231862	b	ooo+ooooo	ACADSB	acyl-Coenzyme A dehydrogenase, short/branched chain
231869	g	o--------	TLN2	talin 2
231885	i	ooo----o-	C14orf25	chromosome 14 open reading frame 25
231895	a	oo+++++++	KIAA1026	null
231899	c	oo++oooo+	null	null
231923	b	oooo+oooo	KIAA1171	null
231929	b	oooooo+oo	LOC388760	null
231933	b	oooo+oooo	null	null
231956	b	ooo+ooooo	LOC391298|LOC440795|LOC402036	null
231984	h	ooooo-ooo	NIT2	nitrilase family, member 2
232013	b	ooo+ooooo	FBXO41	F-box protein 41
232054	h	oooooo-oo	null	null
232057	a	oo+++++++	FLJ39075	null
232070	b	ooo+ooooo	null	null
232084	b	oooooo+oo	null	null
232089	b	ooooooo+o	null	null
232109	b	o+ooooooo	null	null
232117	b	oo+oooooo	null	null
232123	a	ooooooo++	ZNF707	zinc finger protein 707
232162	h	oooo--ooo	null	null
232166	b	ooo+ooooo	LOC440845|TTC15	tetratricopeptide repeat domain 15
232191	b	ooooo+ooo	C8orf68	chromosome 8 open reading frame 68
232203	c	o+oooooo+	TSPAN4	tetraspanin 4
232206	b	ooo++oooo	null	null
232208	b	ooooo+ooo	null	null
232209	b	ooooo+ooo	LOC388284	null
232211	b	ooo+ooooo	null	null
232218	b	ooo+ooooo	null	null
232220	h	o-ooooooo	PTDSR	phosphatidylserine receptor
232228	h	oooo----o	CREB5	cAMP responsive element binding protein 5
232247	b	oooooo+oo	null	null
232254	b	oooooo+oo	null	null
232259	b	ooo+ooooo	null	null
232260	c	oo++oooo+	null	null
232291	i	o-oo-----	null	null
232322	b	ooo+ooooo	null	null
232327	b	ooo+ooooo	ASB6	ankyrin repeat and SOCS box-containing 6
232328	a	oooooooo+	null	null
232356	h	o-ooooooo	null	null
232363	a	oo+++++++	FLJ39575	null
232387	h	oooo----o	BRCC2	null
232403	b	oooo+oooo	LOC253805	null
232428	b	oooo+oooo	PTEN	phosphatase and tensin homolog (mutated in multiple advanced cancers 1)
232445	a	ooooooo++	null	null
232455	b	oo+oooooo	EHMT1	euchromatic histone-lysine N-methyltransferase 1
232465	i	o--ooooo-	null	null
232506	b	ooooooo+o	LOC286083	null
232507	b	oooo+oooo	H6PD	hexose-6-phosphate dehydrogenase (glucose 1-dehydrogenase)
232524	a	oo+++++++	null	null
232527	a	o++++++++	NUP153	nucleoporin 153kDa
232548	a	ooo++++++	LOC283377	null
232587	a	oo+++++++	FLJ38028	null
232606	b	ooooo+ooo	null	null
232623	c	oo++o++++	null	null
232629	b	ooooooo+o	null	null
232642	b	ooo+ooooo	null	null
232665	b	oooo+oooo	null	null
232666	i	o---o----	null	null
232671	b	oooooo+oo	null	null

232672	b	oo+oooooo	null	null
232673	b	oooo+oooo	LOC283487	null
232695	b	o+ooooooo	LOC147004	null
232704	b	ooooooo+o	C14orf58	chromosome 14 open reading frame 58
232729	g	o--------	null	null
232764	g	oooo-----	null	null
232769	b	oooo+oooo	null	null
232774	k	ooooo-+oo	null	null
232783	b	ooooooo+o	null	null
232901	b	o+ooooooo	LOC283486	null
232908	b	ooo++oooo	C20orf112	chromosome 20 open reading frame 112
232918	b	ooooooo+o	LOC400620	null
232929	g	oooooooo-	null	null
232964	b	ooooooo+o	null	null
232976	c	ooo+++o++	C10orf54	chromosome 10 open reading frame 54
232980	b	ooo+ooooo	NDST1	N-deacetylase/N-sulfotransferase (heparan glucosaminyl) 1
232999	a	ooo++++++	null	null
233035	a	oooooooo+	null	null
233042	i	oooo-o---	LOC146517	null
233046	g	o--------	null	null
233055	h	oooo--ooo	null	null
233058	b	oooooo+oo	null	null
233095	b	oooo+oooo	null	null
233114	b	oooo+oooo	null	null
233118	g	o--------	null	null
233145	h	ooooooo-o	C14orf43	chromosome 14 open reading frame 43
233153	h	oooo----o	LOC144742	null
233189	b	ooo+++ooo	null	null
233221	a	oooooooo+	null	null
233232	a	oooooooo+	C17orf60	chromosome 17 open reading frame 60
233275	b	ooo+ooooo	null	null
233278	g	oooooooo-	null	null
233304	b	ooooooo+o	null	null
233305	b	ooo++oooo	null	null
233312	a	ooo++++++	LOC400027	null
233346	b	ooooo+ooo	KLHDC2	kelch domain containing 2
233348	b	oo++ooooo	MGC10850	null
233364	a	ooooo++++	ID2B	inhibitor of DNA binding 2B, dominant negative helix-loop-helix protein
233366	g	o--------	null	null
233375	g	oooooooo-	LOC203510	null
233402	g	o--------	LOC285501	null
233419	h	oooo----o	null	null
233456	b	oo+oooooo	null	null
233460	h	oooooo--o	null	null
233475	g	o--------	null	null
233507	b	ooo+ooooo	PRKCA	protein kinase C, alpha
233520	h	oooo----o	null	null
233549	g	o--------	null	null
233554	b	ooo+++ooo	DKFZp451A211	null
233576	i	o-oo-----	null	null
233618	b	ooo+ooooo	C1orf188	chromosome 1 open reading frame 188
233658	b	oooo+oooo	null	null
233668	b	oooo+oooo	null	null
233702	a	ooo++++++	LOC199800	null
233710	b	ooo++oooo	null	null
233714	g	oooooo---	null	null
233716	b	ooo+ooooo	ZNF629	zinc finger protein 629
233786	b	oooo+oooo	null	null
233795	b	ooooooo+o	null	null
233835	g	oooooooo-	LOC388564	null
233838	b	ooooooo+o	NHS	Nance-Horan syndrome (congenital cataracts and dental anomalies)
233860	b	ooooooo+o	null	null
233864	b	ooooo+ooo	null	null
233881	b	ooooo+ooo	LOC121838	null
233900	a	ooo++++++	PHIP	pleckstrin homology domain interacting protein
233906	h	ooooooo-o	null	null
233928	a	ooo++++++	LOC153546	null
233933	b	ooooo+ooo	NBPF3	neuroblastoma breakpoint family, member 3
233955	c	oo++oooo+	KRTAP12-2	keratin associated protein 12-2
233967	a	oooooooo+	null	null
233986	b	oooooo+oo	null	null

234009	b	ooo+ooooo	LOC284214	null
234020	g	o--------	KIAA1698	KIAA1698
234026	a	ooo++++++	CRAMP1L	Crm, cramped-like (Drosophila)
234041	h	ooo-ooooo	null	null
234049	b	oo+oooooo	HSPC268	null
234061	b	ooooo+ooo	null	null
234092	b	oooo+oooo	null	null
234106	f	oo++----+	null	null
234119	b	ooo+ooooo	null	null
234132	a	oooooooo+	CLDN4	claudin 4
234176	b	ooooo+ooo	LOC391636	null
234186	h	o-ooooooo	null	null
234189	g	oo-------	null	null
234197	g	ooooooo--	null	null
234211	b	ooooooo+o	null	null
234214	b	ooo+ooooo	null	null
234216	a	oooooooo+	KIAA1193	KIAA1193
234266	a	ooooooo++	IBRDC2	IBR domain containing 2
234327	c	oo+o+++++	null	null
234339	b	ooo+ooooo	null	null
234388	a	ooo++++++	RFPL1S	ret finger protein-like 1 antisense
234389	b	ooo+ooooo	null	null
234449	b	oooooo+oo	null	null
234451	a	ooooo++++	null	null
234456	h	o-ooooooo	SBNO1	sno, strawberry notch homolog 1 (Drosophila)
234493	b	ooo+ooooo	C10orf74	chromosome 10 open reading frame 74
234504	b	oooooo+oo	null	null
234514	b	ooo+ooooo	null	null
234519	a	oo+++++++	LOC284100	null
234537	a	oooooo+++	MAN2C1	mannosidase, alpha, class 2C, member 1
234549	b	ooo+ooooo	null	null
234555	g	o--------	null	null
234561	b	ooooo+ooo	null	null
234565	h	oooooo-oo	null	null
234570	a	oooooooo+	NAGA	N-acetylgalactosaminidase, alpha-
234584	g	oooo-----	null	null
234598	g	ooo------	null	null
234608	b	ooo+ooooo	MKL1	megakaryoblastic leukemia (translocation) 1
234658	b	o+ooooooo	null	null
234673	b	ooo++oooo	null	null
234743	b	ooooooo+o	FAM62C	family with sequence similarity 62 (C2 domain containing), member C
234754	c	oo++o++++	null	null
234758	a	ooooooo++	LOC284454	null
234770	b	ooo+ooooo	ZNF452	zinc finger protein 452
234781	b	ooo+ooooo	MGC27345	null
234801	b	ooo+ooooo	ABHD2|C11orf2	abhydrolase domain containing 2|chromosome 11 open reading frame2
234815	g	ooooo----	null	null
234839	g	oooo-----	null	null
234866	g	o--------	null	null
234878	g	ooo------	null	null
234888	b	ooo+++++o	null	null
234892	b	ooo++oooo	FMN1	formin 1
234900	g	o--------	null	null
234901	b	oooo+oooo	CLASP2	cytoplasmic linker associated protein 2
234903	b	ooo+ooooo	DACT2	dapper, antagonist of beta-catenin, homolog 2 (Xenopus laevis)
234904	a	oooooooo+	null	null
234907	i	o-oo-----	null	null
234913	h	oooooo-oo	null	null
234993	b	ooo+ooooo	null	null
235006	h	o-ooooooo	BAIAP2L2	BAI1-associated protein 2-like 2
235029	g	oooooooo-	KRT8	keratin 8
235043	b	ooo+ooooo	null	null
235044	i	o---o----	SOX6	SRY (sex determining region Y)-box 6
235058	b	ooooooo+o	null	null
235153	h	oooooo-oo	null	null
235164	b	oooo+oooo	null	null
235168	b	ooo+ooooo	null	null
235212	h	o-ooooooo	null	null
235214	i	o--o-----	CCDC33	coiled-coil domain containing 33
235227	g	o--------	null	null
235248	b	ooooo+ooo	C6orf85	chromosome 6 open reading frame 85

235257	h	oooooo-oo	CKMT2	creatine kinase, mitochondrial 2 (sarcomeric)
235259	b	oooo+oooo	PROCA1	null
235265	f	oo++----+	null	null
235294	b	oooo+oooo	null	null
235304	b	o+ooooooo	null	null
235317	b	ooooooo+o	DKFZp434M131	null
235336	g	oooooooo-	null	null
235362	a	oooooooo+	LOC400125	null
235419	f	oo++----+	C10orf81	chromosome 10 open reading frame 81
235477	b	ooo+ooooo	null	null
235481	f	oo++----+	C21orf15	chromosome 21 open reading frame 15
235482	c	oo++oooo+	null	null
235527	i	o--ooooo-	NUP188	nucleoporin 188kDa
235553	a	ooooo++++	null	null
235554	b	ooo+ooooo	PKD1L1	polycystic kidney disease 1 like 1
235624	b	ooo+ooooo	null	null
235636	a	ooo++++++	null	null
235637	b	ooo+ooooo	null	null
235673	i	o-oo-----	null	null
235699	a	ooo++++++	null	null
235700	h	oooo----o	null	null
235714	b	oooo+oooo	MGC39900	null
235716	b	ooo+ooooo	MYST2|LOC401606	MYST histone acetyltransferase 2
235726	b	ooo+ooooo	null	null
235745	b	oooo+oooo	null	null
235753	b	o+ooooooo	null	null
235754	b	o+ooooooo	ZNF396	zinc finger protein 396
235774	b	ooo+ooooo	null	null
235775	a	o++++++++	null	null
235777	b	ooo+ooooo	CTAGE4|LOC442780	CTAGE family, member 4
235805	b	ooo+ooooo	null	null
235812	i	o-ooo----	null	null
235820	a	oo+++++++	null	null
235830	b	ooooo+++o	ARSD	arylsulfatase D
235863	h	oooo----o	null	null
235877	a	ooo++++++	IQCB1	IQ motif containing B1
235893	f	oo++----+	null	null
235896	b	ooo+ooooo	null	null
235925	b	ooo+ooooo	C10orf26	chromosome 10 open reading frame 26
235930	b	ooo+++++o	null	null
235946	b	ooooooo+o	null	null
235984	b	ooo+ooooo	null	null
235991	a	oooooooo+	null	null
235995	b	ooo++oooo	null	null
236052	f	oo++----+	null	null
236056	b	ooo+ooooo	null	null
236106	g	o--------	null	null
236166	a	oooooo+++	null	null
236273	g	oooooooo-	null	null
236293	a	ooooooo++	null	null
236345	c	oo++oooo+	null	null
236353	c	oo++oooo+	null	null
236357	b	ooo+ooooo	null	null
236412	h	oooooo-oo	null	null
236452	d	oooo+ooo-	LOC441761	null
236479	d	ooo+oooo-	null	null
236501	b	ooo++oooo	null	null
236534	c	oooo+ooo+	null	null
236648	b	ooo+ooooo	LOC441655	null
236687	b	oooo+oooo	DKFZp686A1627	null
236707	i	o-oo-----	LOC400968	null
236897	h	o-ooooooo	null	null
236949	h	oooooo-oo	SPCS2P	signal peptidase complex subunit 2 homolog (S. cerevisiae) pseudogene
237001	a	ooo++++++	LOC401915	null
237020	b	oooooo+oo	null	null
237030	b	oooo+oooo	CHR415SYT	null
237056	b	ooo+ooooo	null	null
237098	a	ooo++++++	null	null
237385	b	oo++ooooo	null	null

239071	g	o--------	H2AFY	H2A histone family, member Y
239486	h	oooo-oooo	null	null
240102	b	ooo+ooooo	FLJ21616	null
240666	a	oo+++++++	null	null
240806	b	oo+oooooo	null	null
243758	h	ooo----oo	null	null
244965	a	ooooooo++	null	null
245221	g	o--------	LOC401480	null
245665	b	oooooo+oo	C10orf37|C6orf182	chromosome 10 open reading frame 37|chromosome 6 open reading frame 182
248889	b	oooooo++o	null	null
248921	b	oooo+oooo	LOC284581	null
254853	b	oooo+oooo	null	null
256249	b	oooo+oooo	null	null
257423	h	oooo----o	LOC339237	null
257718	g	o--------	null	null
257752	a	oo+++++++	LOC401619	null
259140	b	oooooo++o	null	null
261013	f	oo++----+	null	null
261069	b	oooo+oooo	FLJ38717	null
261236	b	ooo+ooooo	null	null
261411	b	oooo++ooo	C14orf78	chromosome 14 open reading frame 78
264394	b	oooooo+oo	FLJ00038|MGC52000|LOC349338|FLJ25222	null
264940	h	o-ooooooo	null	null
267421	h	oooo----o	ZNF468	null
268211	h	oooooo-oo	LOC144571	null
268286	b	oooo+oooo	null	null
270300	b	ooooooo+o	null	null
271955	a	oo+++++++	null	null
271995	g	o--------	CD200R2	null
272417	b	ooooo+ooo	null	null
272419	b	oooo+oooo	DEFT1P	defensin, theta 1 pseudogene
280200	b	ooooo+ooo	KBTBD4	kelch repeat and BTB (POZ) domain containing 4
282043	f	oo++----+	null	null
286070	g	oooooooo-	null	null
286511	a	oo+++++++	null	null
291775	b	ooo+ooooo	null	null
294369	b	ooo++++oo	LOC196549	null
295023	a	oo+++++++	null	null
295404	a	ooooo++++	null	null
297089	f	oo++----+	LOC442497	null
297119	b	oooo+oooo	null	null
297413	b	oooo+oooo	null	null
300613	b	ooo+ooooo	C13orf25	chromosome 13 open reading frame 25
303585	b	ooooo+ooo	LOC340094	null
304659	a	ooo++++++	SLC16A6|LOC440459	solute carrier family 16 (monocarboxylic acid transporters), member 6
304688	b	ooo+ooooo	BNC2	basonuclin 2
306070	a	oo+++++++	ZMYND17	zinc finger, MYND-type containing 17
309443	c	oo++oooo+	LOC441191	null
317027	a	oo+++++++	C3orf35	chromosome 3 open reading frame 35
326916	b	oooooo+oo	null	null
327287	b	oo+oooooo	null	null
331647	a	oo+++++++	SAPS2	SAPS domain family, member 2
332645	b	o+ooooooo	null	null
334283	c	oo++oooo+	null	null
335182	g	o--------	null	null
336701	i	ooo--oo--	null	null
340385	g	o--------	null	null
341055	b	oo+oooooo	null	null
343680	a	oo+++++++	null	null
345973	g	o--------	null	null
348900	b	oooooo+oo	null	null
349371	b	oooooo+oo	null	null
351916	a	oo+++++++	null	null
355921	b	ooo+ooooo	null	null
358470	b	ooo+ooooo	null	null
359013	f	oo++----+	FLJ36980	null
366849	b	ooooo+ooo	LOC149913	null

367681	b	oo+oooooo	null	null
371288	b	ooo+ooooo	null	null
373518	b	ooo+ooooo	LOC253044	null
384686	b	ooo+ooooo	null	null
389373	a	oo+++++++	null	null
397208	b	ooooo+ooo	LOC441189	null
397748	h	oooooo-oo	MRGPRE	MAS-related GPR, member E
400486	f	oo++----+	null	null
405604	a	oo+++++++	FLJ31104	null
407295	b	ooo+ooooo	null	null
408180	g	oooooooo-	null	null
408619	g	o--------	RP11-133N1.2	null
408810	g	oooooooo-	null	null
411528	g	oooooo---	HMCN2	hemicentin 2
416527	b	ooooo+ooo	null	null
417159	l	oo--+----	null	null
423179	c	oo++++o++	LOC286442	null
424254	a	oooooooo+	null	null
424910	b	oooo++ooo	null	null
425760	a	oo+++++++	null	null
426464	h	oooooo-oo	LCE5A	late cornified envelope 5A
429918	a	oo+++++++	null	null
433615	b	oooo+oooo	LOC442117	null
441132	b	ooo+ooooo	null	null
441605	b	ooo+ooooo	LCE3B	late cornified envelope 3B
441609	b	ooo+ooooo	null	null
441929	b	ooo+ooooo	KIAA1856	null
445006	h	o-----ooo	null	null
449613	b	o+ooooooo	CSS3	null
453283	h	o-ooooooo	OPA3	optic atrophy 3 (autosomal recessive, with chorea and spastic paraplegia)
454339	b	ooo+ooooo	null	null
454495	g	o--------	PLEKHH2	pleckstrin homology domain containing, family H (with MyTH4 domain) member 2
458204	i	oo----o--	KIR3DL2	killer cell immunoglobulin-like receptor, three domains, long cytoplasmic tail, 2
460136	b	oooo+oooo	null	null
461154	b	oooo+oooo	ADAM5	ADAM metallopeptidase domain 5
464135	a	oo+++++++	null	null
464153	c	oo++oooo+	null	null
465359	b	ooooo+ooo	null	null
466024	a	oo+++++++	null	null
467477	g	oooooooo-	GIYD2|GIYD1	GIY-YIG domain containing 2|GIY-YIG domain containing 1
467496	b	oooo+oooo	LOC254027|LOC402505	null
468021	b	ooooo+ooo	null	null
471465	c	oo++oooo+	null	null
472751	b	ooo+++++o	null	null
473100	g	oo-------	null	null
477234	b	ooo+ooooo	null	null
478137	h	oooo-oooo	null	null
481600	b	oooo+oooo	null	null
481671	b	ooooooo+o	null	null
482264	a	oo+++++++	LOC283404	null
482282	g	o--------	LOC400145	null
482460	b	oooooo+oo	null	null
483224	b	oooooo+oo	SRL	sarcalumenin
486948	a	ooo++++++	null	null
487291	h	oooo-oooo	null	null
487491	b	ooooo+ooo	null	null
488616	b	ooo+ooooo	ATAD3C	ATPase family, AAA domain containing 3C
490209	a	oo+++++++	C15orf27	chromosome 15 open reading frame 27
497764	c	ooo+oo+++	LOC286434	null
505945	g	o--------	IGLC2	immunoglobulin lambda constant 2 (Kern-Oz- marker)
507727	b	ooooo+ooo	KRTAP20-2	keratin associated protein 20-2
510396	a	oo+++++++	null	null
511888	j	o-+++++++	null	null
515447	b	oooo+oooo	LOC284191	null
518960	a	oo+++++++	LOC440252|FLJ35785	null
519015	b	ooo++oooo	null	null
519577	b	oooo+oooo	null	null
520680	a	oo+++++++	null	null
522449	a	oo+++++++	C9orf10OS	chromosome 9 open reading frame 10 opposite strand

523577	h	oooo----o	null	null
523707	b	oooo+oooo	C18orf58	chromosome 18 open reading frame 58
526989	a	oooooooo+	null	null
530862	h	oooo----o	null	null
531143	g	o--------	null	null
536185	b	ooo+ooooo	RPS6KB1	ribosomal protein S6 kinase, 70kDa, polypeptide 1
536546	b	ooooooo+o	null	null
539197	a	oo+++++++	C3orf34	chromosome 3 open reading frame 34
540912	c	ooo+ooo++	STK6P	serine/threonine kinase 6 pseudogene
541411	c	oooo++o++	null	null
541734	a	o++++++++	LOC283711	null
541778	b	ooooooo+o	null	null
543606	b	ooo+ooooo	LOC132241	null
543648	a	oo+++++++	ZNF600	zinc finger protein 600
543659	b	ooooo+ooo	null	null
543821	a	oo+++++++	LOC286208	null
544258	b	ooo+++ooo	null	null
544290	a	ooo++++++	null	null
544895	b	oo++ooooo	null	null
545447	b	ooo+ooooo	null	null
545687	a	oo+++++++	null	null
546721	b	oooooo+oo	TICAM1	toll-like receptor adaptor molecule 1
547137	b	oooooo+oo	null	null
547559	b	ooo+ooooo	PRKCBP1	protein kinase C binding protein 1
547893	b	ooo+ooooo	LOC339237	null
547954	g	oooooooo-	SNRPA1	small nuclear ribonucleoprotein polypeptide A'
548321	b	ooo++oooo	null	null
548935	b	oo++ooooo	null	null
549085	h	oooooo-oo	LOC441025	null
557998	b	ooooooo+o	null	null
561969	b	ooooo+ooo	null	null
563594	a	oo+++++++	ZNF69	zinc finger protein 69 (Cos5)
567028	h	oooo----o	null	null
570676	b	ooo++++oo	null	null
579362	a	oo+++++++	null	null
579970	b	ooo+ooooo	STARD13	START domain containing 13
580152	b	oooo+oooo	null	null
587239	b	ooooooo+o	null	null
591492	b	ooo+ooooo	null	null
592040	a	o++++++++	null	null
592455	c	ooo+o++++	null	null
592747	g	o--------	null	null
593651	h	oooo----o	null	null
593700	b	ooooo+ooo	null	null
596935	a	ooo++++++	null	null
599896	b	ooo++oooo	MUC5AC	mucin 5, subtypes A and C, tracheobronchial/gastric
600514	b	o+ooooooo	null	null
609194	b	oooooo+oo	null	null
611537	b	oooooo+oo	LOC284274	null
611817	b	oooo+oooo	null	null
612290	h	oooo----o	null	null
613253	b	ooo+ooooo	null	null
613344	a	oo+++++++	null	null
616362	f	oo++----+	null	null
618097	b	ooo+ooooo	null	null
618708	h	o-ooooooo	null	null
625107	h	o-ooooooo	LOC285224	null
626382	g	o--------	LOC400792	null
626833	b	oooo+oooo	null	null
638716	b	ooo++oooo	FLJ42957	null
640464	g	o--------	OR52E8	olfactory receptor, family 52, subfamily E, member 8
642473	a	oooooooo+	null	null
644075	b	ooo+ooooo	null	null
646838	b	ooo+ooooo	null	null
650393	g	o--------	null	null
655954	g	o--------	null	null
658650	b	ooo+ooooo	ZBTB41	zinc finger and BTB domain containing 41
660069	d	ooo+oooo-	null	null
660714	b	ooooooo+o	null	null
661814	h	oooo----o	LOC144776	null
662018	b	ooo+ooooo	null	null

669652	b	ooo++++oo	null	null
670496	a	ooooo++++	null	null
670716	a	o++++++++	LOC284001	null
670775	a	o++++++++	NOMO1	NODAL modulator 1
671174	a	ooo++++++	null	null
675142	a	oooooo+++	null	null
676003	b	ooooooo+o	null	null
679798	h	ooooooo-o	null	null
680492	f	oo++----+	FLJ37201	null
681826	f	oo++----+	LOC442532	null
684151	a	oo+++++++	LOC442319|LOC442695	null
687856	a	oo+++++++	null	null
688396	b	ooooooo+o	null	null
688524	b	ooo+ooooo	C4orf13	chromosome 4 open reading frame 13
689728	h	ooo-----o	LOC401445	null
690010	a	ooooooo++	null	null
690745	b	ooooooo+o	null	null
690826	g	o--------	null	null
690975	b	ooo+ooooo	null	null
691129	b	ooo+ooooo	null	null
692566	b	oooooo+oo	C1orf157	chromosome 1 open reading frame 157
692868	b	ooooooo+o	LOC283177	null
692991	g	oo-------	null	null
693387	b	ooo+ooooo	LOC285878	null
693712	b	o+ooooooo	PDLIM2	PDZ and LIM domain 2 (mystique)
693991	a	oo+++++++	null	null
694208	b	ooooo+ooo	LOC554206	null
694438	b	oooo+oooo	null	null
694601	b	oo++ooooo	null	null
694903	a	oooooo+++	null	null
695143	a	oo+++++++	null	null
695191	b	ooo+ooooo	null	null
695272	b	oo++ooooo	null	null
695704	a	ooooo++++	null	null
696028	b	oooooo+oo	null	null
696238	b	oooo+oooo	null	null
696887	a	ooooo++++	LOC388638	null
697450	a	o++++++++	null	null
697587	h	o-ooooooo	null	null
697779	a	oo+++++++	LOC149157	null
698080	b	oooooo+oo	null	null
698132	g	oooooooo-	null	null
698285	b	ooooooo+o	null	null
698451	a	ooo++++++	ZNF623	zinc finger protein 623
698461	b	oo++ooooo	null	null
698503	a	oo+++++++	null	null
698834	a	oo+++++++	null	null
698913	a	ooo++++++	LOC439911	null
699375	b	oooo+oooo	null	null
699542	a	oo+++++++	null	null
699695	j	o-oooooo+	FLJ16287	null
700248	b	ooooo+ooo	null	null
700357	b	oooo+oooo	null	null
700578	h	ooooooo-o	null	null
700714	a	oooooooo+	TAS2R44	taste receptor, type 2, member 44
700972	f	oo++----+	TCERG1L	transcription elongation regulator 1-like
701114	b	oo+oooooo	CALD1	caldesmon 1
701184	b	oooo+oooo	null	null
701322	b	o++oooooo	null	null
701655	f	oo++----+	null	null
701694	b	oooo+oooo	null	null
701848	a	o++++++++	NOMO3|NOMO2	NODAL modulator 3|NODAL modulator 2
702299	b	ooo+++ooo	null	null
702434	b	oooooo+oo	OR2W3	olfactory receptor, family 2, subfamily W, member 3
702618	b	ooooo+ooo	FLJ44715	null
702861	b	ooooooo+o	null	null
703181	b	ooo+ooooo	IFNE1	null
703355	a	ooo++++++	null	null
703394	b	ooo+ooooo	GPRC5C	G protein-coupled receptor, family C, group 5, member C
703467	c	ooooo+oo+	IQSEC3	IQ motif and Sec7 domain 3

703675	a	o++++++++	AMY1A|AMY1B|AMY1C|AMY2A	amylase, alpha 1A; salivary|amylase, alpha 1B; salivary|amylase, alpha 1C; salivary|amylase, alpha 2A; pancreatic
703868	b	o+ooooooo	OR4C6	olfactory receptor, family 4, subfamily C, member 6
703877	g	o--------	FLJ40411	null
704077	b	oooo+oooo	null	null
704350	c	ooo++o+++	C1orf61	chromosome 1 open reading frame 61
705174	a	oo+++++++	null	null
705420	g	o--------	null	null
705453	h	o-------o	null	null
705650	b	oooo+oooo	null	null
706139	b	ooo+ooooo	LOC441240	null
706317	g	o--------	null	null
706352	f	oo++----+	null	null
706453	c	oo+++o+++	LOC285331	null
706648	b	ooooo+ooo	RBM11	RNA binding motif protein 11
706801	b	ooooooo+o	AGRN	agrin
707077	b	ooo+ooooo	ZNF700	zinc finger protein 700
707086	b	ooo+ooooo	ZNF69	zinc finger protein 69 (Cos5)
707175	g	o--------	MEF2B	MADS box transcription enhancer factor 2, polypeptide B (myocyte enhancer factor 2B)
707408	b	oooooo+oo	TAS2R48	taste receptor, type 2, member 48
707537	a	ooo++++++	LOC402031	null
707864	a	oo+++++++	LOC339047|NPIP	nuclear pore complex interacting protein
707950	g	o--------	null	null
707993	b	ooo++oooo	null	null
708017	b	ooo+ooooo	null	null
708046	i	o----o---	null	null
708164	h	o-ooooooo	null	null
708365	b	oooooo+oo	TAS2R45	taste receptor, type 2, member 45
708492	a	oo+++++++	null	null
708603	h	o-ooooooo	MGA	MAX gene associated
708627	b	ooooo+ooo	null	null
708742	b	ooo+ooooo	null	null
708744	b	ooooooo+o	null	null
709073	h	o-ooooooo	null	null
709195	b	oooooo+oo	null	null
709215	b	ooo+ooooo	null	null
709529	a	ooooooo++	null	null
709647	b	ooo+ooooo	KRTAP12-4	keratin associated protein 12-4
709869	h	oooo----o	null	null
709939	b	oooooo+oo	null	null
710378	h	oooo----o	LOC133308	null
710608	c	oooo+ooo+	MGC11257	null
710730	a	oo+++++++	LOC149157	null
710818	g	o--------	REXO1L1|REXO1L7P|REXO1L2P	REX1, RNA exonuclease 1 homolog (S. cerevisiae)-like 1|REX1, RNA exonuclease 1 homolog (S. cerevisiae)-like 7 (pseudogene)|REX1, RNA exonuclease 1 homolog (S. cerevisiae)-like 2 (pseudogene)
710900	b	oooooo+oo	PRO2852	null
710945	g	ooooo----	WDR6	WD repeat domain 6
711142	b	oooooo+oo	LOC201617	null
711181	b	oooo+oooo	null	null
711358	b	oooo+oooo	null	null
711583	a	oo+++++++	FTS	fused toes homolog (mouse)
711951	b	ooo+++++o	null	null
712531	a	oo+++++++	null	null
712587	b	oo+oooooo	DEFA3|DEFA1	defensin, alpha 3, neutrophil-specific|defensin, alpha 1
712595	b	ooooooo+o	LOC440585	null
712642	g	oo-------	null	null
712930	h	o-ooooooo	LOC285711	null
712974	b	oooo+oooo	null	null
713144	a	oooooo+++	null	null
713561	c	oo+ooo+++	RAB42	RAB42, member RAS homolog family
713562	b	oooo+oooo	null	null
713792	g	oooooooo-	LOC128499	null
713820	h	o-ooooooo	LOC440518	null
713929	b	oooo++ooo	null	null
714079	b	ooooooo+o	LOC440095	null
714113	a	o++++++++	C1orf189	chromosome 1 open reading frame 189
714402	b	oooo+oooo	null	null
714879	a	o++++++++	null	null
715039	f	oo++----+	DIO3OS	deiodinase, iodothyronine, type III opposite strand

715240	b	oooo+oooo	null	null
715525	c	oooo+ooo+	OR10J5	olfactory receptor, family 10, subfamily J, member 5
716041	h	oooooo-oo	KIAA0514	KIAA0514
716048	b	ooooo+ooo	LATS2	LATS, large tumor suppressor, homolog 2 (Drosophila)
716167	b	ooooo+ooo	null	null
716176	b	oo++ooooo	null	null
716495	b	ooo+ooooo	null	null
716578	i	o------o-	null	null
716616	b	ooooooo+o	null	null
716632	b	ooo+ooooo	OR4M1	olfactory receptor, family 4, subfamily M, member 1
716738	g	o--------	null	null
