# Supplementary figures and images for: BIM and NOXA are mitochondrial effectors of TAF6δ-driven apoptosis
Source: Cell Death Dis. 2018 Jan 22;9(2):70. doi: 10.1038/s41419-017-0115-3 (PMC5833734; doi:10.1038/s41419-017-0115-3)

a

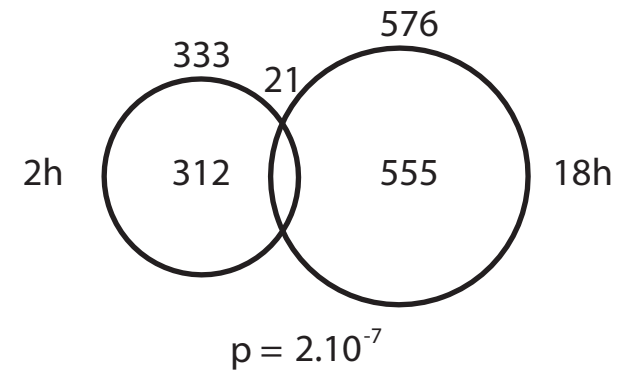

b

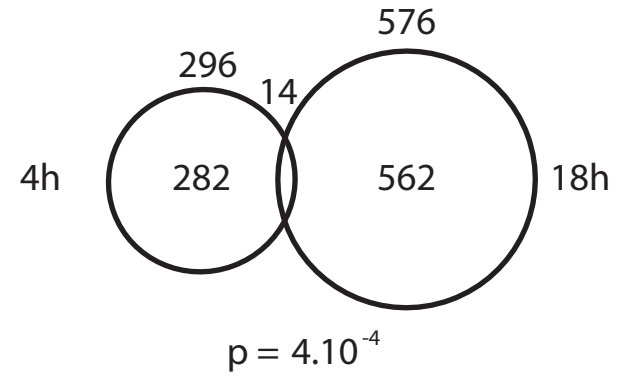

c

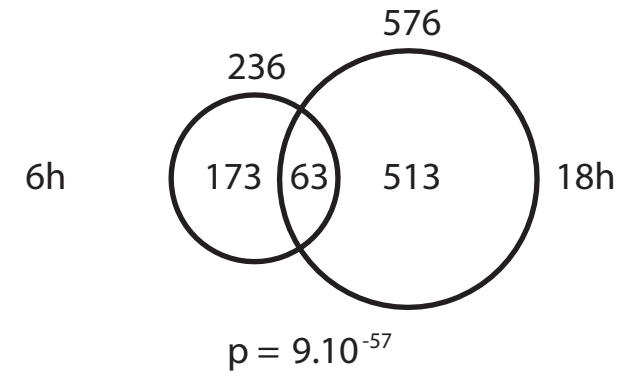

d

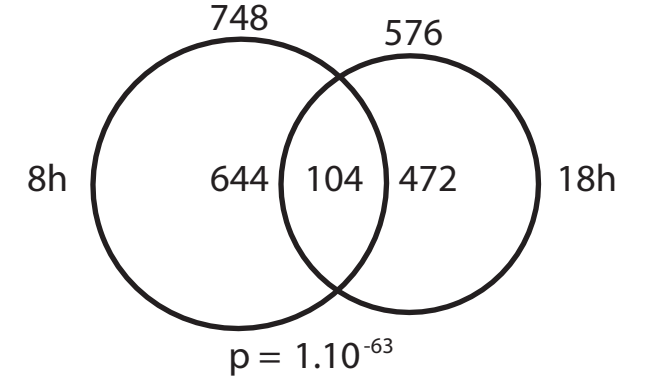

e

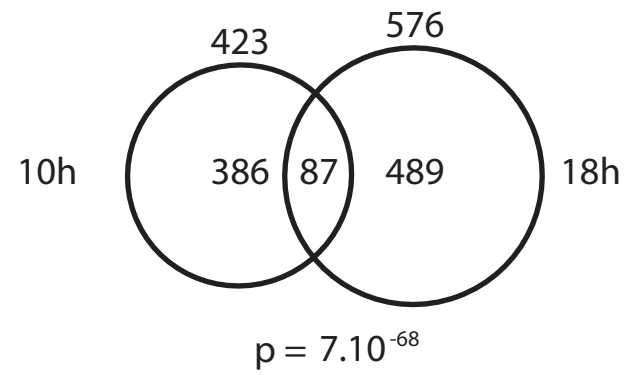

f

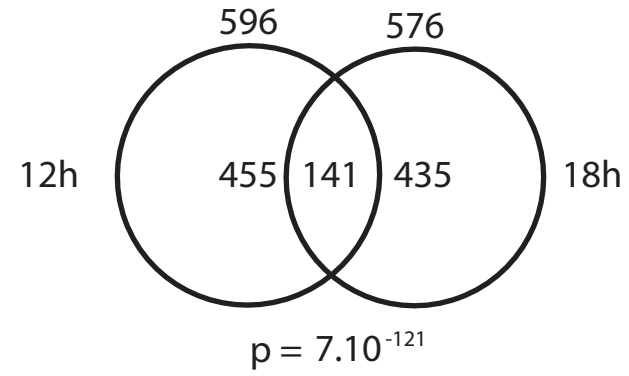

g

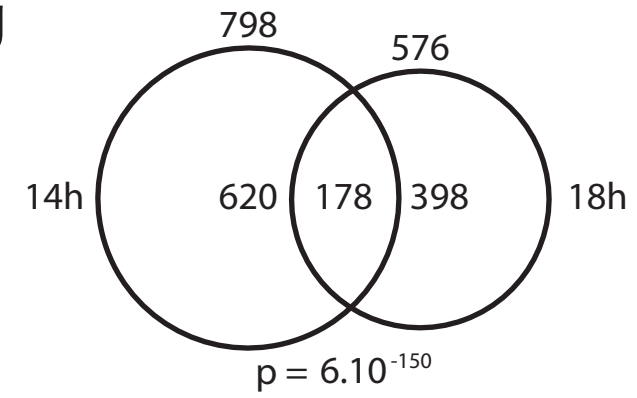

h

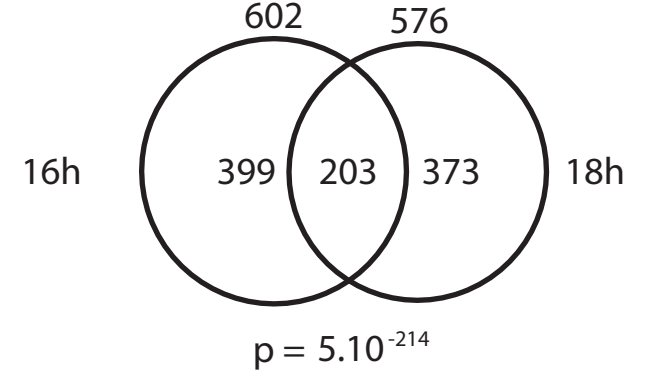

Supplement: Supplementary file 3 — Supplementary Figure 1 [file 41419_2017_115_MOESM3_ESM.pdf]

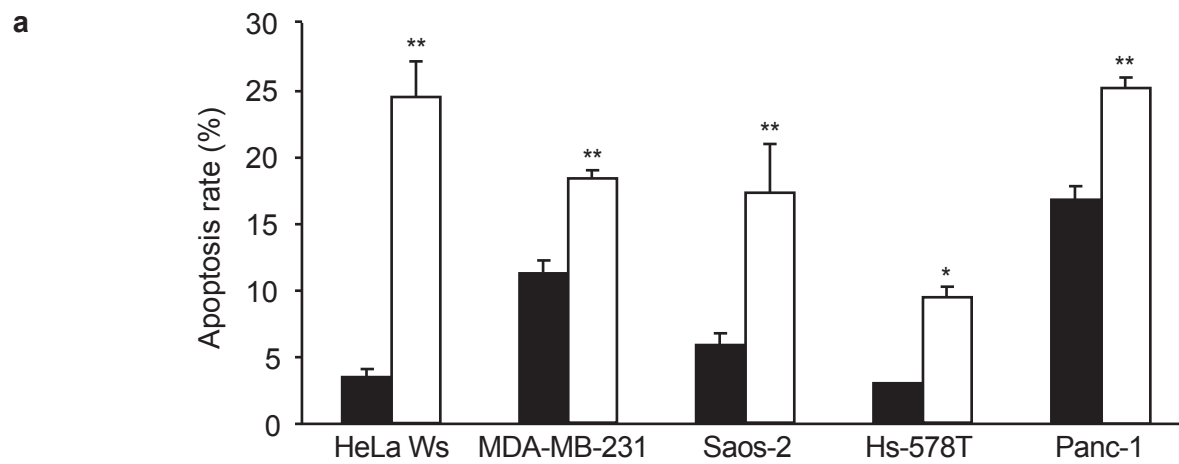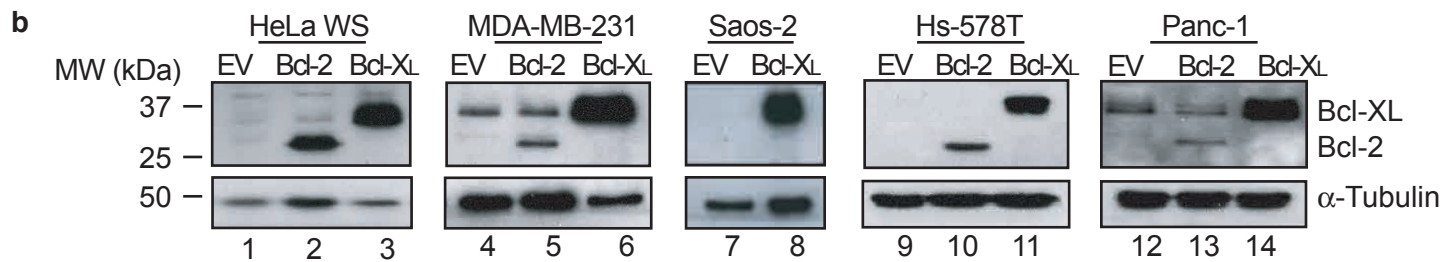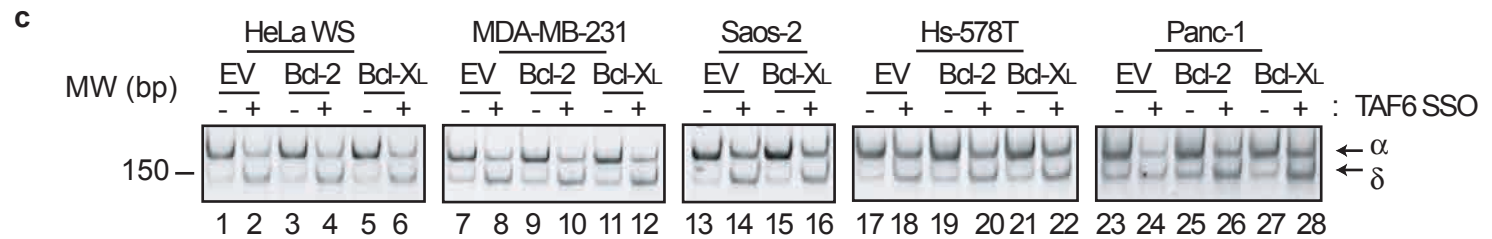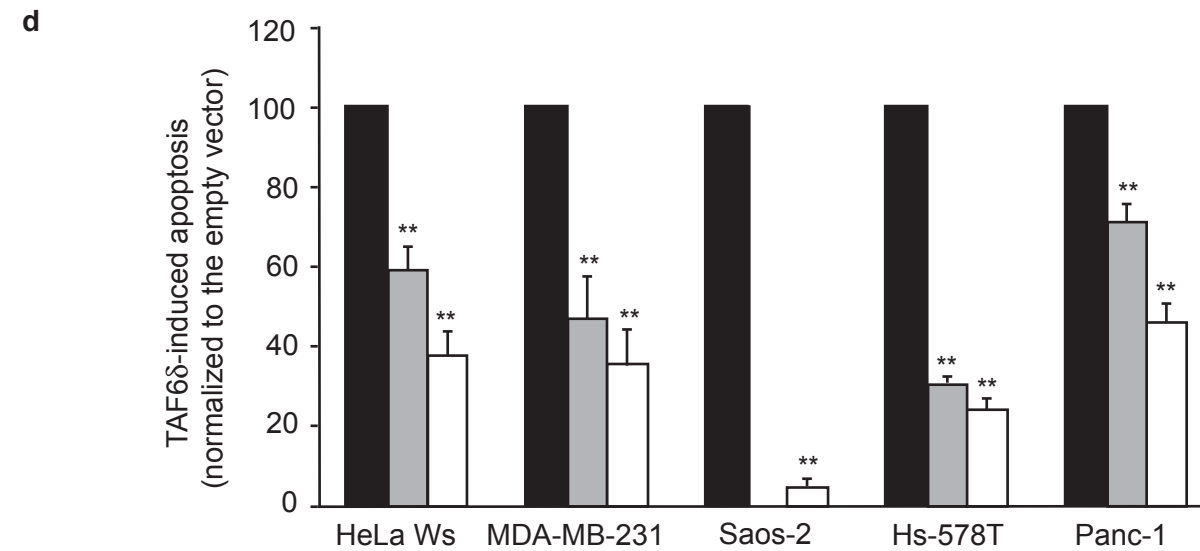

Supplement: Supplementary file 6 — Supplementary Figure 2 [file 41419_2017_115_MOESM6_ESM.pdf]

**a**

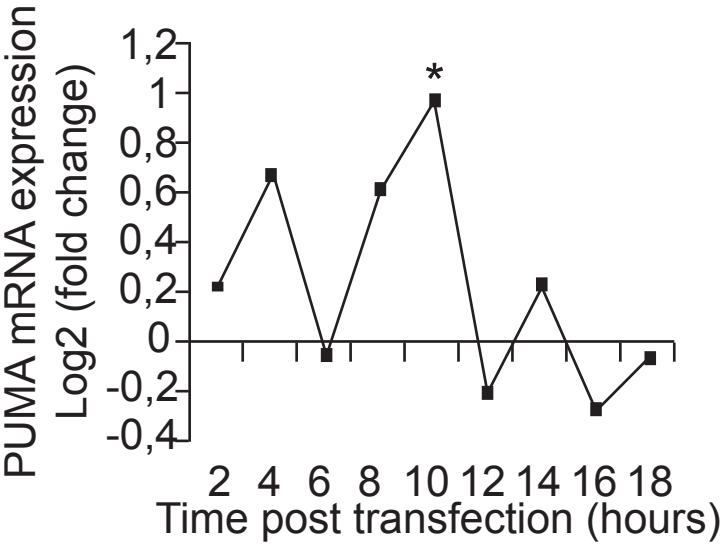

**b**

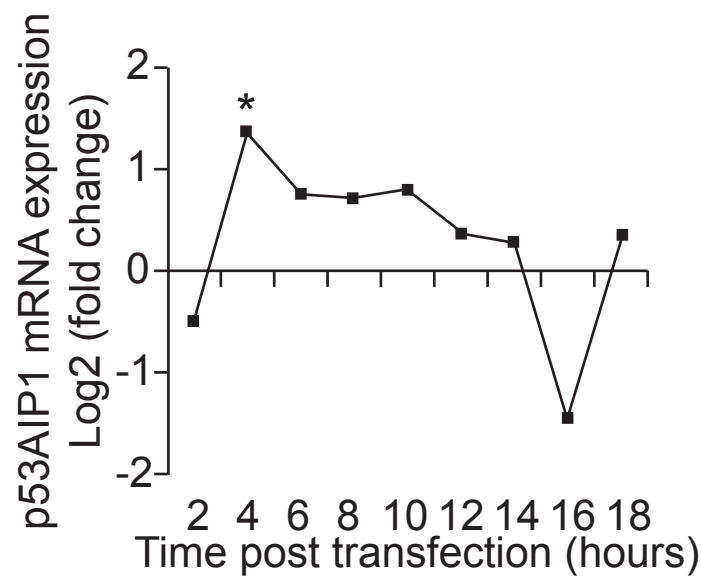

Supplement: Supplementary file 8 — Supplementary Figure 3 [file 41419_2017_115_MOESM8_ESM.pdf]
