# Supplementary material for: BIM and NOXA are mitochondrial effectors of TAF6δ-driven apoptosis
Source: Cell Death Dis. 2018 Jan 22;9(2):70. doi: 10.1038/s41419-017-0115-3 (PMC5833734; doi:10.1038/s41419-017-0115-3)
Supplement: Supplementary file 4 — Supplementary Table 2 [file 41419_2017_115_MOESM4_ESM.pdf]

Supplementary Table 2.

	Ontologies	Ref	Obs	Exp	+/-	pValue
**2h**	Cytoskeletal regulation by Rho GTPase	175	6	1.7	+	0.006
	Thiamine metabolism	2	1	0.0	+	0.019
	Heterotrimeric G-protein signaling pathway-Gq alpha and Go alpha mediated pathway	192	5	1.9	+	0.028
**4h**	Pyrimidine Metabolism	18	2	0.2	+	0.010
**6h**	p53 pathway	169	5	1.2	+	0.005
	p53 pathway feedback loops 2	87	3	0.6	+	0.019
**8h**	Axon guidance mediated by Slit/Robo	51	4	1.1	+	0.020
	Phenylethylamine degradation	12	2	0.3	+	0.026
	Thiamine metabolism	2	1	0.0	+	0.041
	Lysine biosynthesis	2	1	0.0	+	0.041
	Inflammation mediated by chemokine and cytokine signaling pathway	402	13	8.6	+	0.042
	Wnt signaling pathway	454	14	9.8	+	0.047
**10h**	FGF signaling pathway	196	9	2.4	+	0.001
	Apoptosis signaling pathway	187	7	2.3	+	0.006
	T cell activation	207	7	2.5	+	0.010
	B cell activation	113	5	1.4	+	0.010
	Pyrimidine Metabolism	18	2	0.2	+	0.019
	Circadian clock system	18	2	0.2	+	0.019
**12h**	Circadian clock system	18	3	0.3	+	0.004
	Oxidative stress response	89	6	1.5	+	0.004
	Glycolysis	40	3	0.7	+	0.028
	FGF signaling pathway	196	7	3.4	+	0.034
**14h**	Oxidative stress response	89	6	2.0	+	0.013
	Notch signaling pathway	66	5	1.5	+	0.015
	TGF-beta signaling pathway	188	9	4.3	+	0.019
	DNA replication	26	3	0.6	+	0.020
	FAS signaling pathway	48	4	1.1	+	0.021
	Angiogenesis	296	11	6.8	+	0.040
	Ubiquitin proteasome pathway	136	0	3.1	-	0.043
	Succinate to proprionate conversion	2	1	0.0	+	0.044
**16h**	Oxidative stress response	89	6	1.6	+	0.004
	Apoptosis signaling pathway	187	9	3.3	+	0.004
	Axon guidance mediated by Slit/Robo	51	4	0.9	+	0.011
	Asparagine and aspartate biosynthesis	10	2	0.2	+	0.013
	TGF-beta signaling pathway	188	7	3.3	+	0.031
	B cell activation	113	5	2.0	+	0.035
	Interferon-gamma signaling pathway	47	3	0.8	+	0.041
	Inflammation mediated by chemokine and cytokine signaling pathway	402	11	7.0	+	0.046
	Toll receptor signaling pathway	87	4	1.5	+	0.049
**18h**	Oxidative stress response	89	9	1.5	+	0.000
	p38 MAPK pathway	86	6	1.4	+	0.003
	O-antigen biosynthesis	6	2	0.1	+	0.005
	p53 pathway	169	8	2.8	+	0.006
	TGF-beta signaling pathway	188	8	3.1	+	0.010
	N-acetylglucosamine metabolism	10	2	0.2	+	0.012
	Asparagine and aspartate biosynthesis	10	2	0.2	+	0.012
	EGF receptor signaling pathway	203	8	3.4	+	0.014
	Huntington disease	251	9	4.2	+	0.016
	p53 pathway feedback loops 2	87	4	1.5	+	0.043
	Axon guidance mediated by Slit/Robo	51	3	0.9	+	0.044
