# Supplementary material for: BIM and NOXA are mitochondrial effectors of TAF6δ-driven apoptosis
Source: Cell Death Dis. 2018 Jan 22;9(2):70. doi: 10.1038/s41419-017-0115-3 (PMC5833734; doi:10.1038/s41419-017-0115-3)
Supplement: Supplementary file 5 — Supplementary Table 3 [file 41419_2017_115_MOESM5_ESM.pdf]

**Supplementary Table 3.**

| probe | $R^2$ | P | Entrez Gene ID | Celera Gene ID | Gene_Symbol | Gene_Name |
|---|---|---|---|---|---|---|
| 106600 | 0.993 | 1.07E-07 | 4616 | hCG22705.4 | GADD45B | growth arrest and DNA-damage-inducible, beta |
| 179827 | 0.988 | 5.88E-07 | 7538 | hCG43352.3 | ZFP36 | zinc finger protein 36, C3H type, homolog (mouse) |
| 121840 | 0.988 | 6.39E-07 | 1906 | hCG37405.3 | EDN1 | endothelin 1 |
| 211412 | 0.984 | 1.56E-06 | 1316 | hCG24940.3 | KLF6 | Kruppel-like factor 6 |
| 162382 | 0.983 | 2.02E-06 | 8335 | hCG1787383.1 | HIST1H2AB | histone 1, H2ab |
| 177445 | 0.983 | 2.15E-06 | 51278 | hCG1766702.2 | IER5 | immediate early response 5 |
| 113849 | 0.982 | 2.61E-06 | null | hCG1818557.1 | null | null |
| 111844 | 0.981 | 3.06E-06 | 8330 | hCG1643754.3 | HIST1H2AK | histone 1, H2ak |
| 107292 | 0.980 | 3.95E-06 | 83667 | hCG19575.3 | SESN2 | sestrin 2 |
| 105377 | 0.977 | 5.93E-06 | 1491 | hCG21682.4 | CTH | cystathionase (cystathionine gamma-lyase) |
| 174556 | 0.976 | 6.74E-06 | 10365 | hCG36957.3 | KLF2 | Kruppel-like factor 2 (lung) |
| 185687 | 0.975 | 7.50E-06 | 467 | hCG37734.3 | ATF3 | activating transcription factor 3 |
| 225333 | 0.975 | 8.04E-06 | 205860 | hCG2025667.1 | FLJ25801 | null |
| 144113 | 0.974 | 8.92E-06 | 2354 | hCG20725.3 | FOSB | FBJ murine osteosarcoma viral oncogene homolog B |
| 202436 | 0.973 | 1.08E-05 | 83729 | hCG1818161.1 | INHBE | inhibin, beta E |
| 222445 | 0.972 | 1.18E-05 | 8365 | hCG1640981.4 | HIST1H4H | histone 1, H4h |
| 202780 | 0.971 | 1.39E-05 | 133 | hCG23989.3 | ADM | adrenomedullin |
| 164901 | 0.966 | 2.30E-05 | 128439 | hCG2019855.1 | LOC128439 | null |
| 114661 | 0.960 | 3.92E-05 | 10949 | hCG1639951.4 | HNRPA0 | heterogeneous nuclear ribonucleoprotein A0 |
| 118988 | 0.960 | 4.15E-05 | null | hCG2042712 | null | null |
| 176983 | 0.959 | 4.42E-05 | 3280 | hCG16317.2 | HES1 | hairy and enhancer of split 1, (Drosophila) |
| 223324 | 0.958 | 4.83E-05 | 541472 | null | LOC541472 | null |
| 163509 | 0.957 | 5.05E-05 | 1649 | hCG39661.3 | DDIT3 | DNA-damage-inducible transcript 3 |
| 173408 | 0.957 | 5.32E-05 | 2920 | hCG16361.2 | CXCL2 | chemokine (C-X-C motif) ligand 2 |
| 173294 | 0.955 | 5.87E-05 | 353322 | hCG16632.2 | ANKRD37 | ankyrin repeat domain 37 |
| 129829 | 0.954 | 6.41E-05 | 390 | hCG15792.3 | RND3 | Rho family GTPase 3 |
| 111012 | 0.954 | 6.46E-05 | 23040 | hCG1990275 | MYT1L | myelin transcription factor 1-like |
| 182404 | 0.954 | 6.74E-05 | 9518 | hCG37044.2 | GDF15 | growth differentiation factor 15 |
| 190669 | 0.952 | 7.52E-05 | 112970 | hCG2039599 | KTI12 | KTI12 homolog, chromatin associated (S. cerevisiae) |
| 177389 | 0.952 | 7.54E-05 | 2921 | hCG16366.3 | CXCL3 | chemokine (C-X-C motif) ligand 3 |
| 157342 | 0.951 | 7.90E-05 | 57035 | hCG1981326 | C1orf63 | chromosome 1 open reading frame 63 |
| 147353 | 0.951 | 8.06E-05 | 1958 | hCG18777.1 | EGR1 | early growth response 1 |
| 121612 | 0.951 | 8.43E-05 | 1847 | hCG1811162.1 | DUSP5 | dual specificity phosphatase 5 |
| 200967 | 0.950 | 8.55E-05 | null | hCG1820954.2 | null | null |
| 163612 | 0.950 | 8.59E-05 | 9592 | hCG26671.3 | IER2 | immediate early response 2 |
| 233928 | 0.950 | 8.67E-05 | 153546 | hCG2003913 | LOC153546 | null |
| 226336 | 0.950 | 8.78E-05 | 9935 | hCG37146.3 | MAFB | v-maf musculoaponeurotic fibrosarcoma oncogene homolog B (avian) |
| 180957 | 0.950 | 8.98E-05 | 4758 | hCG43692.3 | NEU1 | sialidase 1 (lysosomal sialidase) |
| 127242 | 0.948 | 9.84E-05 | 5366 | hCG1776475.1 | PMAIP1 | phorbol-12-myristate-13-acetate-induced protein 1 |
| 694903 | 0.948 | 1.00E-04 | null | hCG2038998 | null | null |
| 204743 | 0.948 | 1.02E-04 | 3397 | hCG37143.3 | ID1 | inhibitor of DNA binding 1, dominant negative helix-loop-helix protein |
| 163241 | 0.947 | 1.04E-04 | 3569 | hCG38231.4 | IL6 | interleukin 6 (interferon, beta 2) |
| 177711 | 0.945 | 1.22E-04 | null | hCG1820665.1 | null | null |
| 113820 | 0.944 | 1.29E-04 | 29946 | hCG201413.3 | SERTAD3 | SERTA domain containing 3 |
| 197326 | 0.944 | 1.33E-04 | 379 | hCG28385.1 | ARL4D | ADP-ribosylation factor-like 4D |
| 154907 | 0.943 | 1.36E-04 | 1490 | hCG22108.3 | CTGF | connective tissue growth factor |
| 197613 | 0.943 | 1.39E-04 | 8370\|554313 | null | HIST2H4\|H4/o | histone 2, H4 |
| 192258 | 0.942 | 1.42E-04 | 1647 | hCG21703.4 | GADD45A | growth arrest and DNA-damage-inducible, alpha |
| 196687 | 0.940 | 1.62E-04 | 135112 | hCG2030497 | NCOA7 | nuclear receptor coactivator 7 |
| 121695 | 0.940 | 1.62E-04 | 1027 | hCG27692.2 | CDKN1B | cyclin-dependent kinase inhibitor 1B (p27, Kip1) |
| 225617 | 0.940 | 1.67E-04 | 8357 | hCG1640464.3 | HIST1H3H | histone 1, H3h |
| 102081 | 0.939 | 1.70E-04 | 8013 | hCG28754.3 | NR4A3 | nuclear receptor subfamily 4, group A, member 3 |
| 232527 | 0.938 | 1.80E-04 | 9972 | hCG1979965 | NUP153 | nucleoporin 153kDa |
| 166747 | 0.938 | 1.81E-04 | 51526 | hCG38102.3 | C20orf111 | chromosome 20 open reading frame 111 |
| 122554 | 0.938 | 1.88E-04 | 8331 | hCG1643194.3 | HIST1H2AJ | histone 1, H2aj |
| 228107 | 0.937 | 1.91E-04 | 257358 | hCG1978968 | LOC257358 | null |
| 103618 | 0.937 | 1.93E-04 | 11080 | hCG22857.4 | DNAJB4 | DnaJ (Hsp40) homolog, subfamily B, member 4 |
| 184838 | 0.937 | 1.95E-04 | 25976 | hCG27716.2 | TIPARP | TCDD-inducible poly(ADP-ribose) polymerase |
| 104428 | 0.936 | 2.09E-04 | 9205 | hCG2019830 | ZMYM5 | zinc finger, MYM-type 5 |
| 183615 | 0.935 | 2.13E-04 | 29950 | hCG1777718.2 | SERTAD1 | SERTA domain containing 1 |
| 113737 | 0.935 | 2.19E-04 | 57761 | hCG2019820.2 | TRIB3 | tribbles homolog 3 (Drosophila) |
| 133326 | 0.934 | 2.23E-04 | 57602 | hCG95566.3 | USP36 | ubiquitin specific peptidase 36 |
| 159394 | 0.934 | 2.33E-04 | 6376 | hCG15105.2 | CX3CL1 | chemokine (C-X3-C motif) ligand 1 |
| 108623 | 0.933 | 2.39E-04 | 56892 | hCG1640844.3 | C8orf4 | chromosome 8 open reading frame 4 |
| 204620 | 0.933 | 2.43E-04 | 5876 | hCG22856.3 | RABGGTB | Rab geranylgeranyltransferase, beta subunit |
| 197227 | 0.933 | 2.43E-04 | 3885 | hCG1641096.3 | KRTHA4 | keratin, hair, acidic, 4 |
| 175188 | 0.931 | 2.64E-04 | 79074 | hCG1642079.2 | MGC5509 | null |
| 107157 | 0.931 | 2.67E-04 | 5876 | null | RABGGTB | Rab geranylgeranyltransferase, beta subunit |
| 123273 | 0.931 | 2.68E-04 | 3725 | hCG1780282.1 | JUN | v-jun sarcoma virus 17 oncogene homolog (avian) |
| 210401 | 0.930 | 2.72E-04 | 1022 | hCG1988840 | CDK7 | cyclin-dependent kinase 7 (MO15 homolog, Xenopus laevis, cdk-activating kinase) |
| 134083 | 0.930 | 2.75E-04 | 7357 | hCG27600.2 | UGCG | UDP-glucose ceramide glucosyltransferase |
| 144049 | 0.929 | 2.86E-04 | 694 | hCG21188.3 | BTG1 | B-cell translocation gene 1, anti-proliferative |
| 109708 | 0.929 | 2.93E-04 | 85028 | hCG1773592.1 | C1orf79 | chromosome 1 open reading frame 79 |
| 160257 | 0.928 | 3.02E-04 | 54541 | hCG2024489 | DDIT4 | DNA-damage-inducible transcript 4 |
| 133600 | 0.928 | 3.08E-04 | 55422 | hCG2009080 | ZNF331 | zinc finger protein 331 |
| 156527 | 0.928 | 3.14E-04 | 7494 | hCG40980.4 | XBP1 | X-box binding protein 1 |
| 174736 | 0.927 | 3.28E-04 | 7425 | hCG1640612.3 | VGF | VGF nerve growth factor inducible |
| 103451 | 0.926 | 3.39E-04 | 57132 | hCG1780859.1 | CHMP1B | chromatin modifying protein 1B |
| 166020 | 0.925 | 3.57E-04 | 3491 | hCG23652.3 | CYR61 | cysteine-rich, angiogenic inducer, 61 |
| 204006 | 0.925 | 3.60E-04 | 1030 | hCG28310.3 | CDKN2B | cyclin-dependent kinase inhibitor 2B (p15, inhibits CDK4) |
| 221617 | 0.923 | 3.79E-04 | 6428 | hCG14802.2 | SFRS3 | splicing factor, arginine/serine-rich 3 |
| 104873 | 0.923 | 3.79E-04 | 55629 | hCG1728885.2 | PNRC2 | proline-rich nuclear receptor coactivator 2 |
| 166946 | 0.923 | 3.80E-04 | 468 | hCG41721.3 | ATF4 | activating transcription factor 4 (tax-responsive enhancer element B67) |
| 132057 | 0.923 | 3.85E-04 | 8334 | hCG19120.4 | HIST1H2AC | histone 1, H2ac |
| 206606 | 0.923 | 3.85E-04 | 60370 | hCG1642760.4 | AVPI1 | arginine vasopressin-induced 1 |
| 100596 | 0.923 | 3.92E-04 | 1054 | hCG20141.2 | CEBPG | CCAAT/enhancer binding protein (C/EBP), gamma |
| 213319 | 0.922 | 4.12E-04 | null | hCG1820858.1 | null | null |
| 141605 | 0.921 | 4.27E-04 | 114915 | hCG1810794.1 | TIGA1 | null |

| | | | | | | |
|---|---|---|---|---|---|---|
| 123450 | 0.920 | 4.48E-04 | 4929 | hCG38357.2 | NR4A2 | nuclear receptor subfamily 4, group A, member 2 |
| 116772 | 0.919 | 4.54E-04 | 388796 | null | LOC388796 | null |
| 221618 | 0.919 | 4.60E-04 | 149647 | hCG2036883 | FAM71A | family with sequence similarity 71, member A |
| 194691 | 0.918 | 4.72E-04 | 93622 | hCG1981777 | LOC93622 | null |
| 175225 | 0.918 | 4.78E-04 | 4794 | hCG18999.4 | NFKBIE | nuclear factor of kappa light polypeptide gene enhancer in B-cells inhibitor, epsilon |
| 147930 | 0.918 | 4.83E-04 | 2872 | hCG22717.3 | MKNK2 | MAP kinase interacting serine/threonine kinase 2 |
| 119663 | 0.918 | 4.87E-04 | 64784 | null | CRTC3 | CREB regulated transcription coactivator 3 |
| 116793 | 0.917 | 4.91E-04 | 5997 | hCG41052.3 | RGS2 | regulator of G-protein signalling 2, 24kDa |
| 144834 | 0.917 | 4.99E-04 | 126961\|449003 | null | HIST2H3C\|H3/o | histone 2, H3c |
| 205128 | 0.917 | 5.01E-04 | 2353 | hCG22355.3 | FOS | v-fos FBJ murine osteosarcoma viral oncogene homolog |
| 144265 | 0.916 | 5.21E-04 | 8332 | hCG1743060.2 | HIST1H2AL | histone 1, H2al |
| 162911 | 0.916 | 5.28E-04 | 7057 | hCG1787130.4 | THBS1 | thrombospondin 1 |
| 179647 | 0.915 | 5.49E-04 | 146434 | hCG16640.4 | ZNF597 | zinc finger protein 597 |
| 129189 | 0.914 | 5.57E-04 | 23161 | hCG2009798.1 | SNX13 | sorting nexin 13 |
| 191821 | 0.913 | 5.94E-04 | 7852 | hCG25754.2 | CXCR4 | chemokine (C-X-C motif) receptor 4 |
| 168741 | 0.912 | 6.16E-04 | 7128 | hCG16787.4 | TNFAIP3 | tumor necrosis factor, alpha-induced protein 3 |
| 135806 | 0.912 | 6.19E-04 | 7737 | hCG22489.2 | RNF113A | ring finger protein 113A |
| 194026 | 0.912 | 6.22E-04 | 286343 | hCG27957.3 | C9orf150 | chromosome 9 open reading frame 150 |
| 148950 | 0.911 | 6.32E-04 | 91179 | null | SCARF2 | scavenger receptor class F, member 2 |
| 172931 | 0.911 | 6.42E-04 | 84324 | hCG2016179 | CIP29 | null |
| 149253 | 0.910 | 6.55E-04 | 9182 | hCG20392.2 | PAMCI | peptidylglycine alpha-amidating monooxygenase COOH-terminal interactor |
| 149647 | 0.910 | 6.59E-04 | 8337\|8338 | hCG2036722\|hCG2039933 | HIST2H2AA\|HIST2H2AC | histone 2, H2aa\|histone 2, H2ac |
| 151000 | 0.910 | 6.61E-04 | 1052 | hCG18323.3 | CEBPD | CCAAT/enhancer binding protein (C/EBP), delta |
| 149591 | 0.908 | 7.14E-04 | 388 | hCG31737.3 | RHOB | ras homolog gene family, member B |
| 175491 | 0.908 | 7.18E-04 | 6046 | hCG17503.3 | BRD2 | bromodomain containing 2 |
| 106376 | 0.907 | 7.43E-04 | 388677 | null | NOTCH2NL | Notch homolog 2 (Drosophila) N-terminal like |
| 170698 | 0.906 | 7.68E-04 | null | null | null | null |
| 233364 | 0.905 | 7.97E-04 | 84099 | hCG22212.3 | ID2B | inhibitor of DNA binding 2B, dominant negative helix-loop-helix protein |
| 148153 | 0.904 | 8.30E-04 | 1846 | hCG1639778.4 | DUSP4 | dual specificity phosphatase 4 |
| 192760 | 0.903 | 8.50E-04 | null | hCG2041159\|hCG2039306 | null | null |
| 117132 | 0.903 | 8.50E-04 | 8848 | hCG32172.3 | TSC22D1 | TSC22 domain family, member 1 |
| 176322 | 0.903 | 8.58E-04 | 388677 | null | NOTCH2NL | Notch homolog 2 (Drosophila) N-terminal like |
| 105979 | 0.903 | 8.59E-04 | 92595 | hCG19316.3 | MGC13138 | null |
| 205961 | 0.903 | 8.60E-04 | 1195 | hCG16556.2 | CLK1 | CDC-like kinase 1 |
| 142499 | 0.901 | 9.09E-04 | 84962 | hCG41752.3 | JUB | jub, ajuba homolog (Xenopus laevis) |
| 126351 | 0.901 | 9.26E-04 | 27289 | hCG1999395 | RND1 | Rho family GTPase 1 |
| 115353 | 0.901 | 9.26E-04 | 1848 | hCG26604.2 | DUSP6 | dual specificity phosphatase 6 |
| 187637 | 0.898 | 9.99E-04 | 84837 | hCG1806191.2 | C14orf128 | chromosome 14 open reading frame 128 |
| 148704 | 0.898 | 1.02E-03 | 3039\|3040 | hCG1745306.2 | HBA1\|HBA2 | hemoglobin, alpha 1\|hemoglobin, alpha 2 |
| 193615 | 0.897 | 1.03E-03 | 55603 | hCG401094.3 | FAM46A | family with sequence similarity 46, member A |
| 141393 | 0.897 | 1.04E-03 | 1390 | hCG23484.3 | CREM | cAMP responsive element modulator |
| 167664 | 0.897 | 1.04E-03 | 6617 | hCG22360.2 | SNAPC1 | small nuclear RNA activating complex, polypeptide 1, 43kDa |
| 135984 | 0.897 | 1.05E-03 | 387066 | hCG2031039 | C6orf160 | chromosome 6 open reading frame 160 |
| 191116 | 0.897 | 1.06E-03 | 1974 | hCG1784041.2 | EIF4A2 | eukaryotic translation initiation factor 4A, isoform 2 |
| 130047 | 0.895 | 1.10E-03 | 84124 | hCG1742898.1 | ZNF394 | zinc finger protein 394 |
| 231117 | 0.895 | 1.12E-03 | null | hCG2002074 | null | null |
| 200785 | 0.894 | 1.13E-03 | 401474 | hCG2038156.1\|hCG2041254 | SAMD12 | sterile alpha motif domain containing 12 |
| 713144 | 0.894 | 1.15E-03 | null | hCG2038372 | null | null |
| 112937 | 0.894 | 1.16E-03 | null | hCG39909.2 | null | null |
| 193172 | 0.892 | 1.22E-03 | 8355 | hCG1641781.2 | HIST1H3G | histone 1, H3g |
| 142603 | 0.892 | 1.23E-03 | 6591 | hCG32298.3 | SNAI2 | snail homolog 2 (Drosophila) |
| 213523 | 0.892 | 1.23E-03 | 79027 | hCG1742885.3 | ZNF655 | zinc finger protein 655 |
| 156236 | 0.892 | 1.23E-03 | 23378 | hCG24088.4 | KIAA0409 | KIAA0409 |
| 176580 | 0.892 | 1.23E-03 | 8545 | hCG1641589.3 | CGGBP1 | CGG triplet repeat binding protein 1 |
| 217443 | 0.892 | 1.23E-03 | 7846 | hCG2010765.1 | TUBA3 | null |
| 151956 | 0.891 | 1.26E-03 | 64651 | hCG1640077.4 | AXUD1 | AXIN1 up-regulated 1 |
| 135458 | 0.890 | 1.31E-03 | 10370 | hCG32930.3 | CITED2 | Cbp/p300-interacting transactivator, with Glu/Asp-rich carboxy-terminal domain, 2 |
| 190575 | 0.889 | 1.33E-03 | 126295 | hCG2039133 | LOC126295 | null |
| 177147 | 0.888 | 1.38E-03 | 7738 | hCG25305.4 | ZNF184 | zinc finger protein 184 (Kruppel-like) |
| 201581 | 0.885 | 1.52E-03 | 4938 | hCG40366.2 | OAS1 | 2',5'-oligoadenylate synthetase 1, 40/46kDa |
| 125937 | 0.885 | 1.53E-03 | 9249 | hCG1738619.1 | DHRS3 | dehydrogenase/reductase (SDR family) member 3 |
| 203869 | 0.882 | 1.63E-03 | 57820 | hCG40484.3 | CCNB1IP1 | cyclin B1 interacting protein 1 |
| 232123 | 0.882 | 1.66E-03 | 286075 | hCG1995000 | ZNF707 | zinc finger protein 707 |
| 130316 | 0.882 | 1.66E-03 | 9120 | hCG27295.4 | SLC16A6 | solute carrier family 16 (monocarboxylic acid transporters), member 6 |
| 108216 | 0.882 | 1.67E-03 | null | hCG2040856 | null | null |
| 221305 | 0.881 | 1.69E-03 | null | hCG1995620 | null | null |
| 178581 | 0.881 | 1.71E-03 | 387763 | hCG2042143 | LOC387763 | null |
| 111796 | 0.880 | 1.73E-03 | 151887 | hCG17537.5 | URB | null |
| 134459 | 0.880 | 1.73E-03 | 55647 | hCG33043.2 | RAB20 | RAB20, member RAS oncogene family |
| 213429 | 0.880 | 1.74E-03 | null | hCG1641325.2 | null | null |
| 671174 | 0.880 | 1.77E-03 | null | hCG1984640 | null | null |
| 304659 | 0.878 | 1.84E-03 | 9120\|440459 | hCG1748598.1\|hCG27295.4 | SLC16A6\|LOC440459 | solute carrier family 16 (monocarboxylic acid transporters), member 6 |
| 203863 | 0.876 | 1.97E-03 | 4783 | hCG30387.3 | NFIL3 | nuclear factor, interleukin 3 regulated |
| 209689 | 0.875 | 2.02E-03 | 8446 | hCG40732.3 | DUSP11 | dual specificity phosphatase 11 (RNA/RNP complex 1-interacting) |
| 104009 | 0.873 | 2.11E-03 | 2553 | hCG2002404.1 | GABPB2 | GA binding protein transcription factor, beta subunit 2 |
| 104119 | 0.872 | 2.15E-03 | 24147 | hCG27948.2 | FJX1 | four jointed box 1 (Drosophila) |
| 132403 | 0.872 | 2.16E-03 | 54494 | hCG2032602 | FLJ20010 | null |
| 670496 | 0.872 | 2.19E-03 | null | hCG27695.2 | null | null |
| 128130 | 0.872 | 2.19E-03 | 4853\|388677 | null | NOTCH2\|NOTCH2NL | Notch homolog 2 (Drosophila)\|Notch homolog 2 (Drosophila) N-terminal like |
| 133742 | 0.870 | 2.28E-03 | 8613 | hCG32470.2 | PPAP2B | phosphatidic acid phosphatase type 2B |
| 101333 | 0.869 | 2.32E-03 | 80345 | hCG1643757.2 | ZNF435 | zinc finger protein 435 |
| 160743 | 0.869 | 2.36E-03 | 677 | hCG20766.3 | ZFP36L1 | zinc finger protein 36, C3H type-like 1 |
| 135443 | 0.869 | 2.37E-03 | 9338 | hCG17969.3 | TCEAL1 | transcription elongation factor A (SII)-like 1 |
| 425760 | 0.867 | 2.45E-03 | null | hCG2038130 | null | null |
| 228255 | 0.867 | 2.48E-03 | null | hCG1813033.1 | null | null |
| 111356 | 0.867 | 2.48E-03 | 4792 | hCG21175.2 | NFKBIA | nuclear factor of kappa light polypeptide gene enhancer in B-cells inhibitor, alpha |
| 225045 | 0.867 | 2.48E-03 | 7050 | hCG1994498 | TGIF | TGFB-induced factor (TALE family homeobox) |
| 132027 | 0.867 | 2.48E-03 | 3606 | hCG39294.2 | IL18 | interleukin 18 (interferon-gamma-inducing factor) |
| 236293 | 0.867 | 2.50E-03 | null | hCG1787165.2 | null | null |
| 203549 | 0.866 | 2.52E-03 | 29107 | hCG22366.2 | NXT1 | NTF2-like export factor 1 |
| 133032 | 0.866 | 2.54E-03 | 64778 | hCG2023140 | FNDC3B | fibronectin type III domain containing 3B |

| | | | | | | |
|---|---|---|---|---|---|---|
| 131321 | 0.866 | 2.57E-03 | 7003 | hCG1994587 | TEAD1 | TEA domain family member 1 (SV40 transcriptional enhancer factor) |
| 222840 | 0.865 | 2.60E-03 | 254427 | hCG1644926.5 | C10orf47 | chromosome 10 open reading frame 47 |
| 173518 | 0.865 | 2.63E-03 | 10106 | hCG40163.3 | CTDSP2 | CTD (carboxy-terminal domain, RNA polymerase II, polypeptide A) small phosphatase 2 |
| 205016 | 0.864 | 2.67E-03 | 10135 | hCG1730556.4 | PBEF1 | pre-B-cell colony enhancing factor 1 |
| 195499 | 0.863 | 2.73E-03 | 2296 | hCG21926.2 | FOXC1 | forkhead box C1 |
| 194788 | 0.863 | 2.74E-03 | null | hCG38342.1 | null | null |
| 135086 | 0.863 | 2.76E-03 | 1543 | hCG40803.4 | CYP1A1 | cytochrome P450, family 1, subfamily A, polypeptide 1 |
| 131975 | 0.862 | 2.77E-03 | 3621 | hCG1774813.3 | ING1 | inhibitor of growth family, member 1 |
| 223116 | 0.860 | 2.92E-03 | 196792 | hCG2023314.1 | FAM24B | family with sequence similarity 24, member B |
| 125726 | 0.860 | 2.94E-03 | 6967\|6974 | hCG2010232.4 | TRGC2\|TRGV2 | T cell receptor gamma constant 2\|T cell receptor gamma variable 2 |
| 232524 | 0.860 | 2.94E-03 | null | hCG1997081 | null | null |
| 182589 | 0.859 | 3.02E-03 | 23189 | hCG2041742 | ANKRD15 | ankyrin repeat domain 15 |
| 191842 | 0.857 | 3.13E-03 | 26471 | hCG1745228.2 | P8 | null |
| 131347 | 0.857 | 3.17E-03 | 8349 | hCG1736972.2 | HIST2H2BE | histone 2, H2be |
| 190833 | 0.857 | 3.18E-03 | 79663 | hCG17209.3 | HSPBAP1 | HSPB (heat shock 27kDa) associated protein 1 |
| 186321 | 0.857 | 3.18E-03 | null | hCG1744064.1 | null | null |
| 161671 | 0.854 | 3.37E-03 | 11099 | hCG20029.2 | PTPN21 | protein tyrosine phosphatase, non-receptor type 21 |
| 152585 | 0.854 | 3.42E-03 | 124790 | hCG28073.2 | HEXIM2 | hexamthylene bis-acetamide inducible 2 |
| 108883 | 0.853 | 3.48E-03 | 122809 | hCG1790822.1 | SOCS4 | suppressor of cytokine signaling 4 |
| 156763 | 0.852 | 3.57E-03 | 10734 | hCG2024106.1 | STAG3 | stromal antigen 3 |
| 183778 | 0.851 | 3.66E-03 | 114789 | hCG96668.3 | SLC25A25 | solute carrier family 25 (mitochondrial carrier; phosphate carrier), member 25 |
| 208388 | 0.847 | 3.93E-03 | 5743 | hCG39885.3 | PTGS2 | prostaglandin-endoperoxide synthase 2 (prostaglandin G/H synthase and cyclooxygenase) |
| 142827 | 0.847 | 3.93E-03 | 1850 | hCG1784236.2 | DUSP8 | dual specificity phosphatase 8 |
| 207402 | 0.847 | 3.96E-03 | 79748\|594855 | hCG40812.2 | LMAN1L\|CPLX3 | lectin, mannose-binding, 1 like\|complexin 3 |
| 218433 | 0.847 | 4.00E-03 | 152048 | hCG1998594.2 | FLJ31715 | null |
| 104923 | 0.846 | 4.07E-03 | 3486 | hCG1735376.2 | IGFBP3 | insulin-like growth factor binding protein 3 |
| 122628 | 0.845 | 4.14E-03 | 113510 | hCG2027611 | HEL308 | null |
| 116962 | 0.845 | 4.14E-03 | 9570 | hCG1993582 | GOSR2 | golgi SNAP receptor complex member 2 |
| 522449 | 0.843 | 4.31E-03 | 158293 | hCG2038073 | C9orf10OS | chromosome 9 open reading frame 10 opposite strand |
| 124516 | 0.843 | 4.35E-03 | 79609 | hCG2013210 | C14orf138 | chromosome 14 open reading frame 138 |
| 147106 | 0.842 | 4.43E-03 | 81572 | hCG38820.2 | PDRG1 | p53 and DNA damage regulated 1 |
| 170626 | 0.840 | 4.56E-03 | 284422 | hCG1811317.1 | LOC284422 | null |
| 186148 | 0.840 | 4.57E-03 | 1477 | hCG39097.3 | CSTF1 | cleavage stimulation factor, 3' pre-RNA, subunit 1, 50kDa |
| 696887 | 0.840 | 4.60E-03 | 388638 | hCG1818789.1 | LOC388638 | null |
| 117752 | 0.839 | 4.71E-03 | 10435 | hCG2039452 | CDC42EP2 | CDC42 effector protein (Rho GTPase binding) 2 |
| 199946 | 0.836 | 5.01E-03 | 10443 | hCG32190.3 | PFAAP5 | null |
| 143102 | 0.835 | 5.11E-03 | 80824 | hCG27696.4 | DUSP16 | dual specificity phosphatase 16 |
| 147385 | 0.834 | 5.20E-03 | 84996 | hCG1818251.1 | C21orf119 | chromosome 21 open reading frame 119 |
| 158607 | 0.833 | 5.27E-03 | 23710 | hCG38151.3 | GABARAPL1 | GABA(A) receptor-associated protein like 1 |
| 136906 | 0.833 | 5.31E-03 | 55330 | hCG1640491.4 | CNO | cappuccino homolog (mouse) |
| 219582 | 0.833 | 5.33E-03 | 7378 | hCG2009894 | UPP1 | uridine phosphorylase 1 |
| 172733 | 0.832 | 5.45E-03 | 8507 | hCG37104.2 | ENC1 | ectodermal-neural cortex (with BTB-like domain) |
| 173155 | 0.831 | 5.46E-03 | 55056 | hCG2042213 | FLJ10038 | null |
| 126367 | 0.828 | 5.84E-03 | 220064 | hCG27990.3 | ORAOV1 | oral cancer overexpressed 1 |
| 179404 | 0.828 | 5.90E-03 | 8412 | hCG2028796.1 | BCAR3 | breast cancer anti-estrogen resistance 3 |
| 144028 | 0.827 | 5.98E-03 | 63924 | hCG201422.2 | CIDEC | cell death-inducing DFFA-like effector c |
| 216440 | 0.826 | 6.08E-03 | 56985 | hCG31075.4 | C17orf48 | chromosome 17 open reading frame 48 |
| 115022 | 0.826 | 6.11E-03 | 7779 | hCG24259.3 | SLC30A1 | solute carrier family 30 (zinc transporter), member 1 |
| 139142 | 0.825 | 6.18E-03 | 7775 | hCG1775917.2 | ZNF232 | zinc finger protein 232 |
| 236166 | 0.825 | 6.21E-03 | null | hCG1642089.3 | null | null |
| 114304 | 0.823 | 6.40E-03 | 3229 | hCG23347.2 | HOXC13 | homeobox C13 |
| 161660 | 0.823 | 6.41E-03 | 57494 | hCG2033535 | FAM80B | family with sequence similarity 80, member B |
| 196119 | 0.823 | 6.43E-03 | 338758 | hCG1820760.1 | LOC338758 | null |
| 211519 | 0.822 | 6.59E-03 | 7572 | hCG22510.3 | ZNF24 | zinc finger protein 24 (KOX 17) |
| 102937 | 0.821 | 6.66E-03 | 10957 | hCG33071.3 | PNRC1 | proline-rich nuclear receptor coactivator 1 |
| 167773 | 0.819 | 6.97E-03 | 92 | hCG19691.3 | ACVR2A | activin A receptor, type IIA |
| 193054 | 0.818 | 7.07E-03 | 285237 | hCG32497.3 | C3orf38 | chromosome 3 open reading frame 38 |
| 165993 | 0.817 | 7.15E-03 | 29092 | hCG1981007 | HSPC157 | null |
| 120260 | 0.817 | 7.15E-03 | 79630 | hCG1780608.3 | C1orf54 | chromosome 1 open reading frame 54 |
| 224381 | 0.817 | 7.22E-03 | 8969 | hCG1641458.3 | HIST1H2AG | histone 1, H2ag |
| 154858 | 0.817 | 7.23E-03 | 55904 | hCG2014496 | MLL5 | myeloid/lymphoid or mixed-lineage leukemia 5 (trithorax homolog, Drosophila) |
| 208606 | 0.816 | 7.36E-03 | 10018 | hCG15901.3 | BCL2L11 | BCL2-like 11 (apoptosis facilitator) |
| 157665 | 0.816 | 7.36E-03 | 51132 | hCG1993146 | RNF12 | ring finger protein 12 |
| 106892 | 0.814 | 7.59E-03 | null | hCG21032.3 | null | null |
| 229985 | 0.814 | 7.62E-03 | null | hCG1990334 | null | null |
| 218400 | 0.814 | 7.63E-03 | null | hCG2042547 | null | null |
| 223459 | 0.813 | 7.68E-03 | 8339 | hCG1640985.1 | HIST1H2BG | histone 1, H2bg |
| 215251 | 0.812 | 7.80E-03 | 466 | hCG1811506.3 | ATF1 | activating transcription factor 1 |
| 174543 | 0.812 | 7.87E-03 | 80176 | hCG22401.2 | SPSB1 | splA/ryanodine receptor domain and SOCS box containing 1 |
| 182228 | 0.810 | 8.15E-03 | 2624 | null | GATA2 | GATA binding protein 2 |
| 146761 | 0.809 | 8.33E-03 | 84981 | hCG1820470.1 | MGC14376 | null |
| 204791 | 0.808 | 8.42E-03 | null | hCG2040590 | null | null |
| 158499 | 0.808 | 8.43E-03 | 26150 | hCG16863.3 | RIBC2 | RIB43A domain with coiled-coils 2 |
| 168565 | 0.807 | 8.58E-03 | 55602 | hCG95823.3 | CARF | null |
| 125930 | 0.806 | 8.68E-03 | 163126 | hCG1820503.1 | CRI2 | CREBBP/EP300 inhibitor 2 |
| 106979 | 0.806 | 8.68E-03 | 81631 | hCG32374.3 | MAP1LC3B | microtubule-associated protein 1 light chain 3 beta |
| 130677 | 0.806 | 8.69E-03 | 3433 | hCG1643352.4 | IFIT2 | interferon-induced protein with tetratricopeptide repeats 2 |
| 170989 | 0.806 | 8.74E-03 | 7764 | hCG37127.2 | ZNF217 | zinc finger protein 217 |
| 188749 | 0.805 | 8.87E-03 | 55596 | hCG1811822.1 | ZCCHC8 | zinc finger, CCHC domain containing 8 |
| 218145 | 0.803 | 9.11E-03 | 10018 | hCG15901.3 | BCL2L11 | BCL2-like 11 (apoptosis facilitator) |
| 197310 | 0.803 | 9.21E-03 | 3475 | hCG15767.3 | IFRD1 | interferon-related developmental regulator 1 |
| 10715276 | 0.801 | 9.45E-03 | null | hCG39092.3 | null | null |
| 193960 | 0.801 | 9.50E-03 | 79621 | hCG28706.3 | FLJ11712 | null |
