# Supplementary material for: BIM and NOXA are mitochondrial effectors of TAF6δ-driven apoptosis
Source: Cell Death Dis. 2018 Jan 22;9(2):70. doi: 10.1038/s41419-017-0115-3 (PMC5833734; doi:10.1038/s41419-017-0115-3)
Supplement: Supplementary file 7 — Supplementary Table 4 [file 41419_2017_115_MOESM7_ESM.pdf]

Supplementary Table 4.

Pathway	Obs	Genes	Exp	Fold Enrichment	pValue
BH3-only proteins associate with and inactivate anti-apoptotic BCL-2 members	2	BIM<br>NOXA	0.08	25.94	2.81E-03
Activation of BIM and translocation to mitochondria	1	BIM	0.03	30.26	3.25E-02
↘ Activation of BH3-only proteins	2	BIM<br>NOXA	0.31	6.48	3.87E-02
Activation of NOXA and translocation to mitochondria	1	NOXA	0.04	22.70	4.31E-02
